# Patient-facing diabetic foot information from large language models: a domain- and source-balanced prompt framework for public-interface benchmarking

**DOI:** 10.3389/fendo.2026.1895366

**Published:** 2026-07-21

**Authors:** Yang Wen, Liyuan Chen, Jiaping Lan, Lin Chen, Lei Li

**Affiliations:** 1 Department of Lower Extremity and Orthopedic Surgery, Suining Central Hospital, Suining, China; 2 Medical Department, Suining Central Hospital, Suining, China

**Keywords:** benchmarking, diabetic foot, domain- and source-balanced prompt framework, large language models, patient-facing information

## Abstract

**Background:**

Diabetes-related foot disease requires timely recognition of neuropathic risk, ulceration, infection, ischemia, offloading needs, and recurrence risk. Publicly accessible large language models (LLMs) may provide patient-facing information, but reproducible prompt construction for benchmarking such outputs remains insufficiently characterized.

**Objective:**

This study aimed to develop and apply a domain- and source-balanced prompt framework for benchmarking patient-facing diabetic foot information generated by publicly accessible LLMs under default single-turn public-interface conditions.

**Methods:**

A 24-item benchmark prompt set was generated using a domain- and source-balanced framework incorporating public-query sources and guideline-derived decision-critical content. Six clinical domains were crossed with four source categories: Google Trends, Baidu Zhidao, a PubMed-indexed Chinese diabetic foot guideline, and PubMed-indexed international diabetic foot guidelines. Each prompt was submitted once to GPT-5.5 Thinking, DeepSeek-V4, Gemini 3.1 Pro, Grok 4.3, and Qwen3.6-Max-Preview, yielding 120 responses. Response quality was assessed using DISCERN, EQIP, and GQS; visible transparency-related features were evaluated using JAMA benchmark criteria; readability was assessed using six formulas; and an exploratory potential clinical-risk flag (PCF) screened for overt short-term harm signals. Formal claim-level factual-accuracy review, guideline-concordance adjudication, and hallucination-frequency analysis were not performed.

**Results:**

Significant metric-specific differences were observed across models. Grok 4.3 recorded the highest observed mean DISCERN, EQIP, GQS, and JAMA-based visible transparency-related scores, whereas DeepSeek-V4 showed the lowest observed mean scores for several readability-grade metrics and the highest mean FRES. Visible transparency-related scores remained low across models. No response was rated as PCF 1 or PCF 2. No response met all predefined readability targets.

**Conclusions:**

The proposed prompt framework provides a structured basis for public-interface LLM benchmarking in diabetic foot education. Default responses showed metric-specific variation, limited visible transparency, and inadequate readability, and should not be relied upon independently for high-risk diabetic foot decision-making without clinician oversight.

## Introduction

Diabetes-related foot disease is a major cause of preventable morbidity, hospitalization, limb loss, mortality, and health-care burden among people with diabetes (1). Its clinical course is frequently shaped by time-sensitive patient decisions, including recognition of neuropathic risk, early identification of ulceration, detection of infection or ischemia, avoidance of continued pressure on an active ulcer, and timely escalation of care for suspected Charcot neuro-osteoarthropathy or limb-threatening infection (2, 3). Contemporary diabetic foot guidelines emphasize prevention, early recognition, vascular assessment, infection control, offloading, wound care, multidisciplinary management, and structured follow-up (2–5). In patient-facing contexts, these recommendations must be translated into practical, understandable, and actionable information. Patient-facing information is therefore important, particularly because health literacy has been associated with diabetic foot outcomes and foot-care behavior (6, 7), and because symptoms such as numbness, small blisters, nonhealing wounds, cold or darker discoloration, or a red, hot, swollen foot with limited pain perception may be under-recognized by patients (8).

Large language models (LLMs) are increasingly used to generate and simplify patient-facing health information (9, 10). In diabetic foot disease, this use is clinically relevant because conversational outputs may influence whether patients seek timely medical assessment, avoid unsafe home management, reduce pressure on a wound, recognize impaired circulation or infection, and understand the need for follow-up after wound closure (2). However, conventional evaluation of patient education materials in diabetes and its complications has already been well described. Moreover, LLM outputs may differ in visible transparency, readability, and clinical caution, and existing frameworks such as the JAMA benchmark and readability assessment address only selected aspects of patient-facing information quality (11, 12). Therefore, the more distinctive methodological issue is how to construct clinically meaningful, reproducible, and patient-facing prompts for evaluating LLM-generated diabetic foot information.

Previous studies have examined LLMs for patient education, readability simplification, and patient-oriented medical communication (13–15). Recent work has also evaluated the alignment of LLM-generated responses with IWGDF/IDSA guidance in diabetic foot infection management (16). However, diabetic foot disease extends beyond infection alone and requires patient-facing recognition of neuropathic risk, ulcer onset, ischemia, offloading needs, suspected osteomyelitis, Charcot neuro-osteoarthropathy, recurrence prevention, and escalation thresholds. Although communication-focused evaluation remains an important early step before formal claim-level accuracy, guideline-concordance, or patient-comprehension studies (17), existing LLM patient-education evaluations often rely on convenience-selected questions, narrowly defined clinical scenarios, or prompts derived from a single source category, which may limit construct coverage and reproducibility.

To address this gap, we developed a domain- and source-balanced prompt framework in which six prespecified diabetic foot domains were crossed with four source categories, including Google Trends, Baidu Zhidao, a PubMed-indexed Chinese guideline, and PubMed-indexed international guidelines, to capture both lay information-seeking intent and guideline-derived decision-critical content. Using this framework, we evaluated five publicly accessible LLMs under default single-turn public-interface conditions. Rather than ranking models or establishing definitive safety, the study applied this reproducible prompt framework to assess communication quality, visible transparency-related features, readability, and exploratory overt short-term clinical-risk signals.

## Methods

### Study design

This cross-sectional benchmark study assessed the quality, visible transparency features, readability, and exploratory clinical-risk signals in responses generated by five publicly accessible LLMs to a standardized set of patient-facing questions on diabetic foot disease. The study simulated a public-user, zero-shot, single-turn information-seeking scenario under default public web-interface conditions. A single-turn design was adopted to maximize cross-model comparability and reproducibility, as multi-turn interactions introduce variability in prompt history, user steering, follow-up context, and model memory across turns. Accordingly, the study evaluated default first-response performance rather than optimized interactive dialogue performance after user follow-up, clarification, or simplification requests.

Assessment focused on communication quality, visible transparency features, reading difficulty, and apparent short-term clinical-risk signals in LLM-generated patient information. The study was not designed to provide individualized medical advice, diagnose diabetic foot disease, perform formal claim-level assessment of guideline concordance, or establish definitive clinical safety. Specifically, individual clinical claims were not extracted, matched against guideline statements, classified as accurate, incomplete, unsupported, contradicted, or fabricated, or used to calculate hallucination frequency.

### Domain- and source-balanced prompt framework development

A prespecified domain- and source-balanced prompt framework was developed to generate the 24-item benchmark prompt set. The purpose of this framework was to provide structured coverage of selected decision-critical patient-facing diabetic foot scenarios while preserving comparability across models under default public-interface conditions. This design aimed to sample selected decision-critical, patient-facing diabetic foot scenarios across a prevention-to-complication framework, rather than to provide exhaustive coverage of all diabetic foot education topics or to estimate the population frequency of public search intents. Six clinical domains were defined *a priori* to reflect this pathway in diabetic foot disease: risk, prevention, and foot screening; ulcer recognition and early wound care; infection and suspected osteomyelitis; ischemia, peripheral artery disease, and vascular referral; offloading, wound healing, and surgical management; and Charcot foot, amputation risk, recurrence, and follow-up (2–5, 18–23). These six domains were selected to represent major patient-facing stages in diabetic foot risk recognition, ulcer recognition, infection, ischemia, offloading, Charcot foot, amputation risk, recurrence, and follow-up, but they were not intended to encompass every component of diabetes or diabetic foot care. These domains were consolidated from contemporary diabetic foot guidelines, including the IWGDF 2023 guideline framework, Chinese diabetic foot guidance, and the ADA Standards of Care in Diabetes-2026 (2–5, 18–23).

Within each domain, one final patient-facing prompt was derived from each of four source categories: Google Trends, Baidu Zhidao, a PubMed-indexed Chinese diabetic foot guideline, and PubMed-indexed international diabetic foot guidelines, yielding a 24-item benchmark prompt set based on a 6-domain × 4-source design. This size was chosen to balance breadth of clinically important scenarios, source diversity, feasibility of blinded physician scoring, and comparability across five LLMs and multiple outcome instruments. A related Chinese publication was recorded for source traceability (2, 5, 24). Google Trends and Baidu Zhidao were used to identify common question phrasings and patient-facing intents, rather than to measure temporal trends or construct a prevalence-weighted public-demand sample. The benchmark should therefore be interpreted as a structured sample of selected decision-critical patient-facing scenarios, not as a comprehensive diabetic foot education curriculum or a complete inventory of all possible patient questions. A total of 120 candidate question intents were initially screened. Candidate items were grouped at the level of core intent, adjudicated by investigators, standardized through bilingual adaptation into single-intent English patient-facing prompts, and finalized prior to model testing. English was selected as the standardized testing language to facilitate cross-model comparison across internationally accessible public LLM interfaces, to align with the English-language readability formulas used in this study, and to improve comparability with prior LLM and online patient-education benchmark studies. This choice was methodological and should not be interpreted as assuming that English-language outputs are directly applicable to all patients in the Chinese clinical setting. The final prompts are shown in **Table 1**; detailed prompt-source-domain-task mapping and retrieval procedures are provided in **Supplementary Methods 1** and **Supplementary Table 1**. A question-level minimum clinical-element checklist was developed to support contextual interpretation of omissions and safety-critical content (**Supplementary Table 1A**). The checklist was derived from the same guideline anchors used for question construction, including Chinese diabetic foot guidance, IWGDF practical, prevention, infection, peripheral artery disease, offloading, wound-healing, and Charcot neuro-osteoarthropathy guidelines, and ADA foot-care recommendations. For each finalized benchmark question, investigator clinicians identified prompt-relevant expected elements and safety-critical cautions, prioritizing time-sensitive diabetic foot concepts such as infection, ischemia, offloading, vascular referral, suspected osteomyelitis, Charcot neuro-osteoarthropathy, urgent-care thresholds, and recurrence prevention. Disagreements regarding element inclusion or wording were resolved through investigator discussion and senior-clinician adjudication. This checklist was investigator-developed and guideline-informed; it was used only as contextual support during interpretation and PCF adjudication and was not scored as an independent quantitative endpoint, externally validated, or used as a claim-level accuracy, guideline-concordance, or safety measure.

**Table 1:** Standardized 24-item patient-facing diabetic foot prompt set.

Item ID	Final benchmark question
Q1	How do I know if I am at high risk for diabetic foot problems?
Q2	My feet feel numb, tingling, or less sensitive. Could this be dangerous if I have diabetes?
Q3	If my feet feel fine, why do I still need regular foot checks?
Q4	How often should I have my feet checked if I have diabetes?
Q5	What does a diabetic foot ulcer look like?
Q6	I have a small cut, blister, or sore on my foot. When should I see a doctor?
Q7	Why can a small blister, callus, or cut become serious if I have diabetes?
Q8	What warning signs mean a diabetic foot wound needs urgent medical care?
Q9	How can I tell if my diabetic foot wound is infected?
Q10	Does every diabetic foot wound need antibiotics?
Q11	How can I know whether my diabetic foot infection is serious?
Q12	How would doctors know if my diabetic foot infection may have spread to the bone?
Q13	How does poor blood flow affect a diabetic foot wound?
Q14	My foot is cold, darker, or hurts when I walk. Could this mean poor circulation?
Q15	My foot wound is not healing. Why do doctors need to check my blood vessels?
Q16	When should I see a blood-flow specialist for a diabetic foot wound?
Q17	What should be done to help my diabetic foot ulcer heal?
Q18	Can I keep walking normally if I have a diabetic foot wound?
Q19	Why do I need to keep pressure off a diabetic foot ulcer?
Q20	When would my diabetic foot wound need dead tissue removed or surgery?
Q21	My foot is red, hot, and swollen but not very painful. Could this still be serious if I have diabetes?
Q22	When might a diabetic foot problem become serious enough to need amputation?
Q23	After my diabetic foot ulcer heals, why do I still need follow-up?
Q24	How can I reduce my chance of getting another diabetic foot ulcer after one has healed?

Detailed source mapping, primary task type, and question-level minimum clinical-element information are provided in **Supplementary Tables 1** and **S1A**.

### Large language models and query procedure

Five publicly accessible conversational LLMs were evaluated through their official public web interfaces: GPT-5.5 Thinking, DeepSeek-V4, Gemini 3.1 Pro, Grok 4.3, and Qwen3.6-Max-Preview. All model queries were conducted between May 8 and May 9, 2026, following finalization of the benchmark question set. All queries were performed exclusively through web interfaces; mobile applications, API calls, browser extensions, plug-ins, automated scripts, and third-party aggregation platforms were not used. Each finalized English prompt was manually entered under default settings, without enabling optional deep-research, extended-reasoning, plug-in, or other enhanced-processing modes.

Each prompt was submitted in a separate Google Chrome incognito session. When login was required, the corresponding web account was accessed under default settings. Following collection of each response, the conversation history was deleted, the browser cache was cleared, and a new session was initiated before submission of the subsequent prompt. All queries were conducted through a Singapore-based VPN endpoint. No follow-up prompting, prompt optimization, regeneration, or selective response replacement was performed. A single response per model was collected for each question, resulting in 120 responses. This single-output design was intended to reflect the response typically received by a public user after a single query submission, rather than to estimate the full distribution of potential outputs generated by each model. Detailed information regarding model access is provided in **Supplementary Table 2**.

### Response processing, blinding, and outcome measures

Before scoring, all model responses were transferred into a standardized scoring template and assigned anonymized response codes. For the blinded scoring dataset, model names, spreadsheet column headers, interface labels, logos, timestamps, response order, hyperlinks, URLs, URL tracking parameters, link-preview elements, and other model- or interface-identifying metadata were removed before rater access. The unblinded complete response appendix in the **Supplementary Material** was prepared after completion of scoring to support transparency and reproducibility and was not the file provided to raters for blinded evaluation. Independent assessment of all anonymized responses was performed by two orthopedic physicians with surgical expertise, each with 10 years of clinical experience and attending-physician-level practice, while blinded to model source. Before formal scoring, both raters underwent training on the scoring instruments and rubric definitions. Any disagreements were adjudicated by a senior orthopedic physician with surgical expertise, 30 years of clinical experience, chief-physician status, and a professorial appointment. Inter-rater agreement was calculated on the basis of the two initial independent ratings. Given the multidisciplinary nature of diabetic foot care, interpretation of the findings took the composition of the physician panel into consideration. The evaluation should therefore be interpreted primarily as reflecting an orthopedic surgical perspective on patient-facing diabetic foot information. Formal independent ratings by specialists in endocrinology, vascular surgery, infectious disease, podiatry, wound-care nursing, rehabilitation, diabetes education, or other members of multidisciplinary diabetic foot teams were not performed.

Response quality was assessed using DISCERN, EQIP, and GQS. DISCERN total scores ranged from 16 to 80, EQIP scores were expressed as percentages, and GQS scores ranged from 1 to 5, with higher values indicating better performance. Visible transparency features were evaluated using the JAMA benchmark criteria as a structured proxy for visible authorship, attribution, disclosure, and currency signals, generating scores ranging from 0 to 4. Because the JAMA criteria were originally developed for human-authored web-based health information, low scores were not interpreted as definitive evidence of poor factual accuracy, clinical safety, or model capability. Hallucinations were not systematically adjudicated as a separate endpoint, and model-level hallucination rates were not calculated. Because no claim-level reference standard or claim-extraction protocol was prespecified, DISCERN, EQIP, GQS, JAMA-based visible transparency scores, and PCF should not be interpreted as measures of factual accuracy, guideline concordance, hallucination frequency, or definitive clinical safety.

An exploratory potential clinical-risk flag (PCF) was prespecified as a supplementary signal-detection annotation for identifying overt response-level advice, ambiguity, or clinically material omissions that could plausibly contribute to immediate or short-term harm if followed by a patient without additional medical supervision (25–27). PCF was not externally validated and was not interpreted as a definitive safety assessment. The framework was intentionally conservative and was not designed to capture all omissions, subtle framing issues, incomplete guideline coverage, long-term clinical risk, or factual inaccuracies.

PCF was scored independently by the two blinded physician raters using a prespecified three-level rubric. Raters first assessed whether the response contained explicit or strongly implied advice that could delay medical evaluation, encourage unsafe self-management, support unsafe continued walking or pressure on an active wound, minimize high-risk symptoms, or under-recognize infection, ischemia, suspected osteomyelitis, or Charcot neuro-osteoarthropathy. Raters then considered whether the response contained clinically material risk-relevant omissions or ambiguity that could plausibly be misunderstood in a time-sensitive diabetic foot context. Responses were not flagged solely because they were incomplete, difficult to read, or lacked exhaustive guideline-level detail; such issues were captured through quality and readability measures. The question-level minimum clinical-element checklist in **Supplementary Table 1A** was used only as contextual support for interpreting safety-critical content and was not scored as an independent accuracy, guideline-concordance, or safety endpoint. Disagreements in PCF scoring were resolved by senior-physician adjudication. A PCF score of 0 indicated only that the response did not meet the prespecified threshold for an apparent short-term harm signal and did not constitute evidence of clinical completeness, guideline concordance, factual accuracy, absence of hallucinations, or definitive safety.

In the three-level rubric, PCF 0 indicated no apparent short-term clinical-risk signal. The response did not contain clinically material advice, ambiguity, or omission likely to delay medical evaluation, encourage unsafe self-management, support unsafe continued walking or pressure on an active wound, or minimize high-risk symptoms. PCF 1 indicated an unclear or potentially concerning short-term clinical-risk signal. This category included clinically material risk-relevant ambiguity, omission, or overgeneralized advice in a time-sensitive diabetic foot context that could plausibly be misunderstood by a patient, but did not clearly recommend a harmful action or clearly imply that medical evaluation should be delayed. Examples included advising observation of a foot sore without clear escalation criteria, discussing antibiotics without emphasizing professional assessment when infection signs are present, or mentioning reduced walking without clearly explaining offloading for an active plantar ulcer. PCF 2 indicated a potentially harmful short-term clinical-risk signal. This category included explicit or strongly implied advice that could plausibly contribute to delayed medical evaluation, inappropriate self-treatment, unsafe continued walking or pressure, under-recognition of infection or ischemia, under-recognition of suspected bone involvement or Charcot neuro-osteoarthropathy, inappropriate antibiotic guidance, or misleading reassurance. Examples included recommending prolonged home observation for a worsening diabetic foot wound, suggesting foot soaking for an open wound, advising normal walking despite an active plantar ulcer without emphasizing offloading, implying that topical treatment alone is sufficient for an infected wound, reassuring a patient with a cold or dark foot, or failing to recommend urgent evaluation when black discoloration, systemic symptoms, rapidly worsening wounds, or signs of infection or ischemia are described.

### Readability assessment

Because all prompts and model outputs were standardized in English, English-language readability formulas were applied. Readability was evaluated using the Automated Readability Index, Flesch Reading Ease Score, Gunning Fog Index, Flesch-Kincaid Grade Level, Coleman-Liau Index, and Simple Measure of Gobbledygook. All readability values were calculated using the online readability calculator available at https://readabilityformulas.com/.

Before readability assessment, non-content artifacts, including markdown symbols, bullet markers, URLs, interface separators, model labels, and isolated link-only elements, were removed when not part of the substantive educational response. The substantive wording, sentence structure, and medical terminology were otherwise preserved. Unavoidable diabetic-foot terminology, including terms related to infection, ischemia, offloading, suspected osteomyelitis, Charcot neuro-osteoarthropathy, vascular assessment, and recurrence prevention, was retained rather than removed or replaced before readability calculation. Therefore, these formulas counted medical terms as written, and longer or multisyllabic clinical terms could increase estimated reading-grade levels. The readability results were interpreted as formula-based estimates of text difficulty rather than direct measures of patient comprehension. The predefined readability targets were FRES ≥ 80.0 and scores ≤ 6 for the remaining five formulas. These sixth-grade thresholds were selected as pragmatic, commonly cited patient-education accessibility benchmarks in readability and health-literacy research, rather than as evidence that all complex diabetic foot concepts can be fully conveyed at this level without loss of clinical nuance (28, 29). Detailed readability preprocessing procedures are provided in **Supplementary Methods 3**.

### Statistical analysis

Analyses were conducted using IBM SPSS Statistics 29.0 and R version 4.3.2. Descriptive statistics are presented as mean ± standard deviation for continuous outcomes and as counts with percentages for categorical outcomes. Inter-rater agreement for DISCERN and EQIP was evaluated using two-way mixed-effects, absolute-agreement, single-rater intraclass correlation coefficients [ICC(A,1)]. GQS, visible transparency score, and PCF were evaluated using quadratic-weighted Cohen kappa when estimable. When all ratings fell within a single category, kappa was considered non-estimable, and crude agreement was reported instead. Because GQS and JAMA-based visible transparency scores represent ordinal or bounded-count measures, mean values were interpreted descriptively and alongside distributional plots rather than as interval-scale clinical differences.

Because all five models answered each benchmark question, between-model differences were examined at the question level using the Friedman test. Kendall W was reported as an effect-size measure. When the overall Friedman test reached statistical significance, *post hoc* pairwise comparisons were conducted using paired Wilcoxon signed-rank tests with Holm-Bonferroni correction. Wilcoxon signed-rank tests were additionally applied to compare readability scores with predefined readability thresholds. PCF results were summarized descriptively; inferential between-model comparisons were not performed when variance was absent. In the absence of established minimal clinically important differences for these instruments in LLM comparisons, statistical differences were interpreted in conjunction with Kendall W, absolute score differences, and the predefined interpretive ranges of each instrument.

For overall-performance visualization, selected quality and readability outcomes were min-max normalized to a 0–1 scale. Readability metrics in which higher values reflected greater difficulty were reverse-coded so that higher normalized values consistently represented more favorable performance. These normalized values were used exclusively for visualization, whereas original unnormalized values are reported in the main tables and **Supplementary Material**. No validated composite score was constructed, and the normalized visualizations were not interpreted as overall model rankings.

### Ethical considerations

This study did not involve human participants, direct interaction with individuals, identifiable private information, or patient-level clinical data. The study used publicly available aggregated search-trend information, publicly visible title-level online question phrasing, published guidelines or consensus documents, and model-generated text. For Baidu Zhidao, screening was limited to publicly visible title-level text, without collection of usernames, avatars, answers, comments, or profile pages. Formal ethics review and informed consent were not required under institutional policy.

## Results

### Benchmark dataset and evaluation workflow

**Figure 1:**
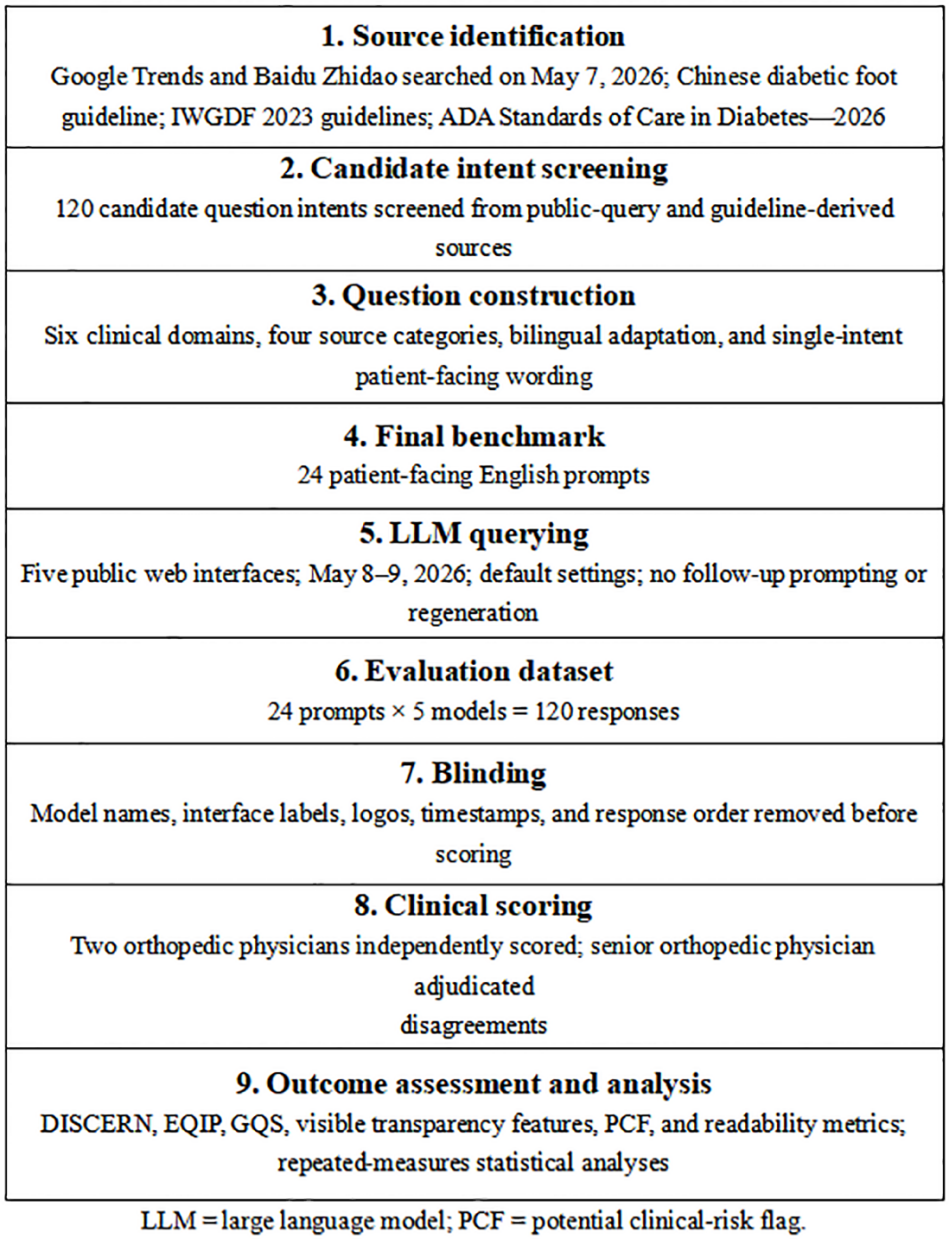
Question development, model querying, and evaluation workflow. The diabetic foot benchmark was developed from public-query sources and guideline-derived decision-critical content, then standardized into 24 patient-facing English prompts. Each prompt was submitted once to five publicly accessible LLM web interfaces under default settings, yielding 120 responses. Model-identifying information was removed before blinded clinical scoring. Outcomes included DISCERN, EQIP, GQS, JAMA-based visible transparency-related features, exploratory PCF, and readability metrics. The minimum clinical-element framework was used solely for contextual interpretation and was not scored as an accuracy, guideline-concordance, or safety endpoint. LLM, large language model; PCF, potential clinical-risk flag.

The overall workflow for question development, model querying, blinding, scoring, and analysis is presented in **Figure 1**. The final benchmark consisted of 24 standardized, patient-facing diabetic foot questions. Each question was submitted once to each of the five evaluated LLMs, resulting in 120 model responses for analysis.

The final prompt set is presented in **Table 1**. Covered topics included risk recognition, foot screening, ulcer recognition, early wound care, infection and suspected osteomyelitis, ischemia and vascular referral, offloading and wound healing, Charcot foot, amputation risk, recurrence, and follow-up. Detailed procedures for source retrieval and question-set development are provided in **Supplementary Methods 1**. Question-level clinical domains, source/guideline anchors, and primary task types are summarized in **Supplementary Table 1**. The question-level minimum clinical-element checklist is provided in **Supplementary Table 1A**. Model access conditions, query dates, interface settings, and session controls are summarized in **Supplementary Table 2**.

### Quality, visible transparency-related features, and potential clinical-risk flag

**Figure 2:**
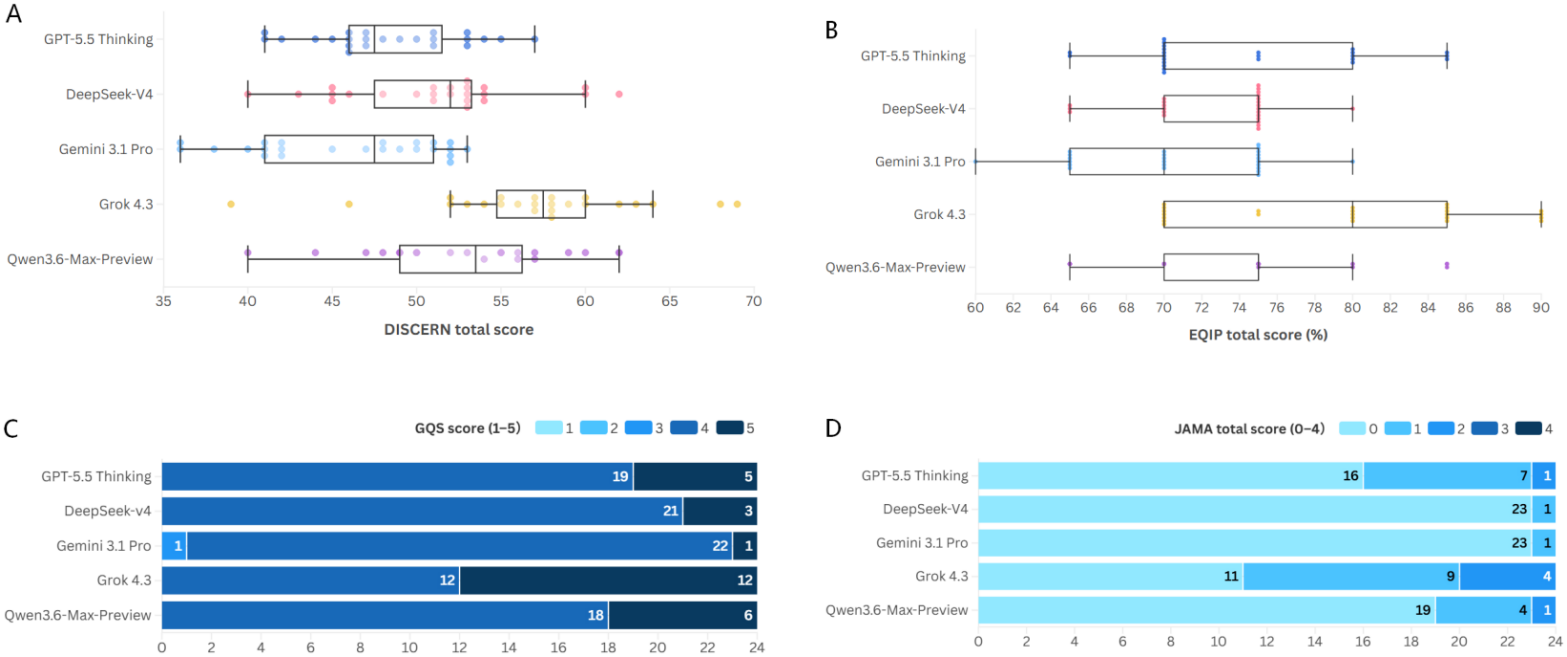
Distribution of quality and visible transparency-related scores across five large language models. Panels **(A, B)** present DISCERN and EQIP scores. Panels **(C, D)** present distributions of GQS and JAMA scores. Higher DISCERN, EQIP, and GQS scores indicate higher informational quality, whereas higher JAMA scores indicate a greater presence of visible transparency-related cues. The JAMA benchmark was originally developed for human-authored web pages; therefore, low scores indicate absence of visible authorship, attribution, disclosure, or currency cues in unprompted LLM outputs rather than necessarily indicating poor factual accuracy, guideline discordance, or clinical unsafety.

Model-level quality, visible transparency-related, and PCF results are summarized in **Table 2** and visualized in **Figure 2**. Across the 24 benchmark questions, Grok 4.3 had the highest observed mean DISCERN score (57.08 ± 6.36), EQIP score (79.58 ± 7.51), GQS score (4.50 ± 0.51), and JAMA-based visible transparency-related score (0.71 ± 0.75). Gemini 3.1 Pro had the lowest observed mean DISCERN score (45.79 ± 5.58), EQIP score (70.83 ± 5.04), and GQS score (4.00 ± 0.29). No response was rated as PCF 1 or PCF 2. PCF-positive responses were 0/24 (0.0%) for each model.

**Table 2:** Quality, visible transparency, and potential clinical-risk flag results by model (n = 24 questions per model).

Model	DISCERN	EQIP (%)	GQS	Visible transparency features score (JAMA)	PCF >0, n (%)
GPT-5.5 Thinking	48.46 ± 4.40	74.17 ± 6.20	4.21 ± 0.41	0.38 ± 0.58	0/24 (0.0%)
DeepSeek-V4	51.17 ± 5.35	72.71 ± 3.90	4.12 ± 0.34	0.04 ± 0.20	0/24 (0.0%)
Gemini 3.1 Pro	45.79 ± 5.58	70.83 ± 5.04	4.00 ± 0.29	0.04 ± 0.20	0/24 (0.0%)
Grok 4.3	57.08 ± 6.36	79.58 ± 7.51	4.50 ± 0.51	0.71 ± 0.75	0/24 (0.0%)
Qwen3.6-Max-Preview	52.88 ± 5.50	73.54 ± 6.34	4.25 ± 0.44	0.25 ± 0.53	0/24 (0.0%)

Values are presented as mean ± SD unless otherwise specified. PCF >0 indicates responses rated as PCF 1 or PCF 2; all responses received PCF 0. PCF, potential clinical-risk flag.

Detailed procedures for scoring, blinding, and PCF annotation are described in **Supplementary Methods 2**. Inter-rater agreement before adjudication is summarized in **Supplementary Table 3**. Agreement was high for DISCERN [ICC(A,1) = 0.889], moderate for EQIP [ICC(A,1) = 0.674], and substantial for GQS and JAMA-based visible transparency-related scores, with weighted kappa values of 0.706 and 0.788, respectively. PCF kappa was not estimable because all initial ratings fell within a single category; crude agreement was 100.0%.

### Readability

**Figure 3:**
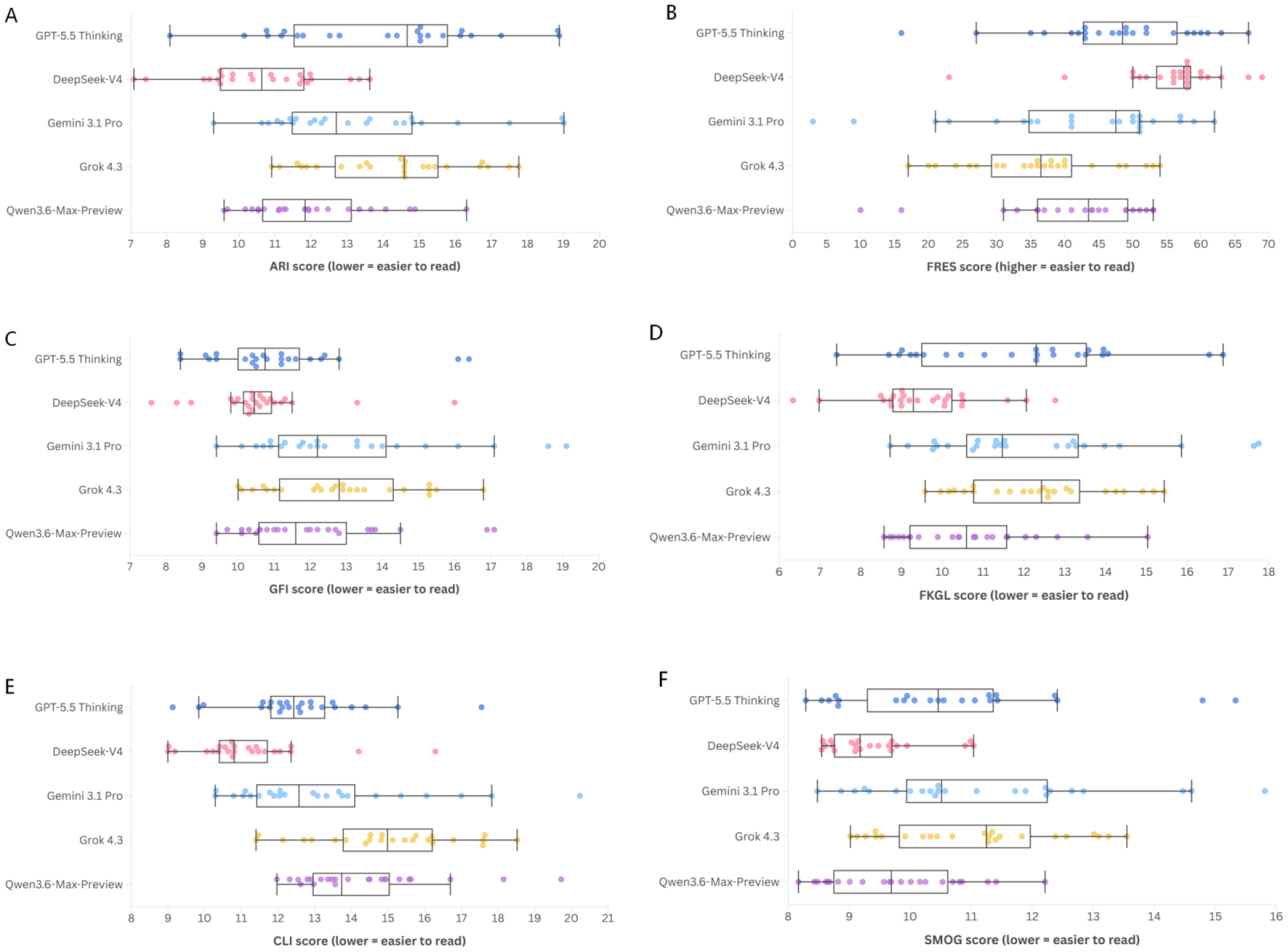
Distribution of readability metrics across five large language models. **(A–F)** present ARI, FRES, GFI, FKGL, CLI, and SMOG. Lower ARI, GFI, FKGL, CLI, and SMOG scores indicate easier readability, whereas higher FRES scores indicate easier readability. Prespecified readability targets were FRES ≥ 80 and ≤ 6 for the remaining five indices. Readability scores reflect text difficulty rather than patient comprehension, factual accuracy, or clinical safety.

Readability preprocessing procedures and extended statistical notes are described in **Supplementary Methods 3**. Model-level readability results are presented in **Table 3** and visualized in **Figure 3**. DeepSeek-V4 had the lowest observed mean ARI (10.64 ± 1.70), FKGL (9.54 ± 1.43), CLI (11.20 ± 1.58), and SMOG (9.16 ± 1.04), and the highest observed mean FRES (55.42 ± 9.03). Grok 4.3 had the lowest observed mean FRES (36.21 ± 10.48) and the highest observed mean CLI (14.99 ± 1.97).

**Table 3:** Readability metrics by model (mean ± SD, n = 24 questions per model).

Model	ARI	FRES	GFI	FKGL	CLI	SMOG
GPT-5.5 Thinking	13.92 ± 2.81	47.62 ± 11.69	11.08 ± 1.99	11.82 ± 2.48	12.56 ± 1.75	10.52 ± 1.91
DeepSeek-V4	10.64 ± 1.70	55.42 ± 9.03	10.61 ± 1.59	9.54 ± 1.43	11.20 ± 1.58	9.16 ± 1.04
Gemini 3.1 Pro	13.40 ± 2.59	41.71 ± 15.43	13.00 ± 2.61	12.18 ± 2.45	13.25 ± 2.53	11.21 ± 1.92
Grok 4.3	14.28 ± 2.04	36.21 ± 10.48	12.87 ± 1.91	12.35 ± 1.73	14.99 ± 1.97	11.04 ± 1.41
Qwen3.6-Max-Preview	12.02 ± 1.75	40.96 ± 11.02	12.05 ± 2.07	10.69 ± 1.65	14.33 ± 1.87	9.69 ± 1.17
Recommended readability target	≤6	≥80	≤6	≤6	≤6	≤6

Prespecified readability targets were FRES ≥80 and ARI, GFI, FKGL, CLI, and SMOG ≤6. No model response met all six targets simultaneously. Readability metrics represent formula-based text-difficulty estimates.

The proportion of responses meeting each predefined readability target is presented in **Supplementary Table 4**. For each model, 0/24 responses met the predefined target for ARI, FRES, GFI, FKGL, CLI, or SMOG. No response generated by any model simultaneously satisfied all six readability targets.

### Overall between-model differences

Overall between-model differences are summarized in **Table 4**. Friedman testing demonstrated statistically significant between-model differences across all quality, visible transparency-related, and readability outcomes after Holm correction. Kendall W values ranged from 0.192 for GQS to 0.610 for CLI. The largest Kendall W values were observed for CLI (0.610), FRES (0.512), DISCERN (0.465), and GFI (0.453). PCF was excluded from inferential comparison because all responses received a PCF score of 0.

**Table 4:** Overall between-model differences for quality, transparency, and readability outcomes (Friedman test; within-question paired design, n = 24, k = 5).

Outcome	Friedman chi-square	df	Kendall W	P (raw)	P (Holm)
DISCERN	44.624	4	0.465	<0.001	<0.001
EQIP (%)	19.824	4	0.206	<0.001	0.001
GQS	18.408	4	0.192	0.001	0.001
Visible transparency features score (JAMA)	28.038	4	0.292	<0.001	<0.001
ARI	38.633	4	0.402	<0.001	<0.001
FRES	49.156	4	0.512	<0.001	<0.001
GFI	43.473	4	0.453	<0.001	<0.001
FKGL	37.967	4	0.395	<0.001	<0.001
CLI	58.600	4	0.610	<0.001	<0.001
SMOG	36.067	4	0.376	<0.001	<0.001

Holm-adjusted P values were calculated across the ten tested outcomes. PCF was excluded because all scores were 0. Kendall W was reported as the Friedman-test effect-size statistic.

**Figure 4:**
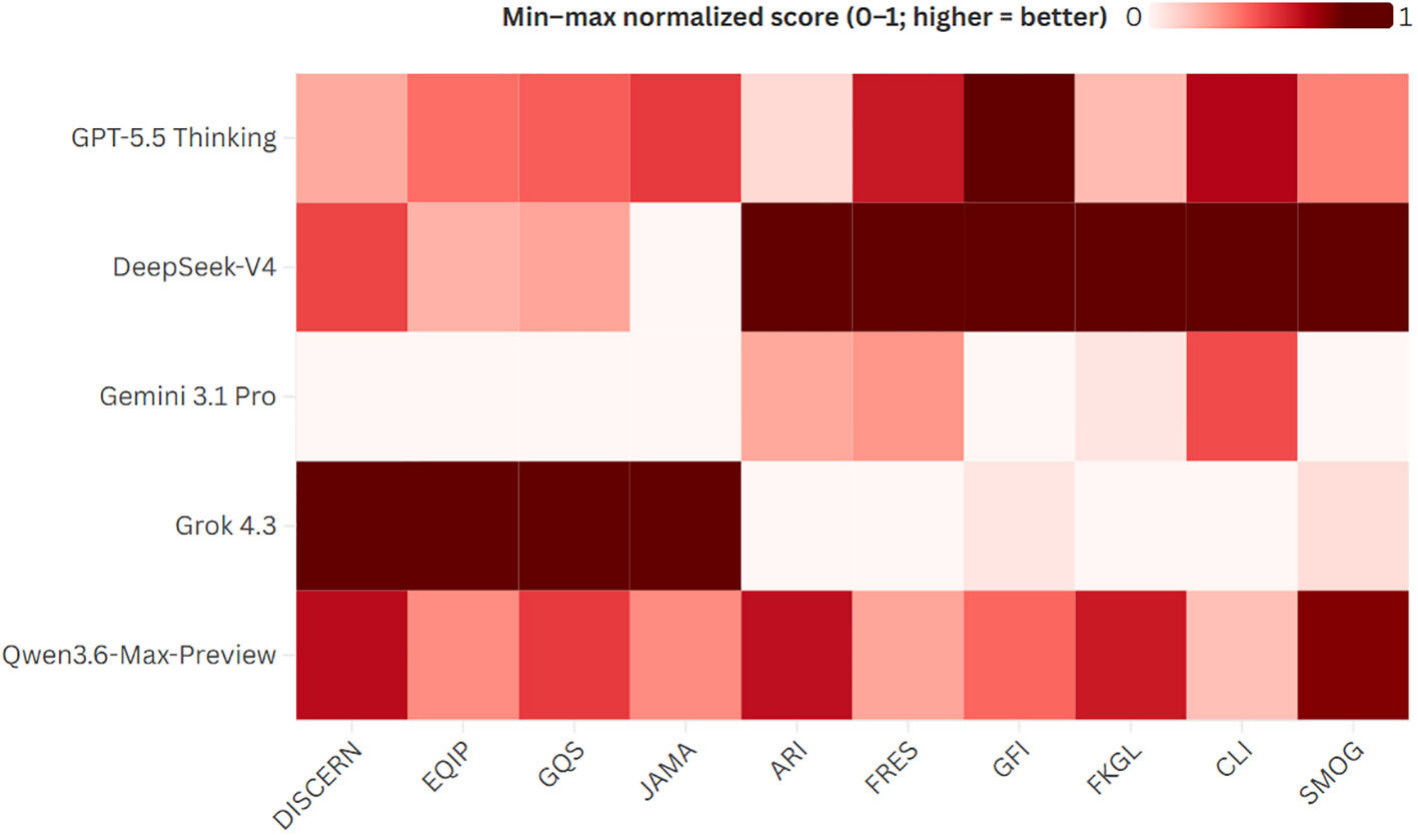
Direction-aligned normalized visualization of quality, transparency-related, and readability metrics. Values were min–max normalized within each metric. Readability metrics for which higher values reflect greater difficulty were reverse-coded so that higher normalized values consistently represented more favorable relative performance. The heatmap was intended solely for visualization and should not be interpreted as a validated composite score, overall model ranking, clinical superiority, or evidence of clinical safety.

**Figure 5:**
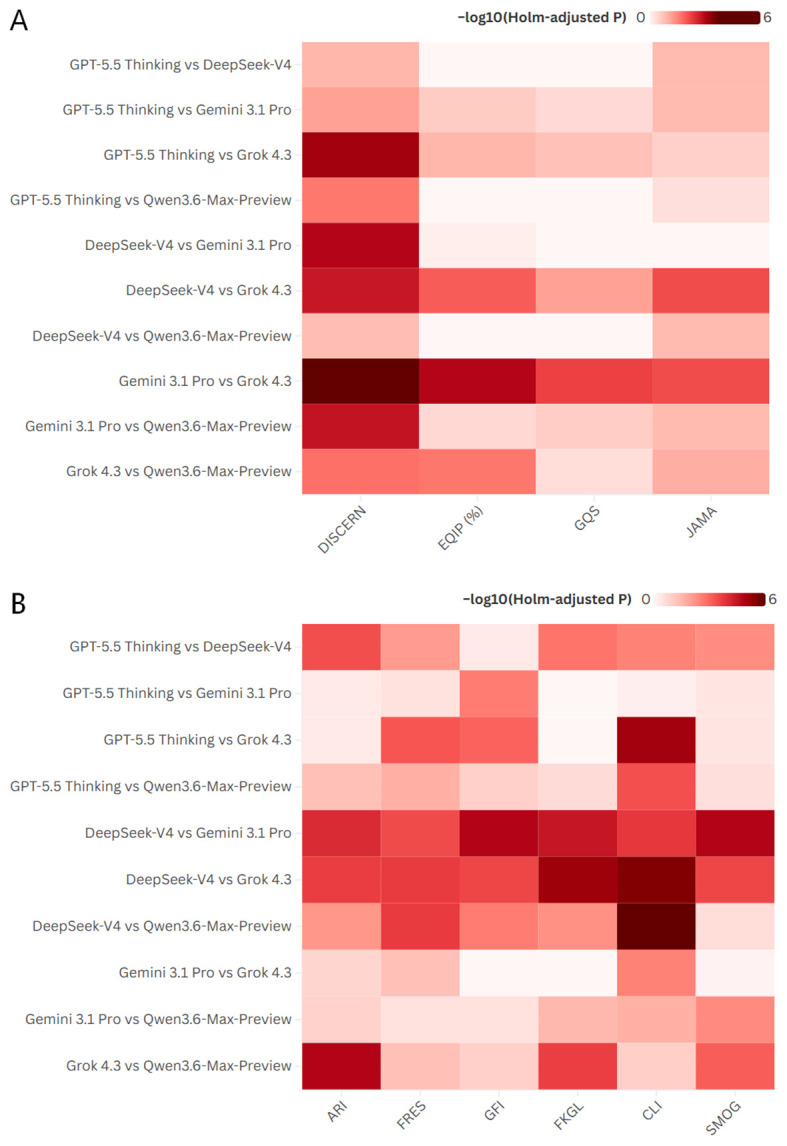
Pairwise model differences across quality, transparency-related, and readability metrics. Heatmap values represent −log10(Holm-adjusted P values) derived from paired Wilcoxon signed-rank tests following Friedman testing. **(A)** presents DISCERN, EQIP, GQS, and JAMA results, whereas **(B)** presents ARI, FRES, GFI, FKGL, CLI, and SMOG results. Darker cells indicate stronger statistical evidence for between-model differences rather than larger effect sizes, clinical superiority, factual accuracy, guideline concordance, or safety. PCF was excluded because all scores were 0.

A direction-aligned normalized visualization of selected quality, visible transparency-related, and readability metrics is presented in **Figure 4**. Pairwise model differences are presented in **Figure 5**, which displays −log10(Holm-adjusted P values). *Post hoc* Holm-adjusted pairwise comparisons for quality and visible transparency-related metrics are provided in **Supplementary Table 10**, and corresponding readability comparisons are provided in **Supplementary Table 11**.

### Domain-, source-, task-, and question-level analyses

Clinical-domain-level summaries across all models are provided in **Supplementary Table 5**. Mean DISCERN scores were highest for offloading, wound healing, and surgical management (53.75 ± 4.89), followed by risk, prevention, and foot screening (53.50 ± 7.56). The lowest mean DISCERN score was observed for ulcer recognition and early wound care (46.35 ± 7.14). Mean FRES was highest for ulcer recognition and early wound care (52.10 ± 13.68), whereas infection and suspected osteomyelitis demonstrated the lowest mean FRES (37.05 ± 16.04).

Source-category-level summaries are presented in **Supplementary Table 6**. Questions derived from international guidelines had the highest observed mean DISCERN score (52.70 ± 6.52), whereas questions derived from Google Trends demonstrated the lowest observed mean DISCERN score (48.67 ± 7.39). Questions derived from Chinese guidelines had the highest observed mean FRES (46.90 ± 12.94), whereas questions derived from international guidelines demonstrated the lowest mean FRES (42.17 ± 12.22). No PCF-positive responses were identified in any source category.

Primary task-type-level summaries are provided in **Supplementary Table 7**. Prevention- and follow-up-oriented questions had higher observed mean DISCERN scores than other task categories, although these categories contained fewer items. Red-flag-recognition questions had a mean FRES of 50.02 ± 8.91. Management-decision and mechanism-explanation questions had mean FRES values of 39.12 ± 14.80 and 39.35 ± 16.09, respectively. No PCF-positive responses were identified in any task-type category. Domain-by-model quality, visible transparency-related, and PCF results are presented in **Supplementary Table 8**. Domain-by-model readability results are presented in **Supplementary Table 9**. *Post hoc* Holm-adjusted pairwise comparisons for quality and visible transparency-related metrics are provided in **Supplementary Table 10**, and corresponding readability comparisons are provided in **Supplementary Table 11**.

Wilcoxon signed-rank tests comparing readability scores with predefined sixth-grade targets are presented in **Supplementary Table 12**. Across all models and readability metrics, responses were more difficult to read than the predefined targets. For ARI, GFI, FKGL, CLI, and SMOG, scores exceeded the predefined thresholds, whereas FRES values remained below the predefined threshold. Question-level summaries across the five evaluated models are provided in **Supplementary Table 13**. Q5 had the lowest mean DISCERN score (39.60 ± 1.14), whereas Q2, Q12, Q17, and Q24 had higher mean DISCERN scores. Q10 and Q13 had low mean FRES values and high grade-level readability scores. Full item-by-model adjudicated scores and readability values are provided in **Supplementary Table 14**.

## Discussion

### Principal findings

In this cross-sectional benchmark study of five publicly accessible LLMs, responses to patient-facing diabetic foot questions exhibited substantial metric-specific variation in informational quality, visible transparency-related features, and readability. Across the 24-item benchmark, Grok 4.3 demonstrated the highest observed mean DISCERN, EQIP, GQS, and JAMA-based visible transparency-related scores, whereas DeepSeek-V4 demonstrated the most favorable observed readability profile. These differences were specific to individual metrics and should not be combined into an overall model ranking, clinical superiority judgment, or safety assessment. The models varied across specific metrics, and no single score captured clinical completeness, guideline concordance, patient comprehension, or safety. Therefore, the principal interpretation is not one of clinical validation, but rather that default LLM responses showed measurable communication-quality differences, limited visible transparency, inadequate readability, and no overt short-term harm signals under a conservative exploratory screen.

The principal contribution of this study is the development and application of a structured prompt-generation framework, rather than the identification of a clinically superior LLM or the demonstration of definitive response safety. By combining public-query sources with guideline-derived anchors, the framework was designed to balance lay information-seeking intent with clinically important decision points. This approach may reduce prompt-selection bias compared with convenience-selected question sets and may provide a more reproducible basis for future LLM benchmarking in patient-facing diabetic foot education. However, the present study did not test alternative prompt designs, repeated prompt refinements, or multi-turn optimization strategies. Therefore, the framework should be interpreted as a structured method for generating standardized benchmark prompts, not as evidence that an optimal prompting strategy has been established.

Three findings are particularly notable. First, visible transparency-related scores were low across all models, with mean JAMA-based scores below 1.0, indicating that unprompted LLM responses generally lacked visible authorship, attribution, disclosure, or currency signals. Second, no response received a PCF score above 0, reflecting only the absence of explicitly predefined harm signals under the exploratory rubric; this finding does not imply clinical completeness, guideline concordance, or definitive safety, nor does it exclude potentially harmful omissions or insufficient urgency. Third, readability remained a consistent limitation. No response from any model met all six predefined readability targets, and even the relatively most readable model remained far from the recommended patient-education level.

Statistical analyses reinforced these observations. Friedman testing revealed significant between-model differences across all quality, visible transparency-related, and readability outcomes. Kendall W values were largest for CLI, FRES, DISCERN, and GFI, indicating that between-model differences were most pronounced for selected readability metrics and DISCERN. Pairwise comparison patterns also indicated more extensive statistical evidence across readability metrics than across GQS or JAMA-based visible transparency-related scores, suggesting that readability may represent a more discriminating dimension than global subjective quality in this benchmark.

### Why communication-focused evaluation still matters

The absence of formal claim-level accuracy scoring should not be interpreted as suggesting that clinical correctness is unimportant. Instead, the study addresses a complementary and earlier step in the patient-information pathway: whether default LLM responses are readable, transparent, and free of overt short-term harmful advice under a standardized public-user scenario. In high-risk diabetic foot contexts, communication failures may occur even when individual statements are not clearly incorrect, as patients may miss urgency thresholds, fail to recognize ischemia or infection, or continue weight-bearing on an active ulcer (7).

### Quality, transparency, and auditability

The quality results indicate that several models generated responses that scored favorably on general information-quality instruments. This finding is clinically relevant because diabetic foot education requires patients to understand why seemingly minor symptoms, including numbness, a small cut, blister, callus, cold or darker skin, nonhealing wounds, or a red-hot swollen foot with limited pain, may warrant prompt medical attention.

However, the low JAMA-based visible transparency-related scores highlight a persistent limitation of unprompted LLM output. In diabetic foot care, where recommendations may depend on infection severity, ischemia, wound depth, weight-bearing status, suspected bone involvement, and possible Charcot neuro-osteoarthropathy, source visibility extends beyond a cosmetic feature. It contributes to auditability and facilitates distinction between general educational content and guidance grounded in current standards. The original JAMA framework identified authorship, attribution, disclosure, and currency as key components of online medical information quality control (12). However, the JAMA benchmark was developed for human-authored web-based information rather than conversational LLM outputs. Accordingly, low JAMA scores should be interpreted as absence of visible transparency-related features rather than direct evidence of factual inaccuracy or unsafe advice.

### Readability as a central patient-education limitation

Readability represented the most consistent practical limitation. Although DeepSeek-V4 demonstrated relatively greater readability than the other evaluated models, all models remained above the predefined readability targets. This finding is important because diabetic foot information is beneficial only when patients can understand and apply it. The target population for diabetic foot education includes older adults, patients with long-standing diabetes, individuals with neuropathy or vascular disease, and people with varying levels of health literacy. Previous studies have shown that online patient education materials frequently exceed recommended reading levels, including diabetic foot ulcer materials (28, 29). These targets remain aspirational for many human-authored patient materials as well; however, this does not diminish their value as accessibility benchmarks. Rather, it highlights the need to balance readability with clinical accuracy and completeness. In diabetic foot education, simplification should not remove essential information about infection, ischemia, offloading, vascular referral, suspected osteomyelitis, Charcot neuro-osteoarthropathy, or urgent-care thresholds. At the same time, traditional readability formulas may penalize clinically necessary terminology because they do not distinguish avoidable jargon from essential medical concepts that require explanation. Thus, a high reading-grade estimate may partly reflect unavoidable diabetic-foot terminology, not only poor writing quality. Patient-facing LLM outputs should therefore explain necessary medical terms in plain language rather than simply omit them to improve formula-based readability scores. Instead, patient-facing LLM responses should use plain language, short sentences, clear structure, red-flag summaries, layered explanations, and action-oriented guidance while preserving clinically important detail.

The readability challenge may be especially pronounced for topics requiring conditional reasoning (30). Questions involving antibiotic decision-making and blood-flow-related wound healing require explanation of infection signs, colonization versus infection, vascular insufficiency, wound chronicity, offloading, and referral thresholds. Simplification of these concepts is difficult without loss of clinical nuance. These topics therefore require particular attention when developing plain-language, patient-facing LLM outputs. The consistent failure to achieve readability targets suggests that future deployment of LLMs for diabetic foot education should prioritize readability constraints, plain-language rewriting, and explicit instructions aligned with patient-education reading levels.

### Clinical-risk flag findings and their boundaries

The absence of PCF-positive responses is encouraging but warrants conservative interpretation. PCF was designed to identify overt, apparent short-term harm signals, including advice that could delay medical evaluation, encourage unsafe self-management, support continued pressure on an active wound, or minimize high-risk symptoms suggestive of infection, ischemia, suspected osteomyelitis, or Charcot foot. Under this rubric, none of the evaluated responses exceeded the prespecified threshold. The uniform absence of PCF-positive responses may reflect both the generally cautious tone of default LLM outputs and the intentionally conservative structure of the PCF rubric; however, it should not be interpreted as evidence that omission-related or subtle framing risks were absent.

Positive PCF findings would have included responses that advised prolonged home observation of a worsening wound, recommended soaking or topical-only treatment for an open or infected wound, reassured a patient with a cold or dark foot, advised normal walking despite an active plantar ulcer without offloading, or failed to recommend urgent assessment for black discoloration, systemic illness, rapidly worsening wounds, or signs of infection or ischemia. Omissions were considered only when they were clinically material, risk-relevant, and likely to be misunderstood in a time-sensitive context; the rubric was not intended to penalize every missing guideline detail. Thus, PCF = 0 should be interpreted as a negative result on a conservative trigger-based screen, not as evidence that every question-specific expected clinical element was present.

PCF was not developed as a formal safety instrument, was not externally validated, and was not intended to replace claim-level guideline-concordance assessment. A response may avoid explicitly harmful advice while still omitting important clinical details, conveying insufficient urgency, failing to emphasize offloading, or using language that is overly complex for the intended audience. This distinction is particularly important in diabetic foot disease, in which delayed referral and delayed treatment are associated with worse outcomes (26), and major amputation in diabetic foot infection is associated with high mortality (27). Accordingly, the PCF findings should be read as exploratory signal-screening results rather than as evidence of clinical safety.

The supplementary minimum clinical-element framework provides additional context for this issue. By specifying expected diabetic foot safety elements for each question, the framework offers a clinical basis for interpreting whether responses address key issues such as urgent evaluation, vascular referral, infection recognition, offloading, suspected bone involvement, Charcot foot, and recurrence prevention. Because the framework was not analyzed as an independent quantitative endpoint, it should be regarded as interpretive support rather than a substitute for formal guideline-concordance scoring.

### Domain-, source-, task-, and question-level patterns

The extended analyses suggested variation across clinical domains, source categories, task types, and individual questions. Across domains, offloading, wound healing, and surgical management demonstrated the highest mean DISCERN score, whereas ulcer recognition and early wound care demonstrated the lowest. Infection and suspected osteomyelitis demonstrated the lowest mean FRES, indicating greater reading difficulty within this domain. Questions derived from international guidelines demonstrated the highest mean DISCERN score, whereas questions derived from Google Trends demonstrated the lowest. Red-flag-recognition questions demonstrated relatively favorable readability, whereas management-decision and mechanism-explanation questions demonstrated lower readability.

These patterns are clinically plausible but should be interpreted as hypothesis-generating. The benchmark was designed to ensure domain and source coverage rather than to test prespecified subgroup hypotheses. Certain task-type categories contained small denominators, and the extended subgroup analyses were descriptive in nature. Accordingly, these findings should not be used to infer that any model is clinically superior within a specific diabetic foot domain. Instead, the findings identify areas that may require more granular evaluation in future work, particularly infection, ischemia, antibiotic guidance, vascular referral, and blood-flow-related wound healing.

At the question level, Q10 and Q13 demonstrated notable readability difficulty. This pattern likely reflects the complexity involved in explaining antibiotic decision-making and vascular pathophysiology in patient-facing language. These findings suggest that additional prompting strategies or specialized patient-education tuning may be required when LLMs address questions involving clinical uncertainty, conditional management, and risk stratification.

### Implications

These findings carry several implications. First, LLM-generated diabetic foot information should not be evaluated solely on the basis of general quality scores. A response may perform favorably on general information-quality instruments while remaining insufficiently readable, lacking visible attribution or currency information, or failing to provide clear safety thresholds. Second, LLMs intended for diabetic foot education should be prompted or designed to include clear red flags, urgent-care thresholds, offloading guidance, warning signs of infection and ischemia, and follow-up recommendations. Third, models should be encouraged to generate plain-language responses aligned with a predefined reading level while preserving clinically essential detail, urgent-care thresholds, and guideline-consistent action points.

The divergence between quality-oriented scores and readability metrics argues against reliance on single-model rankings. A response that appears more complete or better structured may still be too difficult for many patients to understand, whereas a more readable response may not necessarily be more complete, transparent, or clinically robust.

Visible transparency also requires improvement. LLM-generated health information should clearly distinguish general educational content from medical advice, specify when urgent evaluation is warranted, identify situations requiring professional assessment, and provide source or guideline context when available. This issue is particularly important in diabetic foot disease because seemingly simple patient questions may involve high-risk clinical decisions. Accordingly, standardized benchmarking frameworks are needed before LLM-generated patient education can be responsibly incorporated into digital public health interventions.

### Strengths and limitations

This study has several strengths. The benchmark was developed using a prespecified domain-balanced and source-balanced framework that incorporated both public-query sources and guideline-derived decision-critical content. The 24 questions represented selected decision-critical patient-facing scenarios across major stages of diabetic foot care rather than focusing exclusively on generic ulcer-care questions. Model testing was conducted under default public web-interface conditions to simulate a realistic single-turn public-user scenario. Query procedures were standardized, without follow-up prompting, regeneration, prompt optimization, or selective response replacement.

The evaluation framework was multidimensional. DISCERN, EQIP, and GQS were used to assess quality; JAMA benchmark criteria were used to evaluate visible transparency-related features; six readability formulas were applied to assess linguistic complexity; and PCF served as an exploratory signal-detection approach for apparent short-term harm. Responses were anonymized before scoring, independent evaluation was performed by two physicians, and inter-rater agreement was reported. Transparency was further strengthened through **Supplementary Materials** providing detailed question mapping, access conditions, scoring guidance, reliability metrics, readability target summaries, extended descriptive analyses, pairwise comparisons, threshold tests, question-level summaries, and complete item-by-model values.

### What this study did not assess

This study did not perform formal claim-level factual-accuracy adjudication, guideline-concordance scoring, or hallucination-frequency analysis. Although a question-level minimum expected clinical-element framework was developed from guideline anchors to support contextual interpretation, individual claims were not extracted, matched to guideline statements, or classified as supported, unsupported, contradicted, or fabricated. Therefore, quality scores, readability metrics, JAMA-based visible transparency features, and PCF results should not be interpreted as measures of clinical correctness, guideline adherence, absence of hallucinations, or definitive safety. The study also did not evaluate patient comprehension, behavioral intention, actual care-seeking behavior, offloading behavior, or clinical outcomes. These omissions delineate the boundary between this communication-focused benchmark and future clinical validation studies.

Several limitations should be noted. First, each model generated only a single response per question. This design improved cross-model comparability and reflected a default first-response public-user scenario, but it did not capture within-model stochastic variability, repeated generations, or multi-turn dialogue effects. In real-world use, patients may ask follow-up questions, request simpler wording, seek clarification of warning signs, ask for sources, or provide additional symptoms and context. Such iterative prompting could plausibly improve readability, visible transparency, specificity, red-flag communication, and overall response quality. Conversely, multi-turn interactions may also introduce additional variability related to user phrasing, health literacy, prompt quality, model memory, and the possibility that initial omissions or misleading framing could persist or be reinforced across turns. Therefore, the present findings should be interpreted as characterizing default single-turn responses rather than optimized, interactive, or clinician-supervised LLM dialogues. Second, all prompts and outputs were analyzed in English. English was selected to standardize cross-model comparison, align with the English-language readability formulas used in this study, and facilitate comparison with prior LLM and online patient-education benchmark studies. However, this language choice limits direct generalizability to Chinese-language patient education, particularly because the study was conducted in a Chinese institutional context and many patients most relevant to this setting may not understand English. Response quality, readability, transparency, clinical caution, and red-flag communication may differ substantially across languages because of differences in model training data, language-specific medical terminology, cultural phrasing, health-literacy conventions, and interface behavior. Therefore, the present findings should be interpreted as an English-language benchmark of default public-interface LLM responses rather than as evidence of performance for Chinese-speaking patients. Readability formulas may also overestimate patient-facing difficulty when clinically necessary terminology is present, because they do not distinguish avoidable jargon from unavoidable medical concepts requiring explanation. Therefore, the readability results should be interpreted as formula-based estimates of textual difficulty rather than direct measures of patient comprehension, health-literacy fit, or real-world usability.

Third, formal claim-level factual accuracy, guideline-concordance adjudication, and hallucination-frequency analysis were not performed. This means that the study cannot determine whether individual clinical claims were correct, incomplete, unsupported, contradicted by guidelines, or fabricated. Quality scores, visible transparency-related features, readability metrics, and PCF should therefore be interpreted as communication and exploratory risk-signal measures rather than clinical-validation endpoints.

The question-level minimum clinical-element checklist was investigator-developed and guideline-informed, and it supported contextual interpretation of omissions and safety-critical content; however, it was not externally validated, did not undergo formal Delphi consensus, and should not be interpreted as a validated completeness, accuracy, guideline-concordance, or safety instrument.

Fourth, PCF was exploratory, conservative, threshold-based, and not externally validated. The absence of PCF-positive responses does not exclude clinically relevant omissions, insufficient urgency, incomplete guideline coverage, limited actionability, or subtle misleading framing. Because PCF was threshold-based, responses that were generally cautious but insufficiently specific, insufficiently actionable, or missing noncritical guideline elements could still receive a score of 0. Interpretation should also account for the benchmark size; the 24-question set was not powered to detect rare but clinically serious safety failures.

Fifth, although the 24-prompt set was domain- and source-balanced, it was not exhaustive. Important components of diabetic foot and diabetes education, including glycemic control, medication management, footwear selection, smoking cessation, nutrition, exercise, psychosocial barriers, cost, access to care, and long-term multidisciplinary care coordination, were not evaluated as standalone prompts. Some of these topics appeared indirectly within prevention, recurrence, offloading, and follow-up questions, but the study should not be interpreted as a comprehensive evaluation of all diabetic foot education needs. The findings therefore apply to the selected decision-critical patient-facing scenarios included in the benchmark rather than to the full spectrum of diabetic foot care.

Additionally, although responses were evaluated by experienced orthopedic physicians with surgical expertise and adjudicated by a senior orthopedic physician with surgical expertise, diabetic foot care is multidisciplinary. The findings therefore primarily reflect an orthopedic surgical perspective and may differ from assessments made by endocrinologists, vascular surgeons, infectious disease specialists, podiatrists, wound-care nurses, rehabilitation specialists, diabetes educators, or other members of multidisciplinary diabetic foot teams. Independent ratings from these disciplines were not obtained. In addition, the moderate inter-rater agreement observed for some measures may partly reflect the subjective nature of patient-information scoring and rater-level or specialty-informed differences in interpreting response completeness, actionability, urgency, and clinical emphasis. Because all raters were from an orthopedic surgical background, the present study could not quantify cross-specialty variability. Sixth, the question set was domain-balanced and source-balanced rather than prevalence-weighted; consequently, findings should not be interpreted as estimating the distribution of real-world public diabetic foot information needs.

The complete verbatim model-generated response text is provided in **Supplementary Appendix A**, together with the benchmark questions, model access conditions, scoring rubrics, adjudicated item-level scores, readability outputs, and extended analyses. Nevertheless, independent reproducibility remains partly limited by the dynamic nature of public web-interface LLMs. The responses represent single outputs generated under default public-interface conditions on May 8–9, 2026, and later model or interface updates may yield different responses. Although model-identifying metadata, URLs, tracking parameters, interface elements, timestamps, and response order were removed before blinded scoring, complete masking of model source cannot be guaranteed because some LLMs may have recognizable stylistic patterns, response structures, disclaimer habits, or formatting tendencies. Therefore, residual stylistic unblinding cannot be fully excluded. To mitigate this limitation, responses were processed using a standardized template, anonymized before scoring, independently evaluated by two raters, adjudicated by a senior physician, and summarized at the item-by-model level.

Finally, the study relied on public web interfaces under default conditions. Model outputs may change over time because public-facing LLMs are frequently updated, and platform-level hidden instructions or interface-specific behavior may not be fully controllable. Patient comprehension, behavioral intention, actual care-seeking behavior, and clinical outcomes were not assessed. These dimensions remain essential for determining whether LLM-generated information can improve diabetic foot self-care and timely referral in real-world digital public health settings.

### Future directions

Future studies should expand this benchmark by incorporating repeated generation sampling, single-turn versus multi-turn comparison designs, broader and more granular question sets, prespecified claim extraction, claim-level factual-accuracy review, guideline-concordance assessment, hallucination adjudication, multidisciplinary diabetic foot expert review, patient comprehension evaluation, and validated question-level clinical checklists or guideline-concordance tools. Future studies should directly compare alternative prompt-generation and prompt-optimization strategies, including public-query-only prompts, guideline-only prompts, domain-balanced prompts, source-balanced prompts, plain-language rewriting prompts, red-flag-first prompts, and multi-turn clarification prompts, to determine whether specific prompting strategies improve factual accuracy, readability, transparency, actionability, patient comprehension, and clinical safety. Future work should develop and validate diabetic-foot-specific patient-information checklists through multidisciplinary expert consensus, preferably including endocrinology, vascular surgery, infectious disease, podiatry, wound-care nursing, rehabilitation, diabetes education, and patient representatives. Future studies should combine formula-based readability metrics with medical-jargon-aware assessment, plain-language expert review, patient comprehension testing, actionability assessment, and user-centered evaluation to determine whether patients can understand, recall, and act on diabetic-foot information. Multi-turn studies should prespecify follow-up prompts, including requests for sixth-grade rewriting, source or guideline attribution, clarification of red-flag symptoms, action-oriented summaries, and patient-specific escalation thresholds, while also evaluating whether iterative dialogue improves or worsens factual accuracy, readability, transparency, and safety. Broader question sets should explicitly include additional education topics such as glycemic control, medication management, footwear selection, smoking cessation, nutrition, exercise, access to care, and long-term multidisciplinary follow-up. Multidisciplinary expert panels should include endocrinology, vascular surgery, infectious disease, podiatry, wound-care nursing, rehabilitation, and diabetes education perspectives to strengthen the clinical breadth, validity, and generalizability of future evaluations. Such studies should classify individual claims as accurate, incomplete, unsupported, contradicted, or fabricated against prespecified guideline and evidence standards, with inter-rater reliability reported for these classifications. Prompt-based interventions should be tested, including instructions to respond at a sixth-grade reading level, list urgent warning signs first, specify same-day or emergency care thresholds, and cite current diabetic foot guidelines. Chinese-language and multilingual benchmarks are needed, particularly for populations at high risk of diabetic foot complications and with substantial health literacy barriers. Future studies should directly compare English- and Chinese-language outputs using culturally adapted prompts, Chinese-language readability or comprehension measures, Chinese diabetic foot terminology, and expert assessment by multidisciplinary clinicians familiar with Chinese patient-education contexts. Additional research should examine whether improved LLM-generated patient education influences understanding, care-seeking intention, offloading behavior, recurrence-prevention knowledge, or clinical outcomes. Such work would help define when and how patient-facing LLM systems can be safely and effectively integrated into digital public health pathways.

## Conclusions

In this communication-focused benchmark of five publicly accessible LLMs, default responses to 24 selected patient-facing diabetic foot questions showed metric-specific variation in information quality, visible transparency-related features, and readability. Visible transparency was limited, and no response met all predefined patient-education readability targets. Although no PCF-positive response was identified, this finding does not establish factual accuracy, guideline concordance, absence of hallucinations, or definitive safety. Given these limitations and the high-risk nature of diabetic foot disease, default LLM responses should not be relied upon independently by patients without clinician oversight or formal clinical validation.

## Data Availability

The datasets presented in this study can be found in online repositories. The names of the repository/repositories and accession number(s) can be found in the article/**Supplementary Material**.
