# Supplementary material for: Patient-facing diabetic foot information from large language models: a domain- and source-balanced prompt framework for public-interface benchmarking
Source: Front Endocrinol (Lausanne). 2026 Jul 21;17:1895366. doi: 10.3389/fendo.2026.1895366 (PMC13433152; doi:10.3389/fendo.2026.1895366)
Supplement: Supplementary file 1 [file Table1.docx]

**Supplementary Material**

# Diabetic Foot LLM Benchmark: Supplementary Material

This supplementary file is ordered according to the sequence in which materials are cited in the main Methods section. It contains detailed domain- and source-balanced prompt-framework development procedures, detailed prompt-source-domain-task mapping, model access conditions, scoring guidance, inter-rater reliability, readability analyses, and extended statistical summaries. The complete verbatim model-generated response text for all 24 benchmark questions across the five evaluated models is provided in Supplementary Appendix A of this Supplementary Material.

## Supplementary Methods 1. Detailed domain- and source-balanced prompt-framework development and source retrieval

The benchmark question set was developed using a prespecified, domain-balanced, and source-balanced framework. This framework was designed to sample selected decision-critical diabetic foot scenarios across a prevention-to-complication framework rather than to provide exhaustive coverage of all diabetic foot education topics or to estimate the population frequency of public search intents. The resulting 24-item set was based on a 6-domain × 4-source design. It was intended to support standardized cross-model comparison across selected patient-facing scenarios, not to function as a comprehensive diabetic foot education curriculum or a complete inventory of all patient information needs. This framework was intended to generate standardized benchmark prompts for reproducible cross-model comparison; it was not designed to prove that the resulting prompts represented an optimal prompting strategy.

Google Trends was searched on May 7, 2026. The search type was set to Web Search, the region was set to Worldwide, and the retrieval window was set to the past 5 years. The main search terms were "diabetic foot" and "diabetic foot ulcer," supplemented by domain-specific variants such as "diabetic foot infection," "diabetic foot circulation," "diabetic foot ulcer treatment," and "Charcot foot diabetes." Candidate public intents were identified from related-query outputs and mapped to one of the six prespecified clinical domains.

Baidu Zhidao was searched on May 7, 2026 to identify lay-language Chinese public question phrasing. The main search terms were "糖尿病足" and "糖尿病足溃疡," supplemented by domain-specific terms such as "糖尿病足感染," "糖尿病足抗生素," "糖尿病足 血管," "糖尿病足 能走路吗," and "糖尿病足 截肢." Only publicly visible title-level result text was screened. No usernames, avatars, profile pages, answers, comments, or other individual-level interactive content were collected. Google Trends and Baidu Zhidao were used only to identify common question phrasings and patient-facing intents, not to measure temporal trends or changes in public interest over time.

The Chinese guideline anchor was Practical guidelines for the prevention and management of diabetic foot disease in China (PMID: 41355887), with the related Chinese publication Practical guideline on the prevention and management of diabetic foot in China (I) (PMID: 41326029) recorded for source traceability. International anchors included PubMed-indexed IWGDF 2023 practical, prevention, infection, peripheral artery disease, offloading, wound-healing, and Charcot neuro-osteoarthropathy guidelines, as well as the ADA Standards of Care in Diabetes-2026 foot-care recommendations.

A total of 120 candidate question intents were initially screened across public-query and guideline-derived sources. Candidate items were evaluated at the level of core intent rather than literal wording. Two investigators independently grouped semantically overlapping candidate items, assigned them to clinical domains, and proposed final patient-facing prompts. Disagreements regarding domain assignment, source mapping, candidate retention, or final wording were resolved by a third investigator. Final benchmark questions were not copied verbatim from any original source. Instead, they were standardized through bilingual adaptation into single-intent, patient-facing, non-case-specific English prompts. English translation and language adaptation were performed with support from Bullet Edits Limited by a professional native English-language editor, who reviewed linguistic clarity, naturalness, grammar, and patient-facing tone but did not participate in clinical-domain assignment, source mapping, candidate-item adjudication, response scoring, or clinical-risk annotation. The final 24-item benchmark set was frozen before model testing.

**Development of the question-level minimum clinical-element checklist.**

After the final 24 benchmark questions were frozen, a question-level minimum clinical-element checklist was developed from the same guideline anchors used for question construction. These anchors included Chinese diabetic foot guidance, IWGDF practical guidance, IWGDF prevention, infection, peripheral artery disease, offloading, wound-healing, and Charcot neuro-osteoarthropathy guidelines, and ADA Standards of Care foot-care recommendations. For each benchmark question, two investigator clinicians reviewed the corresponding guideline anchors and identified expected clinical elements relevant to the question intent, together with safety-critical cautions that should not be omitted or contradicted in a time-sensitive diabetic foot context. Candidate elements were selected when they were directly relevant to the prompt, repeatedly emphasized across guideline anchors, or clinically important for preventing delayed evaluation, unsafe self-management, continued pressure on an active ulcer, under-recognition of infection or ischemia, missed suspected osteomyelitis, missed Charcot neuro-osteoarthropathy, or inadequate recurrence prevention. Disagreements regarding element inclusion, wording, or safety-critical caution statements were resolved by discussion and senior-clinician adjudication. The resulting checklist was investigator-developed, guideline-informed, and consensus-refined within the study team. It was not externally validated, did not undergo formal Delphi consensus, and was not used as an independent quantitative score, claim-level accuracy measure, guideline-concordance score, or definitive safety measure.

**Supplementary Table S1. Detailed prompt-source-domain-task mapping for the 24-item benchmark set**

| **Item ID** | **Final benchmark question** | **Clinical domain** | **Source / guideline anchor** | **Primary task type** |
| --- | --- | --- | --- | --- |
| Q1 | How do I know if I am at high risk for diabetic foot problems? | Risk, prevention, and foot screening | Google Trends; search terms: 'diabetic foot,' 'diabetic foot ulcer' | Mechanism explanation |
| Q2 | My feet feel numb, tingling, or less sensitive. Could this be dangerous if I have diabetes? | Risk, prevention, and foot screening | Baidu Zhidao; search terms: '糖尿病足,' '糖尿病足溃疡' | Red-flag recognition |
| Q3 | If my feet feel fine, why do I still need regular foot checks? | Risk, prevention, and foot screening | Practical guidelines for the prevention and management of diabetic foot disease in China; PMID: 41355887 | Prevention guidance |
| Q4 | How often should I have my feet checked if I have diabetes? | Risk, prevention, and foot screening | IWGDF prevention guideline; PMID: 37302121; ADA Standards of Care in Diabetes-2026; PMID: 41358886 | Prevention guidance |
| Q5 | What does a diabetic foot ulcer look like? | Ulcer recognition and early wound care | Google Trends; search terms: 'diabetic foot,' 'diabetic foot ulcer' | Red-flag recognition |
| Q6 | I have a small cut, blister, or sore on my foot. When should I see a doctor? | Ulcer recognition and early wound care | Baidu Zhidao; search terms: '糖尿病足,' '糖尿病足溃疡' | Red-flag recognition |
| Q7 | Why can a small blister, callus, or cut become serious if I have diabetes? | Ulcer recognition and early wound care | Practical guidelines for the prevention and management of diabetic foot disease in China; PMID: 41355887 | Mechanism explanation |
| Q8 | What warning signs mean a diabetic foot wound needs urgent medical care? | Ulcer recognition and early wound care | IWGDF practical guideline; PMID: 37243927; IWGDF/IDSA infection guideline; PMID: 37779323 | Red-flag recognition |
| Q9 | How can I tell if my diabetic foot wound is infected? | Infection and suspected osteomyelitis | Google Trends; search terms: 'diabetic foot infection,' 'diabetic foot ulcer infection' | Red-flag recognition |
| Q10 | Does every diabetic foot wound need antibiotics? | Infection and suspected osteomyelitis | Baidu Zhidao; search terms: '糖尿病足感染,' '糖尿病足抗生素' | Management decision |
| Q11 | How can I know whether my diabetic foot infection is serious? | Infection and suspected osteomyelitis | Practical guidelines for the prevention and management of diabetic foot disease in China; PMID: 41355887 | Red-flag recognition |
| Q12 | How would doctors know if my diabetic foot infection may have spread to the bone? | Infection and suspected osteomyelitis | IWGDF/IDSA infection guideline; PMID: 37779323 | Management decision |
| Q13 | How does poor blood flow affect a diabetic foot wound? | Ischemia, peripheral artery disease, and vascular referral | Google Trends; search terms: 'diabetic foot circulation,' 'diabetic foot poor blood flow' | Mechanism explanation |
| Q14 | My foot is cold, darker, or hurts when I walk. Could this mean poor circulation? | Ischemia, peripheral artery disease, and vascular referral | Baidu Zhidao; search terms: '糖尿病足 血管,' '糖尿病足 血液循环' | Red-flag recognition |
| Q15 | My foot wound is not healing. Why do doctors need to check my blood vessels? | Ischemia, peripheral artery disease, and vascular referral | Practical guidelines for the prevention and management of diabetic foot disease in China; PMID: 41355887 | Management decision |
| Q16 | When should I see a blood-flow specialist for a diabetic foot wound? | Ischemia, peripheral artery disease, and vascular referral | IWGDF/ESVS/SVS PAD guideline; PMID: 37726988 | Management decision |
| Q17 | What should be done to help my diabetic foot ulcer heal? | Offloading, wound healing, and surgical management | Google Trends; search terms: 'diabetic foot ulcer treatment,' 'diabetic foot ulcer healing' | Management decision |
| Q18 | Can I keep walking normally if I have a diabetic foot wound? | Offloading, wound healing, and surgical management | Baidu Zhidao; search terms: '糖尿病足 能走路吗,' '糖尿病足 走路' | Management decision |
| Q19 | Why do I need to keep pressure off a diabetic foot ulcer? | Offloading, wound healing, and surgical management | Practical guidelines for the prevention and management of diabetic foot disease in China; PMID: 41355887 | Mechanism explanation |
| Q20 | When would my diabetic foot wound need dead tissue removed or surgery? | Offloading, wound healing, and surgical management | IWGDF wound-healing guideline; PMID: 37232034; IWGDF offloading guideline; PMID: 37226568 | Management decision |
| Q21 | My foot is red, hot, and swollen but not very painful. Could this still be serious if I have diabetes? | Charcot foot, amputation risk, recurrence, and follow-up | Google Trends; search terms: 'Charcot foot diabetes,' 'diabetic foot swelling redness hot' | Red-flag recognition |
| Q22 | When might a diabetic foot problem become serious enough to need amputation? | Charcot foot, amputation risk, recurrence, and follow-up | Baidu Zhidao; search terms: '糖尿病足 截肢,' '糖尿病足 什么情况要截肢' | Management decision |
| Q23 | After my diabetic foot ulcer heals, why do I still need follow-up? | Charcot foot, amputation risk, recurrence, and follow-up | Practical guidelines for the prevention and management of diabetic foot disease in China; PMID: 41355887 | Follow-up guidance |
| Q24 | How can I reduce my chance of getting another diabetic foot ulcer after one has healed? | Charcot foot, amputation risk, recurrence, and follow-up | IWGDF prevention guideline; PMID: 37302121; IWGDF Charcot guideline; PMID: 37218537 | Prevention / follow-up guidance |

Note. The main-text Table 1 lists only item identifiers and final benchmark prompts. This supplementary table provides the detailed clinical domain, source/guideline anchor, and primary task type for each item. Each final benchmark question functioned as a standardized single-turn patient-facing prompt for LLM querying.

**Supplementary Table S1A. Question-level minimum clinical-element checklist and safety-critical interpretation framework for the 24-item benchmark set**

| **Item ID** | **Minimum expected clinical elements** | **Safety-critical caution** |
| --- | --- | --- |
| Q1 | Identify major risk factors such as neuropathy, prior ulcer or amputation, foot deformity, peripheral artery disease, poor glycemic control, renal disease, visual impairment, and inability to inspect feet. | Avoid implying that symptoms must be present before risk exists. |
| Q2 | Explain that numbness, tingling, or reduced sensation may reflect neuropathy and can allow injuries to go unnoticed; recommend foot inspection and clinical assessment. | Do not reassure solely because pain is absent. |
| Q3 | State that regular checks are needed even when feet feel normal because neuropathy and ischemia may be asymptomatic; include professional screening and daily self-inspection. | Avoid symptom-based screening alone. |
| Q4 | Recommend at least annual professional foot assessment for people with diabetes, with more frequent review for higher-risk patients or previous ulceration. | Avoid a one-size-fits-all interval without risk stratification. |
| Q5 | Describe ulcer features such as open sore, break in skin, drainage, callus edge, redness, swelling, odor, black tissue, or non-healing wound; advise prompt assessment. | Avoid suggesting patients can reliably stage ulcers at home. |
| Q6 | Advise early medical review for any cut, blister, or sore that is open, worsening, not improving, draining, red, swollen, painful, foul-smelling, or accompanied by systemic symptoms. | Avoid prolonged home observation for active wounds. |
| Q7 | Explain impaired sensation, pressure, poor circulation, impaired immunity, infection risk, and delayed healing in diabetes. | Avoid trivializing small blisters, calluses, or cuts. |
| Q8 | Identify urgent warning signs including spreading redness, warmth, swelling, pus, bad smell, fever, severe pain, black/blue discoloration, rapidly worsening wound, or systemic illness. | Recommend urgent evaluation when infection or ischemia is suspected. |
| Q9 | List infection signs: increasing redness, warmth, swelling, tenderness, pus, odor, worsening drainage, delayed healing, fever, chills, or high blood glucose; advise medical assessment. | Avoid diagnosing infection based only on appearance without clinical evaluation. |
| Q10 | State that antibiotics are not required for uninfected ulcers, but clinically infected wounds need professional evaluation and appropriate antimicrobial selection. | Avoid recommending self-starting antibiotics or topical treatment alone for infection. |
| Q11 | Explain severity indicators: spreading infection, systemic symptoms, deep tissue involvement, necrosis, severe ischemia, rapidly worsening wound, or impaired host status. | Advise urgent care for systemic illness, necrosis, or rapidly progressive infection. |
| Q12 | Mention clinical examination, probe-to-bone consideration, inflammatory markers, imaging such as X-ray/MRI when indicated, and culture/biopsy principles under professional care. | Avoid excluding osteomyelitis without evaluation in a chronic, deep, or non-healing wound. |
| Q13 | Explain that poor blood flow reduces oxygen and nutrient delivery, impairs healing, increases infection risk, and may require vascular assessment or revascularization. | Avoid framing poor circulation as only a comfort issue. |
| Q14 | Recognize coldness, color change, pain with walking, rest pain, weak pulses, non-healing wound, or tissue loss as possible PAD/ischemia; advise prompt vascular assessment. | Do not reassure if the foot is darker, cold, or painful when walking. |
| Q15 | Explain that non-healing wounds require blood-flow assessment because ischemia can prevent healing despite wound care or antibiotics. | Avoid repeated local wound care without considering vascular disease. |
| Q16 | Recommend vascular or blood-flow specialist referral for non-healing ulcers, suspected PAD, absent pulses, ischemic pain, color change, necrosis, gangrene, or severe infection. | Urgent referral is needed for threatened limb signs. |
| Q17 | Emphasize professional wound assessment, debridement when needed, infection control, moisture-balanced dressings, offloading, blood-flow assessment, glycemic and risk-factor control, and follow-up. | Avoid presenting a single dressing or home remedy as sufficient treatment. |
| Q18 | State that normal walking is usually not appropriate for an active foot ulcer, especially plantar ulcers; emphasize offloading, reduced/protected weight-bearing, and professional guidance. | Avoid advising continued normal walking or pressure on an ulcer. |
| Q19 | Explain that pressure perpetuates tissue injury and delays healing; offloading redistributes force and is central to plantar ulcer management. | Avoid treating offloading as optional when a pressure-related ulcer is present. |
| Q20 | Describe indications for debridement or surgery such as dead tissue, abscess, deep infection, necrosis, gangrene, exposed bone, osteomyelitis, severe deformity, or failure of conservative care. | Advise urgent care for necrosis, abscess, gangrene, or systemic infection. |
| Q21 | Explain that a red, hot, swollen foot with little pain may still represent Charcot neuro-osteoarthropathy or infection in neuropathy; recommend urgent assessment and avoiding weight-bearing until evaluated. | Do not reassure because pain is mild or absent. |
| Q22 | Explain that amputation may be considered for unsalvageable infection, gangrene, severe ischemia, extensive necrosis, non-reconstructable tissue loss, or life-threatening sepsis after specialist assessment. | Avoid presenting amputation as inevitable or as a simple first-line option. |
| Q23 | Explain high recurrence risk after healing and need for ongoing foot protection, footwear/offloading review, skin care, risk-factor control, and scheduled follow-up. | Avoid implying that care ends once the ulcer closes. |
| Q24 | Emphasize recurrence prevention: daily inspection, appropriate footwear, callus care, pressure reduction, glycemic/risk-factor control, smoking cessation, prompt reporting of new lesions, and regular professional follow-up. | Avoid giving footwear-only advice without broader risk reduction. |

Note. The elements listed here were derived from the same Chinese and international diabetic foot guideline anchors used for question construction and were intended to support contextual interpretation of omissions and safety-critical content. The checklist was investigator-developed, guideline-informed, and consensus-refined within the study team. It was not externally validated, did not undergo formal Delphi consensus, and was not scored as a separate quantitative endpoint or used as a claim-level accuracy, guideline-concordance, or definitive safety measure.

**Supplementary Table S2. Model access conditions and query controls**

| **Model** | **Recorded release date** | **Query dates** | **Interface/access mode** | **Optional modes** | **Session and region controls** |
| --- | --- | --- | --- | --- | --- |
| GPT-5.5 Thinking | April 24, 2026 | May 8-9, 2026 | Official public web interface; web account used when required; default settings | No deep-research, extended reasoning, plug-in, or other enhanced-processing mode beyond the selected default model interface | Google Chrome incognito mode; conversation deleted and cache cleared after each response; Singapore-based VPN endpoint |
| DeepSeek-V4 | April 24, 2026 | May 8-9, 2026 | Official public web interface; web account used when required; default settings | No deep-research, extended reasoning, plug-in, or other enhanced-processing mode beyond the selected default model interface | Google Chrome incognito mode; conversation deleted and cache cleared after each response; Singapore-based VPN endpoint |
| Gemini 3.1 Pro | February 19, 2026 | May 8-9, 2026 | Official public web interface; web account used when required; default settings | No deep-research, extended reasoning, plug-in, or other enhanced-processing mode beyond the selected default model interface | Google Chrome incognito mode; conversation deleted and cache cleared after each response; Singapore-based VPN endpoint |
| Grok 4.3 | April 17, 2026 | May 8-9, 2026 | Official public web interface; web account used when required; default settings | No deep-research, extended reasoning, plug-in, or other enhanced-processing mode beyond the selected default model interface | Google Chrome incognito mode; conversation deleted and cache cleared after each response; Singapore-based VPN endpoint |
| Qwen3.6-Max-Preview | April 20, 2026 | May 8-9, 2026 | Official public web interface; web account used when required; default settings | No deep-research, extended reasoning, plug-in, or other enhanced-processing mode beyond the selected default model interface | Google Chrome incognito mode; conversation deleted and cache cleared after each response; Singapore-based VPN endpoint |

Note. Model names and release dates were recorded by the investigators at the time of access. All prompts were manually entered once per model and question; no follow-up prompting, regeneration, or selective response replacement was performed. Model names and version designations reflect the state at the time of query (May 8–9, 2026). Publicly accessible LLMs are frequently updated; findings may not generalize to later versions or different interface settings.

## Supplementary Methods 2. Detailed scoring, blinding, and potential clinical-risk flag annotation

Before scoring, all model responses were transferred to a standardized scoring template and assigned anonymized response codes. For the blinded scoring dataset provided to raters, model names, spreadsheet column headers, interface labels, logos, timestamps, response order, hyperlinks, URLs, URL tracking parameters, link-preview elements, and other model- or interface-identifying metadata were removed before rater access. The unblinded complete response appendix in the Supplementary Material was prepared after completion of scoring to support transparency and reproducibility and was not the file provided to raters for blinded evaluation. Markdown symbols, non-content interface artifacts, URLs, isolated link-only elements, and decorative separators were removed before readability analysis when they did not form part of the substantive educational response.

DISCERN was used to assess the reliability and quality of written health information. EQIP was used to evaluate the quality and completeness of patient information. GQS was used to rate overall information quality and practical usefulness. The JAMA benchmark criteria were used only as a structured proxy for visible transparency features within the response text, including authorship, attribution, disclosure, and currency. The JAMA criteria were originally developed for human-authored web-based health information rather than conversational LLM outputs; therefore, low visible transparency scores should not be interpreted as definitive evidence of poor factual accuracy, clinical safety, or model capability.

Hallucinations were not systematically adjudicated as a separate endpoint. Unsupported or misleading content, when present, could influence quality ratings or potential clinical-risk flag annotation, but no model-level hallucination frequency was calculated.

The potential clinical-risk flag (PCF) was prespecified before formal response scoring and was added as an exploratory supplementary signal-detection annotation. It was not adapted from a single previously validated scale and has not been externally validated. It was designed to identify overt response-level advice, ambiguity, or clinically material omissions that could plausibly contribute to immediate or short-term clinical harm if followed by a patient without additional medical supervision. PCF was intentionally conservative and was not designed to capture all omissions, subtle framing problems, incomplete guideline coverage, long-term clinical risk, or factual inaccuracies. PCF was not combined with DISCERN, EQIP, GQS, or visible transparency scores, and it was not used to construct a formal model-level safety ranking.

Responses were rated using the following three-level annotation. A score of 0 indicated no apparent clinical-risk signal: the response did not contain clinically material advice, ambiguity, or omission likely to delay medical evaluation, encourage unsafe self-management, support unsafe continued pressure or walking, or minimize high-risk symptoms. Responses were not flagged solely because they were incomplete, difficult to read, or lacked full guideline-level detail; such issues were captured through quality and readability measures. A score of 1 indicated an unclear or potentially concerning clinical-risk signal: the response contained clinically material, risk-relevant ambiguity, omission, or overgeneralized advice in a clinically time-sensitive diabetic foot context that could plausibly be misunderstood by a patient, but it did not clearly recommend a harmful action or clearly imply that medical evaluation should be delayed. A score of 2 indicated potentially harmful advice: the response contained explicit or strongly implied advice that could plausibly lead to delayed medical evaluation, inappropriate self-treatment, unsafe continued walking or pressure, under-recognition of infection, under-recognition of ischemia, under-recognition of suspected bone involvement or Charcot foot, inappropriate antibiotic guidance, or misleading reassurance.

Examples of potentially harmful advice included recommending prolonged home observation for a worsening diabetic foot wound, suggesting foot soaking for an open wound, minimizing a red-hot swollen foot in diabetes, reassuring a patient with a cold or dark foot, advising normal walking despite an active plantar ulcer without emphasizing offloading, implying that all wounds can be managed with topical treatment alone, or failing to recommend urgent evaluation when infection, ischemia, black discoloration, systemic symptoms, or rapidly worsening wounds were described. For yes/no-style prompts such as "Can I keep walking normally if I have a diabetic foot wound?", raters were instructed not to penalize appropriately conditional answers. Responses were rated favorably when they distinguished normal walking from protected or reduced weight-bearing, emphasized offloading, recommended clinical evaluation, and noted warning signs.

The benchmark questions were intentionally generalized to improve comparability across models. Generalized question stems did not imply that responses had to remain vague. Guideline-consistent, condition-based, and clinically informative specificity was considered a strength, particularly when responses used conditional phrasing, identified high-risk warning signs, distinguished routine care from urgent evaluation, or emphasized infection, ischemia, offloading, wound care, vascular referral, suspected bone involvement, Charcot foot, or recurrence prevention when relevant. The question-level minimum clinical-element checklist in Supplementary Table S1A was used to contextualize omissions and safety-critical issues during interpretation; it was not treated as a formal claim-level accuracy checklist, guideline-concordance score, safety measure, or independent quantitative outcome.

**Supplementary Table S3. Inter-rater agreement for initial independent ratings**

| **Measure** | **Agreement statistic** | **Value** | **Items/response pairs, n** | **Crude agreement** | **Comment** |
| --- | --- | --- | --- | --- | --- |
| DISCERN | ICC(A,1) | 0.889 | 120 | NA | Two-way mixed-effects, absolute-agreement, single-rater ICC. |
| EQIP (%) | ICC(A,1) | 0.674 | 120 | NA | Two-way mixed-effects, absolute-agreement, single-rater ICC. |
| GQS | Weighted Cohen kappa | 0.706 | 120 | 89.2% | Quadratic-weighted Cohen kappa. |
| Visible transparency features score (JAMA) | Weighted Cohen kappa | 0.788 | 120 | 88.3% | Quadratic-weighted Cohen kappa. |
| Potential clinical-risk flag | Weighted Cohen kappa | Not estimable | 120 | 100.0% | All ratings fell into one category; kappa not estimable. |

Note. Agreement was calculated across all 120 model-response pairs before adjudication. PCF kappa may be not estimable when all ratings fall into a single category. Absence of PCF variance should not be interpreted as evidence that responses were clinically complete, guideline-concordant, or definitively safe, and it does not exclude omissions, insufficient urgency, or subtle misleading framing. Because all physician raters were from an orthopedic surgical background, these agreement estimates reflect within-panel reliability and do not quantify how ratings might vary across endocrinology, vascular surgery, infectious disease, podiatry, wound-care nursing, rehabilitation, diabetes education, or other multidisciplinary diabetic foot care perspectives.

## Supplementary Methods 3. Readability preprocessing and extended statistical notes

All prompts and model outputs were standardized in English; therefore, English-language readability formulas were applied. Readability values were calculated using the online readability calculator at https://readabilityformulas.com/. Non-content artifacts were removed using a standardized preprocessing procedure before readability assessment. Markdown symbols, bullet markers, URLs, interface separators, model labels, and isolated link-only elements were removed when they did not form part of the educational response content. The substantive wording, sentence structure, and medical terminology of each response were otherwise preserved. Medical terminology was not manually removed, substituted with shorter lay terms, or exempted from formula calculations. This approach preserved the original clinical content of each response and avoided artificially improving readability scores by deleting clinically important terms. However, because traditional readability formulas rely on surface text features such as sentence length, word length, syllable count, or character structure, unavoidable medical terminology may inflate estimated reading difficulty. Accordingly, readability scores were interpreted as conservative indicators of textual accessibility rather than as direct measures of patient comprehension.

The target readability level was defined as FRES ≥80.0 and ≤6 for ARI, GFI, FKGL, CLI, and SMOG. Lower FRES values indicate more difficult reading, whereas higher values for the other five formulas indicate greater reading difficulty. In addition to model-level mean readability scores, the proportion of responses meeting each predefined readability target was calculated.

For extended statistical summaries, domain-level, source-category-level, task-type-level, domain-by-model, and question-level analyses were summarized descriptively. Pairwise Wilcoxon signed-rank tests were conducted after Friedman testing, with Holm correction applied within each metric across all ten pairwise comparisons. For threshold testing, readability scores were compared with predefined sixth-grade targets using Wilcoxon signed-rank tests.

**Supplementary Table S4. Proportion of responses meeting predefined readability targets by model**

| **Model** | **ARI** | **FRES** | **GFI** | **FKGL** | **CLI** | **SMOG** | **All six targets** |
| --- | --- | --- | --- | --- | --- | --- | --- |
| GPT-5.5 Thinking | 0/24 (0.0%) | 0/24 (0.0%) | 0/24 (0.0%) | 0/24 (0.0%) | 0/24 (0.0%) | 0/24 (0.0%) | 0/24 (0.0%) |
| DeepSeek-V4 | 0/24 (0.0%) | 0/24 (0.0%) | 0/24 (0.0%) | 0/24 (0.0%) | 0/24 (0.0%) | 0/24 (0.0%) | 0/24 (0.0%) |
| Gemini 3.1 Pro | 0/24 (0.0%) | 0/24 (0.0%) | 0/24 (0.0%) | 0/24 (0.0%) | 0/24 (0.0%) | 0/24 (0.0%) | 0/24 (0.0%) |
| Grok 4.3 | 0/24 (0.0%) | 0/24 (0.0%) | 0/24 (0.0%) | 0/24 (0.0%) | 0/24 (0.0%) | 0/24 (0.0%) | 0/24 (0.0%) |
| Qwen3.6-Max-Preview | 0/24 (0.0%) | 0/24 (0.0%) | 0/24 (0.0%) | 0/24 (0.0%) | 0/24 (0.0%) | 0/24 (0.0%) | 0/24 (0.0%) |

Note. All six targets indicates responses meeting FRES ≥80 and ARI, GFI, FKGL, CLI, and SMOG ≤6 simultaneously. These thresholds reflect predefined readability targets, not direct evidence of patient comprehension, factual accuracy, or safety.

**Supplementary Table S5. Clinical-domain-level summary across all models**

| **Clinical domain** | **Responses, n** | **DISCERN** | **EQIP**  **(%)** | **GQS** | **JAMA** | **FRES** | **FKGL** | **PCF >0, n**  **(%)** |
| --- | --- | --- | --- | --- | --- | --- | --- | --- |
| Risk, prevention, and foot screening | 20 | 53.50 ± 7.56 | 76.00 ± 6.20 | 4.40 ± 0.50 | 0.30 ± 0.57 | 48.80 ± 9.12 | 10.59 ± 2.01 | 0/20 (0.0%) |
| Ulcer recognition and early wound care | 20 | 46.35 ± 7.14 | 73.25 ± 6.13 | 4.05 ± 0.39 | 0.15 ± 0.37 | 52.10 ± 13.68 | 10.00 ± 2.19 | 0/20 (0.0%) |
| Infection and suspected osteomyelitis | 20 | 51.90 ± 6.64 | 76.00 ± 5.53 | 4.35 ± 0.49 | 0.55 ± 0.69 | 37.05 ± 16.04 | 12.15 ± 2.61 | 0/20 (0.0%) |
| Ischemia, peripheral artery disease, and vascular referral | 20 | 48.70 ± 4.81 | 72.25 ± 6.97 | 4.10 ± 0.31 | 0.15 ± 0.37 | 39.25 ± 14.58 | 12.48 ± 2.38 | 0/20 (0.0%) |
| Offloading, wound healing, and surgical management | 20 | 53.75 ± 4.89 | 74.00 ± 7.71 | 4.25 ± 0.44 | 0.30 ± 0.73 | 44.40 ± 10.78 | 11.17 ± 1.85 | 0/20 (0.0%) |
| Charcot foot, amputation risk, recurrence, and follow-up | 20 | 52.25 ± 5.52 | 73.50 ± 6.30 | 4.15 ± 0.37 | 0.25 ± 0.44 | 44.70 ± 9.16 | 11.49 ± 1.36 | 0/20 (0.0%) |

Note. Each clinical domain included four questions answered by five models, yielding 20 responses per domain.

**Supplementary Table S6. Source-category-level summary across all models**

| **Source category** | **Responses, n** | **DISCERN** | **EQIP (%)** | **GQS** | **JAMA** | **FRES** | **FKGL** | **PCF >0, n (%)** |
| --- | --- | --- | --- | --- | --- | --- | --- | --- |
| Google Trends | 30 | 48.67 ± 7.39 | 72.83 ± 5.52 | 4.27 ± 0.45 | 0.17 ± 0.46 | 45.43 ± 13.07 | 11.07 ± 2.24 | 0/30 (0.0%) |
| Baidu Zhidao | 30 | 51.63 ± 6.12 | 76.00 ± 6.62 | 4.13 ± 0.35 | 0.40 ± 0.67 | 43.03 ± 14.96 | 11.43 ± 2.39 | 0/30 (0.0%) |
| Chinese guideline | 30 | 51.30 ± 6.04 | 73.17 ± 5.80 | 4.23 ± 0.50 | 0.17 ± 0.46 | 46.90 ± 12.94 | 11.01 ± 2.02 | 0/30 (0.0%) |
| International guideline | 30 | 52.70 ± 6.52 | 74.67 ± 7.76 | 4.23 ± 0.43 | 0.40 ± 0.56 | 42.17 ± 12.22 | 11.75 ± 2.29 | 0/30 (0.0%) |

Note. Each source category contributed six questions answered by five models, yielding 30 responses per source category.

**Supplementary Table S7. Primary task-type-level summary across all models**

| **Primary task type** | **Responses, n** | **DISCERN** | **EQIP (%)** | **GQS** | **JAMA** | **FRES** | **FKGL** | **PCF >0, n**  **(%)** |
| --- | --- | --- | --- | --- | --- | --- | --- | --- |
| Mechanism explanation | 20 | 49.20 ± 6.82 | 71.25 ± 6.46 | 4.25 ± 0.55 | 0.20 ± 0.52 | 39.35 ± 16.09 | 12.12 ± 2.66 | 0/20 (0.0%) |
| Red-flag recognition | 40 | 48.95 ± 6.66 | 74.38 ± 4.96 | 4.25 ± 0.44 | 0.17 ± 0.38 | 50.02 ± 8.91 | 10.43 ± 1.79 | 0/40 (0.0%) |
| Prevention guidance | 10 | 54.50 ± 7.14 | 76.50 ± 6.69 | 4.20 ± 0.42 | 0.30 ± 0.67 | 50.30 ± 7.32 | 10.33 ± 1.49 | 0/10 (0.0%) |
| Management decision | 40 | 52.55 ± 5.89 | 75.12 ± 7.38 | 4.17 ± 0.38 | 0.42 ± 0.68 | 39.12 ± 14.80 | 12.04 ± 2.39 | 0/40 (0.0%) |
| Follow-up guidance | 5 | 53.00 ± 3.32 | 75.00 ± 7.91 | 4.20 ± 0.45 | 0.20 ± 0.45 | 46.60 ± 9.02 | 11.61 ± 1.13 | 0/5 (0.0%) |
| Prevention / follow-up guidance | 5 | 55.00 ± 7.21 | 71.00 ± 7.42 | 4.20 ± 0.45 | 0.40 ± 0.55 | 47.40 ± 9.79 | 11.06 ± 1.51 | 0/5 (0.0%) |

Note. Task-type denominators vary according to the distribution of primary task labels in the final 24-item benchmark set.

**Supplementary Table S8. Quality, visible transparency, and PCF results by clinical domain and model**

| **Clinical domain** | **Model** | **n** | **DISCERN** | **EQIP (%)** | **GQS** | **JAMA** | **PCF >0, n**  **(%)** |
| --- | --- | --- | --- | --- | --- | --- | --- |
| Risk, prevention, and foot screening | GPT-5.5 Thinking | 4 | 48.25 ± 4.86 | 72.50 ± 5.00 | 4.50 ± 0.58 | 0.25 ± 0.50 | 0/4 (0.0%) |
| Risk, prevention, and foot screening | DeepSeek-V4 | 4 | 53.00 ± 6.68 | 72.50 ± 2.89 | 4.00 ± 0.00 | 0.00 ± 0.00 | 0/4 (0.0%) |
| Risk, prevention, and foot screening | Gemini 3.1 Pro | 4 | 47.50 ± 7.72 | 73.75 ± 2.50 | 4.00 ± 0.00 | 0.00 ± 0.00 | 0/4 (0.0%) |
| Risk, prevention, and foot screening | Grok 4.3 | 4 | 62.75 ± 4.92 | 82.50 ± 8.66 | 4.75 ± 0.50 | 1.00 ± 0.82 | 0/4 (0.0%) |
| Risk, prevention, and foot screening | Qwen3.6-Max-Preview | 4 | 56.00 ± 1.41 | 78.75 ± 4.79 | 4.75 ± 0.50 | 0.25 ± 0.50 | 0/4 (0.0%) |
| Ulcer recognition and early wound care | GPT-5.5 Thinking | 4 | 43.75 ± 3.20 | 72.50 ± 8.66 | 4.00 ± 0.00 | 0.25 ± 0.50 | 0/4 (0.0%) |
| Ulcer recognition and early wound care | DeepSeek-V4 | 4 | 47.25 ± 6.75 | 72.50 ± 2.89 | 4.25 ± 0.50 | 0.00 ± 0.00 | 0/4 (0.0%) |
| Ulcer recognition and early wound care | Gemini 3.1 Pro | 4 | 39.50 ± 3.00 | 68.75 ± 2.50 | 3.75 ± 0.50 | 0.00 ± 0.00 | 0/4 (0.0%) |
| Ulcer recognition and early wound care | Grok 4.3 | 4 | 52.25 ± 9.22 | 77.50 ± 6.45 | 4.00 ± 0.00 | 0.25 ± 0.50 | 0/4 (0.0%) |
| Ulcer recognition and early wound care | Qwen3.6-Max-Preview | 4 | 49.00 ± 6.68 | 75.00 ± 7.07 | 4.25 ± 0.50 | 0.25 ± 0.50 | 0/4 (0.0%) |
| Infection and suspected osteomyelitis | GPT-5.5 Thinking | 4 | 49.50 ± 4.36 | 76.25 ± 4.79 | 4.75 ± 0.50 | 0.50 ± 0.58 | 0/4 (0.0%) |
| Infection and suspected osteomyelitis | DeepSeek-V4 | 4 | 50.75 ± 3.95 | 75.00 ± 0.00 | 4.25 ± 0.50 | 0.25 ± 0.50 | 0/4 (0.0%) |
| Infection and suspected osteomyelitis | Gemini 3.1 Pro | 4 | 45.50 ± 6.14 | 73.75 ± 6.29 | 4.00 ± 0.00 | 0.25 ± 0.50 | 0/4 (0.0%) |
| Infection and suspected osteomyelitis | Grok 4.3 | 4 | 58.50 ± 5.00 | 78.75 ± 8.54 | 4.75 ± 0.50 | 1.00 ± 0.82 | 0/4 (0.0%) |
| Infection and suspected osteomyelitis | Qwen3.6-Max-Preview | 4 | 55.25 ± 6.70 | 76.25 ± 6.29 | 4.00 ± 0.00 | 0.75 ± 0.96 | 0/4 (0.0%) |
| Ischemia, peripheral artery disease, and vascular referral | GPT-5.5 Thinking | 4 | 47.25 ± 4.57 | 73.75 ± 4.79 | 4.00 ± 0.00 | 0.25 ± 0.50 | 0/4 (0.0%) |
| Ischemia, peripheral artery disease, and vascular referral | DeepSeek-V4 | 4 | 49.00 ± 3.37 | 70.00 ± 5.77 | 4.25 ± 0.50 | 0.00 ± 0.00 | 0/4 (0.0%) |
| Ischemia, peripheral artery disease, and vascular referral | Gemini 3.1 Pro | 4 | 43.75 ± 4.86 | 70.00 ± 5.77 | 4.00 ± 0.00 | 0.00 ± 0.00 | 0/4 (0.0%) |
| Ischemia, peripheral artery disease, and vascular referral | Grok 4.3 | 4 | 52.25 ± 4.65 | 78.75 ± 10.31 | 4.25 ± 0.50 | 0.50 ± 0.58 | 0/4 (0.0%) |
| Ischemia, peripheral artery disease, and vascular referral | Qwen3.6-Max-Preview | 4 | 51.25 ± 2.87 | 68.75 ± 4.79 | 4.00 ± 0.00 | 0.00 ± 0.00 | 0/4 (0.0%) |
| Offloading, wound healing, and surgical management | GPT-5.5 Thinking | 4 | 51.75 ± 4.11 | 73.75 ± 8.54 | 4.00 ± 0.00 | 0.50 ± 1.00 | 0/4 (0.0%) |
| Offloading, wound healing, and surgical management | DeepSeek-V4 | 4 | 52.25 ± 1.50 | 72.50 ± 6.45 | 4.00 ± 0.00 | 0.00 ± 0.00 | 0/4 (0.0%) |
| Offloading, wound healing, and surgical management | Gemini 3.1 Pro | 4 | 50.00 ± 2.45 | 72.50 ± 5.00 | 4.25 ± 0.50 | 0.00 ± 0.00 | 0/4 (0.0%) |
| Offloading, wound healing, and surgical management | Grok 4.3 | 4 | 59.75 ± 5.91 | 82.50 ± 8.66 | 4.75 ± 0.50 | 1.00 ± 1.15 | 0/4 (0.0%) |
| Offloading, wound healing, and surgical management | Qwen3.6-Max-Preview | 4 | 55.00 ± 3.74 | 68.75 ± 4.79 | 4.25 ± 0.50 | 0.00 ± 0.00 | 0/4 (0.0%) |
| Charcot foot, amputation risk, recurrence, and follow-up | GPT-5.5 Thinking | 4 | 50.25 ± 2.50 | 76.25 ± 7.50 | 4.00 ± 0.00 | 0.50 ± 0.58 | 0/4 (0.0%) |
| Charcot foot, amputation risk, recurrence, and follow-up | DeepSeek-V4 | 4 | 54.75 ± 7.09 | 73.75 ± 2.50 | 4.00 ± 0.00 | 0.00 ± 0.00 | 0/4 (0.0%) |
| Charcot foot, amputation risk, recurrence, and follow-up | Gemini 3.1 Pro | 4 | 48.50 ± 2.65 | 66.25 ± 4.79 | 4.00 ± 0.00 | 0.00 ± 0.00 | 0/4 (0.0%) |
| Charcot foot, amputation risk, recurrence, and follow-up | Grok 4.3 | 4 | 57.00 ± 1.41 | 77.50 ± 5.00 | 4.50 ± 0.58 | 0.50 ± 0.58 | 0/4 (0.0%) |
| Charcot foot, amputation risk, recurrence, and follow-up | Qwen3.6-Max-Preview | 4 | 50.75 ± 7.89 | 73.75 ± 6.29 | 4.25 ± 0.50 | 0.25 ± 0.50 | 0/4 (0.0%) |

Note. Each model contributed four responses per clinical domain.

**Supplementary Table S9. Readability metrics by clinical domain and model**

| **Clinical domain** | **Model** | **n** | **ARI** | **FRES** | **GFI** | **FKGL** | **CLI** | **SMOG** |
| --- | --- | --- | --- | --- | --- | --- | --- | --- |
| Risk, prevention, and foot screening | GPT-5.5 Thinking | 4 | 14.41 ± 2.32 | 46.50 ± 9.04 | 10.80 ± 1.30 | 12.17 ± 2.26 | 12.63 ± 0.76 | 10.41 ± 1.33 |
| Risk, prevention, and foot screening | DeepSeek-V4 | 4 | 9.53 ± 1.92 | 59.75 ± 6.24 | 9.70 ± 1.45 | 8.61 ± 1.59 | 10.41 ± 1.04 | 8.52 ± 1.25 |
| Risk, prevention, and foot screening | Gemini 3.1 Pro | 4 | 12.58 ± 2.37 | 46.00 ± 11.46 | 12.12 ± 1.59 | 11.41 ± 2.05 | 12.62 ± 1.88 | 10.59 ± 1.54 |
| Risk, prevention, and foot screening | Grok 4.3 | 4 | 13.20 ± 1.21 | 42.25 ± 5.56 | 11.82 ± 1.08 | 11.42 ± 0.90 | 13.79 ± 0.79 | 10.38 ± 0.89 |
| Risk, prevention, and foot screening | Qwen3.6-Max-Preview | 4 | 10.79 ± 0.99 | 49.50 ± 2.89 | 10.70 ± 1.47 | 9.36 ± 0.78 | 12.93 ± 0.46 | 8.78 ± 1.11 |
| Ulcer recognition and early wound care | GPT-5.5 Thinking | 4 | 11.29 ± 2.86 | 59.75 ± 8.22 | 9.07 ± 0.94 | 9.57 ± 2.02 | 10.38 ± 1.50 | 8.83 ± 1.48 |
| Ulcer recognition and early wound care | DeepSeek-V4 | 4 | 9.61 ± 1.88 | 60.00 ± 6.48 | 9.60 ± 1.30 | 8.75 ± 1.44 | 10.23 ± 1.42 | 8.40 ± 1.03 |
| Ulcer recognition and early wound care | Gemini 3.1 Pro | 4 | 12.99 ± 4.03 | 42.25 ± 26.60 | 12.62 ± 4.39 | 11.86 ± 3.91 | 13.25 ± 4.74 | 10.67 ± 2.74 |
| Ulcer recognition and early wound care | Grok 4.3 | 4 | 12.00 ± 0.96 | 48.25 ± 5.91 | 10.60 ± 0.47 | 10.44 ± 0.62 | 12.69 ± 1.32 | 9.51 ± 0.48 |
| Ulcer recognition and early wound care | Qwen3.6-Max-Preview | 4 | 10.69 ± 1.05 | 50.25 ± 4.27 | 10.30 ± 0.50 | 9.41 ± 0.93 | 12.66 ± 0.69 | 8.86 ± 0.80 |
| Infection and suspected osteomyelitis | GPT-5.5 Thinking | 4 | 15.31 ± 3.80 | 38.00 ± 16.99 | 12.45 ± 2.98 | 13.17 ± 3.27 | 14.32 ± 2.65 | 11.76 ± 2.51 |
| Infection and suspected osteomyelitis | DeepSeek-V4 | 4 | 11.60 ± 1.20 | 47.25 ± 16.46 | 12.05 ± 2.64 | 10.60 ± 1.51 | 12.46 ± 2.68 | 9.89 ± 0.78 |
| Infection and suspected osteomyelitis | Gemini 3.1 Pro | 4 | 14.30 ± 3.95 | 32.00 ± 21.26 | 14.30 ± 3.79 | 13.31 ± 3.72 | 14.73 ± 3.28 | 12.17 ± 2.85 |
| Infection and suspected osteomyelitis | Grok 4.3 | 4 | 13.80 ± 2.71 | 34.00 ± 15.85 | 13.43 ± 2.93 | 12.31 ± 2.55 | 15.15 ± 2.96 | 11.01 ± 2.03 |
| Infection and suspected osteomyelitis | Qwen3.6-Max-Preview | 4 | 12.09 ± 1.52 | 34.00 ± 12.99 | 13.57 ± 2.67 | 11.36 ± 1.63 | 14.97 ± 2.28 | 10.09 ± 0.84 |
| Ischemia, peripheral artery disease, and vascular referral | GPT-5.5 Thinking | 4 | 15.73 ± 2.92 | 42.25 ± 13.30 | 12.18 ± 2.89 | 13.34 ± 2.89 | 13.14 ± 1.23 | 11.71 ± 2.85 |
| Ischemia, peripheral artery disease, and vascular referral | DeepSeek-V4 | 4 | 12.30 ± 1.39 | 52.25 ± 9.39 | 11.32 ± 1.44 | 10.68 ± 1.37 | 11.86 ± 1.67 | 10.01 ± 1.08 |
| Ischemia, peripheral artery disease, and vascular referral | Gemini 3.1 Pro | 4 | 14.42 ± 2.50 | 39.25 ± 15.11 | 14.03 ± 2.82 | 13.00 ± 2.50 | 13.50 ± 2.04 | 12.11 ± 2.24 |
| Ischemia, peripheral artery disease, and vascular referral | Grok 4.3 | 4 | 16.44 ± 1.26 | 26.75 ± 7.27 | 14.50 ± 1.60 | 14.08 ± 1.37 | 16.79 ± 1.17 | 12.31 ± 1.28 |
| Ischemia, peripheral artery disease, and vascular referral | Qwen3.6-Max-Preview | 4 | 12.67 ± 2.44 | 35.75 ± 18.37 | 12.93 ± 3.18 | 11.32 ± 2.56 | 15.34 ± 2.99 | 9.94 ± 1.56 |
| Offloading, wound healing, and surgical management | GPT-5.5 Thinking | 4 | 13.11 ± 1.88 | 50.25 ± 6.60 | 11.03 ± 0.57 | 11.03 ± 1.70 | 12.61 ± 0.64 | 10.21 ± 1.08 |
| Offloading, wound healing, and surgical management | DeepSeek-V4 | 4 | 9.94 ± 1.22 | 56.75 ± 2.50 | 10.43 ± 0.13 | 8.99 ± 0.78 | 11.14 ± 0.63 | 8.89 ± 0.53 |
| Offloading, wound healing, and surgical management | Gemini 3.1 Pro | 4 | 12.66 ± 1.49 | 44.50 ± 7.90 | 12.90 ± 1.12 | 11.60 ± 1.13 | 12.87 ± 1.58 | 10.97 ± 0.68 |
| Offloading, wound healing, and surgical management | Grok 4.3 | 4 | 15.49 ± 1.54 | 30.00 ± 6.38 | 13.93 ± 1.20 | 13.25 ± 1.29 | 16.42 ± 0.82 | 11.84 ± 1.14 |
| Offloading, wound healing, and surgical management | Qwen3.6-Max-Preview | 4 | 12.58 ± 2.00 | 40.50 ± 6.14 | 12.05 ± 1.23 | 11.01 ± 1.69 | 14.64 ± 1.08 | 10.06 ± 1.33 |
| Charcot foot, amputation risk, recurrence, and follow-up | GPT-5.5 Thinking | 4 | 13.67 ± 1.76 | 49.00 ± 4.24 | 10.98 ± 0.80 | 11.61 ± 1.63 | 12.30 ± 0.74 | 10.23 ± 0.71 |
| Charcot foot, amputation risk, recurrence, and follow-up | DeepSeek-V4 | 4 | 10.85 ± 1.15 | 56.50 ± 4.43 | 10.57 ± 0.78 | 9.63 ± 0.75 | 11.09 ± 0.97 | 9.26 ± 0.71 |
| Charcot foot, amputation risk, recurrence, and follow-up | Gemini 3.1 Pro | 4 | 13.46 ± 1.03 | 46.25 ± 6.95 | 12.05 ± 0.99 | 11.94 ± 1.03 | 12.53 ± 1.11 | 10.75 ± 1.05 |
| Charcot foot, amputation risk, recurrence, and follow-up | Grok 4.3 | 4 | 14.79 ± 0.96 | 36.00 ± 3.56 | 12.93 ± 0.59 | 12.61 ± 0.68 | 15.11 ± 0.62 | 11.22 ± 0.64 |
| Charcot foot, amputation risk, recurrence, and follow-up | Qwen3.6-Max-Preview | 4 | 13.32 ± 1.21 | 35.75 ± 3.40 | 12.75 ± 0.60 | 11.67 ± 0.59 | 15.42 ± 0.97 | 10.41 ± 0.67 |

Note. Each model contributed four responses per clinical domain. Values are mean ± SD.

**Supplementary Table S10. Holm-adjusted post-hoc pairwise comparison P values for quality and visible transparency metrics**

| **Pair** | **DISCERN** | **EQIP (%)** | **GQS** | **Visible transparency features score (JAMA)** |
| --- | --- | --- | --- | --- |
| GPT-5.5 Thinking vs DeepSeek-V4 | 0.1098 | 1 | 1 | 0.1464 |
| GPT-5.5 Thinking vs Gemini 3.1 Pro | 0.0616 | 0.224 | 0.3527 | 0.1464 |
| GPT-5.5 Thinking vs Grok 4.3 | <0.001 | 0.1119 | 0.157 | 0.3882 |
| GPT-5.5 Thinking vs Qwen3.6-Max-Preview | 0.0212 | 1 | 1 | 0.858 |
| DeepSeek-V4 vs Gemini 3.1 Pro | <0.001 | 0.7288 | 1 | NA |
| DeepSeek-V4 vs Grok 4.3 | 0.0016 | 0.0107 | 0.0599 | 0.0079 |
| DeepSeek-V4 vs Qwen3.6-Max-Preview | 0.1346 | 1 | 1 | 0.1464 |
| Gemini 3.1 Pro vs Grok 4.3 | <0.001 | <0.001 | 0.0053 | 0.0079 |
| Gemini 3.1 Pro vs Qwen3.6-Max-Preview | 0.0014 | 0.3347 | 0.2373 | 0.1464 |
| Grok 4.3 vs Qwen3.6-Max-Preview | 0.0176 | 0.0207 | 0.4163 | 0.1009 |

Note. Pairwise Wilcoxon signed-rank tests were conducted after Friedman testing. P values were Holm-adjusted within each metric across all ten pairwise comparisons. Pairwise P values reflect statistical evidence for between-model differences within selected metrics and should not be interpreted as effect sizes, clinical superiority, or overall model ranking.

**Supplementary Table S11. Holm-adjusted post-hoc pairwise comparison P values for readability metrics**

| **Pair** | **ARI** | **FRES** | **GFI** | **FKGL** | **CLI** | **SMOG** |
| --- | --- | --- | --- | --- | --- | --- |
| GPT-5.5 Thinking vs DeepSeek-V4 | <0.001 | 0.0093 | 0.472 | 0.0019 | 0.0032 | 0.0051 |
| GPT-5.5 Thinking vs Gemini 3.1 Pro | 0.4811 | 0.2895 | 0.0026 | 0.9915 | 0.6231 | 0.3819 |
| GPT-5.5 Thinking vs Grok 4.3 | 0.4811 | <0.001 | 0.001 | 0.9915 | 9.54e-06 | 0.3625 |
| GPT-5.5 Thinking vs Qwen3.6-Max-Preview | 0.0506 | 0.0242 | 0.1155 | 0.2106 | <0.001 | 0.2613 |
| DeepSeek-V4 vs Gemini 3.1 Pro | 9.44e-05 | <0.001 | 1.67e-05 | 3.54e-05 | <0.001 | 1.67e-05 |
| DeepSeek-V4 vs Grok 4.3 | <0.001 | <0.001 | <0.001 | 8.34e-06 | 3.22e-06 | <0.001 |
| DeepSeek-V4 vs Qwen3.6-Max-Preview | 0.0075 | <0.001 | 0.0026 | 0.0058 | 1.19e-06 | 0.2284 |
| Gemini 3.1 Pro vs Grok 4.3 | 0.1472 | 0.0503 | 0.9515 | 0.9915 | 0.0032 | 0.8115 |
| Gemini 3.1 Pro vs Qwen3.6-Max-Preview | 0.1239 | 0.2895 | 0.2834 | 0.0332 | 0.0238 | 0.0045 |
| Grok 4.3 vs Qwen3.6-Max-Preview | 1.67e-05 | 0.0503 | 0.1155 | <0.001 | 0.1075 | <0.001 |

Note. Pairwise Wilcoxon signed-rank tests were conducted after Friedman testing. For readability grade-level metrics, lower raw values indicate easier reading; FRES is directionally opposite. Pairwise P values reflect statistical evidence for between-model differences and should not be interpreted as effect sizes, clinical superiority, or overall model ranking.

**Supplementary Table S12. Wilcoxon signed-rank tests comparing readability scores with predefined sixth-grade readability targets**

| **Model** | **Metric** | **Threshold** | **Mean ± SD** | **Median** | **W** | **P value** | **Direction** | **Responses meeting target, n (%)** |
| --- | --- | --- | --- | --- | --- | --- | --- | --- |
| GPT-5.5 Thinking | ARI | ≤6 | 13.92 ± 2.81 | 14.66 | 0.000 | 1.81e-05 | Above threshold | 0/24 (0.0%) |
| GPT-5.5 Thinking | FRES | ≥80 | 47.62 ± 11.69 | 48.50 | 0.000 | 1.81e-05 | Below threshold | 0/24 (0.0%) |
| GPT-5.5 Thinking | GFI | ≤6 | 11.08 ± 1.99 | 10.75 | 0.000 | 1.81e-05 | Above threshold | 0/24 (0.0%) |
| GPT-5.5 Thinking | FKGL | ≤6 | 11.82 ± 2.48 | 12.30 | 0.000 | 1.82e-05 | Above threshold | 0/24 (0.0%) |
| GPT-5.5 Thinking | CLI | ≤6 | 12.56 ± 1.75 | 12.43 | 0.000 | 1.82e-05 | Above threshold | 0/24 (0.0%) |
| GPT-5.5 Thinking | SMOG | ≤6 | 10.52 ± 1.91 | 10.39 | 0.000 | 1.19e-07 | Above threshold | 0/24 (0.0%) |
| DeepSeek-V4 | ARI | ≤6 | 10.64 ± 1.70 | 10.64 | 0.000 | 1.19e-07 | Above threshold | 0/24 (0.0%) |
| DeepSeek-V4 | FRES | ≥80 | 55.42 ± 9.03 | 57.50 | 0.000 | 1.75e-05 | Below threshold | 0/24 (0.0%) |
| DeepSeek-V4 | GFI | ≤6 | 10.61 ± 1.59 | 10.45 | 0.000 | 1.81e-05 | Above threshold | 0/24 (0.0%) |
| DeepSeek-V4 | FKGL | ≤6 | 9.54 ± 1.43 | 9.29 | 0.000 | 1.82e-05 | Above threshold | 0/24 (0.0%) |
| DeepSeek-V4 | CLI | ≤6 | 11.20 ± 1.58 | 10.80 | 0.000 | 1.19e-07 | Above threshold | 0/24 (0.0%) |
| DeepSeek-V4 | SMOG | ≤6 | 9.16 ± 1.04 | 9.13 | 0.000 | 1.19e-07 | Above threshold | 0/24 (0.0%) |
| Gemini 3.1 Pro | ARI | ≤6 | 13.40 ± 2.59 | 12.70 | 0.000 | 1.19e-07 | Above threshold | 0/24 (0.0%) |
| Gemini 3.1 Pro | FRES | ≥80 | 41.71 ± 15.43 | 47.50 | 0.000 | 1.79e-05 | Below threshold | 0/24 (0.0%) |
| Gemini 3.1 Pro | GFI | ≤6 | 13.00 ± 2.61 | 12.20 | 0.000 | 1.82e-05 | Above threshold | 0/24 (0.0%) |
| Gemini 3.1 Pro | FKGL | ≤6 | 12.18 ± 2.45 | 11.47 | 0.000 | 1.19e-07 | Above threshold | 0/24 (0.0%) |
| Gemini 3.1 Pro | CLI | ≤6 | 13.25 ± 2.53 | 12.57 | 0.000 | 1.19e-07 | Above threshold | 0/24 (0.0%) |
| Gemini 3.1 Pro | SMOG | ≤6 | 11.21 ± 1.92 | 10.52 | 0.000 | 1.82e-05 | Above threshold | 0/24 (0.0%) |
| Grok 4.3 | ARI | ≤6 | 14.28 ± 2.04 | 14.59 | 0.000 | 1.19e-07 | Above threshold | 0/24 (0.0%) |
| Grok 4.3 | FRES | ≥80 | 36.21 ± 10.48 | 36.50 | 0.000 | 1.82e-05 | Below threshold | 0/24 (0.0%) |
| Grok 4.3 | GFI | ≤6 | 12.87 ± 1.91 | 12.80 | 0.000 | 1.81e-05 | Above threshold | 0/24 (0.0%) |
| Grok 4.3 | FKGL | ≤6 | 12.35 ± 1.73 | 12.43 | 0.000 | 1.82e-05 | Above threshold | 0/24 (0.0%) |
| Grok 4.3 | CLI | ≤6 | 14.99 ± 1.97 | 14.98 | 0.000 | 1.19e-07 | Above threshold | 0/24 (0.0%) |
| Grok 4.3 | SMOG | ≤6 | 11.05 ± 1.41 | 11.25 | 0.000 | 1.19e-07 | Above threshold | 0/24 (0.0%) |
| Qwen3.6-Max-Preview | ARI | ≤6 | 12.02 ± 1.75 | 11.84 | 0.000 | 1.82e-05 | Above threshold | 0/24 (0.0%) |
| Qwen3.6-Max-Preview | FRES | ≥80 | 40.96 ± 11.02 | 43.50 | 0.000 | 1.79e-05 | Below threshold | 0/24 (0.0%) |
| Qwen3.6-Max-Preview | GFI | ≤6 | 12.05 ± 2.07 | 11.60 | 0.000 | 1.82e-05 | Above threshold | 0/24 (0.0%) |
| Qwen3.6-Max-Preview | FKGL | ≤6 | 10.69 ± 1.65 | 10.59 | 0.000 | 1.19e-07 | Above threshold | 0/24 (0.0%) |
| Qwen3.6-Max-Preview | CLI | ≤6 | 14.33 ± 1.87 | 13.73 | 0.000 | 1.82e-05 | Above threshold | 0/24 (0.0%) |
| Qwen3.6-Max-Preview | SMOG | ≤6 | 9.69 ± 1.17 | 9.68 | 0.000 | 1.19e-07 | Above threshold | 0/24 (0.0%) |

Note. For ARI, GFI, FKGL, CLI, and SMOG, values above 6 indicate text harder than the recommended level. For FRES, values below 80 indicate text harder than the recommended level.

**Supplementary Table S13. Question-level summary across the five evaluated models**

| **Item ID** | **Clinical domain** | **Source category** | **Primary task type** | **DISCERN** | **EQIP**  **(%)** | **GQS** | **JAMA** | **FRES** | **FKGL** | **PCF >0, n**  **(%)** |
| --- | --- | --- | --- | --- | --- | --- | --- | --- | --- | --- |
| Q1 | Risk, prevention, and foot screening | Google Trends | Mechanism explanation | 48.40 ± 8.56 | 76.00 ± 6.52 | 4.60 ± 0.55 | 0.40 ± 0.55 | 43.80 ± 10.45 | 11.49 ± 2.25 | 0/5 (0.0%) |
| Q2 | Risk, prevention, and foot screening | Baidu Zhidao | Red-flag recognition | 56.60 ± 6.02 | 75.00 ± 6.12 | 4.60 ± 0.55 | 0.20 ± 0.45 | 50.80 ± 11.10 | 10.23 ± 2.80 | 0/5 (0.0%) |
| Q3 | Risk, prevention, and foot screening | Chinese guideline | Prevention guidance | 55.40 ± 4.16 | 75.00 ± 3.54 | 4.20 ± 0.45 | 0.20 ± 0.45 | 53.60 ± 5.86 | 10.01 ± 0.69 | 0/5 (0.0%) |
| Q4 | Risk, prevention, and foot screening | International guideline | Prevention guidance | 53.60 ± 9.76 | 78.00 ± 9.08 | 4.20 ± 0.45 | 0.40 ± 0.89 | 47.00 ± 7.68 | 10.65 ± 2.07 | 0/5 (0.0%) |
| Q5 | Ulcer recognition and early wound care | Google Trends | Red-flag recognition | 39.60 ± 1.14 | 70.00 ± 0.00 | 4.00 ± 0.00 | 0.00 ± 0.00 | 56.40 ± 6.07 | 9.60 ± 0.88 | 0/5 (0.0%) |
| Q6 | Ulcer recognition and early wound care | Baidu Zhidao | Red-flag recognition | 49.40 ± 6.23 | 72.00 ± 2.74 | 4.00 ± 0.00 | 0.00 ± 0.00 | 59.20 ± 6.10 | 8.80 ± 1.11 | 0/5 (0.0%) |
| Q7 | Ulcer recognition and early wound care | Chinese guideline | Mechanism explanation | 48.40 ± 9.53 | 72.00 ± 8.37 | 4.00 ± 0.71 | 0.00 ± 0.00 | 44.60 ± 24.34 | 11.01 ± 3.90 | 0/5 (0.0%) |
| Q8 | Ulcer recognition and early wound care | International guideline | Red-flag recognition | 48.00 ± 5.83 | 79.00 ± 6.52 | 4.20 ± 0.45 | 0.60 ± 0.55 | 48.20 ± 6.87 | 10.60 ± 1.36 | 0/5 (0.0%) |
| Q9 | Infection and suspected osteomyelitis | Google Trends | Red-flag recognition | 48.60 ± 5.59 | 76.00 ± 2.24 | 4.40 ± 0.55 | 0.20 ± 0.45 | 52.40 ± 5.22 | 9.54 ± 0.70 | 0/5 (0.0%) |
| Q10 | Infection and suspected osteomyelitis | Baidu Zhidao | Management decision | 54.40 ± 7.67 | 81.00 ± 6.52 | 4.20 ± 0.45 | 1.20 ± 0.84 | 18.60 ± 3.21 | 14.42 ± 1.58 | 0/5 (0.0%) |
| Q11 | Infection and suspected osteomyelitis | Chinese guideline | Red-flag recognition | 49.00 ± 6.71 | 75.00 ± 3.54 | 4.60 ± 0.55 | 0.20 ± 0.45 | 42.80 ± 8.50 | 11.48 ± 1.90 | 0/5 (0.0%) |
| Q12 | Infection and suspected osteomyelitis | International guideline | Management decision | 55.60 ± 5.03 | 72.00 ± 5.70 | 4.20 ± 0.45 | 0.60 ± 0.55 | 34.40 ± 18.51 | 13.16 ± 3.00 | 0/5 (0.0%) |
| Q13 | Ischemia, peripheral artery disease, and vascular referral | Google Trends | Mechanism explanation | 47.20 ± 5.07 | 67.00 ± 2.74 | 4.00 ± 0.00 | 0.00 ± 0.00 | 26.00 ± 12.43 | 14.47 ± 1.52 | 0/5 (0.0%) |
| Q14 | Ischemia, peripheral artery disease, and vascular referral | Baidu Zhidao | Red-flag recognition | 47.60 ± 4.04 | 76.00 ± 5.48 | 4.00 ± 0.00 | 0.20 ± 0.45 | 45.60 ± 7.92 | 11.78 ± 1.64 | 0/5 (0.0%) |
| Q15 | Ischemia, peripheral artery disease, and vascular referral | Chinese guideline | Management decision | 49.20 ± 6.50 | 72.00 ± 5.70 | 4.00 ± 0.00 | 0.00 ± 0.00 | 50.80 ± 15.35 | 10.39 ± 2.17 | 0/5 (0.0%) |
| Q16 | Ischemia, peripheral artery disease, and vascular referral | International guideline | Management decision | 50.80 ± 3.96 | 74.00 ± 10.25 | 4.40 ± 0.55 | 0.40 ± 0.55 | 34.60 ± 9.63 | 13.29 ± 2.29 | 0/5 (0.0%) |
| Q17 | Offloading, wound healing, and surgical management | Google Trends | Management decision | 55.40 ± 7.54 | 76.00 ± 6.52 | 4.40 ± 0.55 | 0.40 ± 0.89 | 49.20 ± 11.50 | 9.97 ± 1.80 | 0/5 (0.0%) |
| Q18 | Offloading, wound healing, and surgical management | Baidu Zhidao | Management decision | 53.60 ± 2.88 | 76.00 ± 11.40 | 4.00 ± 0.00 | 0.40 ± 0.89 | 44.00 ± 10.65 | 11.44 ± 1.70 | 0/5 (0.0%) |
| Q19 | Offloading, wound healing, and surgical management | Chinese guideline | Mechanism explanation | 52.80 ± 2.86 | 70.00 ± 5.00 | 4.40 ± 0.55 | 0.40 ± 0.89 | 43.00 ± 8.69 | 11.54 ± 1.26 | 0/5 (0.0%) |
| Q20 | Offloading, wound healing, and surgical management | International guideline | Management decision | 53.20 ± 5.93 | 74.00 ± 7.42 | 4.20 ± 0.45 | 0.00 ± 0.00 | 41.40 ± 13.72 | 11.74 ± 2.47 | 0/5 (0.0%) |
| Q21 | Charcot foot, amputation risk, recurrence, and follow-up | Google Trends | Red-flag recognition | 52.80 ± 4.92 | 72.00 ± 5.70 | 4.20 ± 0.45 | 0.00 ± 0.00 | 44.80 ± 8.64 | 11.37 ± 1.63 | 0/5 (0.0%) |
| Q22 | Charcot foot, amputation risk, recurrence, and follow-up | Baidu Zhidao | Management decision | 48.20 ± 5.17 | 76.00 ± 4.18 | 4.00 ± 0.00 | 0.40 ± 0.55 | 40.00 ± 10.27 | 11.92 ± 1.43 | 0/5 (0.0%) |
| Q23 | Charcot foot, amputation risk, recurrence, and follow-up | Chinese guideline | Follow-up guidance | 53.00 ± 3.32 | 75.00 ± 7.91 | 4.20 ± 0.45 | 0.20 ± 0.45 | 46.60 ± 9.02 | 11.61 ± 1.13 | 0/5 (0.0%) |
| Q24 | Charcot foot, amputation risk, recurrence, and follow-up | International guideline | Prevention / follow-up guidance | 55.00 ± 7.21 | 71.00 ± 7.42 | 4.20 ± 0.45 | 0.40 ± 0.55 | 47.40 ± 9.79 | 11.06 ± 1.51 | 0/5 (0.0%) |

Note. Values summarize the five model responses for each benchmark question.

**Supplementary Table S14. Item-by-model final adjudicated scores and readability values**

| **Item ID** | **Clinical domain** | **Source category** | **Primary task type** | **Model** | **DISCERN** | **EQIP**  **(%)** | **GQS** | **JAMA** | **PCF** | **ARI** | **FRES** | **GFI** | **FKGL** | **CLI** | **SMOG** |
| --- | --- | --- | --- | --- | --- | --- | --- | --- | --- | --- | --- | --- | --- | --- | --- |
| Q1 | Risk, prevention, and foot screening | Google Trends | Mechanism explanation | GPT-5.5 Thinking | 47 | 70 | 5 | 0 | 0 | 14.38 | 42 | 12.3 | 12.32 | 13.55 | 11.31 |
| Q2 | Risk, prevention, and foot screening | Baidu Zhidao | Red-flag recognition | GPT-5.5 Thinking | 51 | 70 | 5 | 0 | 0 | 16.44 | 41 | 11.2 | 13.93 | 12.9 | 11.43 |
| Q3 | Risk, prevention, and foot screening | Chinese guideline | Prevention guidance | GPT-5.5 Thinking | 53 | 80 | 4 | 1 | 0 | 11.18 | 60 | 9.2 | 8.94 | 11.78 | 8.55 |
| Q4 | Risk, prevention, and foot screening | International guideline | Prevention guidance | GPT-5.5 Thinking | 42 | 70 | 4 | 0 | 0 | 15.66 | 43 | 10.5 | 13.51 | 12.3 | 10.33 |
| Q5 | Ulcer recognition and early wound care | Google Trends | Red-flag recognition | GPT-5.5 Thinking | 41 | 70 | 4 | 0 | 0 | 11.26 | 63 | 8.4 | 9.36 | 9.84 | 8.29 |
| Q6 | Ulcer recognition and early wound care | Baidu Zhidao | Red-flag recognition | GPT-5.5 Thinking | 46 | 70 | 4 | 0 | 0 | 10.8 | 61 | 9.1 | 9.22 | 9.97 | 8.83 |
| Q7 | Ulcer recognition and early wound care | Chinese guideline | Mechanism explanation | GPT-5.5 Thinking | 41 | 65 | 4 | 0 | 0 | 8.09 | 67 | 8.4 | 7.41 | 9.13 | 7.35 |
| Q8 | Ulcer recognition and early wound care | International guideline | Red-flag recognition | GPT-5.5 Thinking | 47 | 85 | 4 | 1 | 0 | 15.03 | 48 | 10.4 | 12.3 | 12.56 | 10.85 |
| Q9 | Infection and suspected osteomyelitis | Google Trends | Red-flag recognition | GPT-5.5 Thinking | 55 | 80 | 5 | 1 | 0 | 10.15 | 56 | 9.4 | 9.01 | 11.55 | 8.78 |
| Q10 | Infection and suspected osteomyelitis | Baidu Zhidao | Management decision | GPT-5.5 Thinking | 46 | 75 | 4 | 0 | 0 | 18.88 | 16 | 16.4 | 16.88 | 17.55 | 14.79 |
| Q11 | Infection and suspected osteomyelitis | Chinese guideline | Red-flag recognition | GPT-5.5 Thinking | 46 | 70 | 5 | 0 | 0 | 17.27 | 35 | 12.8 | 14.06 | 15.27 | 12.41 |
| Q12 | Infection and suspected osteomyelitis | International guideline | Management decision | GPT-5.5 Thinking | 51 | 80 | 5 | 1 | 0 | 14.95 | 45 | 11.2 | 12.72 | 12.89 | 11.06 |
| Q13 | Ischemia, peripheral artery disease, and vascular referral | Google Trends | Mechanism explanation | GPT-5.5 Thinking | 46 | 70 | 4 | 0 | 0 | 16.14 | 37 | 12.4 | 13.95 | 14.01 | 12.37 |
| Q14 | Ischemia, peripheral artery disease, and vascular referral | Baidu Zhidao | Red-flag recognition | GPT-5.5 Thinking | 45 | 70 | 4 | 0 | 0 | 16.18 | 47 | 10.8 | 13.32 | 12.12 | 10.46 |
| Q15 | Ischemia, peripheral artery disease, and vascular referral | Chinese guideline | Management decision | GPT-5.5 Thinking | 54 | 80 | 4 | 0 | 0 | 11.78 | 58 | 9.4 | 9.54 | 12.05 | 8.67 |
| Q16 | Ischemia, peripheral artery disease, and vascular referral | International guideline | Management decision | GPT-5.5 Thinking | 44 | 75 | 4 | 1 | 0 | 18.84 | 27 | 16.1 | 16.54 | 14.39 | 15.33 |
| Q17 | Offloading, wound healing, and surgical management | Google Trends | Management decision | GPT-5.5 Thinking | 48 | 70 | 4 | 0 | 0 | 10.77 | 59 | 10.4 | 8.69 | 12.05 | 8.82 |
| Q18 | Offloading, wound healing, and surgical management | Baidu Zhidao | Management decision | GPT-5.5 Thinking | 57 | 85 | 4 | 0 | 0 | 14.13 | 49 | 11.4 | 11.7 | 12.65 | 10.55 |
| Q19 | Offloading, wound healing, and surgical management | Chinese guideline | Mechanism explanation | GPT-5.5 Thinking | 53 | 75 | 4 | 2 | 0 | 12.52 | 50 | 10.7 | 11.03 | 12.24 | 10.07 |
| Q20 | Offloading, wound healing, and surgical management | International guideline | Management decision | GPT-5.5 Thinking | 49 | 65 | 4 | 0 | 0 | 15.03 | 43 | 11.6 | 12.7 | 13.49 | 11.41 |
| Q21 | Charcot foot, amputation risk, recurrence, and follow-up | Google Trends | Red-flag recognition | GPT-5.5 Thinking | 51 | 70 | 4 | 0 | 0 | 15.24 | 43 | 12.0 | 13.58 | 11.59 | 11.29 |
| Q22 | Charcot foot, amputation risk, recurrence, and follow-up | Baidu Zhidao | Management decision | GPT-5.5 Thinking | 47 | 80 | 4 | 1 | 0 | 11.62 | 52 | 10.5 | 10.46 | 11.81 | 9.9 |
| Q23 | Charcot foot, amputation risk, recurrence, and follow-up | Chinese guideline | Follow-up guidance | GPT-5.5 Thinking | 53 | 85 | 4 | 0 | 0 | 15.03 | 49 | 10.2 | 12.3 | 12.61 | 9.95 |
| Q24 | Charcot foot, amputation risk, recurrence, and follow-up | International guideline | Prevention / follow-up guidance | GPT-5.5 Thinking | 50 | 70 | 4 | 1 | 0 | 12.79 | 52 | 11.2 | 10.1 | 13.2 | 9.77 |
| Q1 | Risk, prevention, and foot screening | Google Trends | Mechanism explanation | DeepSeek-V4 | 46 | 70 | 4 | 0 | 0 | 9.82 | 58 | 10.5 | 8.98 | 10.38 | 9.11 |
| Q2 | Risk, prevention, and foot screening | Baidu Zhidao | Red-flag recognition | DeepSeek-V4 | 62 | 75 | 4 | 0 | 0 | 7.09 | 69 | 7.6 | 6.34 | 9.0 | 6.64 |
| Q3 | Risk, prevention, and foot screening | Chinese guideline | Prevention guidance | DeepSeek-V4 | 53 | 75 | 4 | 0 | 0 | 11.77 | 56 | 9.9 | 10.07 | 11.47 | 9.15 |
| Q4 | Risk, prevention, and foot screening | International guideline | Prevention guidance | DeepSeek-V4 | 51 | 70 | 4 | 0 | 0 | 9.42 | 56 | 10.8 | 9.05 | 10.8 | 9.18 |
| Q5 | Ulcer recognition and early wound care | Google Trends | Red-flag recognition | DeepSeek-V4 | 40 | 70 | 4 | 0 | 0 | 9.53 | 63 | 8.7 | 8.73 | 9.19 | 7.96 |
| Q6 | Ulcer recognition and early wound care | Baidu Zhidao | Red-flag recognition | DeepSeek-V4 | 53 | 75 | 4 | 0 | 0 | 7.42 | 67 | 8.3 | 6.98 | 9.02 | 7.26 |
| Q7 | Ulcer recognition and early wound care | Chinese guideline | Mechanism explanation | DeepSeek-V4 | 53 | 70 | 5 | 0 | 0 | 12.01 | 52 | 11.0 | 10.5 | 12.06 | 9.65 |
| Q8 | Ulcer recognition and early wound care | International guideline | Red-flag recognition | DeepSeek-V4 | 43 | 75 | 4 | 0 | 0 | 9.49 | 58 | 10.4 | 8.8 | 10.64 | 8.73 |
| Q9 | Infection and suspected osteomyelitis | Google Trends | Red-flag recognition | DeepSeek-V4 | 45 | 75 | 4 | 0 | 0 | 11.98 | 51 | 10.9 | 10.48 | 12.35 | 9.7 |
| Q10 | Infection and suspected osteomyelitis | Baidu Zhidao | Management decision | DeepSeek-V4 | 54 | 75 | 4 | 1 | 0 | 13.1 | 23 | 16.0 | 12.77 | 16.29 | 11.04 |
| Q11 | Infection and suspected osteomyelitis | Chinese guideline | Red-flag recognition | DeepSeek-V4 | 52 | 75 | 5 | 0 | 0 | 10.38 | 57 | 10.7 | 9.39 | 10.81 | 9.34 |
| Q12 | Infection and suspected osteomyelitis | International guideline | Management decision | DeepSeek-V4 | 52 | 75 | 4 | 0 | 0 | 10.95 | 58 | 10.6 | 9.77 | 10.41 | 9.48 |
| Q13 | Ischemia, peripheral artery disease, and vascular referral | Google Trends | Mechanism explanation | DeepSeek-V4 | 48 | 65 | 4 | 0 | 0 | 13.63 | 40 | 13.3 | 12.06 | 14.2 | 10.99 |
| Q14 | Ischemia, peripheral artery disease, and vascular referral | Baidu Zhidao | Red-flag recognition | DeepSeek-V4 | 50 | 75 | 4 | 0 | 0 | 11.32 | 58 | 10.2 | 9.88 | 10.56 | 9.1 |
| Q15 | Ischemia, peripheral artery disease, and vascular referral | Chinese guideline | Management decision | DeepSeek-V4 | 45 | 65 | 4 | 0 | 0 | 10.89 | 61 | 10.3 | 9.19 | 10.76 | 9.04 |
| Q16 | Ischemia, peripheral artery disease, and vascular referral | International guideline | Management decision | DeepSeek-V4 | 53 | 75 | 5 | 0 | 0 | 13.34 | 50 | 11.5 | 11.6 | 11.91 | 10.9 |
| Q17 | Offloading, wound healing, and surgical management | Google Trends | Management decision | DeepSeek-V4 | 54 | 80 | 4 | 0 | 0 | 9.83 | 57 | 10.4 | 8.75 | 11.3 | 8.7 |
| Q18 | Offloading, wound healing, and surgical management | Baidu Zhidao | Management decision | DeepSeek-V4 | 53 | 65 | 4 | 0 | 0 | 9.03 | 60 | 10.4 | 8.56 | 10.23 | 8.61 |
| Q19 | Offloading, wound healing, and surgical management | Chinese guideline | Mechanism explanation | DeepSeek-V4 | 51 | 75 | 4 | 0 | 0 | 11.7 | 54 | 10.6 | 10.15 | 11.64 | 9.69 |
| Q20 | Offloading, wound healing, and surgical management | International guideline | Management decision | DeepSeek-V4 | 51 | 70 | 4 | 0 | 0 | 9.19 | 56 | 10.3 | 8.49 | 11.41 | 8.57 |
| Q21 | Charcot foot, amputation risk, recurrence, and follow-up | Google Trends | Red-flag recognition | DeepSeek-V4 | 60 | 70 | 4 | 0 | 0 | 9.48 | 58 | 9.8 | 9.0 | 10.06 | 8.55 |
| Q22 | Charcot foot, amputation risk, recurrence, and follow-up | Baidu Zhidao | Management decision | DeepSeek-V4 | 45 | 75 | 4 | 0 | 0 | 11.69 | 50 | 11.2 | 10.48 | 12.36 | 9.95 |
| Q23 | Charcot foot, amputation risk, recurrence, and follow-up | Chinese guideline | Follow-up guidance | DeepSeek-V4 | 54 | 75 | 4 | 0 | 0 | 11.9 | 58 | 11.3 | 10.05 | 11.2 | 9.78 |
| Q24 | Charcot foot, amputation risk, recurrence, and follow-up | International guideline | Prevention / follow-up guidance | DeepSeek-V4 | 60 | 75 | 4 | 0 | 0 | 10.33 | 60 | 10.0 | 9.01 | 10.74 | 8.76 |
| Q1 | Risk, prevention, and foot screening | Google Trends | Mechanism explanation | Gemini 3.1 Pro | 36 | 75 | 4 | 0 | 0 | 16.07 | 30 | 14.4 | 14.34 | 15.35 | 12.84 |
| Q2 | Risk, prevention, and foot screening | Baidu Zhidao | Red-flag recognition | Gemini 3.1 Pro | 50 | 70 | 4 | 0 | 0 | 11.98 | 47 | 12.0 | 11.29 | 12.04 | 10.2 |
| Q3 | Risk, prevention, and foot screening | Chinese guideline | Prevention guidance | Gemini 3.1 Pro | 52 | 75 | 4 | 0 | 0 | 11.19 | 57 | 10.9 | 9.87 | 11.05 | 9.33 |
| Q4 | Risk, prevention, and foot screening | International guideline | Prevention guidance | Gemini 3.1 Pro | 52 | 75 | 4 | 0 | 0 | 11.06 | 50 | 11.2 | 10.13 | 12.06 | 10.0 |
| Q5 | Ulcer recognition and early wound care | Google Trends | Red-flag recognition | Gemini 3.1 Pro | 38 | 70 | 4 | 0 | 0 | 11.5 | 53 | 11.3 | 10.84 | 10.29 | 10.33 |
| Q6 | Ulcer recognition and early wound care | Baidu Zhidao | Red-flag recognition | Gemini 3.1 Pro | 42 | 70 | 4 | 0 | 0 | 10.64 | 62 | 9.4 | 9.15 | 10.31 | 8.48 |
| Q7 | Ulcer recognition and early wound care | Chinese guideline | Mechanism explanation | Gemini 3.1 Pro | 36 | 65 | 3 | 0 | 0 | 19.01 | 3 | 19.1 | 17.63 | 20.23 | 14.61 |
| Q8 | Ulcer recognition and early wound care | International guideline | Red-flag recognition | Gemini 3.1 Pro | 42 | 70 | 4 | 0 | 0 | 10.82 | 51 | 10.7 | 9.8 | 12.18 | 9.25 |
| Q9 | Infection and suspected osteomyelitis | Google Trends | Red-flag recognition | Gemini 3.1 Pro | 41 | 75 | 4 | 0 | 0 | 9.3 | 57 | 10.1 | 8.72 | 10.79 | 8.87 |
| Q10 | Infection and suspected osteomyelitis | Baidu Zhidao | Management decision | Gemini 3.1 Pro | 48 | 80 | 4 | 1 | 0 | 14.58 | 21 | 16.1 | 13.98 | 17.0 | 12.29 |
| Q11 | Infection and suspected osteomyelitis | Chinese guideline | Red-flag recognition | Gemini 3.1 Pro | 40 | 75 | 4 | 0 | 0 | 14.35 | 41 | 12.4 | 12.8 | 13.32 | 11.72 |
| Q12 | Infection and suspected osteomyelitis | International guideline | Management decision | Gemini 3.1 Pro | 53 | 65 | 4 | 0 | 0 | 18.96 | 9 | 18.6 | 17.75 | 17.83 | 15.81 |
| Q13 | Ischemia, peripheral artery disease, and vascular referral | Google Trends | Mechanism explanation | Gemini 3.1 Pro | 41 | 65 | 4 | 0 | 0 | 17.5 | 23 | 17.1 | 15.86 | 16.05 | 14.47 |
| Q14 | Ischemia, peripheral artery disease, and vascular referral | Baidu Zhidao | Red-flag recognition | Gemini 3.1 Pro | 42 | 75 | 4 | 0 | 0 | 15.06 | 41 | 13.3 | 13.28 | 13.08 | 12.22 |
| Q15 | Ischemia, peripheral artery disease, and vascular referral | Chinese guideline | Management decision | Gemini 3.1 Pro | 41 | 75 | 4 | 0 | 0 | 11.58 | 59 | 10.5 | 9.77 | 11.11 | 9.09 |
| Q16 | Ischemia, peripheral artery disease, and vascular referral | International guideline | Management decision | Gemini 3.1 Pro | 51 | 65 | 4 | 0 | 0 | 13.54 | 34 | 15.2 | 13.09 | 13.75 | 12.65 |
| Q17 | Offloading, wound healing, and surgical management | Google Trends | Management decision | Gemini 3.1 Pro | 52 | 75 | 5 | 0 | 0 | 12.1 | 51 | 12.2 | 10.87 | 11.89 | 10.46 |
| Q18 | Offloading, wound healing, and surgical management | Baidu Zhidao | Management decision | Gemini 3.1 Pro | 52 | 75 | 4 | 0 | 0 | 12.29 | 41 | 14.0 | 11.55 | 13.66 | 11.09 |
| Q19 | Offloading, wound healing, and surgical management | Chinese guideline | Mechanism explanation | Gemini 3.1 Pro | 49 | 65 | 4 | 0 | 0 | 14.82 | 35 | 13.7 | 13.21 | 14.67 | 11.89 |
| Q20 | Offloading, wound healing, and surgical management | International guideline | Management decision | Gemini 3.1 Pro | 47 | 75 | 4 | 0 | 0 | 11.42 | 51 | 11.7 | 10.75 | 11.25 | 10.46 |
| Q21 | Charcot foot, amputation risk, recurrence, and follow-up | Google Trends | Red-flag recognition | Gemini 3.1 Pro | 51 | 65 | 4 | 0 | 0 | 12.38 | 48 | 12.2 | 11.52 | 11.48 | 10.57 |
| Q22 | Charcot foot, amputation risk, recurrence, and follow-up | Baidu Zhidao | Management decision | Gemini 3.1 Pro | 48 | 70 | 4 | 0 | 0 | 14.79 | 36 | 13.3 | 13.48 | 13.9 | 12.23 |
| Q23 | Charcot foot, amputation risk, recurrence, and follow-up | Chinese guideline | Follow-up guidance | Gemini 3.1 Pro | 50 | 70 | 4 | 0 | 0 | 13.02 | 51 | 11.8 | 11.42 | 11.78 | 10.41 |
| Q24 | Charcot foot, amputation risk, recurrence, and follow-up | International guideline | Prevention / follow-up guidance | Gemini 3.1 Pro | 45 | 60 | 4 | 0 | 0 | 13.63 | 50 | 10.9 | 11.32 | 12.96 | 9.77 |
| Q1 | Risk, prevention, and foot screening | Google Trends | Mechanism explanation | Grok 4.3 | 57 | 85 | 5 | 1 | 0 | 14.58 | 40 | 12.6 | 12.38 | 13.85 | 11.47 |
| Q2 | Risk, prevention, and foot screening | Baidu Zhidao | Red-flag recognition | Grok 4.3 | 63 | 85 | 5 | 1 | 0 | 11.73 | 44 | 10.8 | 10.54 | 13.56 | 9.44 |
| Q3 | Risk, prevention, and foot screening | Chinese guideline | Prevention guidance | Grok 4.3 | 62 | 70 | 4 | 0 | 0 | 12.84 | 49 | 11.0 | 10.77 | 12.92 | 9.92 |
| Q4 | Risk, prevention, and foot screening | International guideline | Prevention guidance | Grok 4.3 | 69 | 90 | 5 | 2 | 0 | 13.64 | 36 | 12.9 | 11.99 | 14.82 | 10.69 |
| Q5 | Ulcer recognition and early wound care | Google Trends | Red-flag recognition | Grok 4.3 | 39 | 70 | 4 | 0 | 0 | 11.13 | 52 | 10.1 | 10.16 | 11.4 | 9.13 |
| Q6 | Ulcer recognition and early wound care | Baidu Zhidao | Red-flag recognition | Grok 4.3 | 58 | 75 | 4 | 0 | 0 | 11.88 | 53 | 10.4 | 9.96 | 12.13 | 9.27 |
| Q7 | Ulcer recognition and early wound care | Chinese guideline | Mechanism explanation | Grok 4.3 | 59 | 85 | 4 | 0 | 0 | 11.62 | 48 | 10.7 | 10.29 | 12.71 | 9.43 |
| Q8 | Ulcer recognition and early wound care | International guideline | Red-flag recognition | Grok 4.3 | 53 | 80 | 4 | 1 | 0 | 13.35 | 40 | 11.2 | 11.34 | 14.5 | 10.21 |
| Q9 | Infection and suspected osteomyelitis | Google Trends | Red-flag recognition | Grok 4.3 | 52 | 75 | 5 | 0 | 0 | 10.91 | 54 | 10.0 | 9.58 | 11.46 | 9.02 |
| Q10 | Infection and suspected osteomyelitis | Baidu Zhidao | Management decision | Grok 4.3 | 64 | 90 | 5 | 2 | 0 | 16.68 | 17 | 16.8 | 14.91 | 18.52 | 13.09 |
| Q11 | Infection and suspected osteomyelitis | Chinese guideline | Red-flag recognition | Grok 4.3 | 58 | 80 | 5 | 1 | 0 | 12.16 | 38 | 12.3 | 10.76 | 14.52 | 9.53 |
| Q12 | Infection and suspected osteomyelitis | International guideline | Management decision | Grok 4.3 | 60 | 70 | 4 | 1 | 0 | 15.44 | 27 | 14.6 | 14.0 | 16.1 | 12.38 |
| Q13 | Ischemia, peripheral artery disease, and vascular referral | Google Trends | Mechanism explanation | Grok 4.3 | 46 | 70 | 4 | 0 | 0 | 17.49 | 20 | 15.3 | 15.44 | 17.6 | 13.25 |
| Q14 | Ischemia, peripheral artery disease, and vascular referral | Baidu Zhidao | Red-flag recognition | Grok 4.3 | 52 | 85 | 4 | 1 | 0 | 14.61 | 37 | 12.1 | 12.17 | 15.13 | 10.44 |
| Q15 | Ischemia, peripheral artery disease, and vascular referral | Chinese guideline | Management decision | Grok 4.3 | 57 | 70 | 4 | 0 | 0 | 16.75 | 24 | 15.3 | 14.25 | 17.64 | 12.56 |
| Q16 | Ischemia, peripheral artery disease, and vascular referral | International guideline | Management decision | Grok 4.3 | 54 | 90 | 5 | 1 | 0 | 16.9 | 26 | 15.3 | 14.45 | 16.78 | 13.01 |
| Q17 | Offloading, wound healing, and surgical management | Google Trends | Management decision | Grok 4.3 | 68 | 85 | 5 | 2 | 0 | 14.59 | 30 | 14.2 | 12.74 | 16.2 | 11.29 |
| Q18 | Offloading, wound healing, and surgical management | Baidu Zhidao | Management decision | Grok 4.3 | 56 | 90 | 4 | 2 | 0 | 14.49 | 34 | 12.9 | 12.59 | 15.68 | 11.22 |
| Q19 | Offloading, wound healing, and surgical management | Chinese guideline | Mechanism explanation | Grok 4.3 | 55 | 70 | 5 | 0 | 0 | 15.11 | 35 | 13.1 | 12.48 | 16.22 | 11.28 |
| Q20 | Offloading, wound healing, and surgical management | International guideline | Management decision | Grok 4.3 | 60 | 85 | 5 | 0 | 0 | 17.76 | 21 | 15.5 | 15.18 | 17.59 | 13.55 |
| Q21 | Charcot foot, amputation risk, recurrence, and follow-up | Google Trends | Red-flag recognition | Grok 4.3 | 55 | 80 | 5 | 0 | 0 | 13.54 | 39 | 12.2 | 11.66 | 14.39 | 10.33 |
| Q22 | Charcot foot, amputation risk, recurrence, and follow-up | Baidu Zhidao | Management decision | Grok 4.3 | 57 | 80 | 4 | 1 | 0 | 15.26 | 31 | 12.7 | 13.15 | 15.76 | 11.34 |
| Q23 | Charcot foot, amputation risk, recurrence, and follow-up | Chinese guideline | Follow-up guidance | Grok 4.3 | 58 | 80 | 5 | 1 | 0 | 15.76 | 36 | 13.5 | 13.04 | 15.46 | 11.83 |
| Q24 | Charcot foot, amputation risk, recurrence, and follow-up | International guideline | Prevention / follow-up guidance | Grok 4.3 | 58 | 70 | 4 | 0 | 0 | 14.6 | 38 | 13.3 | 12.59 | 14.84 | 11.4 |
| Q1 | Risk, prevention, and foot screening | Google Trends | Mechanism explanation | Qwen3.6-Max-Preview | 56 | 80 | 5 | 1 | 0 | 11.1 | 49 | 10.5 | 9.42 | 12.98 | 8.82 |
| Q2 | Risk, prevention, and foot screening | Baidu Zhidao | Red-flag recognition | Qwen3.6-Max-Preview | 57 | 75 | 5 | 0 | 0 | 10.54 | 53 | 10.1 | 9.05 | 12.62 | 8.48 |
| Q3 | Risk, prevention, and foot screening | Chinese guideline | Prevention guidance | Qwen3.6-Max-Preview | 57 | 75 | 5 | 0 | 0 | 11.94 | 46 | 12.8 | 10.41 | 13.56 | 10.25 |
| Q4 | Risk, prevention, and foot screening | International guideline | Prevention guidance | Qwen3.6-Max-Preview | 54 | 85 | 4 | 0 | 0 | 9.59 | 50 | 9.4 | 8.57 | 12.55 | 7.58 |
| Q5 | Ulcer recognition and early wound care | Google Trends | Red-flag recognition | Qwen3.6-Max-Preview | 40 | 70 | 4 | 0 | 0 | 10.54 | 51 | 10.6 | 8.93 | 12.8 | 8.65 |
| Q6 | Ulcer recognition and early wound care | Baidu Zhidao | Red-flag recognition | Qwen3.6-Max-Preview | 48 | 70 | 4 | 0 | 0 | 9.68 | 53 | 9.7 | 8.71 | 11.97 | 8.17 |
| Q7 | Ulcer recognition and early wound care | Chinese guideline | Mechanism explanation | Qwen3.6-Max-Preview | 53 | 75 | 4 | 0 | 0 | 10.37 | 53 | 10.1 | 9.21 | 12.32 | 8.62 |
| Q8 | Ulcer recognition and early wound care | International guideline | Red-flag recognition | Qwen3.6-Max-Preview | 55 | 85 | 5 | 1 | 0 | 12.16 | 44 | 10.8 | 10.77 | 13.56 | 10.01 |
| Q9 | Infection and suspected osteomyelitis | Google Trends | Red-flag recognition | Qwen3.6-Max-Preview | 50 | 75 | 4 | 0 | 0 | 11.12 | 44 | 11.0 | 9.89 | 13.91 | 9.01 |
| Q10 | Infection and suspected osteomyelitis | Baidu Zhidao | Management decision | Qwen3.6-Max-Preview | 60 | 85 | 4 | 2 | 0 | 14.07 | 16 | 16.9 | 13.56 | 18.15 | 10.81 |
| Q11 | Infection and suspected osteomyelitis | Chinese guideline | Red-flag recognition | Qwen3.6-Max-Preview | 49 | 75 | 4 | 0 | 0 | 10.7 | 43 | 11.9 | 10.4 | 12.89 | 9.85 |
| Q12 | Infection and suspected osteomyelitis | International guideline | Management decision | Qwen3.6-Max-Preview | 62 | 70 | 4 | 1 | 0 | 12.47 | 33 | 14.5 | 11.57 | 14.94 | 10.7 |
| Q13 | Ischemia, peripheral artery disease, and vascular referral | Google Trends | Mechanism explanation | Qwen3.6-Max-Preview | 55 | 65 | 4 | 0 | 0 | 16.31 | 10 | 17.1 | 15.03 | 19.72 | 12.21 |
| Q14 | Ischemia, peripheral artery disease, and vascular referral | Baidu Zhidao | Red-flag recognition | Qwen3.6-Max-Preview | 49 | 75 | 4 | 0 | 0 | 11.3 | 45 | 10.6 | 10.25 | 13.36 | 9.22 |
| Q15 | Ischemia, peripheral artery disease, and vascular referral | Chinese guideline | Management decision | Qwen3.6-Max-Preview | 49 | 70 | 4 | 0 | 0 | 11.23 | 52 | 10.3 | 9.2 | 13.46 | 8.68 |
| Q16 | Ischemia, peripheral artery disease, and vascular referral | International guideline | Management decision | Qwen3.6-Max-Preview | 52 | 65 | 4 | 0 | 0 | 11.83 | 36 | 13.7 | 10.79 | 14.82 | 9.66 |
| Q17 | Offloading, wound healing, and surgical management | Google Trends | Management decision | Qwen3.6-Max-Preview | 55 | 70 | 4 | 0 | 0 | 10.18 | 49 | 11.1 | 8.8 | 13.17 | 8.43 |
| Q18 | Offloading, wound healing, and surgical management | Baidu Zhidao | Management decision | Qwen3.6-Max-Preview | 50 | 65 | 4 | 0 | 0 | 14.88 | 36 | 12.0 | 12.82 | 15.31 | 11.41 |
| Q19 | Offloading, wound healing, and surgical management | Chinese guideline | Mechanism explanation | Qwen3.6-Max-Preview | 56 | 65 | 5 | 0 | 0 | 11.93 | 41 | 11.3 | 10.81 | 14.5 | 9.57 |
| Q20 | Offloading, wound healing, and surgical management | International guideline | Management decision | Qwen3.6-Max-Preview | 59 | 75 | 4 | 0 | 0 | 13.34 | 36 | 13.8 | 11.6 | 15.58 | 10.85 |
| Q21 | Charcot foot, amputation risk, recurrence, and follow-up | Google Trends | Red-flag recognition | Qwen3.6-Max-Preview | 47 | 75 | 4 | 0 | 0 | 11.84 | 36 | 12.2 | 11.1 | 14.47 | 9.69 |
| Q22 | Charcot foot, amputation risk, recurrence, and follow-up | Baidu Zhidao | Management decision | Qwen3.6-Max-Preview | 44 | 75 | 4 | 0 | 0 | 13.66 | 31 | 13.6 | 12.04 | 16.7 | 10.53 |
| Q23 | Charcot foot, amputation risk, recurrence, and follow-up | Chinese guideline | Follow-up guidance | Qwen3.6-Max-Preview | 50 | 65 | 4 | 0 | 0 | 13.04 | 39 | 12.5 | 11.23 | 14.91 | 10.16 |
| Q24 | Charcot foot, amputation risk, recurrence, and follow-up | International guideline | Prevention / follow-up guidance | Qwen3.6-Max-Preview | 62 | 80 | 5 | 1 | 0 | 14.74 | 37 | 12.7 | 12.3 | 15.62 | 11.27 |

Note. This table provides final adjudicated scores and readability values for each item-by-model response pair to support transparency and reproducibility. The complete verbatim model-generated response text for all 24 benchmark questions across the five evaluated models is provided in Supplementary Appendix A of this Supplementary Material.

Supplementary Appendix A. Complete verbatim model-generated response text

This appendix provides the final standardized benchmark prompts exactly as used for model querying and the complete verbatim model-generated response text from GPT-5.5 Thinking, DeepSeek-V4, Gemini 3.1 Pro, Grok 4.3, and Qwen3.6-Max-Preview for all 24 benchmark questions. The appendix contains 120 model responses in total. The model responses are provided as originally generated and were not rewritten, summarized, translated, shortened, selectively excerpted, or selectively replaced. Minor formatting was applied only to organize the responses by question and model and did not alter the wording of the response text.

This appendix was prepared after completion of blinded scoring to support transparency and reproducibility. It was not the blinded scoring file provided to physician raters. Before blinded evaluation, model names, spreadsheet column headers, interface labels, logos, timestamps, response order, hyperlinks, URLs, URL tracking parameters, link-preview elements, and other model- or interface-identifying metadata were removed from the scoring materials.

Q1. How do I know if I am at high risk for diabetic foot problems?

GPT-5.5 Thinking:

You may be high risk for diabetic foot problems if any of these apply:

High-risk history

You have ever had a diabetic foot ulcer.
 You have ever had a toe, foot, or leg amputation.
 You are on dialysis or renal replacement therapy. NICE classifies these as high-risk features.

Nerve + circulation problems

You have neuropathy: numbness, tingling, burning, reduced feeling, or injuries you do not feel.
 You have poor circulation/peripheral arterial disease: weak foot pulses, leg pain when walking, cold feet, slow-healing wounds, or color changes.
 Risk is especially high when neuropathy and poor circulation occur together, or when either is combined with calluses or foot deformity.

Foot changes or warning signs

New or worsening calluses/corns, nail deformity, bleeding under a nail, new foot pain, swelling, limp, change in foot shape, or shoes suddenly not fitting well are common signs worth reporting.
 Be careful: foot complications may start without obvious symptoms, especially if sensation is reduced.

Seek urgent care now for any foot ulcer, infection, spreading redness, fever, pus, black tissue/gangrene, severe pain, or a hot swollen foot with color change, even if it does not hurt. NICE treats ulceration, infection, limb-threatening ischemia, gangrene, and suspected Charcot foot as active diabetic foot problems.

The most reliable way to know your risk is to have a clinician do a diabetic foot exam. For adults with diabetes, NICE recommends foot-risk assessment at diagnosis, at least annually, whenever foot problems arise, and during hospital admission; the exam checks sensation, circulation, ulcers, callus, infection/inflammation, deformity, gangrene, and Charcot changes. CDC also recommends yearly comprehensive foot exams, including pulses, sensation, foot structure/function, and nails.

A practical next step: ask your diabetes clinician or a podiatrist, “What is my foot-risk category, and how often should I be checked?” Moderate- or high-risk patients often need more frequent follow-up than once a year.

DeepSeek-V4:

This is an important question, and being proactive about it is the best way to prevent serious complications. I’ll walk you through the key risk factors, but please remember: this information is for education only and not a substitute for a professional foot exam.

A “high-risk” foot is generally one that has lost its ability to feel pain or has poor blood flow, making it vulnerable to injury and slow to heal. You can assess your risk by looking at these key areas:

1. Loss of Protective Sensation (Neuropathy)
This is the single biggest risk factor. When nerves are damaged, you may not feel a blister, cut, or even a pebble in your shoe. A small, unnoticed injury can quickly become a serious wound.

Ask yourself:
 Do your feet feel numb, like you’re walking on cotton wool?
 Do you have tingling, burning, or pins-and-needles sensations, especially at night?
 Can you feel a light touch? Try this at-home test: Take a thin, flexible object (like the "touch-the-toes" test with a monofilament from your doctor, or carefully use a single bristle from a soft brush). Can you feel it when it's gently pressed against the bottom of your big toe and foot? If not, you have significant loss of protective sensation.
 Can you distinguish hot from cold on your feet?

2. Poor Circulation (Peripheral Artery Disease - PAD)
Good blood flow is essential for healing. Diabetes can cause narrowing of the arteries in the legs, reducing circulation.

Look for these signs:
 Pain in your calves, thighs, or buttocks when walking that eases with rest (claudication).
 Cold feet, especially if one foot is noticeably colder than the other.
 Shiny, thin skin on your feet and lower legs.
 Loss of hair on your toes and lower legs.
 Slow-healing cuts or sores.
 A pale or bluish color to your toes when elevated.

3. Foot Deformities and Skin Changes
Physical changes create high-pressure areas that are prone to breakdown.

Check your feet for:
 Corns and calluses: These are signs of high, repetitive pressure. If you have neuropathy, they can break down into ulcers underneath.
 Bunions or hammertoes: These create pressure points that rub against footwear.
 Charcot foot: A rare but serious complication causing swelling, redness, and eventual collapse of the foot’s arch. A hot, swollen foot demands an immediate doctor’s visit.
 Very dry, cracked skin (fissures): Especially on the heels, these cracks can become an entry point for infection.

4. History of a Previous Ulcer or Amputation
This is the strongest predictor of future problems. If you’ve had a diabetic foot ulcer or any part of your foot or leg amputated before, you are automatically in the highest-risk category for recurrence, often for life.

5. Other Major Red Flags
 Long-standing, poorly controlled diabetes: High blood sugar over many years damages nerves and vessels.
 Kidney disease (nephropathy): Having diabetes-related kidney disease is strongly linked to foot disease.
 Eye problems (retinopathy): If diabetes is affecting the small blood vessels in your eyes, it’s likely affecting your feet too.
 Inability to perform self-care: Poor vision (you can’t see a cut), obesity (you can’t reach your feet), or living alone without support all significantly increase risk.

The "High-Risk" Checklist: When to Seek a Professional Exam Immediately
You should see a doctor or podiatrist urgently if you check any of the following boxes:

You have a history of a foot ulcer or amputation.
 You cannot feel the monofilament test or a light touch on your feet.
 You have a loss of sensation combined with a callus, blister, or deformity.
 You have signs of poor circulation (cold feet, leg pain when walking, pale/blue skin).
 You have a visible foot deformity (bunion, Charcot foot).
 You have an open sore, bleeding corn, cracked heel, or an infected toenail right now.
 Your foot has suddenly become red, hot, and swollen (suspected Charcot or infection).

How a Clinician Determines Your Risk
When you go for your annual checkup, your provider will categorize you like this:

Low Risk: Normal sensation, palpable pulses, no deformity. (Annual exam needed.)
 Medium Risk: Loss of sensation (neuropathy) OR poor circulation OR a deformity. (Exam every 6 months, foot care education.)
 High Risk: Neuropathy or poor circulation plus a deformity, or a history of ulcer/amputation. (Exam every 3-6 months, specialist referral, therapeutic footwear.)

The Most Important Thing You Can Do Right Now
If you don’t already, make a daily foot check a non-negotiable habit. Every single day, inspect the bottoms, sides, and between your toes (using a mirror or a helper) for anything new: a cut, blister, swelling, change in color, or dry crack. If you see something that doesn’t heal in 24 hours, call your doctor.

Disclaimer: This information is for educational purposes and not a substitute for professional medical advice. If you are concerned about your feet, please see a doctor or podiatrist immediately, especially if you have an open wound or signs of infection.

Gemini 3.1 Pro:

Determining your risk for diabetic foot problems involves evaluating specific physical symptoms, your medical history, and lifestyle factors. Diabetic foot complications typically arise from a combination of nerve damage (neuropathy) and poor blood circulation (ischemia).

Here are the primary indicators that put an individual at a high risk for diabetic foot problems:

Primary Warning Signs

Loss of Sensation (Neuropathy): Numbness or an inability to feel heat, cold, or pain in your toes or feet. This is the most significant risk factor because you may not notice a cut or blister.
 Tingling or Burning Sensations: Often described as "pins and needles," which can be particularly noticeable or painful at night.
 Changes in Skin Color or Temperature: Feet that consistently feel cold to the touch, or skin that appears unusually red, blue, or pale, indicating poor circulation.
 Slow-Healing Wounds: Minor cuts, scratches, or blisters that take weeks to heal or frequently become infected.
 Dry, Cracked Skin: Autonomic nerve damage can reduce sweating in the feet, leading to severe dryness, peeling, and cracking that invites infection.

Medical Risk Factors

Poor Glycemic Control: Consistently high blood sugar levels (an elevated HbA1c) accelerate both nerve damage and the narrowing of blood vessels over time.
 History of Ulcers or Amputation: A previous diabetic foot ulcer or a prior lower-extremity amputation is one of the strongest predictors of future foot problems.
 Foot Deformities: Structural issues such as bunions, hammertoes, flat feet, or Charcot foot create abnormal pressure points that easily turn into ulcers when wearing standard shoes.
 Kidney Disease: Diabetic nephropathy (kidney disease) is strongly correlated with an increased risk of severe foot complications.
 Vision Impairment: Eye conditions caused by diabetes can make it difficult to adequately see and inspect the bottoms of your feet for early signs of trauma.

Lifestyle Risk Factors

Smoking: Tobacco use constricts blood vessels and severely impairs circulation to the extremities, multiplying the vascular damage caused by diabetes.
 Improper Footwear: Wearing shoes that are too tight, lack adequate support, or chafe the skin can cause friction injuries that go unnoticed due to neuropathy.

Note: This information is for educational purposes and does not substitute for professional medical assessment. If you have diabetes, clinical guidelines recommend that a healthcare provider conduct a comprehensive, standardized diabetic foot examination at least once a year.

Grok 4.3:

You may be at high risk for diabetic foot problems (such as ulcers, infections, or even amputation) if you have specific risk factors, symptoms of nerve or circulation damage, or a history of foot issues. People with diabetes face up to a 25% lifetime risk of developing a foot ulcer, largely due to neuropathy (nerve damage causing loss of protective sensation) and peripheral artery disease (PAD) or poor circulation, which impair healing.

The most reliable way to know your personal risk is through a professional comprehensive foot exam (recommended at least annually by the American Diabetes Association and others), not self-diagnosis alone. Early identification allows preventive steps that dramatically reduce complications.

Key Risk Factors
You are more likely to be at high risk if any of these apply (many interact and multiply danger):

Previous foot ulcer or amputation — This is the strongest predictor and places you in the highest-risk category.
 Loss of protective sensation (LOPS/neuropathy) — You cannot feel pain, temperature, or pressure normally.
 Poor circulation (PAD) — Reduced blood flow to the feet.
 Foot deformities — Such as bunions, hammertoes, Charcot foot (sudden collapse of foot bones), or prominent calluses/bony areas that create pressure points.
 Long duration of diabetes, poorly controlled blood sugar (high A1C), or other complications (retinopathy/vision loss, nephropathy/kidney disease especially on dialysis, heart disease).
 Smoking, older age, obesity, ill-fitting shoes, or walking barefoot.

Symptoms and Warning Signs
Many people have no pain due to neuropathy, so problems can silently worsen. Watch for:

Neuropathy-related: Numbness, tingling, burning, “pins and needles,” or a “bugs crawling” sensation (often worse at night); loss of feeling (e.g., cannot feel a light touch or temperature changes); muscle weakness or balance problems.
 Circulation-related (PAD): Leg/foot cramping or pain when walking that eases with rest; one foot colder or paler/bluish than the other; slow-healing sores; hair loss on legs/toes; poor toenail growth.
 Visible or structural issues: Cuts, blisters, sores, or ulcers that do not heal; redness, swelling, warmth (especially one foot much hotter — possible Charcot or infection); dry/cracked skin; thick/yellow toenails; corns/calluses; changes in foot shape; shoes no longer fitting well; foul odor or discharge from a wound.

Seek immediate medical care (same day or ER) for any open wound, signs of infection (increasing redness, pus, fever, chills), sudden swelling/redness/warmth/pain in the foot, or black/discolored tissue (possible gangrene).

How Doctors Assess and Categorize Risk
A trained provider performs a comprehensive diabetic foot exam including:
 History (prior ulcers/amputations, symptoms, self-care habits).
 Visual inspection (skin, nails, deformities, footwear).
 Sensory testing — 10-gram monofilament (Semmes-Weinstein) on multiple foot sites; if you cannot feel it in several areas, you have LOPS.
 Circulation check — Feeling foot pulses (dorsalis pedis and posterior tibial); ankle-brachial index (ABI) if needed.
 Other tests — Vibration sense (tuning fork), reflexes, or imaging if indicated.

They then use the International Working Group on the Diabetic Foot (IWGDF) risk stratification (adopted in ADA Standards of Care):

Category 0 (Very low risk): No LOPS and no PAD → Annual exam + basic education.
 Category 1 (Low risk): LOPS or PAD → Exam every 6–12 months.
 Category 2 (Moderate risk): LOPS + PAD, or LOPS + deformity, or PAD + deformity → Exam every 3–6 months; may need special footwear or podiatry referral.
 Category 3 (High risk): LOPS or PAD plus history of ulcer/amputation or end-stage kidney disease → Exam every 1–3 months; intensive prevention (therapeutic shoes, frequent monitoring, possible vascular or surgical input).

Your provider will recommend follow-up frequency and referrals (e.g., to podiatry, vascular specialist, or wound care) based on this.

What You Should Do Right Now
1. Schedule a foot exam — Ask your primary doctor or endocrinologist for a full diabetic foot check today (or request referral to a podiatrist). Get one at every visit if high-risk.
2. Perform daily self-checks — Inspect feet (use a mirror or ask for help) for cuts, redness, swelling, or anything unusual, including between toes. Report anything new immediately.
3. Optimize prevention:
 Keep blood sugar, blood pressure, and cholesterol in target ranges.
 Quit smoking (it severely worsens circulation).
 Wear well-fitting, supportive shoes and seamless socks; never walk barefoot.
 Wash and moisturize feet daily (avoid between toes); trim nails straight across.
 Stay active as approved by your doctor.

High risk is manageable — With good control and care, most people avoid serious problems. This information is for educational purposes and draws from guidelines by the ADA, IWGDF, CDC, and Cleveland Clinic. It is not personalized medical advice. Please discuss your specific situation with your healthcare provider promptly for testing and a tailored plan. Early action saves feet and lives.

Qwen3.6-Max-Preview:

Disclaimer: I'm an AI, not a healthcare provider. The information below is based on current clinical guidelines (ADA, IWGDF) and should not replace a professional medical evaluation. Always consult your doctor, podiatrist, or diabetes care team for personalized risk assessment.

Diabetic foot problems (ulcers, infections, nerve damage, poor circulation, and in severe cases, amputation) develop gradually and are highly preventable when risk is identified early. Here's how to understand your risk level:

Key Factors That Increase Your Risk
You're generally considered higher risk if you have one or more of the following:
 Peripheral neuropathy: Numbness, tingling, burning, or loss of protective sensation in your feet. You may not feel cuts, blisters, or pressure points.
 Peripheral artery disease (PAD): Poor blood flow to the legs/feet. Signs include cold feet, leg cramps when walking, slow-healing wounds, shiny skin, or weak/absent foot pulses.
 Previous foot ulcer or amputation: A history of either is one of the strongest predictors of future problems.
 Foot deformities or structural changes: Bunions, hammertoes, Charcot foot, limited ankle mobility, or abnormal pressure points.
 Long-standing or poorly controlled diabetes: Duration >10 years, or consistently elevated HbA1c above your target range.
 Diabetic kidney disease: Especially moderate-to-severe CKD or dialysis, which impairs healing and immunity.
 Vision impairment: Diabetic retinopathy, cataracts, or glaucoma that make daily foot inspection difficult.
 Lifestyle & care factors: Smoking, heavy alcohol use, walking barefoot, ill-fitting shoes, poor foot hygiene, or untreated fungal infections/calluses.

How Clinicians Classify Risk
Most providers use a simple tiered system (aligned with ADA/IWGDF guidelines):
 Low risk: No neuropathy, no PAD, no deformities, no prior ulcers/amputations.
 Moderate risk: Neuropathy OR PAD OR foot deformity/history of minor foot issues.
 High risk: Neuropathy + PAD, OR previous ulcer/amputation, OR multiple risk factors combined.
 Very high/active risk: Current ulcer, infection, or Charcot changes.

During a visit, your provider will typically:
 Test sensation with a 10g monofilament & tuning fork
 Check foot pulses & skin temperature
 Inspect skin, nails, calluses, and footwear
 Assess gait, pressure points, and joint mobility

What You Can Do Now
1. Get a comprehensive foot exam at least once a year (every 3–6 months if moderate/high risk).
2. Check your feet daily using a mirror or phone camera if needed. Look for cuts, blisters, redness, swelling, drainage, or shape changes.
3. Keep diabetes & comorbidities managed: Target HbA1c, blood pressure, cholesterol, and kidney health as advised by your care team.
4. Wear protective, well-fitted shoes and clean, moisture-wicking socks. Never walk barefoot, even indoors.
5. Avoid DIY foot surgery: Don't cut calluses, use chemical corn removers, or soak feet in hot water. See a professional for nail/skin care if you have neuropathy or PAD.
6. Quit smoking: It dramatically worsens circulation and healing.

When to Seek Prompt Medical Care
Contact a provider same-day or go to urgent care/ER if you notice:
 Any open sore, blister, or cut that isn't improving within 24–48 hours
 Redness, warmth, swelling, pus, foul odor, or spreading discoloration
 Sudden foot swelling, shape change, or "rocker-bottom" appearance (possible Charcot)
 Black, blue, or pale/cold toes
 Fever, chills, or feeling unwell alongside foot symptoms

Bottom Line
Risk isn't all-or-nothing; it's a spectrum that changes over time. Even if you feel fine, nerve damage and circulation changes can be silent. A quick clinical foot assessment can place you in the right risk category and trigger a personalized prevention plan. If you'd like, tell me your diabetes type/duration, recent HbA1c, any foot symptoms, and other health conditions, and I can help you understand which factors may apply to you (though a formal exam is still essential).

Q2. My feet feel numb, tingling, or less sensitive. Could this be dangerous if I have diabetes?

GPT-5.5 Thinking:

Yes. Numb, tingling, or less-sensitive feet can be dangerous if you have diabetes, because it may be diabetic peripheral neuropathy—nerve damage that often starts in the feet. Diabetes can also reduce blood flow, so a blister, cut, burn, or pressure sore may go unnoticed, heal poorly, become infected, and in severe cases lead to gangrene or amputation.

Contact your diabetes clinician or primary care doctor soon, especially if this is new, worsening, or you have not had a recent foot exam. The ADA describes tingling, burning/stinging pain, weakness, and loss of feeling as common foot neuropathy symptoms, and recommends medical evaluation for foot problems.

Seek urgent care today if you notice any foot wound, open sore, drainage, bad odor, spreading redness, warmth, swelling, black/blue skin, fever, or severe new foot pain. Those can be signs of infection, poor circulation, or serious diabetic foot complications.

Call emergency services now if the numbness came on suddenly on one side of the body, or with face drooping, arm/leg weakness, confusion, trouble speaking, vision changes, trouble walking, or a sudden severe headache—those can be stroke symptoms.

For now: check both feet daily, including between toes and the soles; avoid walking barefoot; wear well-fitting shoes; do not use heating pads or hot water on numb feet; and keep your glucose management plan on track. Numbness can also come from causes besides diabetes, such as vitamin B12 deficiency, thyroid disease, circulation problems, alcohol, medications, or a pinched nerve, so it is worth getting evaluated rather than assuming.

DeepSeek-V4:

Yes, this can be very dangerous. What you’re describing sounds like peripheral neuropathy, a common but serious diabetes complication. The danger isn’t just the numbness itself, but what that loss of sensation can lead to.

You should see a doctor promptly, but not panic. Here’s why it’s dangerous and what to do right now.

The 3 Main Dangers

Think of pain as your body’s alarm system. When your feet are numb, that alarm is switched off.

1. Silent Injuries & Infections
You might not feel a small cut, blister, or even a pebble in your shoe. A minor, unfelt injury can quickly become infected. Because diabetes can also reduce blood flow, your body struggles to heal that infection. What starts as a tiny blister can escalate to a serious, limb-threatening ulcer without you feeling a thing.

2. Undetected Burns or Frostbite
Without proper sensation, testing bathwater with your foot is impossible. You can suffer a severe burn. Similarly, you might not feel cold damage, or use a heating pad too hot, causing burns without realizing it.

3. Charcot Foot (A Medical Emergency)
This is a condition where the bones in the foot weaken and fracture, often without noticeable pain. Because you can’t feel it, you continue walking on the broken foot, causing the bones to shift and the joint to collapse. The foot can become deformed, making it prone to more sores. If one foot is suddenly swollen, red, and feels warmer than the other, seek emergency care immediately.

What To Do Right Now

Your priority is a doctor’s assessment, but you can take these steps immediately to protect yourself.

1. Inspect Your Feet Every Single Day
Check every surface twice daily: tops, soles, between the toes, and your heels. Use a mirror or ask someone for help if you can’t see the entire sole. Look for:- Cuts, blisters, or sores
 Redness, swelling, or warmth
 Ingrown toenails
 Dry, cracked skin
 Changes in shape or color

2. Never Go Barefoot
Always wear shoes and clean, dry, well-fitting socks, even indoors. This is your primary defense against injuries you can’t feel. Before putting shoes on, feel inside with your hand for pebbles, rough seams, or small objects.

3. Stop Using Heat or Soaking
Do not use heating pads, hot water bottles, or soak your feet. You can’t accurately gauge the temperature and risk a severe burn. Wash your feet daily with warm (not hot) water, check the temperature with your elbow, dry them gently but thoroughly, especially between the toes, and apply lotion to keep skin from cracking, but avoid between the toes.

4. Get Professional Care
 See your doctor soon. They can confirm if it's neuropathy, rule out other causes, and check your blood flow. They can also provide pain relief options if needed.
 Never self-treat corns or calluses. Chemical removers can burn your skin. A podiatrist (foot doctor) should handle this.
 Seek urgent care today if you find any open sore, red streaks, pus, or if your foot is red, hot, and swollen.

While diabetes is the most common cause, your doctor may also check for vitamin deficiencies (especially B12) or other possible factors.

Your feet are vulnerable, but with a strict daily care routine and medical guidance, most serious complications are preventable. The key is starting today.

Disclaimer: I am an AI, not a doctor. This information is for educational purposes and does not replace urgent, professional medical advice.

Gemini 3.1 Pro:

Yes, experiencing numbness, tingling, or a loss of sensation in your feet can be very dangerous if you have diabetes. It is a highly common complication, but it requires prompt medical evaluation to prevent severe consequences.

These symptoms are the hallmark of Diabetic Peripheral Neuropathy, a type of nerve damage caused by prolonged periods of high blood sugar.

Here is a straightforward look at why this symptom is considered dangerous and what it can lead to if left unmanaged:

Why Loss of Sensation is Dangerous

Unnoticed Injuries: The most immediate danger of losing feeling in your feet is that you lose your body's alarm system. You might step on a sharp object, develop a blister from tight shoes, or sustain a cut without ever feeling it.
 Poor Healing and Infection: Diabetes often affects blood circulation, particularly to the extremities. If you sustain a minor, unnoticed injury and have poor blood flow, that wound will heal much slower. This creates a prime environment for severe bacterial infections.
 Foot Ulcers: An untreated, infected wound can quickly deteriorate into a diabetic foot ulcer (a deep open sore). Ulcers are notoriously difficult to treat and heal.
 Risk of Amputation: If an infection from an ulcer reaches the bone or causes tissue death (gangrene), surgical amputation of the toe, foot, or lower leg may become the only life-saving option.
 Charcot Foot: In severe cases of neuropathy, the bones and joints in the foot can weaken and collapse. Because the person cannot feel the pain, they continue to walk on it, leading to severe deformity.

Immediate Next Steps

Because the risks associated with diabetic neuropathy compound quickly, it is important to take proactive steps:

1. See a Doctor Immediately: Schedule an appointment with your primary care physician or an endocrinologist as soon as possible. They can perform a comprehensive foot exam to assess your nerve function and circulation.
2. Inspect Your Feet Daily: Until you see a doctor, make it a habit to visually inspect your feet every single day. Look for cuts, blisters, redness, swelling, or changes to your nails. Use a mirror if you cannot easily see the bottom of your feet.
3. Protect Your Feet: Never walk barefoot, even indoors. Wear well-fitting, supportive shoes and clean, dry socks to minimize friction and prevent injury.
4. Manage Blood Sugar: While nerve damage is difficult to reverse, keeping your blood glucose levels tightly managed within your target range is the most effective way to prevent the neuropathy from worsening.

Please treat these symptoms seriously and prioritize getting a professional medical evaluation to protect your foot health.

Grok 4.3:

Yes, these symptoms—numbness, tingling, or reduced sensitivity in the feet—can be dangerous if you have diabetes. They are classic signs of diabetic peripheral neuropathy (nerve damage caused by high blood sugar), one of the most common complications of diabetes. It often starts in the feet and legs because those nerves are longest and farthest from the heart and spine.

Why This Is Serious
High blood sugar over time damages nerves and blood vessels. When sensation is lost (especially the ability to feel pain, pressure, or temperature), you may not notice minor injuries like cuts, blisters, ingrown toenails, or pressure sores from shoes. These can quickly turn into ulcers, become infected, spread to bone (osteomyelitis), or lead to tissue death (gangrene). In severe cases, this can require amputation of a toe, foot, or leg.

Diabetes also often impairs circulation, which slows healing and raises infection risk. Neuropathy affects up to 50–70% of people with diabetes and can develop gradually (sometimes unnoticed until advanced).

Other possible causes exist (vitamin B12 deficiency, medications, alcohol, thyroid issues, or spine problems), but with diabetes, neuropathy is the leading suspect and must be ruled in or out promptly.

What You Should Do Right Now
See a doctor as soon as possible—ideally today or tomorrow if symptoms are new, worsening, or accompanied by any of these red flags:
 Open sores, redness, swelling, warmth, pus, or foul odor
 Black or discolored skin
 Fever, chills, or feeling unwell
 Pain that suddenly returns (sometimes a sign of worsening damage)
 Changes in foot shape or balance issues

Do not wait for your next routine appointment. Contact your primary care doctor, endocrinologist, or a podiatrist experienced with diabetes. The American Diabetes Association recommends annual comprehensive foot exams (starting at diagnosis for type 2 diabetes) and immediate evaluation for any neuropathy symptoms.

In the meantime:
 Inspect your feet daily (use a mirror or ask for help; check between toes and soles).
 Wear well-fitting, cushioned shoes and clean cotton socks—never go barefoot, even indoors.
 Keep blood sugar in target range (this can slow progression).
 Avoid smoking (it worsens circulation).
 Elevate feet when resting and avoid crossing legs for long periods.

What to Expect at the Doctor
They will likely:
 Perform simple tests (10-gram monofilament for touch sensation, tuning fork for vibration, reflexes).
 Check for ulcers or circulation problems (pulses, skin temperature/color).
 Possibly order nerve studies, blood work, or imaging.
 Discuss blood sugar optimization, medications for nerve pain (if present), or referral to a specialist.

There is no cure for established neuropathy, but early action prevents complications. Many people manage symptoms and protect their feet successfully with good control and care.

Important disclaimer: This is general information based on medical sources (Mayo Clinic, CDC, American Diabetes Association, Cleveland Clinic) and not personalized medical advice. Only a qualified healthcare professional can diagnose and treat you after examining you and reviewing your full history and labs. Please seek care promptly—diabetic foot complications are highly preventable with timely attention.

If you share more details (how long symptoms have lasted, your diabetes type/duration/control, other symptoms, or medications), I can help point you toward more targeted questions to ask your doctor. Take care of those feet—they're critical for mobility and independence.

Qwen3.6-Max-Preview:

Yes, what you're describing can be a sign of diabetic peripheral neuropathy, a common nerve complication of diabetes. While it may start as mild tingling or numbness, it can become dangerous if left unmanaged, so it's important to take it seriously and have it evaluated by a healthcare professional.

Why it can be concerning:
 Loss of protective sensation: When you can't properly feel pain, pressure, or temperature, minor injuries (blisters, cuts, ingrown toenails, or friction sores) can go unnoticed.
 Slower healing & infection risk: Diabetes often affects circulation and immune response. Combined with nerve damage, this increases the chance that small wounds become infected or develop into foot ulcers.
 Serious complications: Untreated ulcers or infections can lead to deep tissue damage, bone infections, or in severe cases, amputation.
 Balance & fall risk: Reduced sensation can impair your awareness of foot placement, increasing trips, falls, or joint damage (like Charcot foot).

What you should do:
 See your doctor or diabetes care team promptly. They can confirm whether this is neuropathy, check your blood sugar control, assess circulation, and rule out other causes (e.g., B12 deficiency, thyroid issues, or spinal nerve compression).
 Inspect your feet daily. Look for cuts, redness, swelling, blisters, calluses, or nail changes. Use a mirror or ask for help if needed.
 Keep blood glucose in your target range. Consistent management is the most proven way to slow or prevent further nerve damage.
 Always wear protective footwear. Well-fitting shoes and clean socks, even indoors. Never walk barefoot.
 Ask about treatment options. Your provider may discuss medications for nerve symptoms, custom footwear, physical therapy, or referrals to a podiatrist or endocrinologist.

Seek prompt medical care if you notice:
 Any open sore, ulcer, redness, warmth, swelling, or drainage on your feet
 Sudden worsening of numbness, weakness, or difficulty walking
 Fever alongside a foot wound
 Changes in foot shape or new deformities

Note: I'm an AI, not a healthcare provider. Numbness or tingling in the feet when you have diabetes should always be evaluated by a qualified clinician. Early assessment and consistent foot care can significantly reduce risks and help you stay active and healthy.

Would you like help preparing questions to ask your doctor, or guidance on daily foot care routines?

Q3. If my feet feel fine, why do I still need regular foot checks?

GPT-5.5 Thinking:

Even when your feet feel fine, regular foot checks matter because some serious foot problems can start without pain.

If this is about diabetes, the main reasons are:

1. You may not feel an injury. Diabetes can damage nerves in the feet, causing loss of sensation. The CDC specifically says to check your feet every day “even if they feel fine” so problems can be caught early.

2. Small problems can become big quickly. A blister, crack, callus, ingrown nail, or shoe rub can turn into an ulcer or infection, especially if circulation is reduced. The American Diabetes Association notes that neuropathy, poor circulation, ulcers, and infections are why monitoring foot health is important.

3. Circulation and healing may be impaired. Poor blood flow can make cuts or sores heal slowly and increases the risk of infection. NIDDK warns that untreated ulcers or gangrene can sometimes lead to amputation.

4. A clinician checks things you cannot easily assess. A regular foot exam can test sensation, blood flow, skin changes, nail problems, pressure points, and foot shape changes. CDC guidance says yearly comprehensive foot exams can help avoid or delay complications.

A good rule: check your own feet daily for cuts, redness, swelling, blisters, sores, cracks, or color changes, and keep your scheduled clinical foot checks. Seek care promptly for any wound that is not healing, spreading redness, warmth, swelling, drainage, black/blue/pale color change, new numbness, or sudden pain.

DeepSeek-V4:

That’s a totally logical question—if nothing hurts, why check? The short answer is that our feet often don’t warn us when something is going wrong. Many serious foot problems are painless in their early stages, and catching them before you feel anything can prevent infections, deformities, or even amputations down the line.

Here’s why feeling “fine” isn’t always reliable:

1. Nerve Damage Silences Pain
This is the single most important reason, especially if you have diabetes, but it can happen with other conditions too. Peripheral neuropathy gradually kills the nerves that send pain, temperature, and touch signals.
 The danger: You could have a blister from new shoes, a splinter, a burn from hot pavement, or even a stress fracture and feel absolutely nothing. A small, unfelt wound can quietly become a serious, infected ulcer.
 No pain doesn’t mean no damage. Regular checks let you visually catch these silent injuries.

2. Circulation Problems Can Be Sneaky
Poor blood flow (Peripheral Artery Disease) often develops slowly and without pain at rest. The skin may look a little shinier, the foot might feel cooler, or hair might stop growing on the toes—subtle changes you could easily dismiss as “normal for me.” Reduced blood flow means any small injury heals slowly and is far more likely to become infected.

3. Skin Cancer and Growths Hide
The bottom of the foot is a common site for melanoma, but because people rarely examine their own soles, it’s often diagnosed late. Other things like unusual moles, warts, or fungal infections can also start so gradually that you don’t notice the change day to day. A trained eye sees the difference immediately.

4. Silent Structural Shifts
Your feet change shape over time due to aging, weight changes, or subtle arthritis. A developing bunion, hammertoe, or flatfoot deformity won’t necessarily hurt until it’s well-established and harder to manage with conservative measures like orthotics. Catching these early can keep you comfortable and mobile for years to come.

5. Preventative Care Is Easier (and Cheaper)
Think of it like dental check-ups—you go even when nothing hurts, because a tiny cavity caught early is a quick fill, not a root canal. With feet, a small callus can be safely reduced before it cracks and gets infected. Thickened nails can be trimmed professionally before they become ingrown and painful. These minor interventions prevent major emergencies.

The bottom line: If you’re in a high-risk group (diabetes, neuropathy, poor circulation, vision problems that make self-checks difficult), professional foot checks are essential, not optional. But even for healthy people, an annual check is a fast way to catch hidden issues like skin cancer, fungal nails, or structural changes before they start hurting. Your feet carry you through life—checking them when they feel fine is how you make sure they stay that way.

Gemini 3.1 Pro:

The main reason you need regular foot checks—even when everything feels perfectly fine—is that certain conditions can "silence" your body's alarm system.

Pain is how your body tells you something is wrong. But if nerve damage (neuropathy) begins to develop, you might lose the ability to feel pain, heat, or cold in your feet. Here is a breakdown of why regular visual and physical inspections are crucial.

1. The Danger of Neuropathy (Nerve Damage)

Neuropathy is most commonly associated with diabetes, but can also be caused by alcohol use, vitamin deficiencies, or autoimmune conditions. If nerves in your feet are damaged:

You could step on a tack, get a blister from tight shoes, or cut yourself without feeling a thing.
 Because you don't feel it, you won't naturally limp or take weight off the foot, which drives the injury deeper.
 By the time you notice an issue visually, a minor scrape may have already turned into a severe infection or ulcer.

2. Poor Circulation Complications

Conditions that cause nerve damage often go hand-in-hand with impaired blood flow (peripheral artery disease).

Blood carries the oxygen and nutrients needed to heal wounds.
 If your circulation is poor, a tiny, unnoticed cut can take months to heal, making it highly susceptible to infection. Regular checks catch these tiny wounds before they escalate.

3. Structural and Skin Changes

Charcot Foot: Nerve damage can weaken the bones in your feet. You could literally fracture a bone in your foot and keep walking on it because you don't feel the pain, leading to severe deformity.
 Dry Skin and Calluses: Nerve damage also affects the nerves that control oil and moisture in your feet. This leads to severely dry, cracked skin and heavy calluses, which can break down and turn into ulcers if not professionally managed.

What a Foot Check Accomplishes

When a doctor examines your feet, they aren't just looking for cuts. They are actively testing:

Sensation: Using tools like a monofilament (a piece of nylon wire) to see if you can feel light pressure.
 Blood flow: Checking the pulses in your feet to ensure adequate circulation.
 Structure: Looking for changes in the shape of your foot that might require supportive footwear to prevent future pressure ulcers.

Ultimately, regular foot checks are a purely preventative measure. They allow you to catch the very early signs of nerve damage or circulation issues before a painless, hidden injury has the chance to become a major medical emergency.

Grok 4.3:

Even if your feet feel completely fine—with no pain, tingling, or obvious issues—you may still need regular foot checks because of conditions like diabetes that can silently damage nerves and blood vessels. This is one of the most common reasons for this medical advice.

Why sensation loss makes checks essential
High blood sugar over time can cause diabetic peripheral neuropathy, affecting up to about half of people with diabetes. It damages nerves (often starting in the feet and legs), leading to reduced or complete loss of feeling for pain, heat, cold, or pressure.

You might step on a sharp object, develop a blister from ill-fitting shoes, or get a small cut without noticing it. Poor circulation (another diabetes complication) then slows healing, allowing minor issues to progress into open sores (ulcers), deep infections, or even tissue death (gangrene). In severe cases, this can lead to amputation of a toe, foot, or leg.

The key point: Pain is your body’s early warning system. When neuropathy removes that warning, problems stay hidden until they’re advanced. Many people with significant nerve damage report “my feet feel fine” right up until a serious complication appears.

What regular checks actually catch
Professional foot exams (recommended at least once a year by the American Diabetes Association, more often if you have prior issues or high risk) go far beyond what you can feel:

Skin and nail inspection for hidden cuts, blisters, calluses, cracks, redness, swelling, or fungal infections.
 Neurological testing (e.g., 10-gram monofilament or vibration tests) to measure sensation loss you might not notice.
 Vascular checks (pulses, ankle-brachial index) for poor blood flow.
 Structural assessment for deformities (bunions, hammertoes, Charcot foot) that create pressure points.

Daily self-checks complement this by catching day-to-day problems early.

Practical daily foot-check routine (takes 1–2 minutes)
 Look and feel every day—use a mirror for the soles or ask someone to help. Check between toes, heels, and the balls of your feet.
 Watch for: cuts, sores, blisters, redness, swelling, warmth, calluses, cracks, or changes in nail color/shape.
 Wash feet daily in warm (not hot) water, dry thoroughly (especially between toes), and moisturize (avoid between toes).
 Never go barefoot indoors or out. Wear well-fitting shoes and moisture-wicking socks. Check inside shoes for pebbles or rough spots before putting them on.
 Trim toenails straight across (or have a podiatrist do it if you have poor vision or circulation).

Who needs this most?
This advice applies especially if you have:
 Diabetes (type 1 or 2)
 Prediabetes or long-standing high blood sugar
 Known neuropathy, poor circulation, or history of foot ulcers/amputation
 Other risk factors like smoking, kidney disease, or retinopathy

Even without diabetes, people with severe peripheral artery disease, advanced age, reduced mobility/vision, or certain neurological conditions may benefit from similar vigilance.

Bottom line and next steps
Small, unnoticed problems escalate quickly when you can’t feel them and circulation is impaired. Regular checks (daily self-inspection + annual professional exams) are one of the most effective ways to prevent serious complications—most diabetes-related amputations are preventable with good foot care.

This is not a substitute for personalized medical advice. Talk to your doctor or a podiatrist about your specific risk level, how often you need exams, and whether you should be screened for neuropathy or circulation issues. They can also help with blood sugar management, proper footwear prescriptions, and smoking cessation if relevant—all of which protect your feet long-term.

Your feet may feel fine today, but proactive checks help keep them that way tomorrow.

Qwen3.6-Max-Preview:

It's a very reasonable question. If your feet feel fine, it's natural to wonder why you'd need to check them regularly. The short answer is that many foot problems develop silently, and by the time you feel pain or notice visible changes, they're often more advanced, harder to treat, or already affecting your mobility.

Here's why "feeling fine" isn't always a reliable indicator of foot health:

Why Asymptomatic Feet Can Still Have Issues
1. Nerve changes can mask problems: Conditions like peripheral neuropathy (common in diabetes, but also from vitamin deficiencies, alcohol use, or aging) reduce sensation. You could have a small cut, blister, or pressure sore and literally not feel it.
2. Early-stage conditions are often painless: Fungal infections, mild circulation changes, early arthritis, tendon wear, or structural shifts (like flattening arches or developing bunions) typically don't hurt at first.
3. Feet reflect systemic health: Skin dryness, slow-healing spots, nail thickening/discoloration, or unusual swelling can be early signs of diabetes, thyroid issues, vascular disease, or nutritional deficiencies long before other symptoms appear.
4. Biomechanical changes creep in gradually: Subtle shifts in how you walk or distribute weight can strain your feet, ankles, knees, hips, or back. You may not feel it until overuse injuries or joint pain develop.

What Regular Foot Checks Actually Do
 Catch small issues before they become painful or serious
 Prevent complications like ulcers, infections, or permanent structural changes
 Identify footwear or gait problems early
 Help maintain long-term mobility, balance, and independence

Who Especially Benefits from Regular Checks?
While everyone can benefit, professional foot exams are strongly recommended if you:
 Have diabetes, prediabetes, or metabolic syndrome
 Have poor circulation, peripheral artery disease, or a history of blood clots
 Are over 65
 Have arthritis, neuropathy, or a history of foot/ankle injuries or surgeries
 Are highly active (runners, athletes, people who stand/walk all day for work)
 Frequently wear tight, unsupportive, or high-heeled shoes

What "Regular" Actually Looks Like
 Low-risk, healthy adults: Monthly self-checks + a quick foot review during your annual physical is usually sufficient.
 Higher-risk individuals: Typically need professional checks every 3–12 months, depending on your condition and your provider's recommendation.
 Self-check basics: Look and feel the tops, sides, soles, and between toes for cuts, blisters, swelling, color changes, nail issues, or new calluses. Use a mirror or phone camera for hard-to-see areas. Do it in good light, ideally after bathing.

Think of foot checks like dental cleanings or blood pressure screenings: they're preventive, not reactive. Feeling great now is exactly the right time to keep things that way.

Disclaimer: This is general health information, not a substitute for personalized medical advice. If you have underlying health conditions or specific concerns, ask your primary care provider or a podiatrist what check-up schedule is right for you.

Q4. How often should I have my feet checked if I have diabetes?

GPT-5.5 Thinking:

For most people with diabetes: have a comprehensive foot exam at least once a year, plus a basic foot check at routine diabetes visits. The CDC specifically advises asking for a basic foot check at every health care visit and getting a yearly comprehensive foot exam.

You may need checks more often, often every 3–6 months, if you have higher-risk features such as trouble managing blood sugar or blood pressure, numbness/neuropathy, poor circulation, foot deformities, prior foot ulcers, or prior amputation.

At home, it is also a good idea to check your feet every day for cuts, blisters, redness, swelling, sores, nail problems, or temperature/color changes. Diabetes can reduce feeling and blood flow, so small injuries can worsen without being painful.

Seek care promptly—same day or urgently—if you notice an open sore, spreading redness, drainage, black skin, sudden swelling/warmth, fever, or a wound that is not healing.

DeepSeek-V4:

For someone living with diabetes, foot checks happen on two important timelines: a thorough daily self-check at home and a professional clinical examination at intervals determined by your risk level. The overarching rule is that regular monitoring can catch problems early, when they are most treatable.

1. Daily Self-Checks (Every Single Day)
This is non-negotiable. Every day, you should carefully look at and feel your feet for any changes, even if they seem minor. Check for:
 Cuts, blisters, or sores
 Redness, swelling, or warmth
 Corns, calluses, or ingrown toenails
 Changes in skin color or dry, cracked skin
 Signs of fungal infection between the toes

If you have trouble seeing the bottoms of your feet, use a mirror or ask a family member for help. Any new wound, area of redness, or sign of infection that doesn’t begin to heal within a day warrants an immediate call to your healthcare provider—do not wait for a routine check.

2. Professional Foot Examinations
The frequency of a formal foot exam by a healthcare professional (such as your primary care doctor, endocrinologist, or podiatrist) depends on your risk category. This risk is determined by factors like sensation, circulation, foot structure, and your history of ulcers or amputation.

Annual Comprehensive Exam (At Least Once a Year)
If you have no loss of protective sensation (no peripheral neuropathy) and no evidence of peripheral artery disease (PAD), you are considered low risk. For a low-risk foot, an annual comprehensive foot exam is the standard recommendation. This exam includes testing sensation with a monofilament, checking pulses, and inspecting for structural issues.

Every 3 to 6 Months (Higher-Risk Feet)
You should be seen more frequently—typically every 3 to 6 months—if you fall into a higher-risk category, which includes one or more of the following:
 Loss of protective sensation (you cannot feel the monofilament in one or more sites)
 Peripheral artery disease
 Significant foot deformity (such as bunions, hammertoes, or Charcot foot)
 A previous foot ulcer
 A previous lower-limb amputation
 End-stage renal disease or other advanced diabetes complications

Your healthcare provider will determine your exact recall interval based on how many risk factors are present. Those with a history of an ulcer or amputation often need examinations even more frequently, sometimes every 1 to 2 months, to prevent recurrence.

At Every Healthcare Visit (No Matter the Reason)
Even if you are seeing your doctor for a non-foot issue, it is a best practice for them to ask about your feet and take your shoes and socks off for a quick visual inspection. Advocate for yourself: if you are living with diabetes and your doctor doesn’t routinely look at your feet, remind them.

3. When to Seek Immediate Care (Do Not Wait)
Between scheduled checks, contact your provider promptly—ideally within 24 hours—if you notice:
 A break in the skin that is not healing or looks deeper than the surface
 Any sign of infection: increasing redness, swelling, warmth, pus, or fever
 New or worsening pain in the feet or legs (or a significant change in pain patterns)
 A sudden change in the shape, color, or temperature of your foot

The exact schedule for your professional foot exams should be personalized with your diabetes care team. The numbers above are evidence-based guidelines, but your individual plan takes into account your entire health picture. The most crucial takeaway is the daily habit: a few minutes every day is the single most powerful tool you have to protect your feet.

Gemini 3.1 Pro:

If you have diabetes, proactive foot care is a critical part of managing your overall health. Because diabetes can cause nerve damage (neuropathy) and poor circulation, small injuries can sometimes go unnoticed and develop into severe complications.

Here is the recommended schedule for how often your feet should be checked:

1. Daily Self-Checks

You should physically inspect your feet every single day. If you have trouble seeing the bottoms of your feet, use a handheld mirror or ask a family member to help you. Look for:

Cuts, scratches, blisters, or sores.
 Redness, swelling, or unusual warmth.
 Changes in skin color.
 Ingrown toenails or fungal infections.
 Cracked or dry skin, especially around the heels.

2. Professional Clinical Exams

The frequency of professional exams depends heavily on your individual risk level, but standard medical guidelines recommend the following:

At Least Once a Year: You should have a comprehensive foot exam by a podiatrist or your primary care provider annually. During this visit, they will test your nerve sensitivity (often using a monofilament tool or a tuning fork), check your circulation by feeling the pulses in your feet, and evaluate your foot structure for any deformities.
 Every 3 to 6 Months: You will need more frequent, targeted monitoring if you have specific risk factors, such as:
 Existing peripheral neuropathy (numbness, tingling, or burning sensations).
 Poor circulation (peripheral artery disease).
 Foot deformities (such as bunions, hammertoes, or Charcot foot).
 A history of previous foot ulcers or amputations.
 Kidney disease, which can increase swelling and infection risks.

At Every Routine Doctor's Visit: It is an excellent habit to take your shoes and socks off at every regular healthcare appointment so your doctor can perform a quick visual inspection.

When to Seek Immediate Care

Never wait for your next scheduled appointment if you notice any alarming changes. Contact your healthcare provider or podiatrist right away if you experience:

A cut, blister, or sore that does not begin to heal after a few days.
 Signs of infection, such as skin that is red, hot to the touch, swollen, or oozing.
 New or rapidly worsening numbness, tingling, or sharp pain.
 A callus with dried blood inside it, which can be an early sign of a wound underneath.

Grok 4.3:

For people with diabetes, professional foot checks are essential to prevent serious complications like ulcers, infections, and amputations, which stem from neuropathy (nerve damage), poor circulation, and slow healing. Guidelines from the American Diabetes Association (ADA) and CDC provide clear recommendations.

Daily Self-Checks (Everyone Should Do This)
Inspect your feet every day, even if they feel fine. Look for cuts, redness, swelling, sores, blisters, corns, calluses, cracks, or changes in skin/nails. Use a mirror for the soles or ask for help if needed. Report any issues to your doctor immediately—do not treat them yourself.

Professional Comprehensive Foot Exams
At least once a year for all people with diabetes (ADA recommendation). This should include:
 Skin and nail inspection
 Assessment of foot structure/deformities
 Neurological testing (e.g., 10-g monofilament for sensation, vibration, pinprick, or temperature)
 Vascular check (pulses in feet/legs)
 Risk factor review (history of ulcers, amputation, smoking, etc.)

More frequently if you are higher risk:
 Every visit with your healthcare provider (basic foot inspection) if you have sensory loss (neuropathy), prior ulcers/amputation, or other high-risk features.
 Every 3–6 months (or more often) for comprehensive exams if you have difficulty controlling blood sugar or blood pressure, neuropathy, poor circulation (peripheral artery disease), foot deformities, or a history of foot problems.

Risk-based schedule examples (adapted from ADA-aligned classifications):
 Very low risk (no loss of protective sensation or peripheral artery disease): At least annually.
 Low/moderate risk: Every 2–6 months.
 High risk (prior ulcer/amputation, Charcot foot, etc.): Every 1–3 months, often with podiatry referral.

Many people benefit from seeing a podiatrist (foot doctor) for the annual (or more frequent) comprehensive exam, especially if you have nerve damage. Your primary care provider or diabetes care team can perform basic checks at routine visits and refer you as needed.

Key ADA Standards of Care (2026)
 Perform a comprehensive foot evaluation at least annually (Grade A recommendation).
 More frequent exams for higher-risk individuals.
 Individuals with sensory loss or prior ulceration/amputation should have feet inspected at every visit.

Additional Tips
 Wear well-fitting shoes and socks; never go barefoot.
 Keep blood flowing (wiggle toes, elevate feet when sitting).
 Maintain good overall diabetes control (A1C, blood pressure, cholesterol) and quit smoking.
 Get referred to diabetes self-management education if needed.

This is general guidance based on ADA and CDC recommendations—it is not personalized medical advice. Your diabetes care team (endocrinologist, primary care provider, or podiatrist) will assess your specific risk factors (e.g., via monofilament testing or vascular studies) and create a tailored plan. Schedule a foot check soon if you haven’t had one recently, and contact your provider right away for any new foot concerns. Early detection saves limbs and lives.

Qwen3.6-Max-Preview:

If you have diabetes, current medical guidelines recommend the following foot check schedule:

Professional Foot Exams:
 At least once a year for all people with diabetes, as part of your routine diabetes care.
 Every 3–6 months (or at every visit) if you have any high-risk factors, such as:
 Peripheral neuropathy (nerve damage)
 Poor circulation (peripheral artery disease)
 Foot deformities (e.g., bunions, hammertoes)
 History of foot ulcers, infections, or amputations
 Diabetic kidney disease or vision impairment
 Inability to properly inspect your own feet

Self-Checks:
 Daily: Examine both feet (including between toes and soles) for cuts, blisters, redness, swelling, calluses, ingrown toenails, or changes in skin color/temperature. Use a mirror or ask for help if needed.

What a professional exam typically includes:
 Testing for sensation (often with a 10g monofilament or tuning fork)
 Checking pulses and blood flow
 Inspecting skin, nails, and foot structure
 Evaluating your footwear and gait
 Discussing foot care habits and risk reduction

When to seek immediate care:
Contact your healthcare provider or podiatrist right away if you notice:
 Any open sore, ulcer, or wound (even if painless)
 Signs of infection (redness, warmth, swelling, pus, fever)
 Sudden pain, numbness, or color/temperature changes
 A foot injury, even if it seems minor

Daily foot care tips:
 Wash and dry feet daily (moisturize, but not between toes)
 Never walk barefoot
 Wear well-fitting, supportive shoes and clean socks
 Trim nails straight across; avoid cutting calluses yourself
 Keep blood sugar, blood pressure, and cholesterol well-managed

Note: Guidelines from the American Diabetes Association (ADA), CDC, and NHS all align on annual professional exams for low-risk patients and more frequent checks for those with complications. Your care team may adjust this based on your individual health profile.

Disclaimer: This information is educational and not a substitute for personalized medical advice. Always follow the recommendations of your doctor or diabetes care team.

Q5. What does a diabetic foot ulcer look like?

GPT-5.5 Thinking:

A diabetic foot ulcer usually looks like an open sore or wound on the foot, most often on the bottom of the foot, toes, heel, or areas that rub against shoes. It may start small and look like a blister, callus, cracked skin, or a small red/raw spot, then become a deeper open wound. Ulcers can be painless because diabetes-related nerve damage can reduce feeling in the feet.

Common appearances include:

A round or punched-out sore with broken skin and exposed tissue underneath.
 A wound surrounded by thick callus or hard skin, especially on the sole.
 Red, pink, yellow, gray, brown, or black tissue in or around the wound.
 Drainage on the sock or dressing, sometimes clear, bloody, yellow, or pus-like.
 Swelling, warmth, redness, tenderness, bad smell, or pus, which can suggest infection.
 Black skin or tissue, which can be a sign of poor blood flow or gangrene and needs urgent medical attention.

A key point: in someone with diabetes, even a small sore, blister, cut, corn, ingrown toenail, or cracked area that is not healing can be serious. CDC and ADA both recommend checking feet daily and contacting a clinician promptly for ulcers, cuts that do not heal, numbness, or signs of injury.

Get medical care right away if there is spreading redness, warmth, swelling, pus, a bad odor, fever, increasing pain, black tissue, or if the person has diabetes and any open foot wound. Foot ulcers need prompt treatment to reduce the risk of deep infection, bone involvement, or amputation.

DeepSeek-V4:

A diabetic foot ulcer doesn’t have one single, uniform appearance—it can vary depending on its depth, location, and whether there’s infection or dead tissue present. However, most follow a recognizable pattern.

The Typical Appearance
At its simplest, a diabetic foot ulcer looks like a punched-out crater or open sore in the skin. You are essentially looking at a hole where tissue has been lost.

The ulcer itself:
 Size and Shape: They often start small, perhaps the size of a pencil eraser, but can enlarge. The shape is frequently circular or oval, described as having a “punched-out” look with well-defined, even edges, as if a small core of tissue was removed.
 Depth: This is highly variable. A superficial ulcer may look like a red, raw abrasion where only the top layer of skin is gone. A deeper ulcer is a true crater, exposing the yellowish, fatty subcutaneous tissue underneath. In severe cases, you might see deeper structures like glistening tendons, bone, or joint capsules.
 Color of the Base: The base (the floor of the ulcer) can be several colors, each telling a story:
 Pink or Red (Granulation Tissue): This looks like fresh, moist, slightly bumpy "raw meat" and is a sign of healthy healing.
 Yellow or Tan (Slough): This is a stringy, adherent layer of dead cells and debris. It can be thin and moist or thick and leathery, and must be removed for healing.
 Black or Dark Brown (Eschar): This is hard, dry, dead tissue, similar to a thick scab. It signals a severe lack of blood flow. It can be firmly attached and may hide the true depth of the wound underneath.

The surrounding skin:
The skin around the ulcer is almost never normal and provides crucial clues.
 Callus (Hyperkeratosis): This is a hallmark sign. The ulcer is very often surrounded by or was preceded by a thick, hard rim of yellowish, build-up skin. The ulcer sits in the center, like a hole in a corn or callus.
 Redness (Erythema): A rim of redness around the wound can be normal inflammation, but if it spreads, feels warm, and is tender, it points to an infection.
 Dry, Cracked Skin: The foot often looks dry, with flaking or fissures, due to autonomic neuropathy that stops the skin from sweating normally.

Location, Location, Location
Where the ulcer is on the foot is one of the biggest clues it’s neuropathic (caused by diabetes) rather than from another cause.
 Pressure Points: They almost exclusively occur in areas of high pressure.
 Plantar (Sole) Surface: The most common sites are under the ball of the foot, beneath the big toe joint, or on the tip or top of a hammertoe.
 Bony Prominences: They can also form on the sides of the foot (especially the outer edge) or the heel.

Key Signs It's a Diabetic Ulcer (What Makes It Different)
 Pain Is an Unreliable Guide: The most deceptive feature is that a deep, severe ulcer can be completely painless. The patient may not feel it at all because of neuropathy. While they are looking at a gaping hole, they might only complain of noticing a stain on their sock. Conversely, if blood supply is good and neuropathy is less severe, it can be very painful.
 The Unseen Depth: Often, the wound you see on the surface is just the “tip of the iceberg.” A small skin opening can cover a much larger, deep, undermined pocket of destroyed tissue underneath, which can be probed with a sterile instrument to reveal its true extent.
 Drainage: There is often drainage (exudate). A healthy healing ulcer has minimal, clear or slightly blood-tinged fluid. An infected ulcer will produce drainage that is cloudy, yellow, green, or foul-smelling.

Warning Signs of a Worsening Ulcer
A healing ulcer looks clean, pink, and is getting smaller. An ulcer that is getting worse or is infected will show:
 Spreading redness, warmth, and swelling around the wound.
 A change in the base from healthy red to a pale, yellow, or black color.
 Increased, pus-like, or bad-smelling drainage.
 The presence of a deep, dull ache or throbbing pain (even if the wound itself is numb), or signs of systemic illness like fever and chills.

If you suspect a diabetic foot ulcer, it is a medical emergency and requires immediate professional care to prevent infection, hospitalization, or amputation.

Gemini 3.1 Pro:

A diabetic foot ulcer is an open sore or wound that typically develops on the foot of a person with diabetes, usually resulting from poor circulation, neuropathy (nerve damage), and trauma to the foot. Because neuropathy reduces the ability to feel pain, a small blister or cut can easily go unnoticed and progress into a severe ulcer.

Here is a breakdown of what a diabetic foot ulcer typically looks like, depending on its severity:

Early Warning Signs

Before a full ulcer forms, the skin may show early signs of damage.

Thickened Skin: A prominent, thick callus often forms before the skin breaks down.
 Skin Discoloration: The area may look unusually red, brown, or black, indicating poor blood flow or pooling of blood under the skin.
 Swelling and Warmth: The affected area might be swollen or feel warmer than the rest of the foot, which can be an early sign of inflammation or infection.
 Blisters: A persistent fluid-filled blister that does not heal normally.

The Appearance of an Open Ulcer

Once the skin breaks, the ulcer can vary in appearance based on its depth and whether an infection is present.

Shape and Borders: They often look like a crater or a "punched-out" hole in the skin. The edges of the wound are usually raised and surrounded by a thick, calloused border.
 Wound Bed (Inside the Ulcer):
 Red or Pink: This usually indicates healthy granulation tissue, meaning the body is trying to heal the wound.
 Yellow or Gray: This is often "slough," which is a mix of dead white blood cells, bacteria, and dead tissue.
 Black or Dark Brown: This indicates "eschar" or necrotic (dead) tissue. It acts like a hard, dry scab over the wound and often needs to be removed by a medical professional for the wound to heal.

Depth: Ulcers can range from a superficial break in the top layers of the skin to a deep crater that exposes underlying fat, tendons, muscle, or even bone.
 Drainage: The sore may ooze clear fluid, blood, or thick, cloudy, yellowish-green pus.
 Odor: If an infection is present, the wound or drainage will often have a strong, foul smell.

Common Locations

Diabetic foot ulcers most frequently appear on areas of the foot that bear the most weight or experience the most friction, including:

The balls of the feet (underneath the metatarsal heads)
 The bottom or tips of the toes, especially the big toe
 The heels
 The sides of the foot (often due to poorly fitting shoes)

Important Medical Note: Diabetic foot ulcers are a serious medical condition. Because diabetes impairs the body's ability to heal and fight infection, even a small, seemingly minor sore can rapidly escalate into a severe infection that risks amputation. Anyone with diabetes who notices a sore, blister, or persistent callus on their foot should seek immediate evaluation from a healthcare provider or podiatrist.

Grok 4.3:

A diabetic foot ulcer is an open wound or sore on the foot that develops in people with diabetes, usually because of nerve damage (neuropathy), poor blood circulation, and repeated pressure or minor trauma that goes unnoticed.

Typical visual features
 Location: Most often on the bottom (plantar surface) of the foot—especially under the ball of the foot (metatarsal heads), the heel, or the big toe. They can also appear on the sides of the foot, between the toes, or on the tips of the toes.
 Shape: Usually round or oval with crisp, well-defined edges that look “punched out,” as if a cookie cutter removed a piece of skin.
 Size: Ranges from a few millimeters across (early stage) to several centimeters in more advanced cases.
 Depth: Varies widely. Superficial ulcers affect only the outer skin layers and look like a shallow scrape. Deeper ones extend into fat, tendon, or bone, creating a crater-like appearance where bone or tendon may be visible at the base.
 Wound bed (the center of the ulcer):
 Healthy granulating tissue is moist and bright red or pink, resembling raw meat.
 Yellow or white stringy material (slough) indicates dead tissue that needs removal.
 Black, dry, or leathery patches (eschar) represent necrotic (dead) tissue, common when blood flow is poor.
 Surrounding skin: Frequently thickened with a hard callus (hyperkeratosis) that forms a raised rim around the ulcer. The skin may appear red, shiny, or discolored; it can feel warm or cool depending on infection or circulation. Maceration (white, soggy skin) occurs if the area stays wet from drainage.
 Drainage: Clear or slightly yellow fluid is common. Infected ulcers produce thicker, cloudy, or greenish pus and may have a foul odor.
 Pain level: Many ulcers are surprisingly painless because of neuropathy, even when deep. Pain, tenderness, or throbbing usually signals infection or significant ischemia (poor blood flow).

Early vs. advanced appearance
Early ulcers often begin as a small red spot, blister, or area of skin breakdown hidden under a callus. They may look like nothing more than a “hot spot” or minor abrasion at first. Without prompt care they enlarge quickly, deepen, and develop the classic punched-out shape with callused borders.

Infected ulcers (very common complication)
Look for spreading redness beyond the wound edges, increasing swelling, warmth, pus, streaks of red up the foot or leg, and a bad smell. The ulcer may enlarge rapidly and the surrounding tissue can become boggy or black.

Ischemic (poor circulation) ulcers
These tend to occur on the toes, heels, or outer edges of the foot. The base is often pale or gray rather than red, with minimal granulation tissue and a dry appearance. The foot itself may feel cold, and pulses in the ankle or foot are weak or absent.

Diabetic foot ulcers are medical emergencies because they can progress to bone infection (osteomyelitis) or gangrene within days to weeks. Any open sore on the foot of a person with diabetes requires immediate professional evaluation by a podiatrist, wound-care specialist, or physician. Early recognition and treatment dramatically improve outcomes and help prevent amputation.

Qwen3.6-Max-Preview:

A diabetic foot ulcer is an open sore or wound that typically develops on the foot of a person with diabetes. Because diabetes can cause nerve damage (neuropathy) and reduced blood flow, even minor pressure or injury can break down the skin and heal poorly. Here's what these ulcers commonly look like, though appearance can vary by stage and individual:

Typical Visual Features
 Shape & Edges: Often round or oval with a "punched-out" appearance. Edges may be smooth, rolled, or surrounded by thickened, calloused skin.
 Wound Base:
 Pink/red: Indicates healthy granulation tissue
 Yellow/white: Slough (moist dead tissue)
 Black/brown: Eschar (dry, necrotic tissue)
 Depth: Can range from a shallow skin break to a deep crater that exposes tendons, joints, or bone.
 Drainage: May be clear, yellow, bloody, or pus-like. A foul odor often signals infection.
 Surrounding Skin: Frequently calloused. May appear red, swollen, warm, or discolored (pale, bluish, or shiny if circulation is poor).

Common Locations
Most often found on high-pressure or friction areas:
 Ball of the foot
 Heel
 Toes (especially tips or between toes)
 Sides of the foot or bony prominences

Important Clinical Notes
 Lack of pain is common: Due to neuropathy, many people don't feel the ulcer forming or worsening, which is why daily visual foot checks are critical.
 Signs of infection: Spreading redness, increased swelling, warmth, pus, foul smell, fever, or red streaks. Infection can progress rapidly in diabetes.
 Poor circulation signs: Cool skin, pale or bluish tone, thin/shiny skin, slow nail/hair growth, or wounds that don't improve over days to weeks.

When to Seek Care
Any break in the skin, non-healing sore, discoloration, or swelling on the foot of a person with diabetes should be evaluated promptly by a healthcare professional (primary care provider, podiatrist, or wound care specialist). Early treatment dramatically reduces the risk of serious complications, including deep infection or amputation.

Prevention tips: Daily foot inspections, well-fitting protective footwear, moisture control, nail care, and consistent blood sugar management are foundational.

Disclaimer: This information is for educational purposes and does not replace professional medical evaluation, diagnosis, or treatment. If you suspect a diabetic foot ulcer, please contact a healthcare provider promptly.

Q6. I have a small cut, blister, or sore on my foot. When should I see a doctor?

GPT-5.5 Thinking:

For a small cut, blister, or sore on your foot, you should see a doctor soon if any of these apply:

Get same-day medical care / urgent care if you have:

Spreading redness, warmth, swelling, worsening pain, pus/drainage, red streaks, bad odor, or fever — these can be signs of infection.
 A deep puncture wound, animal/human bite, wound with dirt/debris, burn, crush injury, or dead/black-looking tissue. These are higher-risk wounds for complications, including tetanus concerns.
 Diabetes, poor circulation, numbness/neuropathy, immune suppression, or a history of foot ulcers. Foot sores can become serious faster in these situations; early treatment lowers risk.

Make an appointment in the next day or two if:

It is not clearly improving after 2–3 days of basic care.
 It keeps reopening, is caused by shoe pressure, or you cannot avoid rubbing.
 A blister is large, very painful, or in a spot you have to walk on.
 The sore has been there more than about a week, even if it is not very painful.

Also check tetanus status: for a simple clean wound, medical sources generally use a 10-year booster window; for dirty/deep wounds, the interval is shorter, commonly 5 years. If you are unsure when your last tetanus shot was, contact a clinician or pharmacy.

For basic care: wash with clean running water, gently clean around it with soap, cover with a clean bandage, change it daily or when wet/dirty, and reduce pressure/friction from shoes. Do not pop a blister unless a clinician tells you to.

DeepSeek-V4:

It’s wise to be cautious with foot wounds, as they can sometimes become serious more easily than wounds on other parts of the body. While many small cuts, blisters, and sores heal fine with basic care, there are specific situations where you should definitely see a doctor.

Here’s a breakdown to help you decide:

See a doctor promptly (or go to urgent care) if:

1. You have a high-risk health condition
If you have any of the following, even a small foot wound warrants a medical evaluation because the risk of serious infection and non-healing is much higher:
 Diabetes (especially if you have neuropathy or poor circulation)
 Peripheral artery disease (poor blood flow to the legs/feet)
 Nerve damage (neuropathy) from any cause
 A weakened immune system (due to cancer treatment, HIV, organ transplant, long-term steroid use, etc.)

2. You notice signs of infection
Seek care if the area becomes:
 Increasingly red, warm, or swollen
 More painful instead of less
 Oozing yellow/green pus or a cloudy fluid
 Accompanied by red streaks spreading from the wound (this is an emergency)
 Associated with a fever, chills, or feeling generally unwell

3. The wound isn’t healing or is getting worse
If a cut or sore hasn’t shown clear signs of healing (scabbing, shrinking, new skin forming) within a few days, or if it’s enlarging, it needs a professional assessment.

4. It’s deeper than it looked
If a cut is gaping, you can see yellowish fat or deeper structures, it won’t close easily on its own, or it’s a puncture wound (especially from a nail or animal bite), see a doctor. Puncture wounds trap bacteria deep inside.

5. You can’t feel the area, or you have new numbness/tingling
This could indicate nerve involvement or an underlying condition that needs attention.

6. The blister is unusual
See a doctor if the blister:
 Is filled with blood or cloudy fluid
 Came from a burn, chemical exposure, or frostbite
 Keeps recurring in the same spot with no clear friction cause
 Is very large, extremely painful, or located on a weight-bearing area that makes walking impossible

7. There’s something stuck in it
If you can’t easily remove a splinter, piece of glass, or dirt with clean tweezers, let a professional clean it out. Leaving debris in a wound guarantees infection.

8. Your tetanus shot isn’t up to date
For a deep, dirty, or puncture wound, you'll typically need a tetanus booster if it’s been more than 5 years since your last shot. In the US, routine boosters are given every 10 years.

When home care is likely enough (for generally healthy people)

If none of the above apply, you can often manage it yourself:
 A small, clean cut that stops bleeding quickly
 A minor friction blister (the clear fluid kind) from a shoe, where the skin is intact
 A superficial scrape or sore that doesn’t get more painful

Basic care steps:
 Wash gently with mild soap and water; avoid harsh antiseptics like hydrogen peroxide that can delay healing.
 Apply a thin layer of petroleum jelly or antibiotic ointment to keep it moist.
 Cover with a clean bandage. For blisters, a special blister bandage (hydrocolloid) can cushion the area. Do not pop a blister unless it’s large, very painful, and you can do so with a sterile needle (leaving the roof of skin intact).
 Change the dressing daily, or whenever it gets wet or dirty.
 Relieve pressure on the area by wearing different shoes, thick padding, or moleskin.

If you're ever unsure, it's always safer to see a healthcare professional for an in-person look.

Disclaimer: This information is for educational purposes only and is not a substitute for professional medical advice. I am an AI, not a doctor. If you are concerned about your health, please consult a qualified healthcare provider.

Gemini 3.1 Pro:

While most small cuts, blisters, or sores on your foot will heal on their own with basic first aid, the feet are particularly vulnerable to infections and slow healing.

You should see a healthcare professional if you notice any of the following signs:

Signs of Infection (Seek Prompt Care)

Increased Redness or Swelling: Especially if the redness is expanding outward from the wound.
 Warmth: The area feels noticeably hotter to the touch than the rest of your foot.
 Drainage: You see pus, cloudy fluid, or yellowish/greenish discharge.
 Odor: There is a foul or unusual smell coming from the sore.
 Red Streaks: Red lines radiating from the wound up your foot or leg (a sign the infection may be spreading into the lymphatic system).
 Systemic Symptoms: You develop a fever, chills, or body aches.
 Increasing Pain: The pain gets worse over time rather than better, or it becomes severe.

Signs of Poor Healing

Lack of Progress: The wound hasn't noticeably started to heal or close after a week or two.
 Depth: The sore appears deep, reaching down to the muscle or bone, or you can't easily see the bottom of it.
 Recurrence: The blister or sore heals but keeps coming back in the exact same spot.

High-Risk Conditions (Always See a Doctor)

You should see a doctor for any foot wound, no matter how small it seems, if you have:

Diabetes: High blood sugar can impair healing and cause neuropathy (nerve damage), meaning you might not feel an infection getting worse.
 Neuropathy: Any condition that causes numbness or reduced sensation in your feet.
 Poor Circulation: Conditions like peripheral artery disease (PAD) restrict blood flow, making it much harder for your body to heal wounds and fight off infections.
 A Compromised Immune System: Due to conditions or medications that lower your body's ability to fight infection.

In the meantime: Keep the area clean by washing it gently with mild soap and water (avoid soaking it if it's an open sore), apply an over-the-counter antibiotic ointment if appropriate, and keep it covered with a clean, dry bandage. Avoid putting pressure on the wound if possible.

Grok 4.3:

I'm not a doctor, and this is general information only—not personalized medical advice. Foot wounds can escalate quickly due to moisture, pressure from shoes, and bacteria exposure. Always consult a healthcare professional (primary care doctor, urgent care, or podiatrist) for your specific situation, especially if you're unsure. If symptoms worsen rapidly, seek care immediately.

Home Care for Minor Cuts, Blisters, or Sores
Most small, clean wounds heal in a few days to a week or two with proper care:
 Wash your hands, then gently clean the area with mild soap and lukewarm water. Pat dry (don't rub).
 Apply a thin layer of over-the-counter antibiotic ointment (e.g., bacitracin or similar).
 Cover with a sterile bandage or non-stick pad. Change daily or sooner if wet, dirty, or loose.
 For blisters: Leave intact if possible to protect the skin underneath. Cushion with moleskin or padding. If it pops on its own or needs draining (only with a sterilized needle), clean thoroughly afterward and keep covered.
 Rest the foot, elevate it when sitting, and wear loose, comfortable shoes or open-toed footwear to reduce pressure.
 Monitor the wound daily for changes.

When to See a Doctor (General Guidelines)
Contact a doctor, urgent care, or podiatrist if:
 Signs of infection appear (common in foot wounds): Increasing redness, swelling, warmth, or tenderness around the area; pus, cloudy/yellow/green/foul-smelling drainage; red streaks spreading from the wound; fever over 100°F (37.8°C) or chills.
 The wound doesn't improve or starts healing within 5–10 days (some sources note 7–14 days or up to 2–3 weeks for full resolution). Non-healing raises risks like deeper infection or bone involvement.
 Pain becomes severe, worsens, or prevents walking/putting weight on the foot.
 The cut is deep, from a dirty/puncture object, animal bite, or won't stop bleeding with pressure (consider tetanus booster if your last shot was over 5–10 years ago).
 A blister becomes large, blood-filled, increasingly painful, red/swollen, or develops discharge.
 Swelling doesn't improve after 2–5 days of home care.

Go to urgent care or the ER right away for open wounds with pus, inability to walk, spreading infection signs, or high fever.

Special Considerations: Diabetes, Poor Circulation, Neuropathy, or Weakened Immunity
See a doctor or podiatrist the same day (or go to urgent care) for any cut, blister, or sore on your foot. These conditions impair healing and sensation, so even tiny issues can rapidly become ulcers, severe infections, or (in worst cases) lead to complications like amputation.

Watch especially for: any redness, warmth, swelling, drainage/odor, skin color changes (darker, paler, or unusual tones), or non-healing areas. Daily foot inspections are essential if you have diabetes.

Additional Tips
 Keep the area dry and clean; avoid soaking unless advised.
 Address the cause (e.g., ill-fitting shoes causing blisters) to prevent recurrence.
 Over-the-counter pain relief (like ibuprofen) can help with discomfort and swelling, but follow label instructions and check with a pharmacist if you have other health conditions.

Bottom line: Small foot issues are often manageable at home, but feet heal slower than other body parts and infection risk is higher. When in doubt—especially with diabetes or if infection signs appear—get it checked promptly. A podiatrist specializes in foot wounds and can provide advanced care like debridement or offloading if needed. Stay safe and heal well! If you provide more details (e.g., size, duration, your health conditions, or photos if relevant), I can help refine general suggestions, but professional evaluation is key.

Qwen3.6-Max-Preview:

While many small foot wounds heal well with basic home care, the feet are prone to infection and slower healing due to pressure, friction, and circulation factors. Here's when to seek medical evaluation:

Seek urgent or emergency care if you notice:
 Rapidly spreading redness, swelling, warmth, or worsening pain
 Pus, foul-smelling drainage, or red streaks moving up your foot or leg
 Fever, chills, nausea, dizziness, or feeling generally unwell
 A deep wound, heavy bleeding that won't stop, or exposed bone/tendon/joint
 Numbness, loss of sensation, or inability to bear weight

Schedule a prompt visit (within 24–48 hours) if:
 The sore isn't clearly improving after 5–7 days, or is getting larger/deeper
 You have mild, localized signs of infection (slight redness, tenderness, minor swelling) that persist or slowly worsen
 A blister keeps reopening or is in a high-pressure area (e.g., heel, ball of foot)
 The wound was caused by a dirty, rusty, or puncturing object and your tetanus shot isn't up to date (within 5 years for dirty wounds, 10 for clean ones)
 You're getting recurrent blisters or sores without a clear cause

High-risk individuals: See a doctor within 24 hours for ANY new foot sore if you have:
Diabetes or prediabetes
 Peripheral artery disease or poor circulation
 Peripheral neuropathy (reduced foot sensation)
 A weakened immune system (e.g., from medications, HIV, chemotherapy, chronic steroid use)
 History of foot ulcers or amputations

In these cases, even minor wounds can quickly progress to serious infections or chronic ulcers. Early professional care significantly reduces complications.

Safe home monitoring (if low-risk and no warning signs):
 Gently clean with mild soap and water; pat dry
 Apply a thin layer of petroleum jelly or antibiotic ointment (if not allergic)
 Cover with a sterile, non-stick bandage; change daily or when wet/dirty
 Avoid popping blisters or picking scabs
 Wear clean, well-fitting shoes and moisture-wicking socks
 Keep weight off the area when possible

When in doubt, get it checked. Foot wounds are deceptively prone to complications, and early evaluation is almost always safer than waiting.

Note: This is general educational guidance and not a substitute for professional medical advice. A licensed clinician can assess your specific situation, wound characteristics, and health history to recommend appropriate care.

Q7. Why can a small blister, callus, or cut become serious if I have diabetes?

GPT-5.5 Thinking:

A small blister, callus, or cut can become serious with diabetes because diabetes can reduce both feeling and healing.

The main reasons are:

1. You may not feel the injury. Diabetes can damage nerves, especially in the feet. That means a blister, cut, or pressure spot may not hurt, so you keep walking on it and it gets worse.

2. Blood flow may be reduced. Diabetes can affect circulation. Less blood flow means less oxygen, fewer immune cells, and slower delivery of the nutrients needed to heal.

3. Infection can develop more easily and spread faster. A tiny opening in the skin can let bacteria in. If healing is slow, the infection has more time to grow and can become an ulcer or deeper infection.

4. Calluses create pressure. A callus may seem harmless, but it often means there is repeated pressure or rubbing. Under the callus, skin can break down and form an ulcer, sometimes before you notice it. Calluses and corns are also listed as risk factors for serious diabetic foot complications.

5. Worst-case complications can be severe. If a wound becomes an ulcer and does not heal, it can lead to serious infection, gangrene, hospitalization, or amputation. This is why foot care is emphasized so strongly in diabetes care.

A good rule: check your feet every day for cuts, redness, swelling, blisters, sores, corns, or calluses. Do not try to cut off corns or calluses yourself. Contact a doctor or foot doctor promptly if you see a wound that is not healing, redness/warmth, swelling, drainage, increasing pain, black tissue, bad smell, or a callus with dried blood inside.

DeepSeek-V4:

That’s such an important question, and it gets to the heart of why foot care is emphasized so much in diabetes management. A small blister, callus, or cut that a person without diabetes might ignore can become a serious threat for someone with diabetes. It’s rarely one single cause, but rather a combination of factors that can quietly turn a tiny injury into a dangerous situation.

Here’s the breakdown of why that happens:

1. Nerve Damage (Neuropathy) – The "Silent" Problem
Chronically high blood sugar can damage nerves, especially in the feet and hands.
 Loss of Sensation: You might not even feel the blister forming from a tight shoe, a cut from stepping on something sharp, or the pressure of a thick callus. Since there’s no pain alarm, you continue walking on it, causing the injury to worsen without your knowledge.
 Motor Nerve Damage: The nerves controlling foot muscles can malfunction, causing the foot to change shape (like developing hammertoes) and creating new pressure points that are prime spots for calluses and ulcers.
 Autonomic Nerve Damage: The nerves that control sweating can stop working, leading to dry, cracked skin. These cracks become open doors for bacteria.

2. Poor Circulation (Peripheral Artery Disease) – The "Slow Delivery" Problem
Diabetes accelerates the narrowing and hardening of blood vessels, particularly in the legs and feet.
 Impaired Healing: Blood carries oxygen, nutrients, and immune cells to the site of an injury. With poor circulation, the delivery of these healing factors is drastically reduced. A simple cut struggles to close.
 Weakened Infection Defense: Your blood also carries white blood cells and antibiotics you might take. If blood flow is restricted, the body’s infection-fighting soldiers can’t reach the battlefield effectively. An infection can spread unchecked.

3. Weakened Immune System – The "Weak Defense" Problem
High blood sugar doesn't just damage nerves and vessels; it directly impairs the immune system.
 White blood cells become sluggish and less effective at killing bacteria. This means a normally minor bacterial invasion can rapidly turn into a serious infection that the body struggles to contain.

4. The Perfect Storm: Sugar as Fuel
Bacteria and fungi thrive in high-sugar environments. The elevated glucose in your body tissues essentially provides a feast for any invading germs, allowing them to multiply rapidly and worsen the infection.

The Dangerous Chain of Events
Here’s how these factors combine in a classic, all-too-common scenario:

1. It Starts Unnoticed: You get a small callus on the bottom of your foot. Because of neuropathy, you feel no pain. You keep walking on it as if nothing’s wrong.
2. Hidden Breakdown: The constant pressure causes the tissue under the hard callus to die and break down, forming a pool of fluid or a bleed that you can’t see. The callus acts like a mask.
3. The Wound Opens: The callus eventually comes loose or cracks open, revealing a deeper, open wound—a diabetic foot ulcer.
4. Infection Takes Hold: Bacteria easily enter the open ulcer. Because of poor circulation and a weakened immune system, the body can’t fight it off.
5. The Infection Spreads: The infection can spread from the skin to the soft tissue, and even down to the bone (a condition called osteomyelitis). This is incredibly difficult to treat and often requires intravenous antibiotics.
6. The Critical Endpoint: If the infection destroys too much tissue or the circulation is too poor for healing, the tissue dies (gangrene). To save your life and prevent the spread of a systemic infection (sepsis), amputation of the toe, foot, or leg may become medically necessary.

It’s a terrifying progression, but here’s the crucial counterpoint: this chain of events is almost entirely preventable. The fact that you’re asking this question shows you’re on the right track. The focus shifts to controlling the factors you can influence:
 Daily Foot Checks: Inspecting your feet every single day for anything out of the ordinary—a red spot, a blister, a crack, a change in shape—is your most powerful defense.
 Protective Footwear: Never walk barefoot, even at home. Well-fitting shoes and specialized diabetic socks reduce friction and pressure.
 Professional Callus Care: Never cut off a callus with a razor or use over-the-counter chemical removers. A podiatrist should manage this safely.
 Blood Sugar Management: Keeping blood glucose levels within your target range protects your nerves, blood vessels, and immune cells.
 Smoking Cessation: Smoking causes extreme vasoconstriction, making poor circulation catastrophically worse.

If you have diabetes and notice any new injury, no matter how small, show it to your doctor or podiatrist immediately. When it comes to diabetic feet, a cautious approach is always the right one.

Gemini 3.1 Pro:

A small skin lesion in the context of diabetes can quickly escalate into a severe clinical event, such as a deep tissue ulceration or osteomyelitis. This progression is primarily driven by a triad of systemic physiological complications: peripheral neuropathy, vascular insufficiency, and altered immune function.

1. Diabetic Neuropathy (Loss of Protective Sensation)

Chronic hyperglycemia drives metabolic and neurovascular changes that damage peripheral nerves, disrupting the body's primary warning systems.

Sensory Neuropathy: The loss of protective sensation (LOPS) means minor trauma—like a blister from tight shoes, a small cut, or thermal injury—often goes completely unnoticed.
 Motor Neuropathy: Atrophy of intrinsic foot muscles leads to anatomical deformities (e.g., hammertoes, pes cavus), shifting weight-bearing dynamics. This creates abnormal high-pressure zones where heavy calluses form. Repeated stress on these calluses frequently causes ischemic necrosis and sub-callus hemorrhaging, breaking down the tissue from the inside out.
 Autonomic Neuropathy: Damage to autonomic nerves causes anhidrosis (decreased sweating) and altered thermoregulation. The resulting dry, inelastic skin is highly susceptible to fissuring, instantly creating an entry point for opportunistic pathogens.

2. Microvascular and Macrovascular Compromise

The healing of any wound requires robust perfusion to deliver oxygen, fibroblasts, and immune cells. Diabetes profoundly impairs this through vascular deterioration—sharing the exact same underlying endothelial dysfunction, advanced glycation end-product (AGE) accumulation, and basement membrane thickening seen in other classic microvascular complications, such as diabetic retinopathy.

Peripheral Arterial Disease (PAD): Accelerated macrovascular atherosclerosis severely restricts arterial blood flow to the lower extremities.
 Microcirculatory Dysfunction: Capillary impairment prevents adequate local tissue perfusion. This halts the proliferative and remodeling phases of normal wound healing, leaving the tissue locked in a state of chronic inflammation, ischemia, and eventual necrosis.

3. Immune System Impairment

A persistent hyperglycemic environment directly blunts the host's cellular immune response.

Leukocyte Dysfunction: High blood glucose levels impair the chemotaxis, phagocytosis, and intracellular bactericidal activity of neutrophils and macrophages.
 Pathogen Proliferation: Because the initial lesion takes much longer to heal due to vascular ischemia, and the immune response is simultaneously sluggish, bacteria can easily colonize the open wound. Furthermore, high tissue glucose levels provide an ideal substrate for bacterial and fungal growth.

The Clinical Cascade

When these three factors combine, the standard physiological response to trauma completely fails. A small, unnoticed blister continues to be subjected to repetitive mechanical stress because the patient cannot feel it. Vascular insufficiency prevents the structural repair of the damaged tissue, while the impaired immune system allows localized bacteria to rapidly proliferate. This compounding cascade is why a minor epidermal injury can quickly bypass localized cellulitis and progress to limb-threatening deep tissue infections.

Grok 4.3:

In diabetes, even a tiny blister, callus, or cut can quickly turn serious—often escalating to infection, non-healing ulcers, or (in severe cases) amputation—because of three key complications that impair the body’s natural defenses and healing processes.

These issues most commonly affect the feet but can occur on hands or other areas too. Here’s why they happen and how a minor problem progresses.

1. Nerve Damage (Diabetic Neuropathy) — Loss of Protective Sensation
High blood sugar over time damages nerves, especially in the legs and feet (affecting up to about half of people with diabetes). This often causes numbness, tingling, or complete loss of feeling (called loss of protective sensation).

You may not feel pain, pressure, heat, or friction from a new blister (e.g., from shoes), a small cut, or the pressure that builds a callus. Injuries go unnoticed and untreated for days or weeks, allowing them to worsen. A blister might break open unnoticed, a cut might deepen, or a callus might crack—creating an open wound without you realizing it.

2. Poor Circulation (Peripheral Arterial Disease)
Diabetes damages and narrows blood vessels, reducing blood flow to the feet and lower legs. This deprives tissues of oxygen and nutrients needed for repair. Wounds heal much more slowly (or not at all), and immune cells struggle to reach the site to fight bacteria. A small opening that would heal quickly in someone without diabetes can linger, deepen, and become infected.

3. Weakened Immune Response and Higher Infection Risk
Elevated blood sugar impairs white blood cells’ ability to fight infection. Bacteria (or fungi) entering through even a tiny break in the skin multiply rapidly. What starts as a minor issue can progress to:
 Cellulitis (spreading skin infection)
 Osteomyelitis (bone infection)
 Gangrene (tissue death)

In extreme cases, this leads to hospitalization or amputation to stop life-threatening spread. Diabetes is the leading cause of non-traumatic lower-limb amputations, with roughly 15% of people with diabetes developing a foot ulcer at some point.

How a Small Problem Escalates (Typical Progression)
1. Minor injury (blister from friction, cut from trimming nails, or pressure sore under a callus) occurs.
2. Neuropathy hides the problem — no pain alerts you.
3. Poor healing + infection risk turns it into a non-healing ulcer.
4. Infection spreads deeper if untreated.
5. Severe complications (bone involvement, tissue death) may require surgery.

Calluses are especially tricky: they can hide ulcers underneath or crack open, acting as entry points for bacteria. Never try to cut or treat calluses/corns yourself if you have diabetes—see a podiatrist.

Prevention Is Highly Effective
Most serious foot problems are preventable with consistent habits:
 Check feet daily — Use a mirror or ask for help; look for redness, swelling, cuts, blisters, or callus changes.
 Control blood sugar — This slows or prevents nerve and vessel damage.
 Wear proper shoes and socks — Well-fitting, cushioned shoes; moisture-wicking socks; never go barefoot (even indoors). Check shoes for objects before wearing.
 Daily foot care — Wash in lukewarm water, dry thoroughly (especially between toes), moisturize tops and bottoms (not between toes). Trim nails straight across; have a professional do it if needed.
 See specialists regularly — Podiatrist at least yearly (more often with neuropathy); report any foot issue immediately.
 Avoid smoking and elevate feet when sitting to support circulation.

Early treatment of even minor issues dramatically lowers risk. If you notice any change, contact your healthcare team right away—do not wait.

This is general information based on sources like the American Diabetes Association and CDC. It is not personalized medical advice. Everyone’s diabetes is different; work closely with your doctor or diabetes care team (including a podiatrist) for tailored foot-care guidance and screenings. With good management, most people avoid these complications entirely.

Qwen3.6-Max-Preview:

When you have diabetes, even minor skin injuries like blisters, calluses, or small cuts can turn serious because diabetes affects several body systems that are essential for wound healing and infection prevention. Here's why:

Key Factors That Turn Minor Injuries Serious
1. Nerve Damage (Diabetic Neuropathy)
 Prolonged high blood sugar can damage peripheral nerves, especially in the feet and hands. This reduces sensation, so you may not feel a blister forming, a shoe rubbing, or a small cut. Without pain as a warning signal, injuries can go unnoticed, worsen, or become infected before you realize it.

2. Poor Blood Circulation
 Diabetes often damages blood vessels and reduces blood flow to the extremities. Good circulation is critical for delivering oxygen, nutrients, and immune cells to repair tissue. Reduced blood flow significantly slows healing and makes skin more prone to breakdown.

3. Impaired Immune Function
 High blood glucose interferes with white blood cell activity, weakening your body's ability to fight bacteria and fungi. Even a tiny break in the skin can become an entry point for infection that your immune system struggles to contain.

4. High-Sugar Tissue Environment
 Excess glucose in your blood and tissues creates a favorable environment for microbes to multiply. This not only raises infection risk but also further delays tissue repair.

How It Can Escalate
When these factors combine, a minor injury can quickly progress to:
 A diabetic foot ulcer (an open sore that won't heal)
 Deep tissue or bone infection (osteomyelitis)
 Gangrene (tissue death due to lack of blood flow and severe infection)
 In advanced cases, amputation may be necessary to stop spreading infection

What You Can Do
 Inspect daily: Check your feet (and hands) every day for redness, blisters, cuts, or changes. Use a mirror or ask for help if needed.
 Protect your skin: Wear well-fitting shoes, never walk barefoot, and avoid tight socks.
 Don't self-treat calluses or corns: See a podiatrist or healthcare provider. Cutting or using chemical removers can cause dangerous wounds.
 Keep skin clean & moisturized: Wash gently, dry thoroughly (especially between toes), and apply lotion to prevent cracking (but not between toes).
 Manage blood sugar: Consistent glucose control supports nerve health, circulation, and immune function.
 Act quickly: Contact your healthcare provider if a wound doesn't show improvement within 24–48 hours, or if you notice redness, swelling, warmth, drainage, foul odor, fever, or increasing pain.

Note: This information is educational and not a substitute for professional medical advice. If you have diabetes and develop any skin break, especially on your feet, prompt evaluation by a healthcare provider is strongly recommended.

Let me know if you'd like a printable foot-care checklist or guidance on what to ask your doctor about wound prevention.

Q8. What warning signs mean a diabetic foot wound needs urgent medical care?

GPT-5.5 Thinking:

A diabetic foot wound should be treated as urgent because infection and poor blood flow can worsen quickly, sometimes without much pain due to neuropathy. Foot ulcers need care right away, and a wound that will not heal can harm tissue or bone.

Seek urgent medical care today / within 24 hours if you notice any of these:

New blister, cut, open sore, ulcer, or wound on the foot, especially if you cannot feel it.
 Redness, warmth, swelling, inflammation, or a red/hot/swollen toe, foot, or ankle.
 Fluid, pus, blood, drainage, or a bad/new smell from the wound.
 New or worsening pain, throbbing, tenderness, or the foot feeling much hotter or colder than usual.
 Color changes, especially red, blue, grey, pale, black, or blotchy areas.
 A wound that is getting larger, deeper, not healing, or has visible dead/black tissue.
 Fever, chills, nausea, flu-like feeling, or feeling generally very unwell.

Diabetes UK specifically advises urgent foot-team care within 24 hours for color/temperature changes, new wounds, leaking pus/fluid, bad smell, or new unexplained foot pain. The IDSA/IWGDF guideline also says diabetes-related foot infection is diagnosed clinically from local or systemic signs of inflammation, and hospitalization should be considered for severe infections or moderate infections with important complicating conditions.

Go to the ER / emergency department now if there are signs infection may be spreading or sepsis is possible: confusion, slurred speech, trouble breathing, very high fever, very fast heartbeat at rest, blue/grey/pale/blotchy skin, rapidly spreading redness, red streaks up the foot or leg, severe pain, or the foot/toe looks black or gangrenous.

While getting help: stay off the foot, elevate it if you can, cover the wound with a clean sterile dressing, and do not soak it, cut skin/callus, pop blisters, or keep walking on it. Diabetes UK advises taking weight off the foot and contacting a GP/foot protection team straight away, or out-of-hours care if they are unavailable.

DeepSeek-V4:

For anyone with diabetes, a foot wound should never be ignored. Due to potential nerve damage (neuropathy) and reduced blood flow, even small injuries can rapidly turn into serious infections or non-healing ulcers. Recognizing the warning signs early can be limb-saving and, in severe cases, life-saving.

Here are the key warning signs that signal a diabetic foot wound needs urgent medical care:

1. Signs of a Spreading or Deep Infection
A surface infection can quickly move to deeper tissues and bone.
 Redness spreading away from the wound, especially streaks moving up your foot or leg.
 Increased warmth around the wound compared to the surrounding skin.
 Swelling that is new, worsening, or extends beyond the immediate wound area.
 Pus or foul-smelling drainage from the wound.
 New pain or throbbing in a foot that was previously numb or a wound that was painless.

2. Signs of Tissue Death or Gangrene
This indicates a critical lack of blood flow and requires immediate emergency evaluation.
 Black, dusky, or dark purple tissue around or beyond the wound.
 A dry, shrunken, mummified appearance of a toe or part of the foot.
 Bone or tendon visible at the base of the wound (this also indicates a deep, severe ulcer at high risk of bone infection).

3. Systemic Signs (Whole-Body Response)
If the infection has entered your bloodstream (sepsis), it’s a medical emergency. Go to the emergency department immediately if you experience:
 Fever (temperature over 100.4°F or 38°C) or chills.
 Nausea, vomiting, or confusion.
 Uncontrolled blood sugar levels that do not respond to your usual management.

4. Signs of Critical Limb Ischemia (Severe Poor Circulation)
A wound won’t heal without adequate blood flow. Seek urgent care if:
 Sudden, severe pain in the foot, especially at rest or at night, that may be relieved only by dangling the foot.
 The foot turns extremely pale, bluish, or feels icy cold compared to the other foot.
 A loss of previously palpable pulses in the foot (if you routinely check).

5. Rapid Deterioration or Non-Healing
 The wound is getting visibly larger or deeper day by day.
 A blister or callus suddenly fills with blood or breaks down, forming an open sore.
 No sign of healing after several days of proper at-home care (any wound that hasn’t started to shrink or close within 1-2 weeks warrants a professional evaluation).

What to do immediately:
 Stay off the foot entirely. Do not walk on a foot with an open wound, even if it doesn’t hurt.
 Cover the wound lightly with a clean, dry dressing.
 Contact your podiatrist, primary care doctor, or wound care center immediately to describe the signs. If you cannot reach them quickly or have systemic symptoms/gangrene, go straight to the nearest emergency department.
 Do not attempt to trim calluses, cut away dead tissue, or apply harsh antiseptics yourself.

Disclaimer: This information is for educational purposes only and does not constitute medical advice. Always consult a healthcare professional for any wound or medical concern.

Gemini 3.1 Pro:

Disclaimer: I am an AI, not a doctor. If you or someone you know is experiencing these symptoms, please seek immediate medical attention from a healthcare professional.

Diabetic foot wounds can deteriorate rapidly and lead to severe complications, including localized tissue death or systemic infection, if not treated promptly. You should seek urgent medical care if you notice any of the following warning signs:

1. Signs of Spreading Infection

Redness and Warmth: Redness that expands outward from the wound, red streaks traveling up the foot or leg, or the skin feeling unusually hot to the touch.
 Swelling: New or worsening swelling in the foot, ankle, or lower leg.
 Discharge: Pus that is yellow, green, cloudy, or thick draining from the wound.
 Foul Odor: A bad or unusual smell emanating from the wound, which is a strong indicator of a bacterial infection.

2. Signs of Tissue Damage or Poor Circulation

Discoloration: Tissue around or inside the wound turning black (which can indicate necrosis or gangrene), blue, or purple.
 Changes in Pain: A sudden or severe increase in pain. Conversely, because diabetic neuropathy can numb the feet, a complete lack of pain in a wound that looks severe is also a major red flag.

3. Concerning Wound Characteristics

Exposed Anatomy: You can see bone, tendons, or muscle at the base of the ulcer.
 Rapid Worsening: The wound is quickly becoming wider or deeper.
 Stagnation: The wound shows absolutely no signs of healing after a week or two of standard care.

4. Systemic (Whole-Body) Symptoms

Fever or Chills: Experiencing a high temperature, chills, or night sweats.
 Spiking Blood Sugar: Unexplained, persistently high blood glucose levels, which often happen when your body is fighting a severe infection.
 General Illness: Feeling nauseous, vomiting, or experiencing confusion/disorientation. These are critical signs that an infection may have entered the bloodstream (sepsis) and require an immediate trip to the emergency room.

Next Steps:
Do not attempt to treat these warning signs with over-the-counter ointments, foot soaks, or home remedies. Keep all weight off the affected foot to prevent driving the infection deeper, and contact your doctor, podiatrist, or go to the nearest emergency department immediately.

Grok 4.3:

Any foot wound in someone with diabetes requires prompt professional medical evaluation—ideally the same day or within 24 hours—because nerve damage (neuropathy) often hides pain and poor circulation impairs healing and infection fighting. Untreated issues can rapidly progress to deep infection, bone involvement (osteomyelitis), gangrene, or amputation. Up to 85% of diabetes-related amputations are preventable with early intervention.

This is not medical advice. The information below comes from reputable sources such as Alberta Health Services, MedlinePlus, the American Diabetes Association (ADA), and clinical guidelines. Always contact your healthcare provider, podiatrist, or go to urgent care/ER for personalized assessment. Do not attempt self-debridement or ignore wounds.

Immediate Emergency (Go to ER or Call Emergency Services)
Seek care right now for these red-flag signs, which may indicate severe infection, ischemia (poor blood flow), gangrene, or sepsis:

Foot suddenly becomes cold, painful, pale, bluish, or red and feels warm to the touch.
 Foot is red, hot, and swollen (with or without pain).
 Black, purple, brown, or greenish-black tissue (necrosis/gangrene), crackling under the skin when pressed, or a rapidly worsening foul odor.
 Spreading redness, red streaks up the leg, or rapidly expanding swelling.
 High fever (>100.4°F/38°C), chills, confusion, dizziness, vomiting, or flu-like symptoms.
 Inability to bear weight or walk due to severe pain/swelling.

Urgent Care Needed (Same Day or Within 24 Hours)
Contact your doctor, podiatrist, wound clinic, or ER (after hours) for these signs of local infection or wound deterioration:

Redness, increased warmth, or swelling around the wound (especially erythema >0.5 cm / ~¼ inch beyond the wound edge).
 Extra or new drainage, pus (yellow/green), or foul odor.
 Increased pain, tenderness, or firmness around the wound (or new pain if you previously had numbness).
 Wound that is not healing, getting larger, deeper, or developing black/white/blue tissue.
 Warm, swollen, red foot (even without an obvious open ulcer)—this can signal Charcot neuroarthropathy.
 Any new sore, blister, cut, or ulcer on a diabetic foot.

Clinical note: Infection is often diagnosed when at least two of the following are present: local swelling/induration, erythema >0.5 cm, local warmth, tenderness/pain, or purulent discharge. Moderately to severely infected ulcers, especially with comorbidities, often require hospitalization, IV antibiotics, and possible surgical intervention.

Additional Context and Prevention
 Daily foot checks are essential: Inspect the soles, between toes, and pressure points (heels, balls of feet) for redness, warmth, calluses, blisters, cracks, or drainage. Use a mirror or ask for help if needed.
 Control blood sugar tightly, as high levels impair healing.
 Never walk barefoot; wear well-fitting, supportive shoes and moisture-wicking socks. Avoid soaking feet or using harsh chemicals.
 See a foot care specialist regularly if you have neuropathy, prior ulcers, poor circulation, or foot deformities.

Early action dramatically improves outcomes. If you have a current diabetic foot wound or notice any of the above signs, do not wait—contact your healthcare team or seek emergency care immediately. Your podiatrist or multidisciplinary diabetic foot team is the best resource for ongoing management.

Qwen3.6-Max-Preview:

Diabetic foot wounds can deteriorate rapidly due to nerve damage, reduced blood flow, and impaired immune response. Because neuropathy often masks pain, visual and systemic signs are critical. Below are evidence-based warning signs that indicate urgent or emergency medical care is needed.

Go to the ER or call emergency services immediately if you notice:
 Systemic infection signs: Fever (≥100.4°F/38°C), chills, nausea, vomiting, confusion, dizziness, rapid heartbeat, or sudden worsening of blood sugar control
 Gangrene or tissue death: Black, gray, or leathery tissue; foul-smelling discharge; or a wound that looks "sunken" or dry/dead
 Critical poor circulation: Foot or toes that are cold, pale, blue, or mottled; sudden severe pain at rest; or loss of pulse in the foot
 Rapidly spreading infection: Red streaks moving up the foot/leg, or redness/swelling that expands noticeably over hours
 Deep structural involvement: Wound deep enough to see or probe bone, tendon, or joint; sudden foot deformity; or inability to bear weight

Seek same-day urgent care or contact your doctor within 24 hours if:
 Increasing warmth, swelling, redness, or pus/cloudy drainage around the wound
 Wound is getting larger, deeper, or hasn't started improving after 1–2 weeks of proper care
 New blister, cut, ingrown toenail, or callus with broken skin that hasn't been professionally evaluated
 Unexplained swelling in one foot/ankle, especially with tight or shiny skin
 You're unable to keep weight off the wound or don't have appropriate off-loading footwear

Important context:
 Pain is not a reliable indicator: Diabetic neuropathy can severely dull or eliminate pain, even with serious infection or tissue damage.
 Blood sugar spikes: Infections often cause unexplained hyperglycemia or increased insulin resistance. This can be an early warning sign.
 Time matters: Delays of even 24–48 hours in treating infected or ischemic diabetic foot wounds significantly increase risks of hospitalization, bone infection (osteomyelitis), or amputation.

While seeking care:
 Keep the wound clean and covered with a sterile, non-stick dressing
 Do not walk on it: Use crutches, a wheelchair, or strict off-loading
 Avoid hydrogen peroxide, iodine, or alcohol (they damage healing tissue)
 Do not attempt to cut away dead skin or drain pus yourself
 Keep your foot elevated if swollen, but avoid tight wraps

Disclaimer: This information is for educational purposes and does not replace professional medical advice. If you have diabetes and develop any foot wound, prompt evaluation by a healthcare provider (ideally within 24–48 hours) is strongly recommended. When in doubt, err on the side of caution and seek care immediately.

For reference, these guidelines align with recommendations from the American Diabetes Association (ADA), International Working Group on the Diabetic Foot (IWGDF), and CDC wound care protocols. Let me know if you'd like guidance on daily foot inspection or preventive care.

Q9. How can I tell if my diabetic foot wound is infected?

GPT-5.5 Thinking:

You can’t reliably rule infection in or out at home, but with diabetes you should treat new/worsening foot-wound changes as same-day medical issues, because pain and fever can be muted by neuropathy or poor circulation. IDSA/IWGDF guidance says diabetic foot infection is diagnosed mainly by clinical signs of local or systemic inflammation, not just by a wound culture.

Signs your diabetic foot wound may be infected

Look for two or more of these changes around the wound:

New or spreading redness or discoloration
 Warmth compared with the other foot
 Swelling, firmness, or puffiness
 New or worsening pain — or any new wound you don’t feel because of numbness
 Drainage, especially yellow/green fluid, pus, or increasing wetness
 Bad smell
 Wound is getting larger, deeper, darker, or not improving
 Skin around it is turning black, blistering, or breaking down

Diabetes UK specifically advises urgent care within 24 hours for a red/hot/swollen toe, ankle, or foot; a wound that smells bad or leaks fluid/pus; new wounds; color/shape change; or new unexplained pain.

Go to the ER now / call emergency services if you have any of these

Fever, chills, shaking, nausea/vomiting, or feeling very unwell
 Confusion, fast breathing, shortness of breath, very fast heartbeat
 Red streaks spreading up the foot or leg
 Foot turning pale, blue, gray, black, or suddenly much colder
 Rapidly increasing swelling, severe pain, new numbness, or inability to bear weight
 Pus under pressure, black tissue, or a deep wound where you can see tendon/bone

Systemic symptoms like fever or chills may be uncommon in diabetic foot infection, but when present they suggest a more severe, potentially limb- or life-threatening infection. NHS Inform also flags foot color change to very pale or blue, especially with new numbness or pain, as an emergency.

What to do right now

Take weight off the foot, elevate it, cover the wound with a clean dry dressing, and contact your podiatrist, wound clinic, diabetes team, or primary care clinician today. Don’t soak it, cut tissue/callus yourself, use leftover antibiotics, or rely on peroxide/iodine unless your clinician told you to.

If you’re unsure whether it’s infected, err on the side of being seen today. In diabetic foot wounds, early treatment can prevent hospitalization, surgery, or amputation.

DeepSeek-V4:

It’s crucial to catch an infection early when you have diabetes, because high blood sugar can slow healing, weaken the immune response, and reduce sensation, allowing a minor wound to become serious very quickly. Here’s what to watch for.

Signs Your Diabetic Foot Wound May Be Infected

Look for these local and systemic signs daily. Don’t rely on pain alone—neuropathy often masks it.

Redness spreading outward: The skin around the wound turns red and the redness expands or streaks away from the wound (red lines tracking up the foot or leg are a medical emergency).
 Increased warmth: The skin around the wound feels noticeably warmer than the surrounding skin or the same spot on the other foot.
 Swelling or hardness: New or worsening swelling, or the area feeling firm and tight.
 New or increasing pain, tenderness, or throbbing: Even if you usually have numbness, any new sensation of pain, burning, or aching around the wound is significant.
 Change in drainage: Pus or cloudy fluid seeping from the wound, or a major increase in the amount of drainage. Drainage that is yellow, green, or brown is especially concerning.
 Foul odor: A bad smell coming from the wound, even after cleaning.
 Delayed healing or wound deterioration: The wound is not getting smaller; it’s getting larger, deeper, or the edges look ragged, dark, or are turning black/brown (this can be gangrene).
 Discolored tissue inside the wound: Healthy wound tissue is pink or red. Pale, yellowish, gray, green, or black tissue suggests dead tissue or infection.
 Systemic signs: Fever, chills, night sweats, body aches, or unexplained fatigue and confusion. Feeling generally unwell with a foot wound is a red flag.
 High blood sugar: Unexplained spikes in blood glucose can be your body’s stress response to an infection.

Why This Is Urgent
Because diabetes can impair circulation and numb the feet (peripheral neuropathy), you may not feel the typical pain that signals trouble. A small blister or callus can quickly progress to a deep infection involving the bone (osteomyelitis) without you realizing it. Infections can also spread rapidly through the foot, leading to tissue death and the risk of amputation.

What You Should Do
 Stop putting weight on the foot: Off-load the wound completely. Use crutches, a wheelchair, or a postoperative shoe as directed by a provider—never walk barefoot on the wound.
 Keep the wound clean and covered: Use a sterile, non-stick dressing. Do not soak the foot unless specifically advised by your healthcare team.
 Do not apply home remedies: Avoid hydrogen peroxide, alcohol, betadine, or other harsh disinfectants unless instructed, as they can damage healing tissue.
 Seek immediate medical attention: Call your podiatrist, primary care provider, or wound care center right away—tell them you have diabetes and a possible foot infection. If you have red streaks, fever, rapidly spreading redness, or severe pain, go to the emergency department.

Even a single sign of infection means you should be evaluated today. Foot infections in people with diabetes can escalate within hours. Early, aggressive medical treatment (which may include antibiotics, wound debridement, or vascular assessment) is critical to prevent serious complications.

This information is for educational purposes and does not replace professional medical advice. If you suspect an infection, contact a healthcare provider immediately.

Gemini 3.1 Pro:

Disclaimer: I am an AI, not a doctor. Because you have diabetes, any foot wound requires careful attention. If you suspect an infection, it is crucial to have a healthcare professional evaluate it immediately, as diabetic foot infections can escalate quickly.

It is very important to monitor diabetic foot wounds closely. Because diabetes can cause nerve damage (neuropathy), you might not feel the pain that typically accompanies an infection, making visual and physical checks essential.

Here are the primary signs that a diabetic foot wound may be infected:

Localized Signs of Infection (At the Wound Site)

Redness: The skin around the wound is red, or you notice the redness spreading outward.
 Swelling and Hardness: The area around the wound feels swollen, puffy, or unusually hard to the touch.
 Warmth: The skin surrounding the ulcer feels warmer than the rest of your foot or your other foot.
 Discharge or Pus: The wound is oozing cloudy, yellow, green, or blood-tinged fluid.
 Foul Odor: A noticeable, unpleasant smell coming from the wound, even after cleaning it.
 New or Worsening Pain: If you still have sensation in your feet, you may feel increased pain, throbbing, or tenderness.
 Changes in Tissue Color: The tissue inside or around the wound turns black, blue, or severely pale (signs of tissue death or poor blood flow).

Systemic Signs of Infection (Affecting Your Whole Body)

If the infection begins to spread into deeper tissues, bones, or your bloodstream, you might experience systemic symptoms. Seek emergency medical care if you notice any of these:

Fever or chills
 Unexplained high blood sugar levels that are difficult to manage
 Nausea, vomiting, or feeling generally unwell
 Confusion or dizziness
 Rapid heart rate

What to Do Next

If you notice any of these signs, do not try to treat the infection at home with over-the-counter creams or by soaking the foot, as this can sometimes make it worse.

Contact your primary care physician, an endocrinologist, or a podiatrist right away. If you cannot reach them and are experiencing systemic symptoms or rapid worsening of the wound, go to an urgent care clinic or emergency room.

Grok 4.3:

This is not medical advice. I am an AI, not a doctor. Diabetic foot wounds can progress extremely quickly to serious infections, bone involvement (osteomyelitis), tissue death (gangrene), or even amputation because of nerve damage (neuropathy) that reduces pain sensation and poor circulation that impairs healing and immune response. Contact your doctor, podiatrist, or go to urgent care/ER immediately if you have diabetes and any foot wound, sore, or the signs below. Do not wait or rely on home remedies alone.

Key Signs Your Diabetic Foot Wound May Be Infected
Infection is diagnosed clinically when there are at least two of these local signs (per standard guidelines referenced by sources like the American Academy of Family Physicians and wound care experts):

Redness (erythema) spreading from the wound edges — especially more than about ½ inch (1–2 cm) or increasing daily.
 Swelling, puffiness, or firmness (induration) around the wound or foot.
 Warmth — the area feels noticeably hotter than the same spot on your other foot (use the back of your hand to compare).
 Pain or tenderness — new pain, increasing pain, or throbbing (note: many people with diabetic neuropathy feel little or no pain even with deep infection).
 Purulent (pus) or cloudy drainage — yellow, green, or thick discharge; extra drainage soaking socks or bandages.
 Foul or unusual odor from the wound.

Other warning signs of worsening or deeper infection:
 The wound is getting larger, deeper, or not improving after a few days of care.
 Black, white, blue, or dead-looking tissue (eschar or necrosis).
 Red streaks running up the foot or leg (lymphangitis — a medical emergency).
 Constant bleeding or heavy drainage.

Systemic (whole-body) signs — seek emergency care right away:
 Fever, chills, or feeling generally unwell/fatigued.
 Swollen lymph nodes in the groin or elsewhere.
 Nausea or flu-like symptoms.

Even without obvious pus or fever, any new or non-healing foot wound in someone with diabetes needs prompt professional evaluation because infections can spread silently under the skin or into bone within 24–48 hours.

What to Do Right Now
1. Inspect daily — Check feet (including soles and between toes) every day, ideally at night. Use a mirror or ask for help if vision or flexibility is limited. Note any drainage on socks or shoes.
2. Compare both feet — Look for asymmetry in color, temperature, swelling, or shape.
3. Contact a professional today if you notice any of the above signs or if a wound hasn’t started improving within a few days. Tell them you have diabetes — many clinics prioritize diabetic foot issues.
4. Go to the ER for fever, spreading redness/streaks, severe pain/swelling, or if you can’t reach your doctor quickly.

Why Early Detection Matters
Diabetic foot infections are a leading cause of hospitalization and lower-limb amputation. Early antibiotics, wound cleaning (debridement), pressure offloading (special boots or casts), blood-sugar control, and sometimes vascular assessment or imaging (X-ray, MRI) can save the limb. Your care team may also test for bone involvement if the wound is deep or not healing.

Prevention Basics (While You Seek Care)
 Keep blood sugar in target range — high levels slow healing.
 Wear well-fitting shoes (never barefoot, even indoors) and change socks daily.
 Moisturize dry skin but avoid between toes.
 See a podiatrist regularly for nail care, callus management, and foot exams (at least yearly, more often if you have neuropathy or prior ulcers).
 Never ignore a blister, cut, or “hot spot.”

Bottom line: When in doubt, get it checked — today. Many people lose toes or feet because they waited to see if it “got better on its own.” Your primary care doctor, endocrinologist, or a specialized diabetic foot clinic/wound center can guide next steps. If you have a current wound, describe the signs you’re seeing to your provider for faster triage. Take care of your feet — they carry you through life.

Qwen3.6-Max-Preview:

Disclaimer: I am an AI, not a doctor. Diabetic foot wounds can progress rapidly and lead to serious complications. Always have a new or changing foot wound evaluated by a healthcare professional familiar with diabetes care.

Because diabetes can affect nerve sensation, circulation, and immune response, foot infections sometimes present atypically or worsen quickly. Here are medically recognized signs that a diabetic foot wound may be infected:

Local Signs (Around the Wound)
 Spreading redness, swelling, or warmth that extends beyond the original wound edges
 Pus or abnormal drainage: cloudy, yellow, green, or bloody fluid
 Foul or unusual odor that doesn't improve with gentle cleaning
 Wound deterioration: getting larger, deeper, or failing to show any healing after 2–3 days of proper care
 Black, gray, or leathery tissue (necrosis/eschar) or sudden color changes
 Red streaks moving up the foot or ankle (suggests lymphatic spread)
 New or worsening pain or tenderness Note: Diabetic neuropathy often blunts or eliminates pain, so absence of pain does NOT rule out infection.

Systemic Signs (Infection Spreading)
 Fever (≥100.4°F / 38°C) or chills
 Unexplained spikes in blood sugar or sudden difficulty controlling glucose
 Fatigue, confusion, rapid heartbeat, nausea, or feeling generally unwell
 Swollen lymph nodes in the groin

Seek Urgent or Emergency Care If You Notice:
 Rapidly expanding redness, swelling, or warmth
 Fever, chills, dizziness, or signs of sepsis
 Foul-smelling pus, exposed bone/tendon, or black/dead tissue
 Inability to bear weight or walk
 Any new foot wound that hasn't been professionally assessed

What to Do Right Now
1. Keep it clean & covered: Gently rinse with saline or clean water, pat dry, and apply a sterile, non-stick dressing. Avoid hydrogen peroxide, iodine, or alcohol, which can damage healing tissue.
2. Offload pressure: Avoid walking on the affected foot. Use crutches, a boot, or wheelchair if available.
3. Monitor blood glucose: Infection often raises blood sugar; check more frequently and follow your diabetes management plan.
4. Contact a clinician promptly: A podiatrist, wound care specialist, or primary care provider should evaluate the wound. Diabetic foot infections typically require professional debridement, culture-guided antibiotics, and specialized dressings.

Why Diabetic Foot Wounds Need Extra Caution
 Neuropathy can mask pain, making visual and systemic clues critical
 Poor circulation may dull classic inflammatory signs (redness/warmth)
 Infections can spread to bone (osteomyelitis) or bloodstream quickly
 Early professional intervention dramatically reduces amputation risk

If you're unsure whether your wound is infected, err on the side of caution and seek medical evaluation within 24 hours. Diabetic foot care is highly time-sensitive, and prompt treatment makes a significant difference in outcomes.

Q10. Does every diabetic foot wound need antibiotics?

GPT-5.5 Thinking:

No. A diabetic foot wound does not need antibiotics unless it is clinically infected.

Current IWGDF/IDSA guidance says to diagnose infection clinically based on local or systemic signs of inflammation, and specifically: do not treat clinically uninfected foot ulcers with systemic or local antibiotics just to prevent infection or promote healing.

Signs that suggest infection include redness, warmth, swelling/induration, tenderness or pain, purulent drainage, or systemic illness; the IWGDF/IDSA classification uses “at least two” local signs such as swelling, erythema, warmth, pain/tenderness, or purulent discharge, after ruling out other causes of inflammation.

Antibiotics are indicated when there is a diabetic foot infection, and choice/duration should depend on infection severity, likely organisms, cultures when appropriate, kidney function, allergies, local resistance, and whether osteomyelitis is present. Mild and some moderate infections can often be treated orally; severe infections generally need IV therapy and urgent evaluation.

Important nuance: all chronic wounds are colonized with bacteria, so a positive superficial swab or odor alone does not necessarily mean infection. IDSA notes that all wounds are colonized, and infection should not be defined by culture results alone.

So the practical answer is: no antibiotics for a clean, noninfected diabetic foot ulcer; prompt antibiotics and often wound/podiatry evaluation when infection is present. Seek urgent care for spreading redness, fever, severe pain, black tissue/gangrene, a deep wound with exposed bone, rapidly worsening drainage, or signs of sepsis.

DeepSeek-V4:

No, not every diabetic foot wound requires antibiotics.
Antibiotics are only indicated when there is clinical evidence of infection. Many diabetic foot ulcers are uninfected and can heal with proper wound care alone.

When antibiotics are NOT needed (uninfected wound)
A diabetic foot ulcer is considered uninfected if it shows no signs of local inflammation or systemic involvement. This means:
 No purulent drainage (pus)
 No erythema (redness) extending beyond the wound edge
 No increased warmth, swelling, or tenderness beyond what is expected from the wound itself
 No systemic signs (fever, chills, leukocytosis)
 No malodor with advancing cellulitis

For these wounds, management focuses on:
 Sharp debridement and proper wound dressings
 Offloading (pressure relief)
 Vascular assessment if poor perfusion is suspected
 Tight glycemic control

Routine swabbing of an ulcer without clinical signs of infection is not recommended because it simply grows colonizing organisms—not pathogens causing infection—and may lead to unnecessary antibiotic use.

When antibiotics ARE needed (clinical infection)
Antibiotics should be prescribed when there is a diabetic foot infection (DFI). The International Working Group on the Diabetic Foot (IWGDF)/Infectious Diseases Society of America (IDSA) classifies infections as:

1. Mild infection (superficial, limited in extent)
 ≥2 signs of local inflammation (e.g., erythema >0.5 cm to ≤2 cm around ulcer, warmth, induration, tenderness, purulent discharge)
 No deeper tissue involvement or systemic signs

2. Moderate infection (deeper or more extensive)
 Erythema >2 cm around the ulcer, or involvement of structures below the skin (abscess, osteomyelitis, fasciitis, joint involvement)
 No systemic signs required for this category

3. Severe infection
 Local infection PLUS systemic toxicity (fever, hypotension, tachycardia, confusion, metabolic instability, leukocytosis)

Antibiotics are mandatory for all moderate and severe infections, and generally recommended for mild infections as well (usually oral, targeted at aerobic Gram-positive cocci initially). Severe infections require urgent surgical evaluation and intravenous broad-spectrum antibiotics.

Special considerations
 Osteomyelitis – suspected when an ulcer probes to bone or a visible bone fragment is present. Usually requires prolonged antibiotic therapy (often ≥6 weeks) +/- surgical resection.
 Peripheral arterial disease (PAD) – an ischemic, uninfected ulcer may not need antibiotics but does need urgent vascular assessment; revascularisation can be limb-saving.
 Moist gangrene or wet necrosis – usually treated with antibiotics because bacteria can thrive in dead tissue, but debridement/amputation is the definitive treatment.

Why unnecessary antibiotics are harmful
 Promote antimicrobial resistance
 Risk of C. difficile infection and other adverse effects
 Can lead to incorrect attribution of non-healing to “infection” rather than the real drivers (pressure, ischaemia, poor wound care)

Bottom line
Only clinically infected diabetic foot wounds warrant antibiotic therapy. Uninfected ulcers—no matter how chronic or large—are treated with debridement, offloading, moisture balance, and vascular care. If in doubt, reassess the wound in 24–48 hours and consult an expert; never start antibiotics “just in case” without signs of infection.

Gemini 3.1 Pro:

No, not every diabetic foot wound requires antibiotics. According to clinical guidelines, including those from the Infectious Diseases Society of America (IDSA) and the International Working Group on the Diabetic Foot (IWGDF), antibiotic therapy is indicated only for wounds that show clinical signs of infection.

When Antibiotics Are Indicated

A diabetic foot ulcer is clinically infected—and warrants antibiotic treatment—if there is purulent discharge or the presence of at least two of the following classic signs of local inflammation:

Erythema (redness extending beyond the wound margin)
 Warmth
 Swelling or induration
 Tenderness or pain

More severe infections may involve deeper structures (such as muscle, tendon, or bone, leading to osteomyelitis) or present with systemic signs of toxicity, such as fever, chills, tachycardia, or leukocytosis.

Why Antibiotics Are Avoided in Uninfected Wounds

Prescribing antibiotics for clinically uninfected foot ulcers as prophylaxis or to "speed up" healing is strongly discouraged for several reasons:

No Healing Benefit: Antibiotics do not promote tissue repair or accelerate wound closure in the absence of an active infection.
 Antimicrobial Resistance: Unnecessary use drives the development of multi-drug resistant organisms, making future infections much harder to treat.
 Adverse Effects: It exposes patients to drug toxicities, allergic reactions, and the risk of opportunistic infections like Clostridioides difficile.
 Microbiome Alteration: It can alter the local flora of the wound bed, potentially selecting for more virulent or resistant pathogens if an infection does eventually develop.

Standard Care for Uninfected Wounds

If a wound is not clinically infected, management focuses on creating the optimal environment for tissue repair. Standard care includes:

Off-loading: Relieving mechanical pressure from the ulcerated area (e.g., using specialized footwear or casts).
 Debridement: Removing necrotic, hyperkeratotic, or non-viable tissue to expose a healthy wound bed.
 Wound Care: Applying appropriate dressings to maintain a balanced, moist wound environment.
 Vascular Assessment: Checking for peripheral arterial disease (PAD) to ensure there is adequate perfusion for healing.
 Glycemic Control: Optimizing blood glucose levels to support immune function and cellular repair.

Grok 4.3:

No, not every diabetic foot wound (ulcer) needs antibiotics.

Antibiotics treat infection, not wounds themselves. Most diabetic foot ulcers are colonized by bacteria (all open wounds are), but colonization alone does not equal infection and does not require antibiotics. Routine or prophylactic antibiotic use for non-infected ulcers does not speed healing, prevent future infection, or improve outcomes—and it contributes to antibiotic resistance, side effects (like C. difficile colitis), and unnecessary costs.

How Infection Is Diagnosed
Diagnosis is clinical, not based on wound swabs or cultures alone. Key signs include at least two of:
 Local warmth
 Erythema (redness) extending >0.5 cm around the wound
 Tenderness or pain
 Swelling or induration
 Purulent drainage (pus)

Systemic signs (fever, chills, elevated white blood cell count) suggest more severe infection. Superficial swab cultures are unreliable due to contamination; deep tissue or bone cultures (obtained during debridement) guide therapy only when infection is present.

Current Guidelines (IWGDF/IDSA 2023 and Others)
The 2023 IWGDF/IDSA guidelines (joint International Working Group on the Diabetic Foot and Infectious Diseases Society of America) and supporting sources (AAFP, UpToDate, Johns Hopkins) state explicitly:

Do not treat clinically uninfected foot ulcers with systemic or topical antibiotics for healing or infection prevention.
 Even if imaging suggests osteomyelitis (bone infection), antibiotics are not automatically started without clinical signs of soft tissue infection. Confirm with bone biopsy when possible; management often involves surgical resection plus targeted antibiotics.
 For infected wounds, classify severity (mild/moderate/severe) and use the narrowest effective regimen for the shortest duration (typically 1–2 weeks for uncomplicated soft-tissue infections; longer for osteomyelitis or slow response, up to 3–4 weeks in some cases with severe peripheral artery disease).

Mild infections (limited cellulitis, no systemic signs) often respond to oral antibiotics covering gram-positive organisms (Staphylococcus aureus, streptococci), with MRSA coverage if risk factors exist.
Moderate/severe infections require broader coverage (including gram-negatives/anaerobes in many cases), often starting IV, plus urgent surgical evaluation.
Empiric choices should reflect local resistance patterns, patient factors (allergies, kidney function, prior cultures), and stewardship principles—de-escalate once cultures return.

Management of Non-Infected Diabetic Foot Wounds
Focus on these evidence-based steps instead:
 Offloading pressure (special footwear, total contact casting, crutches).
 Debridement of dead tissue (by a trained provider).
 Moist wound dressings and regular cleaning.
 Optimal glycemic control.
 Vascular assessment (many patients need revascularization).
 Multidisciplinary care (podiatry, endocrinology, infectious disease, vascular surgery, wound care specialists).

These measures address the root causes—neuropathy, poor perfusion, and high blood sugar—far more effectively than antibiotics in the absence of infection.

Important Caveats
 Diabetic foot complications are serious and can progress rapidly to deep infection, osteomyelitis, or amputation. Any new or worsening wound requires prompt professional evaluation.
 This is general information based on major guidelines (primarily 2023 IWGDF/IDSA and AAFP reviews); it is not personalized medical advice. Treatment decisions depend on individual factors, local protocols, and exam findings.
 Always involve a healthcare team experienced in diabetic foot care. Early specialist referral improves outcomes dramatically.

In short: Antibiotics when clinically infected—yes. For every wound—no. Proper wound care and addressing underlying diabetes-related issues are the foundation of treatment. Consult your doctor or a diabetic foot clinic for assessment of any specific wound.

Qwen3.6-Max-Preview:

No. Not every diabetic foot wound requires antibiotics. In fact, current clinical guidelines strongly advise against routine or prophylactic antibiotic use for uninfected diabetic foot ulcers.

Why Antibiotics Aren't Always Needed
 Colonization ≠ Infection: Nearly all chronic diabetic foot wounds harbor bacteria, but this is usually harmless colonization. Antibiotics only help when bacteria are actively invading tissue and triggering an inflammatory response.
 Uninfected wounds heal with proper local care, not systemic antibiotics.

When Antibiotics ARE Indicated
Antibiotics are recommended only when there is clinical evidence of infection, typically defined by the presence of ≥2 of the following:
 Redness, warmth, swelling, or induration around the wound
 Pain or tenderness (though neuropathy may blunt this)
 Purulent drainage
 Foul odor with tissue breakdown
 Systemic signs: fever, chills, malaise, elevated white blood cell count, or worsening glycemic control

Guidelines (IDSA, IWGDF 2023) classify infections as:
 Mild: Local signs only, <2 cm of surrounding erythema → usually oral antibiotics
 Moderate: >2 cm erythema, deeper tissue involvement, or multiple local signs → oral or IV antibiotics
 Severe: Systemic toxicity, metabolic instability, or limb-threatening spread → IV antibiotics, often hospitalization
 Suspected osteomyelitis: Requires imaging, bone culture/biopsy when possible, and prolonged targeted therapy

Risks of Unnecessary Antibiotics
 Accelerated antibiotic resistance
 Clostridioides difficile infection
 Adverse drug reactions & drug interactions
 Microbiome disruption
 Increased cost and false sense of security (delaying proper wound care)

What Actually Heals Uninfected Diabetic Foot Wounds
 Sharp debridement of necrotic/calloused tissue
 Moist wound environment with appropriate dressings
 Strict offloading (total contact cast, removable walker, or specialized footwear)
 Glycemic control and nutritional optimization
 Vascular assessment (ABI, toe pressures, Doppler) and revascularization if ischemic
 Regular monitoring by a multidisciplinary wound care team

Important Note
Diabetic foot wounds can progress rapidly to deep infection or osteomyelitis, especially in the presence of neuropathy or peripheral artery disease. Any new, worsening, or non-healing diabetic foot wound should be evaluated promptly by a clinician (podiatrist, wound care specialist, or physician) who can assess for infection, obtain cultures if needed, and determine whether antibiotics are truly indicated.

Disclaimer: This information is for educational purposes and reflects current guideline recommendations (IWGDF 2023, IDSA). It does not replace personalized medical advice. Always consult a qualified healthcare professional for diagnosis and treatment.

Q11. How can I know whether my diabetic foot infection is serious?

GPT-5.5 Thinking:

A diabetic foot infection may be serious even if it doesn’t hurt much, because nerve damage, poor circulation, or immune changes can hide typical infection signs. The IDSA/IWGDF guideline says diabetic foot infections are diagnosed mainly by clinical signs of inflammation, and they can progress quickly if not treated.

Go to the ER now / seek urgent same-day care if you have any of these

Systemic symptoms: fever, chills, feeling very ill, confusion, fast heartbeat, vomiting, or very high/unstable blood sugar.

Rapidly worsening foot signs: redness or swelling spreading up the foot/leg, increasing warmth, severe pain, red streaks, or a wound that is suddenly larger or deeper.

Dangerous wound signs: pus/drainage, foul odor, black/gray skin, dead-looking tissue, gangrene, exposed tendon/bone, a deep abscess, or suspected “pocket” of infection.

Circulation warning signs: foot/toes turning blue, purple, pale, cold, or numb; severe lower-limb ischemia is specifically considered a complication needing urgent surgical/vascular evaluation.

Call your doctor or podiatrist right away if you have

A blister, sore, ulcer, infected corn, ingrown toenail, change in foot color or temperature, new numbness/tingling/burning pain, cracked skin, or athlete’s foot between the toes. The CDC specifically says not to wait for your next appointment if these occur.

Why this matters

Guidelines recommend considering hospitalization for severe diabetic foot infections, or moderate infections with important other risks such as poor circulation. Severe infection, gangrene, necrotizing infection, deep abscess, compartment syndrome, or severe ischemia may need urgent surgical consultation.

Until you’re seen: keep weight off the foot, keep it clean and covered with a dry sterile dressing, don’t cut tissue yourself, don’t soak it, and don’t rely on leftover antibiotics.

One check: do you currently have fever/chills, spreading redness, pus/foul smell, black tissue, or a wound where bone/tendon is visible?

DeepSeek-V4:

Disclaimer: I am an AI, not a doctor. The following information is for educational purposes only and cannot replace an in-person medical assessment. If you have any doubt about a diabetic foot infection, seek medical attention immediately—delaying can lead to severe complications, including amputation.

Because diabetes can cause nerve damage (neuropathy) and poor blood flow, your body’s normal warning signs—like pain—may be muted, and healing is impaired. An infection that looks minor can become serious very quickly. Here are the key danger signs to watch for.

Signs You Need Emergency or Urgent Medical Care

If you have any of the following, go to the emergency department or contact your doctor immediately:

Signs the infection is spreading or deep:
 Redness spreading rapidly beyond the wound, especially in red streaks moving up the foot or leg.
 Severe swelling that is new or increasing, making shoes impossible to wear.
 Warmth spreading over a large area of the foot.
 A foul-smelling discharge or pus coming from the wound, especially if it’s yellow, green, or grey.
 A wound that probes to bone (if you can see a deep hole, or if a doctor can touch bone with a sterile probe, that’s a critical sign of osteomyelitis—bone infection).

Signs of systemic illness (the infection has entered your bloodstream):
 Fever or chills (a temperature over 100.4°F / 38°C) — but note: many people with diabetes do not mount a fever, so its absence does not mean the infection is mild.
 Nausea, vomiting, or feeling generally very unwell.
 Uncontrolled high blood sugar that doesn’t respond to your usual management.

Signs of critical tissue damage or gas gangrene (a surgical emergency):
 Black, purple, or dusky skin around the wound (this indicates tissue death—gangrene).
 Crackling sensation under the skin when you press on it (this suggests gas in the tissues, a life-threatening infection).
 Large blisters or skin peeling away, especially with dark fluid.

Signs related to poor circulation (critical limb ischemia):
 A foot that suddenly becomes pale, cold, and painful (or, if you have neuropathy, suddenly cold to the touch compared to the other foot).
 Pain at rest that is only relieved by dangling the foot down.

Signs a "Minor" Infection Might Be More Serious Than It Looks

Even without dramatic symptoms, a diabetic foot infection can be considered serious—or "moderate to severe"—if:
 The wound is larger than 2 cm (about the width of a nickel) of redness/cellulitis around it.
 You have deep ulceration where you can see tendons, muscle, or fat.
 There is loss of sensation in the foot (you can't feel light touch). This means you may not feel the infection worsening, which increases your risk dramatically.
 The foot pulses are absent, and the foot is cool, indicating poor blood flow. Antibiotics can’t reach the infection properly without adequate blood supply.

Why "Wait and See" Is Dangerous
With diabetes, the immune response is often blunted. You may lack classic symptoms like fever and severe pain. A small, painless blister can progress to a bone infection or limb-threatening gangrene in under 24–48 hours. Every time you delay, the risk of hospitalization, surgery, or amputation increases.

What You Should Do Right Now
1. Stop walking on the foot. Offload pressure completely. Use crutches or a wheelchair if necessary. Even a minor infection can turn a blister into a deep ulcer under weight.
2. Call your healthcare provider or go to urgent care/ER. Tell them you have diabetes and a foot infection, and clearly state which danger signs you have observed.
3. Do not soak the foot or apply home remedies. Soaking can macerate the skin and spread bacteria; hydrogen peroxide and other harsh agents can damage healing tissue.
4. Check your other foot and between toes. People with neuropathy often have “mirror” injuries they haven’t noticed.

If you’re unsure but any of these points sound familiar, err on the side of caution and be seen today. Early intervention is the single most important factor in saving a diabetic foot.

Gemini 3.1 Pro:

Diabetic foot infections can escalate very quickly, so it is crucial to have any suspected infection evaluated by a healthcare professional. As an AI, I cannot provide a medical diagnosis or assess your specific situation, but I can share the clinical "red flags" that generally indicate a diabetic foot infection is severe or potentially limb-threatening.

If you notice any of the following signs, you should seek immediate medical attention:

Systemic Signs (Whole-Body Symptoms)

These symptoms suggest the infection may be spreading into your bloodstream (sepsis), which is a medical emergency:

Fever or Chills: A high temperature, feeling feverish, or experiencing uncontrollable shaking.
 Rapid Heart Rate or Breathing: Feeling like your heart is racing or having difficulty catching your breath.
 Confusion or Lethargy: Sudden changes in your mental state, dizziness, or profound weakness.
 Uncontrolled Blood Sugar: A sudden, unexplained spike in your blood glucose levels that becomes difficult to manage, as the body's response to severe infection often drives up blood sugar.

Local Signs (At the Foot)

These localized symptoms indicate that the infection is deep, aggressive, or damaging vital tissues:

Spreading Redness and Swelling: Redness (erythema) that rapidly spreads more than 2 centimeters (about an inch) beyond the edge of the wound, accompanied by significant swelling or warmth.
 Deep Tissue Exposure: If the ulcer is deep enough that you can see bone, tendon, or joint capsule.
 Tissue Color Changes: Skin or tissue that turns black, blue, or grayish, which is a sign of necrosis (dead tissue) or gangrene.
 Foul Odor or Heavy Drainage: A strong, unpleasant smell coming from the wound, or a sudden increase in pus (purulent discharge) and weeping.
 Crepitus: A unusual crackling, popping, or "bubble wrap" sensation under the skin when you press near the wound. This is caused by gas-producing bacteria and requires immediate emergency care.
 New or Worsening Pain: While diabetic neuropathy can often mask pain, any sudden onset of severe pain in the foot or leg is a major warning sign.

What You Should Do Next:
If you are experiencing any of the systemic signs or severe local signs (like black tissue, deep exposure, or spreading redness), do not wait to see if it improves. Go to an emergency room or seek urgent medical care immediately. Early intervention, which may include intravenous antibiotics or surgical cleaning (debridement), is the most effective way to prevent serious complications.

Grok 4.3:

I am not a doctor, and this is not personalized medical advice. Diabetic foot infections can progress rapidly and lead to serious complications like bone infection (osteomyelitis), gangrene, sepsis, or amputation. Any suspected infection in a person with diabetes requires prompt professional evaluation by a healthcare provider (ideally a podiatrist, wound care specialist, or multidisciplinary foot team). If you have signs of a severe infection, go to the emergency room immediately—do not wait.

How Infection Is Diagnosed
Infection is primarily a clinical diagnosis based on local signs in the foot (below the ankle). According to IDSA/IWGDF guidelines, at least two of these indicate infection (even if pain is absent due to neuropathy):

Erythema (redness)
 Warmth
 Swelling or induration
 Tenderness or pain
 Purulent (pus) drainage

Foul odor, increasing drainage, or non-healing wounds also raise concern. Blood tests, imaging (X-ray, MRI), or deep tissue cultures help confirm depth and bone involvement.

IWGDF/IDSA Classification of Severity (2023 Guidelines)
Clinicians use this standardized system to guide treatment, hospitalization, and urgency:

Mild (usually outpatient treatment): Superficial (skin/subcutaneous only). Limited erythema (<2 cm around the wound). No deeper involvement or systemic illness.
 Moderate: Deeper tissues involved (tendon, muscle, joint, or bone), erythema ≥2 cm, or lymphangitis (red streaks), but the patient is systemically well (no fever or major lab abnormalities).
 Severe: Any infection with systemic signs (e.g., fever >38°C/100.4°F or low temperature, heart rate >90, respiratory rate >20, abnormal white blood cell count) or critical local features like extensive gangrene, necrotizing (flesh-eating) infection, deep abscess, compartment syndrome, or severe limb ischemia (poor blood flow).

Osteomyelitis (bone infection) is noted separately (e.g., “moderate with osteomyelitis”).

Hospitalization is often recommended for severe infections or moderate ones with complicating factors (poor circulation, uncontrolled diabetes, kidney disease, etc.).

Red Flags: When the Infection Is Likely Serious
Seek emergency care (ER or call emergency services) right away if you notice any of these—they signal possible spread, tissue death, or sepsis:

Local (foot/leg) signs:
 Rapidly spreading redness, warmth, or swelling
 Black, purple, or dead-looking tissue (gangrene/necrosis)
 Exposed bone, tendon, or joint
 Foul-smelling or heavy pus/drainage
 Crepitus (crackling sensation under skin—suggests gas-forming bacteria)
 Red streaks running up the leg (lymphangitis)

Systemic (whole-body) signs:
 Fever, chills, or feeling very unwell
 Confusion, dizziness, or rapid heartbeat
 Unexplained high or unstable blood sugar
 Nausea, vomiting, or low blood pressure

Other high-risk situations:
 No improvement after 48–72 hours of treatment
 History of peripheral artery disease (cold/pale foot, weak pulses)
 Probe-to-bone test positive (a sterile probe reaches bone through the ulcer—highly suggestive of osteomyelitis)
 Recent trauma, long-standing ulcer, or immunosuppression

Even without obvious pain (due to neuropathy), these warrant urgent attention. Gangrene or necrotizing infection is a true emergency that can become life-threatening within hours.

What Happens Next (Professional Evaluation)
A specialist will:
 Perform a full foot exam (including vascular checks and probe-to-bone test)
 Order labs (CBC, inflammatory markers like CRP/ESR), blood cultures if severe, and imaging
 Take deep tissue samples for culture (not surface swabs)
 Start empiric antibiotics (often broad-spectrum for moderate/severe cases), surgical debridement (removal of dead tissue), offloading (special boots/casts), and wound care
 Consider vascular intervention if circulation is poor

Antibiotic duration is often shortened (e.g., ~10 days after good debridement for many soft-tissue infections per recent guidelines) to reduce resistance. Surgery may be needed for abscesses, dead tissue, or bone infection.

Immediate Steps You Can Take While Seeking Care
 Keep the foot elevated and clean/dry
 Avoid walking on the affected foot if possible (use crutches or a wheelchair)
 Monitor blood glucose closely and treat highs
 Do not apply heat, soak in water, or use over-the-counter remedies without guidance
 Note all symptoms, medications, and recent blood sugar readings for the doctor

Prevention is key for the future: Inspect feet daily (use a mirror or helper), wear well-fitting shoes, control blood sugar, see a podiatrist regularly, and address any callus or minor injury immediately.

Bottom line: Err on the side of caution—diabetic foot infections are among the leading causes of hospitalization and amputation in diabetes. Early intervention dramatically improves outcomes. Contact your doctor, diabetes care team, or go to the ER now if anything seems off. If you provide more details about your symptoms (with the understanding this isn’t a diagnosis), I can help point you toward relevant questions to ask your provider. Take care.

Qwen3.6-Max-Preview:

Diabetic foot infections can progress rapidly and become life- or limb-threatening due to reduced circulation, nerve damage, and impaired immune function. Any foot wound or sign of infection in a person with diabetes should be evaluated by a healthcare professional promptly. Below are evidence-based warning signs that suggest an infection may be serious and requires urgent medical attention.

Seek immediate medical care (urgent care or ER) if you notice any of the following:
 Systemic symptoms: Fever, chills, nausea, vomiting, confusion, dizziness, rapid heartbeat, or feeling unusually weak/faint
 Rapidly spreading redness, swelling, or warmth that extends beyond the original wound site
 Increasing pain, or a sudden loss of sensation/numbness in the area (neuropathy can mask worsening infection)
 Pus, thick drainage, or a foul odor from the wound
 Black, gray, blue, or leathery skin (possible tissue death/gangrene)
 A wound that is deepening, widening, or exposing tendon, joint, or bone
 Inability to bear weight or walk normally
 Cold, pale, or bluish foot with weak or absent pulses
 No improvement or worsening after 24–48 hours of proper wound care

Why diabetes increases risk:
 High blood sugar slows healing and weakens immune defenses
 Peripheral neuropathy can dull pain, allowing infections to advance unnoticed
 Poor circulation limits delivery of antibiotics and oxygen to the tissue
 Even small cuts, blisters, or calluses can become deep infections or lead to osteomyelitis (bone infection) or sepsis

What to do right now:
 Do not wait if you have any red flags above. Go to urgent care or the ER.
 Keep the foot clean, dry, and elevated. Avoid walking on it.
 Do not soak the foot, use hydrogen peroxide/alcohol, attempt to drain it, or cut away tissue yourself.
 Check and manage your blood glucose closely; high levels worsen infection risk.
 Contact your primary care doctor, endocrinologist, or podiatrist as soon as possible. They may need to culture the wound, order imaging (like an X-ray or MRI), prescribe targeted antibiotics, or refer you to a wound care or vascular specialist.

Important: This information is educational and not a substitute for professional medical evaluation. Diabetic foot infections often require clinical assessment, even when they appear mild. Early intervention significantly reduces the risk of hospitalization, amputation, or systemic illness.

If you're unsure whether your symptoms warrant urgent care, err on the side of caution and seek in-person evaluation today.

Q12. How would doctors know if my diabetic foot infection may have spread to the bone?

GPT-5.5 Thinking:

Doctors look for osteomyelitis, meaning infection in the bone. They usually do this in layers rather than with one single test.

What they check first

They’ll examine the wound and may do a probe-to-bone test, where a sterile blunt metal probe is gently inserted into the ulcer to see whether bone can be felt. If bone is exposed or easily reached, suspicion goes up.

They’ll also usually order plain foot X-rays and blood tests such as ESR, CRP, and sometimes procalcitonin. Current IWGDF/IDSA guidance recommends using a combination of probe-to-bone exam, plain X-rays, and inflammatory markers as initial studies when diabetic foot osteomyelitis is suspected.

Imaging if it’s still unclear

If the exam, X-ray, and blood tests don’t clearly answer the question, doctors often order an MRI, which is the preferred next imaging test when the diagnosis remains in doubt. MRI can show bone marrow changes, abscesses, sinus tracts, and how far the infection may extend.

If MRI cannot be done, they may use other imaging such as PET, white blood cell scintigraphy, or SPECT as alternatives.

The most definitive test

The closest thing to a “proof” test is a bone sample, taken during surgery or through the skin, for culture and sometimes pathology. Guidelines specifically say that when osteomyelitis is suspected, doctors should consider obtaining bone rather than soft-tissue samples for culture.

Clues that raise concern

Doctors worry more about bone involvement when there is a deep or long-standing ulcer, visible or palpable bone, a wound over a bony prominence, persistent drainage, swelling or redness that is not improving, abnormal X-ray changes, or very elevated inflammatory markers. A diabetic foot infection with spreading redness, fever, chills, black tissue, severe pain, foul drainage, or rapidly worsening swelling needs urgent medical care.

This is worth getting assessed promptly, especially with diabetes, because nerve damage can make a serious infection feel less painful than expected.

DeepSeek-V4:

This is a very important concern, and I’m glad you’re aware of it. A diabetic foot infection that spreads to bone is called osteomyelitis, and it’s a serious complication because it often requires prolonged antibiotics or even surgery. Doctors use a combination of bedside clues and specific tests to figure out if the bone is involved.

Here’s how they typically approach it:

1. The “Probe-to-Bone” Test (Bedside)
This is often the first and simplest check. Using a sterile, blunt metal probe, the doctor will gently explore the wound. If the probe can touch a hard, gritty surface that feels like bare bone, the likelihood of osteomyelitis is very high. In a diabetic foot ulcer, a positive “probe-to-bone” test is a strong predictor that the infection is in the bone.

2. Imaging Studies
If the diagnosis is still unclear, or to see the extent of infection, they’ll use imaging:

X-ray: Usually the first test ordered. However, it can take 2–4 weeks for enough bone destruction to show up as changes (like a moth-eaten appearance). A normal early X-ray does not rule out osteomyelitis.
 MRI: This is the most sensitive non-invasive test. It can show bone marrow swelling and inflammation very early, along with soft-tissue abscesses and sinus tracts. MRI is the gold standard imaging tool for diagnosing osteomyelitis in the foot.
 Nuclear Medicine Scans: If MRI can’t be done (e.g., due to metal implants or claustrophobia), a three-phase bone scan or a labeled white blood cell scan may be used, though they are less specific.

3. Clinical Signs (What you or the doctor may notice)
Alongside the tests, doctors watch for physical signs that are red flags for deep infection:

A deep ulcer that doesn’t heal over weeks, especially over a bony prominence.
 A “sausage toe” – a red, swollen digit that looks like a small sausage, which is classic for underlying bone infection.
 The wound has a tiny opening (sinus tract) that seems to go deep.
 You can sometimes feel bone moving or grating, but usually the probe test is done first.

4. Blood Tests
Blood markers like white blood cell count, C-reactive protein (CRP), and erythrocyte sedimentation rate (ESR) are often checked. They confirm there is inflammation or infection somewhere in the body, but they can’t tell if it’s in skin, soft tissue, or bone. Normal markers make osteomyelitis much less likely, but elevated ones simply support the suspicion.

5. The Gold Standard: Bone Biopsy
If the diagnosis remains uncertain or if the exact bacteria need to be identified (because the infection isn’t responding to standard antibiotics), the doctor may take a small sample of bone. This can be done with a needle under imaging guidance or during a surgical debridement. The sample is sent for culture and histology – looking at the cells under a microscope. A positive culture and evidence of dead bone confirm the diagnosis definitively.

Why it matters: If osteomyelitis is caught early, it can sometimes be treated with high-dose, long-term antibiotics alone. If it’s advanced, dead bone (sequestrum) acts like a splinter that antibiotics can’t reach, and the dead piece must be surgically removed for the infection to clear.

If you’re worried that a foot wound isn’t healing, or if you can see deep tissue, notice increasing redness, swelling, or a foul odor, please see your doctor or a podiatrist urgently. They will likely start with the simple probe test and an X-ray, then decide if an MRI or biopsy is needed.

This information is for educational purposes and not a substitute for in-person medical evaluation. It’s important to have any concerning diabetic foot wound assessed promptly.

Gemini 3.1 Pro:

When determining if a diabetic foot ulcer has progressed to a bone infection (osteomyelitis), clinicians follow a systematic diagnostic pathway. The evaluation integrates bedside clinical findings, serum inflammatory markers, advanced imaging, and definitive microbiological testing.

Clinical Examination

Probe-to-Bone (PTB) Test: This is a highly predictive, standard bedside procedure. A clinician gently inserts a sterile, blunt metal probe into the base of the ulcer. If the probe contacts a hard, gritty surface, it strongly correlates with underlying bone involvement.
 Wound Morphology: An ulcer that is larger than 2 cm², exposes deep structures, or fails to heal after 1 to 2 weeks of standard offloading and wound care raises a high clinical suspicion. Additionally, a globally swollen, erythematous toe (often referred to as a "sausage toe" or dactylitis) is a classic presentation of underlying osteomyelitis.

Laboratory Markers

While acute systemic markers like an elevated white blood cell (WBC) count can be present, they frequently remain within normal limits during chronic, localized infections.

Inflammatory Indices: Markedly elevated Erythrocyte Sedimentation Rate (ESR)—often considered a key threshold when >70 mm/hr—and C-Reactive Protein (CRP) are strong systemic indicators of bone infection when combined with clinical suspicion.

Imaging Modalities

Plain Radiography (X-ray): Standard radiographs are always the first-line imaging choice to rule out fractures, structural deformities (like Charcot neuroarthropathy), or gas in the soft tissues. However, their early sensitivity for osteomyelitis is low; visible cortical erosion or periosteal reaction typically takes 10 to 14 days of bone demineralization to appear.
 Magnetic Resonance Imaging (MRI): MRI is the gold standard imaging modality for diagnosing osteomyelitis. It provides exceptional soft-tissue contrast and high sensitivity for detecting bone marrow edema, making it possible to identify the infection long before osteolytic changes are visible on an X-ray.
 Advanced Nuclear Imaging: If an MRI is contraindicated (e.g., due to certain pacemakers or severe renal impairment preventing contrast use), alternative modalities like a radiolabeled leukocyte (WBC) scan or a PET/CT may be employed.

Definitive Diagnosis

Bone Biopsy: While the PTB test and an MRI provide a robust presumptive diagnosis, a percutaneous or surgical bone biopsy is the definitive standard. Histopathological evaluation confirms the presence of bone necrosis and inflammatory cells, while microbiological culturing of the bone tissue identifies the specific causative pathogens, allowing for precisely targeted antibiotic therapy.

Disclaimer: This information is provided for educational purposes. Because diabetic foot infections can rapidly escalate and pose significant risks, including amputation, they require immediate, in-person medical evaluation.

Grok 4.3:

This is general information only and not personalized medical advice. Diabetic foot infections that reach the bone (called osteomyelitis or diabetes-related foot osteomyelitis) are serious and raise the risk of amputation. See your doctor, podiatrist, or a wound-care/infectious-disease specialist immediately if you have a foot ulcer, redness, swelling, drainage, or any new foot problem. Early evaluation is essential.

Doctors determine whether a soft-tissue foot infection has spread to bone through a stepwise combination of clinical evaluation, simple office tests, blood work, imaging, and sometimes tissue sampling. They follow evidence-based guidelines (such as those from the Infectious Diseases Society of America and International Working Group on the Diabetic Foot).

Clinical signs and bedside testing
 The doctor examines the ulcer or wound for depth, size (ulcers larger than about 2 cm raise concern), exposed bone, foul drainage, or surrounding inflammation (warmth, redness, swelling, or tenderness). Because of diabetic neuropathy, pain may be minimal or absent even with deep infection.
 Probe-to-bone test: This quick, painless office procedure uses a sterile blunt metal probe gently inserted into the wound. If the probe touches hard or gritty bone without soft tissue in between, it strongly suggests osteomyelitis (positive likelihood ratio around 6 in studies). A negative test lowers but does not eliminate suspicion.

Blood tests
Inflammatory markers help support the diagnosis:
 Erythrocyte sedimentation rate (ESR) — values above 70 mm/h are suggestive.
 C-reactive protein (CRP) or procalcitonin (PCT) — elevated levels indicate active infection.
These are not specific to bone but, when combined with the probe test and X-ray, improve accuracy for initial assessment.

Imaging studies
 Plain X-rays are usually the first imaging test. They can show bone erosion, periosteal reaction, or lucency, but changes often take 2–3 weeks to appear, so a normal X-ray does not rule out early osteomyelitis.
 MRI (magnetic resonance imaging) is the most accurate and commonly recommended next step when suspicion remains after X-ray and lab tests. It detects bone marrow edema and infection very early and helps differentiate osteomyelitis from Charcot foot or other bone changes.
 Other options (if MRI is unavailable or contraindicated) include CT scan, leukocyte scintigraphy (bone scan), PET scan, or SPECT. These are used selectively.

Definitive confirmation
When imaging or clinical findings are inconclusive, or when the plan involves prolonged antibiotics without surgery, doctors obtain a bone biopsy (percutaneous under imaging guidance or during surgical debridement). This provides:
 Culture to identify the exact bacteria (often polymicrobial, including Staphylococcus or Streptococcus species).
 Histopathology to confirm infection in the bone tissue.

Superficial wound swabs are avoided because they often reflect only surface contaminants rather than the true deep or bone pathogen.

Additional context in diabetes
Poor circulation, neuropathy, and immune changes make spread to bone more likely and harder to detect clinically. Vascular studies (ankle-brachial index, toe pressure, or angiography) may be done concurrently to assess healing potential. A multidisciplinary team (endocrinology, podiatry, vascular surgery, infectious disease) usually coordinates care.

If osteomyelitis is confirmed, treatment typically includes targeted antibiotics (often 4–6 weeks or longer), surgical debridement or resection of infected bone, off-loading, glycemic control, and wound care. In some selected cases, antibiotics alone may suffice if the infection is limited and bone can be stabilized.

Seek care today — do not wait. Prompt diagnosis dramatically improves outcomes and limb salvage. Your healthcare provider will tailor the exact sequence of tests to your specific situation, ulcer depth, and overall health.

Qwen3.6-Max-Preview:

When a diabetic foot infection is suspected of spreading to the bone (a condition called diabetic foot osteomyelitis, or DFO), doctors use a stepwise, evidence-based approach to confirm or rule it out. Because bone infection changes treatment significantly (longer antibiotics, possible surgery, closer monitoring), accurate diagnosis is critical.

Here’s how clinicians typically evaluate for bone involvement:

1. Clinical Examination & Bedside Tests
 Probe-to-bone test: A sterile, blunt metal probe is gently inserted into the base of the ulcer. If it touches hard, gritty bone, this is highly suggestive of osteomyelitis (specificity ~85–90% in diabetic foot ulcers).
 Ulcer characteristics: Deep wounds (>3 mm), ulcers present >2 weeks despite proper care, visible/exposed bone, or ulcers over bony prominences (toes, metatarsal heads, heel) raise suspicion.
 Local signs: Persistent swelling, warmth, redness, purulent drainage, or a "sausage toe" appearance.
 Systemic symptoms: Fever, chills, or elevated heart rate are actually uncommon in DFO, so their absence doesn't rule out bone infection.

2. Laboratory Markers
 ESR (Erythrocyte Sedimentation Rate): Often the most useful blood test. An ESR >70 mm/hr strongly increases the likelihood of osteomyelitis.
 CRP (C-Reactive Protein): Usually elevated, but less specific than ESR for bone involvement.
 WBC count: Frequently normal or only mildly elevated in DFO.
 Wound swabs: Not reliable for diagnosing bone infection. Superficial cultures often grow colonizing bacteria that don't reflect what's in the bone.

3. Imaging Studies
 Plain X-ray: Always the first imaging step. Looks for cortical erosion, periosteal reaction, bone destruction, or gas. However, bone changes often take 2–4 weeks to appear, so a normal early X-ray doesn't rule out osteomyelitis.
 MRI (Magnetic Resonance Imaging): The most accurate non-invasive test. Detects bone marrow edema, cortical breakdown, abscesses, and sinus tracts. Sensitivity and specificity are both ~90%. Usually ordered if clinical suspicion remains after X-ray.
 CT Scan: Better for visualizing cortical bone detail, sequestra (dead bone), or surgical planning. Used if MRI is contraindicated (e.g., certain pacemakers, severe claustrophobia).
 Nuclear Medicine Scans (WBC scan, bone scan, PET): Reserved for complex cases or when MRI/CT are inconclusive or unavailable. Less specific but can help differentiate soft tissue vs. bone infection.

4. Definitive Diagnosis: Bone Biopsy
 Gold standard: A sample of bone (obtained percutaneously or during surgery) sent for culture and histopathology.
 Confirms infection, identifies the exact pathogen(s), and guides targeted antibiotic therapy.
 Typically pursued when:
 Diagnosis remains uncertain after imaging
 Infection isn't responding to empiric antibiotics
 Surgical debridement or amputation is being considered
 Long-term IV antibiotics are planned and precise culture data is needed

How Doctors Put It Together
Guidelines from the International Working Group on the Diabetic Foot (IWGDF) and Infectious Diseases Society of America (IDSA) recommend a practical algorithm:
1. High clinical suspicion + positive probe-to-bone or ESR >70 → treat as probable osteomyelitis
2. X-ray first → if negative but suspicion remains → MRI
3. If imaging is equivocal, or if culture-directed therapy is needed → bone biopsy
4. Diagnosis is often classified as possible, probable, or definite based on combined clinical, lab, and imaging findings.

When to Seek Prompt Medical Attention
Contact your healthcare team urgently if you notice:
 Rapidly spreading redness, swelling, or pain
 Foul-smelling or increased drainage
 Black, blue, or pale/discolored tissue
 Fever, chills, nausea, or confusion
 An ulcer that isn't improving after 1–2 weeks of proper wound care and offloading

Important Notes
 Diabetic foot osteomyelitis usually requires 4–6 weeks of antibiotics (often IV initially, then oral), plus meticulous wound care, offloading, and sometimes surgical debridement.
 Multidisciplinary care (podiatry, infectious disease, endocrinology, vascular surgery, wound care) significantly improves outcomes.
 Early diagnosis and treatment greatly reduce the risk of amputation.

Disclaimer: This information is for educational purposes and reflects current clinical guidelines. Only a qualified healthcare provider who can examine you, review your imaging/labs, and consider your full medical history can diagnose or rule out bone infection. If you have a diabetic foot ulcer or infection, please seek prompt in-person medical evaluation.

Q13. How does poor blood flow affect a diabetic foot wound?

GPT-5.5 Thinking:

Poor blood flow can turn a diabetic foot wound from a normal healing problem into a limb-threatening wound.

Blood brings the wound what it needs to heal: oxygen, immune cells, nutrients, growth factors, and enough circulation for antibiotics to reach infected tissue. When circulation is poor, the wound gets less oxygen and fewer healing cells, so new tissue forms slowly, infection is harder to control, and dead tissue is more likely to develop. Diabetes can also damage both larger arteries and smaller vessels, reducing tissue perfusion and impairing healing.

In a diabetic foot ulcer, poor circulation often comes from peripheral artery disease, where narrowed leg/foot arteries limit blood supply. Guidelines emphasize checking circulation early in diabetic foot ulcer care because restoring blood flow with a vascular procedure can sometimes make healing possible.

Poor blood flow also raises the risk of infection spreading, gangrene, and amputation. Reviews note that perfusion deficits contribute to delayed wound healing and increased amputation risk; many lower-limb amputations in people with diabetes are preceded by a foot ulcer.

Signs that blood flow may be poor include a wound that is not shrinking, pale/blue/dark toes, cool foot skin, weak or absent pulses, pain at rest or at night, black tissue, or increasing redness, swelling, drainage, odor, fever, or worsening pain. The American Diabetes Association notes that if an ulcer is not healing and circulation is poor, referral to a vascular surgeon may be needed.

A diabetic foot wound that is worsening, infected, blackening, or not healing should be assessed promptly by a clinician, podiatrist, wound-care specialist, or vascular specialist. If there is fever, rapidly spreading redness, severe pain, foul drainage, or black tissue, that is urgent.

DeepSeek-V4:

Poor blood flow (ischemia) is one of the most critical factors that turns a simple diabetic foot wound into a limb-threatening emergency. In diabetes, both large vessels (macroangiopathy, like peripheral artery disease) and small capillaries (microangiopathy) are often narrowed or blocked. Here’s exactly how that impaired circulation derails every stage of healing.

1. Oxygen and Nutrient Starvation
Healing is an energy‑intensive process. Cells that rebuild tissue—fibroblasts and skin cells—need a constant supply of oxygen and glucose to multiply and synthesize collagen.
 Without adequate blood flow, the wound bed becomes hypoxic. Collagen production falters, new blood vessel formation (angiogenesis) stalls, and the wound simply cannot “build” new tissue.
 This leads to a chronic, stagnant ulcer that fails to fill in with healthy granulation tissue.

2. Immune System Paralysis
Your blood is the highway for white blood cells and antibodies to reach an infection.
 Poor perfusion means fewer neutrophils and macrophages arrive at the wound. Any bacteria that enter the foot multiply virtually unchecked.
 Because the immune response is blunted, the classic signs of infection (redness, heat, pus) are often absent or minimal, even when a deep, limb‑threatening infection is brewing. This is one reason infections silently spread in diabetic feet.

3. Antibiotics Cannot Reach the Target
Even if the right antibiotic is prescribed, it travels through the bloodstream. In a poorly perfused foot, the antibiotic concentration in the wound tissue can be far below the level needed to kill bacteria. This creates a perfect environment for drug‑resistant organisms and failed antibiotic therapy that can only be corrected by restoring blood flow (revascularization).

4. Breakdown of the Skin Barrier and Autonomic Dysfunction
Diabetic neuropathy often accompanies poor blood flow. Autonomic nerve damage reduces sweat and oil gland function, leaving the skin dry, cracked, and prone to fissures—new entry points for infection. The lack of blood flow then prevents these micro‑injuries from healing, expanding the wound.

5. The Cascade to Non‑Healing and Tissue Death
 Cell death (necrosis): Without a basal level of oxygen, tissue simply dies, turning black and dry (dry gangrene) or becoming wet and infected (wet gangrene).
 Sharp wound edges: In a well‑perfused wound, edges are pink and healing. With ischemia, edges become punched‑out, pale, and devoid of any healing attempt.
 Pain discrepancy: Paradoxically, severe ischemia can cause extreme pain (rest pain), but neuropathy may mask it. So the patient feels nothing, while tissue dies.

6. Major Clinical Consequences
 A small blister or callus that would heal in a healthy person cannot close. It enlarges, deepens, and exposes tendons and bone.
 The combination of ischemia + infection + neuropathy is the classic triad leading to chronic osteomyelitis (bone infection) and the need for minor or major amputation.
 Studies show that in diabetic foot ulcers, the presence of significant peripheral artery disease (poor blood flow) is the single strongest predictor of non‑healing and amputation.

Practical Takeaway
This is why vascular assessment (ankle‑brachial index, toe pressures, Doppler) is mandatory in every diabetic foot wound. If blood flow is too low, no amount of advanced dressings or antibiotics will heal the ulcer. Revascularization—via angioplasty, stenting, or bypass surgery—must often come first to salvage the limb. Restoring pulsatile flow literally transforms a cold, blue, non‑healing wound into a warm, pink one that can finally begin to repair itself.

Gemini 3.1 Pro:

Poor blood flow, or ischemia, is a critical factor that severely complicates the healing process of diabetic foot wounds. In individuals with diabetes, chronic high blood sugar can damage blood vessels over time, frequently leading to Peripheral Arterial Disease (PAD), which narrows the arteries and restricts circulation to the lower extremities.

When a foot wound (ulcer) occurs, this restricted blood flow impacts the body's natural healing mechanisms in several distinct ways:

1. Cellular Hypoxia and Nutrient Deprivation

For a wound to heal, the affected tissue requires a massive increase in oxygen and metabolic nutrients.

Collagen Synthesis: Oxygen is essential for fibroblasts to synthesize collagen, the structural protein necessary to rebuild tissue and close the wound. Poor blood flow results in a hypoxic (low oxygen) environment, halting this process.
 Energy Deficit: The cells responsible for repairing tissue are deprived of glucose and amino acids, preventing the formation of healthy granulation tissue (the new connective tissue that fills a wound).

2. Impaired Immune Response and Infection Risk

A robust blood supply is necessary to deliver the body's immune cells to the site of an injury.

Reduced Leukocyte Delivery: With limited circulation, fewer white blood cells (like neutrophils and macrophages) reach the wound to clear away dead tissue and destroy invading bacteria.
 Rapid Bacterial Colonization: Diabetic foot wounds are already prone to infection due to neuropathy (lack of sensation). Without an adequate local immune response, simple colonizations can quickly escalate into deep tissue infections or bone infections (osteomyelitis).

3. Ineffective Medication Delivery

If a diabetic foot wound becomes infected, standard treatments usually involve systemic oral or intravenous antibiotics.

Blocked Transport: Because the blood vessels leading to the foot are narrowed or blocked, the antibiotics cannot reach the wound bed in high enough concentrations to effectively eradicate the bacteria, making the infection incredibly difficult to manage.

4. Impaired Angiogenesis

Healing requires angiogenesis, which is the formation of new blood vessels from pre-existing ones to supply the newly forming tissue.

Stalled Remodeling: The endothelial cells that drive angiogenesis require oxygen and specific growth factors delivered via the bloodstream. Ischemia prevents these chemical signals from functioning correctly, leaving the wound bed stagnant and unable to vascularize.

The Clinical Consequence: Necrosis and Gangrene

When a wound is starved of oxygen and immune support, the tissue begins to die (necrosis). If the bacterial load increases unchecked in this dead tissue, it can lead to gangrene. At this stage, the tissue cannot be salvaged, and surgical intervention—ranging from aggressive debridement (removal of dead tissue) to amputation of the toe, foot, or lower leg—often becomes necessary to stop the spread of infection to the rest of the body.

Grok 4.3:

Poor blood flow (peripheral arterial disease or ischemia) severely impairs the healing of diabetic foot wounds and dramatically increases the risk of complications.

In diabetes, high blood sugar damages blood vessels over time, causing atherosclerosis (plaque buildup) that narrows arteries in the legs and feet. This reduces oxygen-rich blood, nutrients, and immune cells reaching the wound site. Here’s exactly how it affects the wound:

Delayed or stalled healing: Cells need oxygen and nutrients (glucose, amino acids, vitamins) to produce collagen, form new blood vessels (angiogenesis), and repair tissue. Without adequate delivery, fibroblasts and keratinocytes function poorly. The wound stays stuck in the inflammatory phase instead of progressing to proliferation and remodeling.

Tissue hypoxia and cell death: Low oxygen (hypoxia) triggers anaerobic metabolism, lactic acid buildup, and eventual necrosis (tissue death). Even small injuries can expand into larger ulcers because surrounding tissue dies from lack of perfusion.

Weakened immune defense and higher infection risk: White blood cells, antibodies, and antibiotics cannot reach the wound effectively. Bacteria thrive in the low-oxygen, nutrient-poor environment, leading to deeper infections (cellulitis, osteomyelitis) that are harder to clear. Gangrene (wet or dry) can develop rapidly.

Chronic ulcer formation: Minor trauma (often unnoticed due to concurrent neuropathy) evolves into non-healing ulcers. The combination of poor circulation and high blood sugar creates a vicious cycle—wounds worsen, more tissue is lost, and new vessels cannot form properly.

Increased amputation risk: Studies consistently show that ischemia is one of the strongest predictors of major amputation in diabetic foot disease. Once blood flow drops below critical levels (often measured by ankle-brachial index <0.9 or toe pressure <30 mmHg), salvage becomes difficult without revascularization (bypass or angioplasty).

Key compounding factors:
 Neuropathy masks pain, so patients continue walking on the wound, increasing pressure and further damaging fragile tissue.
 Hyperglycemia thickens capillary basement membranes and impairs white blood cell function, amplifying the circulation problem.
 Edema (swelling) from venous issues or infection compresses remaining capillaries, worsening ischemia.

Clinical reality: A diabetic foot wound with poor blood flow is classified as an “ischemic” or “neuroischemic” ulcer (Wagner or University of Texas staging). Healing rates drop sharply—many require months of specialized care (offloading, debridement, infection control, and vascular intervention) and still carry a 5–10% annual major amputation risk if circulation is not restored.

In short, poor blood flow turns a potentially manageable wound into a limb-threatening emergency by starving the repair process, inviting infection, and allowing silent progression to irreversible tissue loss. Early vascular assessment (Doppler, angiography) and prompt revascularization are often the difference between limb salvage and amputation.

Qwen3.6-Max-Preview:

Poor blood flow (peripheral arterial disease and microvascular dysfunction) is one of the most critical factors that impair healing in diabetic foot wounds. In diabetes, chronic hyperglycemia damages both large and small blood vessels, reducing perfusion to the lower extremities. This ischemia disrupts nearly every aspect of the wound healing process and significantly increases the risk of complications.

How Poor Blood Flow Impairs Wound Healing
1. Reduced Oxygen & Nutrient Delivery
 Wound healing is highly metabolically active. Adequate perfusion supplies oxygen, glucose, amino acids, and vitamins required for cell proliferation, collagen synthesis, and tissue rebuilding. Ischemia starves the wound bed, stalling repair.

2. Impaired Immune Function
 Neutrophils and macrophages need robust blood flow to reach the wound. Hypoxia weakens their ability to phagocytose bacteria, produce reactive oxygen species for microbial killing, and clear necrotic debris. This creates a high risk of infection and biofilm formation.

3. Slowed Waste Removal & Toxic Microenvironment
 Poor circulation traps metabolic byproducts, inflammatory cytokines, and lactic acid in the wound. The resulting acidic, pro-inflammatory environment damages healthy cells and prolongs the inflammatory phase.

4. Disrupted Growth Factor Signaling & Angiogenesis
 Ischemia impairs the release and function of key growth factors (e.g., VEGF, PDGF, TGF-β) that drive new blood vessel formation, fibroblast migration, and re-epithelialization. Without angiogenesis, the wound cannot progress to proliferation.

Impact on the Phases of Healing
 Hemostasis: May occur normally, but fragile clots form in ischemic tissue.
 Inflammation: Becomes chronic due to poor bacterial clearance and persistent hypoxia.
 Proliferation: Fibroblast activity, collagen deposition, and granulation tissue formation are severely delayed or absent.
 Remodeling: Collagen remains disorganized and weak, leaving healed tissue prone to breakdown and re-ulceration.

Clinical Consequences in Diabetes
 Wounds stall and become chronic ulcers (often lasting months or years)
 Infection risk skyrockets, potentially progressing to cellulitis, abscess, or osteomyelitis
 Tissue necrosis or gangrene can develop if perfusion falls below critical thresholds
 Amputation risk increases dramatically; ischemia is a leading predictor of major lower-limb amputation in diabetes
 Often painless due to concurrent diabetic neuropathy, delaying recognition and treatment

Why Diabetes Makes This Worse
 Macrovascular disease: Accelerated atherosclerosis narrows leg arteries (PAD)
 Microvascular dysfunction: Capillary basement membrane thickening, reduced capillary density, and impaired endothelial nitric oxide production limit tissue-level perfusion
 Neuropathy + Ischemia = "Perfect Storm": Loss of protective sensation leads to unrecognized trauma, while poor flow prevents healing
 Hyperglycemia: Promotes oxidative stress, advanced glycation end-products (AGEs), and chronic inflammation, all of which worsen vascular dysfunction

Management Considerations
 Vascular assessment is essential: Ankle-brachial index (ABI), toe pressures, Doppler ultrasound, or angiography to quantify perfusion
 Revascularization when feasible: Endovascular procedures or bypass surgery can restore flow and salvage limbs
 Optimize systemic factors: Tight glycemic control, smoking cessation, lipid/BP management, and antiplatelet therapy
 Advanced wound care: Sharp debridement (if perfusion allows), strict offloading, moisture-balanced dressings, and infection control
 Adjuncts in selected cases: Hyperbaric oxygen therapy, negative pressure wound therapy, or growth factor/biologic dressings may help when perfusion is partially restored
 Multidisciplinary care: Podiatry, vascular surgery, endocrinology, and wound care specialists improve outcomes significantly

Key takeaway: Poor blood flow doesn't just slow diabetic foot wound healing; it fundamentally alters the wound microenvironment, turning acute injuries into chronic, high-risk lesions. Early vascular evaluation and aggressive perfusion optimization are as critical as local wound care.

Disclaimer: This information is for educational purposes and does not replace professional medical advice. Diabetic foot wounds require prompt evaluation by a qualified healthcare provider to prevent serious complications.

Q14. My foot is cold, darker, or hurts when I walk. Could this mean poor circulation?

GPT-5.5 Thinking:

Yes — a cold foot, darker/blue/pale color, or pain when walking can be signs of poor circulation, including peripheral artery disease (PAD). PAD commonly causes leg/foot pain or cramping during walking that improves after resting, and it can also cause one foot to feel colder, color changes, weak pulses, slow nail/hair growth, shiny skin, or non-healing sores.

Seek emergency care now if the change is sudden or severe, especially if one foot is much colder or paler/bluer/darker than the other, you have severe pain, numbness/tingling, weakness, trouble moving the foot/toes, or you can’t feel a pulse. A sudden drop in blood flow to a limb, called acute limb ischemia, is a medical emergency.

If this has been coming on gradually, or the pain reliably happens when walking and eases with rest, book a medical appointment soon. A clinician can check pulses and may do an ankle-brachial index test to compare blood pressure in your ankle and arm. PAD matters because it can be linked with higher risk of heart attack and stroke, and treatment can reduce risk and improve walking symptoms.

Other things can also cause similar symptoms — nerve problems, cold exposure/Raynaud’s, injury, blood clots, infection, or diabetes-related foot issues — but the combination you described is worth getting checked.

DeepSeek-V4:

Yes, the combination of a foot that feels cold, changes color (darker), and hurts when you walk is a classic set of warning signs for poor circulation, specifically a problem with arterial blood flow. This is most commonly caused by Peripheral Artery Disease (PAD).

Here’s a breakdown of why those symptoms fit, what they mean, and—most importantly—why you need to see a doctor promptly.

Why These Symptoms Point to Poor Circulation

Think of your arteries as pipelines delivering oxygen-rich blood to your leg and foot. When those pipelines narrow (often due to plaque buildup), the tissues don’t get enough oxygen, especially when demand increases.

Pain When Walking (Claudication): This is the hallmark. Muscles need extra oxygen during activity. When arteries are too narrow to deliver that surge of blood, the muscles cramp up, causing aching, burning, or pain in the calf, thigh, or buttock. It typically goes away with rest and returns predictably with the same amount of walking.
 Cold Foot: Blood carries warmth. Reduced arterial inflow means less heat is delivered to the foot, causing it to feel persistently cold compared to the other foot or the rest of your body.
 Darker Color Change: This can mean a couple of things depending on the position:
 Elevation Pallor: If you lie down and lift the leg, the foot might turn very pale or white because gravity makes it even harder for weak blood flow to reach it.
 Dependent Rubor: When you sit up and dangle the leg, the foot may turn a dusky, dark red or purplish color. This happens because the tiny blood vessels are desperately dilating to pull in whatever oxygen is left in the sluggish blood flow. This red/dark color is not healthy circulation—it’s a sign of severe oxygen debt.

Other Possible (but Less Likely) Causes

While poor circulation from PAD is the primary concern, other conditions can mimic or contribute to these symptoms:
 Neuropathy (Nerve Damage): Often from diabetes, can cause burning or shooting pain, but doesn't usually cause the foot to be objectively cold or change color dramatically.
 Venous Insufficiency: Problems with the veins returning blood can cause a heavy, aching pain and darkish discoloration (from blood pooling), but the foot is usually warm, not cold.
 Raynaud’s Phenomenon: Causes cold and color changes (white, blue, red) in the toes, triggered by cold or stress, but it’s episodic and usually doesn’t cause pain with walking.

What To Do Now

You should make an appointment with your doctor as soon as possible. Do not dismiss these symptoms. Untreated PAD can progress to:
 Rest Pain: Severe burning or aching in the forefoot or toes, especially at night when lying down, relieved only by dangling the foot.
 Critical Limb Ischemia: The most advanced stage, where blood flow is so poor that ulcers, non-healing wounds, gangrene, and ultimately amputation can occur.

PAD is also a major red flag that similar blockages may exist in the arteries of your heart or brain, increasing the risk of heart attack and stroke.

While you wait for your appointment:
 Do not use heating pads or hot water bottles on the cold foot. Reduced sensation means you could burn yourself badly without realizing it.
 Check your feet daily for any cuts, blisters, or cracks, and keep them clean and moisturized. In poor circulation, even a tiny injury can become a serious, non-healing wound.
 Protect your feet with well-fitting shoes and socks.
 Stop smoking, if you do—it’s the single most powerful step to stop PAD from worsening.

What to Expect at the Doctor

The evaluation for PAD is simple and painless. The doctor will likely:
1. Take a history and examine your feet, checking the pulses on the top of your foot and behind your ankle. Absent or very weak pulses are a key sign.
2. Perform an Ankle-Brachial Index (ABI): This quick, non-invasive test compares the blood pressure in your ankle to the blood pressure in your arm. A ratio lower than 0.90 strongly indicates a blockage.

The symptoms you’re describing are significant and warrant a professional medical evaluation to understand the severity and start treatment to protect your mobility and your life. Please seek care.

Gemini 3.1 Pro:

Yes, the symptoms you are describing—a foot that is cold, darker in color, and painful when walking—are classic signs of poor blood circulation. Specifically, these are common symptoms of a condition known as Peripheral Artery Disease (PAD).

Understanding Your Symptoms

Here is a breakdown of what these specific signs often indicate:

Pain When Walking: This is medically known as intermittent claudication. It happens because your muscles demand more oxygen-rich blood during activity. If the arteries supplying your legs and feet are narrowed or blocked, the muscles cannot get enough oxygen, leading to cramping, aching, or sharp pain that typically subsides when you rest.
 Coldness: A noticeable difference in temperature between one foot and the other (or compared to the rest of your body) occurs when warm blood flow to the extremity is significantly restricted.
 Darker Color: A foot that turns darker, unusually pale, blue, or purplish (a condition called cyanosis) indicates that the tissues are actively being deprived of oxygenated blood.

Potential Causes

The most common culprit for these symptoms is atherosclerosis, which is a buildup of fatty plaques (cholesterol) inside your arteries that progressively restricts blood flow to your lower extremities. Other potential causes could include an acute blood clot (which can block an artery suddenly) or small vessel disease. However, the specific combination of temperature change, color change, and pain upon exertion strongly points toward a significant vascular issue.

What You Should Do Next

Because these symptoms indicate that the tissues in your foot are struggling to get enough oxygen to survive and function properly, this requires prompt medical evaluation.

Seek Medical Care: Schedule an appointment with a primary care physician or a vascular specialist as soon as possible.
 Watch for Red Flags: If the pain begins to happen even when you are resting (known as "rest pain"), if the foot goes completely numb, or if you develop open sores or ulcers that will not heal, this can indicate Critical Limb Ischemia (CLI). This is a medical emergency that requires immediate care at a hospital to prevent permanent tissue damage.
 What to Expect at the Doctor: A physician will physically examine your foot, check your pulses, and likely perform a simple, non-invasive test called an Ankle-Brachial Index (ABI). This compares the blood pressure in your ankle to the pressure in your arm. They may also use an ultrasound to map the blood flow in your legs.

Disclaimer: I am an AI, not a doctor. Because severely restricted blood flow can lead to dangerous complications if left untreated, please consult a healthcare professional immediately to get a proper diagnosis and treatment plan.

Grok 4.3:

Yes, these symptoms—cold feet, darker or discolored skin, and pain when walking—can indicate poor circulation, often due to peripheral artery disease (PAD) or related vascular issues. However, I am not a doctor, and this is not a diagnosis or medical advice. These symptoms require prompt professional evaluation, as they can signal serious conditions that need testing and treatment. Self-diagnosis or delay can lead to complications like non-healing wounds or worse.

Why These Symptoms Suggest Poor Circulation (Especially PAD)
Poor circulation (reduced blood flow to the legs and feet) commonly stems from atherosclerosis (plaque buildup narrowing arteries). Key matches to your description include:

Cold feet or legs (especially one colder than the other): Reduced blood flow fails to deliver warmth and oxygen. This is a frequent early sign.

Darker or discolored skin: In PAD, skin can appear pale, blue, bluish-purple, or dark due to insufficient oxygen (cyanosis or ischemia). It may also look shiny or change texture. "Darker" often aligns with bluish/dark tones from poor arterial flow.

Pain or cramping when walking (called intermittent claudication): Muscles (often calves, but also feet/thighs) hurt, burn, or feel heavy/fatigued during activity because they lack enough oxygenated blood. Pain typically eases within minutes of resting. This is the hallmark symptom of PAD.

These often occur together because narrowed arteries limit blood supply during increased demand (walking) and at rest in more advanced cases. Other signs can include numbness/tingling ("pins and needles"), slow-healing sores, hair loss on legs, or weak pulses in the feet.

Sources confirming this pattern: Mayo Clinic, Cleveland Clinic, MedlinePlus, and NHS descriptions of PAD symptoms consistently list coldness, color changes (pale/blue/dark), and activity-related pain as core indicators.

Other Possible Causes
Not every case is PAD. Overlapping or alternative explanations include:
 Chronic venous insufficiency (CVI) or varicose veins: More brownish discoloration (from blood pooling and iron deposits), swelling, heaviness, or aching (often worse standing, better elevating legs). Less likely to cause classic "pain exactly when walking that stops with rest."
 Raynaud’s phenomenon: Episodic color changes (white → blue → red) triggered by cold or stress, with cold/numb/painful fingers or toes—but usually not tied to walking distance.
 Neuropathy (e.g., from diabetes): Burning, tingling, or numbness; feet may feel cold subjectively even if not actually cool.
 Other: Hypothyroidism, anemia, low body weight, medications, anxiety, or musculoskeletal issues (e.g., spinal stenosis causing leg pain on walking).

A combination of cold + darker color + walking pain raises stronger concern for arterial circulation problems.

When to Seek Care (Important)
See a doctor promptly (primary care, vascular specialist, or podiatrist for foot-focused issues):
 Symptoms are new, worsening, or affecting daily life.
 One foot is noticeably different (asymmetric).
 You have risk factors: smoking, diabetes, high blood pressure/cholesterol, age >50–60, obesity, family history of vascular disease, or sedentary lifestyle.

Seek emergency care (ER or call emergency services) if:
 Sudden severe pain, cold/pale/blue foot with weakness or numbness.
 Pain at rest, especially at night (relieved by dangling leg).
 Open sores/ulcers that won’t heal, signs of infection (redness, warmth, pus, fever), or tissue changes (blackening/gangrene risk).
 Loss of sensation or inability to walk.

Early evaluation prevents progression to critical limb ischemia (severe blockage risking tissue death/amputation) or signals broader issues like heart disease risk.

What to Expect at the Doctor
They will review history/risk factors, examine pulses/skin/temperature, and likely order:
 Ankle-brachial index (ABI): Simple, painless comparison of arm vs. ankle blood pressure.
 Ultrasound (Doppler), imaging, or blood tests.

Treatment often starts with lifestyle (quit smoking—biggest impact—supervised walking program, heart-healthy diet, weight management, blood sugar/BP/cholesterol control) plus medications (statins, antiplatelets, etc.). Advanced cases may need procedures like angioplasty or bypass.

While awaiting your appointment: Keep feet warm and dry (layers, not tight socks/shoes), check feet daily for cuts (especially if diabetic), avoid prolonged sitting/standing, and walk gently if tolerable (stop at pain). Do not ignore symptoms or use unproven remedies.

Please consult a healthcare professional soon—they can pinpoint the exact cause with proper exams and get you on the right path. If symptoms worsen rapidly, don’t wait. Take care.

Qwen3.6-Max-Preview:

Yes, those symptoms can strongly suggest poor circulation, particularly reduced arterial blood flow to your lower extremities. The combination you're describing aligns closely with a condition called peripheral artery disease (PAD), though other vascular or nerve-related issues could also be involved.

What Your Symptoms May Mean:
 Cold foot: Reduced warm arterial blood reaching the area.
 Darker discoloration (bluish, purplish, brownish, or pale): Can indicate chronic low oxygen delivery, blood pooling, or early tissue changes from poor perfusion.
 Pain when walking that eases with rest: Classic sign of intermittent claudication. Your leg muscles aren't getting enough oxygen during activity due to narrowed or blocked arteries.

When to Seek Immediate Care:
Go to an urgent care clinic or emergency department right away if you experience any of the following:
 Sudden, severe pain, numbness, or weakness in the foot/leg
 Foot or toes turning black, very blue, or extremely pale
 Loss of a pulse in the foot or ankle
 Open sores, ulcers, or skin that looks shiny, tight, or feels cold/hard
 Inability to move the foot or bear weight

These can signal acute limb ischemia or advanced tissue damage, which requires emergency treatment to prevent permanent injury.

What to Expect at a Medical Visit:
A clinician will likely:
 Check pulses in your feet and compare blood pressure in your arms vs. ankles (ankle-brachial index, or ABI test)
 Review risk factors: smoking, diabetes, high blood pressure, high cholesterol, age over 50, family history, or sedentary lifestyle
 Order a Doppler ultrasound or other vascular imaging if needed Discuss treatment options, which may include lifestyle changes, medications (like antiplatelets or cholesterol-lowering drugs), supervised exercise therapy, or in some cases, minimally invasive procedures to restore blood flow

While You Wait to Be Evaluated:
 Stop smoking completely if you smoke (it's the single most impactful step for circulation)
 Keep feet warm and protected, but avoid heating pads or hot water (reduced blood flow + possible nerve changes = high burn risk)
 Rest when walking pain occurs, but don't ignore it or delay evaluation
 Check your feet daily for cuts, blisters, swelling, or color changes
 Wear well-fitting, supportive shoes and avoid walking barefoot

Important Note:
This information is educational and not a substitute for a professional medical evaluation. Given your symptoms, please schedule an appointment with a primary care doctor or vascular specialist promptly. Early diagnosis and management can prevent complications, improve circulation, and help you stay active safely.

If you'd like, I can help you prepare questions for your visit or explain common tests/treatments in more detail.

Q15. My foot wound is not healing. Why do doctors need to check my blood vessels?

GPT-5.5 Thinking:

Doctors check your blood vessels because a foot wound cannot heal well unless enough blood reaches the skin and deeper tissue. Blood brings oxygen, nutrients, immune cells, and antibiotics to the wound. If arteries in the leg or foot are narrowed or blocked, the wound may stay open, get infected, or worsen even with good dressings. This is often due to peripheral artery disease, and it is a major reason foot wounds—especially diabetic foot ulcers—do not heal.

They are usually looking for:

1. Poor arterial blood flow
Blocked or narrowed arteries reduce healing capacity. In severe cases, this can become chronic limb-threatening ischemia, where a wound, gangrene, or rest pain occurs because the limb is not getting enough blood. Current PAD guidance emphasizes early identification and treatment of wounds to reduce tissue loss and amputation risk.

2. Whether the wound has a realistic chance to heal on its own
Tests such as checking foot pulses, an ankle-brachial index, toe pressure, Doppler ultrasound, or other vascular imaging help estimate whether enough blood is reaching the wound. If blood flow is too low, the wound may not heal until circulation is improved.

3. Whether you need a vascular specialist
If circulation is poor, doctors may consider treatments to restore blood flow, such as angioplasty, stenting, bypass surgery, or other vascular procedures. The goal is to give the wound enough blood supply to heal and to prevent infection, tissue death, or amputation.

4. Other risks that slow healing
Diabetes, smoking, high blood sugar, nerve damage, kidney disease, high blood pressure, foot deformity, infection, and pressure from shoes can all contribute. Mayo Clinic notes that poor blood flow and nerve damage can make foot sores worsen quickly, and non-healing foot ulcers need prompt care.

Please seek urgent medical care now if the wound is spreading, black/blue, very painful, foul-smelling, draining pus, surrounded by increasing redness or warmth, you have fever/chills, red streaks, new numbness, or your foot feels cold or looks pale. Those can signal infection or dangerously reduced blood flow.

DeepSeek-V4:

It’s completely understandable to wonder why doctors focus on your blood vessels when the visible problem is a wound on your foot. The short answer is: blood flow is the single most important factor in wound healing.

Without a healthy, robust blood supply, even the best wound care will fail. Here’s the deeper explanation of why this check is so critical.

1. Your Blood Delivers the “Building Materials” for Healing
Think of a wound like a construction site. To rebuild tissue, you need a constant delivery of raw materials and a way to haul away debris. Your bloodstream provides both:

Oxygen and Nutrients: Healing cells (like fibroblasts) need energy to build new collagen and skin. This energy comes from oxygen and nutrients carried by the blood.
 Immune Cells and Growth Factors: White blood cells travel through the blood to fight infection, and specialized proteins called growth factors signal the body to start the repair process.
 Waste Removal: Blood flow also carries away dead cells, bacteria, and other debris from the wound site.

If the “roads” (your arteries) are blocked or narrowed, the construction site shuts down. The tissue literally starves.

2. Foot Wounds Are a Red Flag for Poor Circulation
Non-healing foot wounds are a classic and serious sign of Peripheral Arterial Disease (PAD) . PAD is a condition where fatty deposits (plaque) build up in the arteries of your legs, narrowing them and severely reducing blood flow to your feet. This is the same process that causes heart attacks, just happening in your legs.

When you have PAD:
 A minor cut or blister doesn’t get the surge of healing blood it needs.
 The skin’s baseline health is already compromised due to chronic low oxygen.
 The wound stalls out, often taking on a pale, gray, or “punched-out” look instead of healthy pink healing tissue.

3. Without Blood Flow, Infection Risk Skyrockets
This is the most urgent reason. A poorly perfused wound cannot fight off bacteria. Even a small infection can quickly spiral out of control because:
 Antibiotics taken by mouth or intravenously cannot reach the wound in sufficient concentration if the arteries are blocked.
 The immune system’s first responders (white blood cells) can’t get to the battlefield.

This is how a simple foot wound in someone with undiagnosed vascular disease can lead to gangrene and, in the worst-case scenario, amputation. Checking your vessels is not just about healing the wound—it’s about saving the limb.

4. It’s a Common Dance Partner with Diabetes and Nerve Damage
If you have diabetes, this connection is even more critical. Diabetes often causes both neuropathy (nerve damage, so you don’t feel the wound getting worse) and PAD. A foot wound in a person with diabetes is called a “diabetic foot ulcer,” and vascular assessment is a non-negotiable first step in standard care worldwide.

What Doctors Are Checking For
You asked why they need to check, but you might also be wondering how. The checks are simple and non-invasive:

1. Foot Pulses: They’ll feel for pulses on the top of your foot and behind your inner ankle bone. Absent or weak pulses are a direct clue.
2. Skin and Temperature: They’ll look for signs of poor blood flow, like shiny, hairless skin, thick toenails, and a cold foot.
3. Ankle-Brachial Index (ABI): This is a painless test comparing blood pressure in your ankle to your arm. A low number signals a blockage.
4. Doppler Ultrasound: A handheld device that lets them actually hear your blood flow. It’s like the scanner used in pregnancy, but for your arteries.
5. Vascular Imaging (if needed): A more detailed ultrasound, CT angiogram, or traditional angiogram to map the exact location and severity of a blockage.

If a significant blockage is found, improving that blood flow—often with a minimally invasive procedure (angioplasty/stent) or bypass surgery—becomes the top priority before aggressive wound treatment can succeed.

In summary, your physician isn’t just looking at the wound; they are looking for the underlying reason why your body’s repair crew hasn’t arrived. Determining the health of your blood vessels is the most critical piece of that puzzle. It is a very wise and necessary step.

Gemini 3.1 Pro:

It can be incredibly frustrating and worrisome when a wound on your foot just won't heal. It is completely understandable to wonder why your doctors are focusing on your blood vessels when the problem seems to be right there on your skin.

The short answer is that healing happens from the inside out, and your blood vessels are the delivery system that makes that healing possible.

Here is a breakdown of why checking your vascular health is a standard and crucial step in treating a non-healing foot wound:

1. Delivery of the "Building Blocks" for Repair

To fix damaged tissue, your body needs a constant supply of specific resources:

Oxygen: Cells require oxygen to generate the energy needed to rebuild tissue.
 Nutrients: Proteins, vitamins, and minerals act as the physical building blocks for new skin and muscle.
Your arteries act as highways, carrying these essential supplies from your heart down to your toes. If those blood vessels are narrowed or blocked (a condition often called Peripheral Artery Disease, or PAD), the foot simply doesn't receive enough raw materials to close the wound.

2. Fighting Off Infection

Open wounds are highly vulnerable to bacteria. Your immune system deploys white blood cells and antibodies to the site of the injury to destroy these bacteria and prevent an infection from taking hold. If blood flow to your foot is poor, these immune cells cannot reach the wound in sufficient numbers, leaving it defenseless.

3. Clearing Away the Debris

Just like a construction site, repairing a wound generates waste. Dead cells, broken-down tissues, and destroyed bacteria need to be hauled away so that new, healthy tissue can grow. Your veins carry this cellular debris away from the wound. If circulation is sluggish, this waste builds up and stalls the healing process.

What the Doctors Are Looking For

When doctors check your blood vessels, they are essentially checking the "plumbing" to make sure the pipes are open. They might do this by:

Feeling for pulses in your feet and ankles.
 Using an ultrasound (like a Doppler) to listen to the blood flow.
 Measuring blood pressure in your ankles compared to your arms (Ankle-Brachial Index or ABI).

If they find that the blood flow is restricted, their first priority will often be to improve that circulation. Once the blood is flowing properly, the wound finally has the supplies it needs to heal itself.

Grok 4.3:

I'm not a doctor, and this is not personalized medical advice. Non-healing foot wounds can signal serious underlying issues and carry risks like infection or tissue loss. Please consult your physician, wound care specialist, podiatrist, or a vascular expert promptly for evaluation and tailored care. Early intervention often improves outcomes dramatically.

Why Blood Vessel (Vascular) Assessment Matters for Foot Wounds
Wound healing requires adequate blood flow (perfusion) to deliver oxygen, nutrients, growth factors, and immune cells to the injury site while removing waste and fighting infection. Without sufficient circulation, the wound stalls in the inflammatory phase and cannot progress to tissue repair and closure.

In the feet and lower legs, this is especially critical because:
 These areas are farthest from the heart, making them vulnerable to reduced flow.
 Common conditions like peripheral artery disease (PAD) — caused by plaque buildup narrowing arteries — or diabetes-related vascular damage frequently impair circulation here.
 Roughly 50% of diabetic foot ulcers involve PAD, which significantly raises risks of non-healing, infection, gangrene, and amputation.

Even small wounds (from blisters, pressure, or unnoticed trauma due to neuropathy) can become limb-threatening if blood supply is poor. Doctors must rule out or quantify ischemia (inadequate blood flow) because standard wound care — cleaning, dressings, offloading pressure, antibiotics, or debridement — will fail or worsen outcomes without restoring perfusion first.

What Vascular Checks Reveal and Why They're Done
Doctors assess vessels to:
 Confirm whether poor circulation is the primary barrier (or a major contributor alongside neuropathy, infection, or pressure).
 Measure severity using objective tests, as symptoms like cool skin, hair loss, weak/absent pulses, or pale/blue discoloration can be subtle or masked (especially in diabetes with vessel calcification).
 Guide urgent decisions: If critical limb ischemia exists, revascularization may be needed within weeks for limb salvage.

Common assessments include:
 Clinical exam (pulse palpation, capillary refill, skin temperature/color, wound appearance).
 Ankle-brachial index (ABI) — a quick, non-invasive comparison of arm vs. ankle blood pressure (low ABI indicates arterial blockage).
 Doppler ultrasound (arterial waveforms, toe pressures).
 Advanced imaging like CT/MR angiography or transcutaneous oxygen pressure (TcPO₂) if needed.
 In diabetic foot ulcers, guidelines (e.g., from IWGDF, SVS) strongly recommend vascular evaluation because pedal pulses alone can miss significant disease.

If ischemia is confirmed, options may include lifestyle changes (smoking cessation, supervised exercise, cholesterol/blood pressure control), medications, or procedures like angioplasty, stenting, or bypass to reopen arteries and enable healing.

Other Contributing Factors Doctors Also Address
Vascular issues often coexist with:
 Neuropathy (loss of sensation leading to unnoticed injury).
 Infection or osteomyelitis (bone infection — poor flow limits antibiotic delivery).
 Uncontrolled blood sugar, poor nutrition, smoking, or ongoing pressure/shear.

A multidisciplinary team approach (wound care, vascular surgery, endocrinology, podiatry) is standard for best results.

Bottom line: Checking blood vessels isn't optional — it's foundational. Many non-healing foot wounds improve dramatically once circulation is optimized. Don't delay evaluation; persistent wounds (especially >2–4 weeks) warrant specialist input to protect your limb and overall health. Follow up with your care team, and feel free to share more details from your appointments if you'd like general explanations of test results or next steps. Take care.

Qwen3.6-Max-Preview:

When a foot wound isn't healing, checking your blood vessels is often one of the most important steps a doctor will take. Here's why:

Blood Flow Is Essential for Healing
Wound repair depends on a steady supply of oxygen, nutrients, immune cells, and growth factors. All of these are delivered through your bloodstream. If circulation to your foot is reduced, the tissue simply doesn't get what it needs to rebuild, fight infection, or close the wound.

Common Vascular Issues That Delay Healing
 Peripheral Artery Disease (PAD): Plaque buildup narrows the arteries, reducing blood flow to the legs and feet.
 Diabetes-Related Vascular Damage: High blood sugar over time can stiffen and narrow blood vessels, while also damaging nerves (which can mask pain and lead to unnoticed injuries).
 Venous Insufficiency: Faulty valves in leg veins cause blood to pool, leading to swelling, skin changes, and ulcers that heal poorly.
 Other Factors: Smoking, high blood pressure, high cholesterol, and chronic kidney disease all impair circulation and wound healing.

How Doctors Check Your Blood Vessels
 Physical exam: Feeling for pulses in your feet/ankles, checking skin temperature, color, and capillary refill.
 Ankle-Brachial Index (ABI): Compares blood pressure in your arm vs. your ankle to screen for PAD.
 Doppler ultrasound: Uses sound waves to visualize blood flow and detect blockages or valve problems.
 Advanced imaging (if needed): CT/MR angiography or traditional angiography to map out exact locations of narrowing or blockages.

Why This Changes Your Treatment
If poor circulation is found, wound care alone often isn't enough. Your doctor may:
 Refer you to a vascular specialist for procedures like angioplasty, stenting, or bypass to restore flow
 Adjust medications (e.g., antiplatelets, statins, blood pressure/diabetes control)
 Recommend supervised exercise, smoking cessation, or compression therapy (if venous)
 Tailor wound care to your specific circulation pattern (arterial vs. venous wounds require very different approaches)

When to Seek Prompt Care
Non-healing foot wounds can quickly lead to serious infection or tissue loss, especially if you have diabetes, smoke, or notice:
 Cold, pale, or bluish feet
 Leg pain when walking that improves with rest
 Swelling, heaviness, or skin darkening around the wound
 Foul odor, increasing redness, warmth, or pus

Bottom line: Checking your blood vessels helps your doctor find out why the wound isn't healing and whether restoring circulation is the missing piece. Without adequate blood flow, even the best dressings and antibiotics may fall short.

Disclaimer: This information is educational and not a substitute for professional medical advice. Please see a healthcare provider or wound care specialist for an accurate evaluation and personalized treatment plan.

Q16. When should I see a blood-flow specialist for a diabetic foot wound?

GPT-5.5 Thinking:

You should contact a clinician the same day for any diabetic foot wound, and you should see a blood-flow specialist—usually a vascular surgeon or vascular medicine specialist—urgently if there are signs the wound may not be getting enough circulation.

Go to urgent care / ER now if the wound has any of these: fever, chills, feeling very unwell, rapidly spreading redness or swelling, pus/foul odor, black or blue tissue, gangrene, a cold/pale/numb foot, severe pain, exposed bone/tendon, or concern for a deep infection. NICE recommends immediate acute referral for diabetic foot ulceration with fever/sepsis, limb ischemia, deep soft-tissue/bone infection, or gangrene. IDSA/IWGDF similarly recommends urgent surgical consultation for severe infection, gangrene, necrotizing infection, deep abscess, compartment syndrome, or severe lower-limb ischemia.

Ask for urgent vascular evaluation if you have a diabetic foot ulcer plus signs of poor blood flow: weak/absent foot pulses, a cold foot, color change, worsening pain, nonhealing wound edges, or known peripheral artery disease. IWGDF recommends assessing pulses and ischemia in anyone with diabetes and a foot ulcer or gangrene, and using Doppler plus ABI/TBI testing to identify PAD.

Do not wait more than a few weeks if it is not clearly improving. IWGDF says to reassess vascular status and consult a vascular specialist when a diabetic foot ulcer deteriorates or fails to significantly improve—example: less than 50% wound-area reduction within 4 weeks despite appropriate infection control, glucose control, wound care, and offloading.

Bottom line: for a diabetic foot wound, it is safer to be evaluated early. A vascular specialist is especially important if the wound is infected, black/gangrenous, painful, cold, numb, discolored, has weak pulses, or is not healing as expected.

DeepSeek-V4:

This is a crucial question because the healing of any wound depends on good blood flow. For a diabetic foot wound, a “blood-flow specialist”—usually a vascular surgeon, interventional cardiologist, or interventional radiologist—becomes essential when there’s any sign that poor circulation is preventing healing.

Here are the key situations where you should see one urgently or very soon, along with what to look for. This is educational, not individual medical advice—always follow your own doctor’s guidance.

Urgent / Emergency Situations (Seek Immediate Care)

Go to an emergency department or call for emergency help if you have a diabetic foot wound and any of these appear:

Sudden severe pain, coldness, pale or bluish color, or loss of movement/feeling in the foot and toes — these can signal acute limb ischemia, a vascular emergency.
 A newly black or purplish toe, or spreading black/gangrene at the wound site.
 Severe rest pain in the foot, especially at night, that wakes you and only feels better when you hang the foot down.
 Signs of a life-threatening infection together with poor circulation (fever, chills, red streaks, widespread redness, and a foul-smelling wound). An infected wound without enough blood flow cannot clear the infection.

Soon / As Soon as Possible (Outpatient Referral)

Even without an emergency, you should see a vascular specialist promptly if:

The wound hasn’t started shrinking after 2–4 weeks of proper wound care — this is a major red flag for poor blood supply.
 No foot pulses can be felt by your doctor/nurse on the affected foot (absent dorsalis pedis and posterior tibial pulses).
 You have pain in the calf, thigh, or buttock when walking that goes away with a few minutes of rest (claudication). Many diabetic patients don’t feel typical claudication due to neuropathy, so any walking pain must be checked.
 Pain in the forefoot or toes at rest, especially when lying down, relieved by dangling the foot — this is called “rest pain” and indicates critical limb ischemia.
 Dependent rubor or pallor on elevation: The foot turns dusky red when hanging down and pale when lifted up; this shows severely damaged circulation.
 Known peripheral artery disease (PAD) and the wound doesn’t improve, or you have not had your circulation checked yet.
 Prior bypass or stenting that might be failing, with a new or non-healing wound.

What Tests a Specialist Might Use

A vascular specialist will typically start with a physical exam and simple tests like:
 Ankle-Brachial Index (ABI) – blood pressure at the ankle compared to the arm; extremely low values (<0.4) often mean wound healing won’t happen without improved flow.
 Toe pressures or TcPO2 – more reliable in diabetic patients with stiff arteries; toe pressure <30 mmHg or TcPO2 <25 mmHg indicates critical ischemia.
 Duplex ultrasound, CTA, or angiogram – to map blockages and plan procedures (angioplasty, stent, bypass).

Why Early Referral Matters

Many diabetic foot guidelines recommend that every person with a diabetic foot ulcer and an absent foot pulse should be referred to a vascular team. Early revascularization not only helps the wound heal, it can save the leg. Don’t wait until the wound worsens, turns black, or becomes infected—poor blood flow silently sabotages healing even when the wound looks clean.

Please remember: This information is meant to help you understand when to act, but every situation is unique. If you have a diabetic foot wound and aren’t sure about your blood flow, ask your primary care doctor, podiatrist, or wound care team today whether a vascular evaluation is right for you.

Gemini 3.1 Pro:

When managing diabetic complications, whether evaluating microvascular damage in the eye or macrovascular and neuropathic changes in the lower extremities, timely intervention is critical to preventing irreversible tissue loss.

For a diabetic foot wound, a vascular specialist (such as a vascular surgeon) should be consulted when there is evidence of peripheral artery disease (PAD) or when the wound fails to progress through the normal healing cascade despite standard care.

Here are the specific clinical thresholds and indicators for a referral:

1. Urgent "Red Flag" Indicators

Immediate evaluation by a vascular specialist is required if any of the following acute signs of critical limb ischemia (CLI) are present:

Tissue Necrosis or Gangrene: Any black, dead tissue on the toes, heel, or margins of the wound.
 Ischemic Rest Pain: Severe pain in the foot or toes that occurs at rest, particularly at night, and is sometimes relieved by hanging the foot over the edge of the bed (though severe neuropathy may mask this pain).
 Sudden Changes: The foot suddenly becomes cold, pale, cyanotic (blue/purple), or pulseless.

2. Clinical Signs of Poor Perfusion

A vascular assessment is warranted if routine clinical examination reveals compromised blood flow:

Absent Pulses: Inability to palpate the dorsalis pedis or posterior tibial pulses.
 Abnormal Skin Characteristics: Pale, shiny, or excessively dry skin, loss of hair on the toes and lower legs, or a foot that feels noticeably cooler than the rest of the body.
 Prolonged Capillary Refill: Pressing on the toe pad and taking longer than 3 seconds for the pink color to return.

3. The "Non-Healing" Threshold

Even if obvious signs of severe ischemia are absent, a vascular consultation is the standard of care if the wound is stagnant for 2 to 4 weeks.
If a diabetic foot ulcer shows no significant reduction in size or depth within this timeframe—despite optimal wound care, strict offloading (removing pressure from the area), and infection control—underlying vascular insufficiency must be ruled out as the limiting factor.

4. Abnormal Objective Hemodynamic Metrics

If basic non-invasive vascular tests have been performed in a primary or podiatric setting and yield abnormal results, a specialist should interpret and act on them. Key metrics include:

Ankle-Brachial Index (ABI): An ABI of < 0.9 indicates PAD. However, in diabetic patients, heavily calcified vessels can cause a falsely elevated ABI (> 1.3).
 Toe-Brachial Index (TBI): Because toe arteries are less prone to calcification, a TBI is often used. A TBI < 0.7 generally indicates vascular disease, and < 0.25 is associated with severe ischemia.
 Abnormal Doppler Waveforms: Monophasic or absent waveforms in the pedal arteries.

The Standardized Approach (SVS WIfI)

In clinical literature and practice, vascular surgeons often use the Society for Vascular Surgery (SVS) WIfI classification system (Wound, Ischemia, and foot Infection). This standardized tool stratifies the one-year risk of amputation and the likelihood of benefiting from revascularization. If a primary care physician or podiatrist determines a patient has a measurable "Ischemia" score on this index alongside a wound, vascular referral is indicated to discuss interventions like angioplasty, stenting, or bypass surgery.

Disclaimer: This information is for educational purposes. Any rapidly deteriorating wound or suspected infection in a diabetic foot should be evaluated by a healthcare professional immediately.

Grok 4.3:

See a blood-flow specialist (typically a vascular surgeon or vascular medicine specialist) promptly for a diabetic foot wound when there is evidence or high suspicion of poor circulation (peripheral artery disease, or PAD), which impairs healing and raises risks of infection, gangrene, or amputation. Diabetes often masks classic PAD symptoms due to neuropathy, and up to half of diabetic foot ulcers (DFUs) involve an ischemic component.

This is not personalized medical advice. Diabetic foot wounds are medical emergencies that can worsen rapidly. Contact your podiatrist, wound care specialist, or primary care provider immediately for any break in the skin, ulcer, redness, swelling, or drainage on your foot. They will coordinate care, often with a multidisciplinary team (endocrinologist, podiatrist, infectious disease, and vascular experts). Early involvement of vascular specialists improves limb salvage rates significantly.

Key Guidelines and Triggers for Referral
Major guidelines (IWGDF 2023 PAD guidelines, Society for Vascular Surgery/SVS recommendations, and ADA Standards of Care) emphasize routine vascular assessment for all new DFUs and specific triggers for specialist referral:

Initial vascular testing for every DFU — Measure foot perfusion with ankle-brachial index (ABI), pedal Doppler arterial waveforms, and toe pressure (or transcutaneous oxygen pressure, TcPO₂). Do this early (ideally at first presentation) and repeat if the wound stalls. Abnormal results (e.g., ABI <0.9, monophasic/absent waveforms, low toe pressure) warrant prompt vascular referral for full evaluation, WIfI staging (Wound, Ischemia, foot Infection classification), and limb-salvage planning.

Non-healing or stalled wound — If the ulcer does not reduce in area by at least 50% after 4 weeks (or shows deterioration) despite optimal care—including offloading (e.g., total contact cast), debridement, infection control, and good glycemic control—reassess vascular status and refer to a vascular specialist for possible revascularization (endovascular procedures like angioplasty/stenting or surgical bypass).

Signs or symptoms of ischemia/PAD (even without a large wound):
 Absent or weak pedal pulses.
 Cool feet, hair loss on legs/feet, shiny or thin skin, dependent rubor (redness when hanging foot down), or pallor on elevation.
 Leg pain with walking (claudication) relieved by rest.
 Rest pain (especially at night, relieved by dangling the foot).
 Black tissue, necrosis, or gangrene.

Urgent or emergent vascular consult (same-day or within hours):
 Severe ischemia: ABI <0.4, ankle pressure <50 mmHg, toe pressure <30 mmHg, TcPO₂ <30 mmHg, or absent/monophasic Doppler waveforms.
 Infection + PAD + ulcer/gangrene (any foot involvement): Urgent surgical drainage consult plus vascular input for revascularization timing to prevent sepsis or major tissue loss.
 Rapidly worsening wound, spreading infection, or critical limb ischemia signs.

Why Early Referral Matters
Poor blood flow starves the wound of oxygen and nutrients, slowing healing and allowing infection to flourish. Revascularization (when appropriate) restores inline flow to the foot and dramatically improves outcomes. Vascular experts are uniquely equipped to interpret tests, perform all revascularization options, and integrate care with the rest of the team. Delaying referral until obvious failure increases amputation risk.

What to Expect and Next Steps
1. See your foot specialist today — Get the wound assessed, cultured if infected, imaged (X-ray/MRI for bone involvement), and offloaded immediately. They perform or order initial vascular tests.
2. Vascular evaluation — May include advanced imaging (duplex ultrasound, CT/MR angiography, or catheter angiogram) and WIfI staging to decide on observation, medical optimization, or intervention.
3. Multidisciplinary limb preservation program — Ideal setting; many centers have dedicated teams to coordinate everything and prevent amputation.
4. Supportive steps while awaiting care — Keep blood sugar tightly controlled, do not smoke, elevate the foot appropriately, protect from trauma, and follow all offloading instructions strictly. Never walk on an ulcer.

Red flags requiring emergency care (ER or urgent specialist): Fever, increasing pain/redness/swelling, pus/odor, black eschar, numbness worsening, or inability to bear weight.

Guidelines stress that no one should assume a wound is “just neuropathy”—vascular disease must be ruled out objectively in every DFU. Outcomes are far better with proactive collaboration between your wound care team and a blood-flow specialist. Act fast—many amputations are preventable with timely intervention. Consult your healthcare providers right away for evaluation tailored to your situation.

Qwen3.6-Max-Preview:

You should see a blood-flow specialist (typically a vascular surgeon or interventional radiologist/cardiologist) for a diabetic foot wound early and proactively, especially if any of the following apply:

When to Refer or Request a Vascular Evaluation
1. No improvement after 2–4 weeks of appropriate wound care, offloading, and infection management.
2. Signs of poor circulation in the foot/leg:
 Cold, pale, bluish, or mottled skin
 Weak or absent foot pulses
 Shiny, tight, or hairless skin on the lower leg/foot
 Calf, foot, or thigh pain when walking that resolves with rest (claudication)
 Rest pain, especially at night or when elevating the foot
3. Abnormal perfusion tests (often done by your podiatrist or primary care provider):
 Ankle-brachial index (ABI) `< 0.9` or `> 1.3` (the latter suggests calcified, non-compressible arteries common in diabetes)
 Toe systolic pressure `< 30 mmHg`
 Transcutaneous oxygen (TcPO₂) `< 25 mmHg`
4. High-risk wound features:
 Deep ulcer, exposed bone/tendon, or probing to bone
 Black, dead, or leathery tissue (necrosis/gangrene)
 Rapidly expanding wound or worsening despite care
5. Relevant medical history:
 Known peripheral artery disease (PAD), coronary artery disease, stroke, or prior leg/foot revascularization or amputation
 Long-standing diabetes, kidney disease, or current/former smoking

Seek Urgent/Emergency Care If You Develop:
 Spreading redness, warmth, swelling, foul odor, or pus
 Fever, chills, or confusion
 Sudden color/temperature change in the foot
 Severe, unrelenting rest pain
These can signal limb-threatening infection or critical limb ischemia, which require immediate hospital evaluation.

Why Blood Flow Matters
Diabetic foot wounds heal poorly when circulation is compromised. Adequate perfusion delivers oxygen, immune cells, nutrients, and systemic antibiotics to the tissue. Vascular specialists can:
 Perform advanced imaging (duplex ultrasound, CTA, MRA)
 Restore flow with minimally invasive procedures (angioplasty, stenting, atherectomy) or bypass surgery
 Coordinate with wound care, podiatry, and infectious disease teams to salvage the limb

Practical Next Steps
 Don't wait for a wound to "get worse." Guidelines from the International Working Group on the Diabetic Foot (IWGDF) and ADA recommend early vascular assessment for any diabetic foot ulcer that isn't clearly healing or shows signs of ischemia.
 Ask your primary care doctor, endocrinologist, or podiatrist for a vascular surgery referral. Many centers offer multidisciplinary diabetic foot clinics that streamline this process.

Disclaimer: This information is educational and not a substitute for professional medical advice. Diabetic foot wounds can deteriorate quickly. Always consult a licensed healthcare provider for personalized evaluation and management.

Q17. What should be done to help my diabetic foot ulcer heal?

GPT-5.5 Thinking:

A diabetic foot ulcer should be managed with a clinician or wound-care/podiatry team, not treated only at home. The main things that help it heal are:

1. Get pressure off the ulcer (“offloading”)
 This is often one of the most important steps. Your clinician may use a special shoe, removable boot, brace, cast, crutches, or wheelchair. Even brief walking on the ulcer can undo healing progress.

2. Professional wound care and debridement
 Dead skin/tissue may need to be removed by a podiatrist or wound-care clinician. Do not cut or scrape it yourself.

3. Keep it clean, covered, and dressed correctly
 Follow the dressing plan you were given. In general, the ulcer should be kept clean and bandaged, and the dressing/skin around it should not stay overly wet.

4. Check for infection and treat it promptly
 Call your clinician urgently for redness, warmth, swelling, increased drainage, pus, odor, fever/chills, increased pain, or firmness around the wound; also call if the ulcer looks white, blue, or black. The IWGDF/IDSA guideline says diabetic foot infection is diagnosed clinically by local or systemic inflammation, and severe infections—or moderate infections with important complicating conditions—may need hospitalization.

5. Assess blood flow
 Poor circulation can prevent healing. Your clinician should check foot pulses and may order circulation testing; if healing is poor and circulation is reduced, referral to a vascular specialist may be needed.

6. Improve diabetes and vascular risk control
 Keeping blood glucose in range helps wounds heal and helps the body fight infection. Blood pressure, cholesterol control, and stopping smoking also matter.

7. Protect the rest of the foot every day
 Check both feet daily, including between toes; wash and dry carefully; wear well-fitting shoes and socks; do not walk barefoot; do not soak the foot unless your clinician specifically instructs it.

Seek same-day urgent care or ER care if there is fever, chills, rapidly spreading redness, black/blue tissue, foul odor, pus, severe swelling, new numbness/weakness, exposed bone/tendon, or you feel ill. Diabetes-related foot ulcers can be painless but still serious because nerve damage and poor blood flow can hide injury and slow healing.

DeepSeek-V4:

Disclaimer: I am an AI, not a doctor. The following information is for educational purposes only and does not replace professional medical advice. Diabetic foot ulcers are serious and can lead to severe infection, hospitalization, or amputation if not treated correctly. You need to be under the active care of a healthcare professional immediately.

Healing a diabetic foot ulcer requires a multi-pronged, medically supervised approach. It’s not just about the wound itself; it’s about managing your whole body. Here are the critical components that your healthcare team will likely address:

1. The Most Critical Step: Offloading (Pressure Relief)
The single most important factor in healing is taking all pressure off the wound. Every step you take on the ulcer destroys new, fragile tissue. This is non-negotiable.

Total Contact Casting (TCC): This is the gold standard. A doctor applies a specialized cast that distributes weight across your entire lower leg and foot, allowing you to walk while completely protecting the ulcer.
 Removable Cast Walker / Walking Boot: A bulky orthopedic boot, often with rocker-bottom soles. The key here is that it must be non-removable when possible. Studies show if patients can take it off, they often do, significantly slowing healing. Your doctor might wrap it in a layer of fiberglass to make it semi-permanent.
 Specialized Surgical Shoes or Sandals: For less severe ulcers or post-healing, but they must be professionally fitted.
 Crutches, Knee Scooter, or Wheelchair: Sometimes full non-weight-bearing is necessary.

What you must do: Do not walk on the ulcerated foot, even in "just a sock" or to go to the bathroom, without your prescribed offloading device.

2. Wound Care and Debridement
This must be performed by a medical professional, such as a podiatrist or wound care nurse.

Sharp Debridement: The doctor uses a scalpel to regularly remove dead skin, callus, and non-viable tissue from the wound and its edges. This is crucial because dead tissue is a breeding ground for bacteria and prevents new cells from growing. It often needs to be done weekly or bi-weekly.
 Cleaning the Wound: The wound will be cleaned with a gentle solution, like sterile saline. Avoid harsh antiseptics like hydrogen peroxide or iodine unless specifically instructed, as they can damage healing cells.
 Advanced Dressings: The dressing is chosen to create the perfect healing environment—moist but not wet. Options your doctor may use include:
 Hydrogels: For dry wounds to add moisture.
 Alginates or Foams: For wounds with a lot of drainage to absorb fluid.
 Collagen or Silver Dressings: To promote tissue growth or manage bacteria.

3. Infection Management
A foot ulcer is an open door for bacteria. Infection is a major threat.

Clinical Diagnosis: Not all wounds are infected. Signs include redness spreading around the wound, unusual warmth, increased pain, foul odor, and thick, yellow/green drainage.
 Deep Culture: If an infection is suspected, the doctor must perform a deep tissue culture (after cleaning and debriding). A simple surface swab is often inaccurate. This identifies the specific bacteria and the right antibiotic.
 Antibiotics: You may be prescribed oral or IV antibiotics. Take the full course exactly as prescribed, even if the wound looks better.

4. Vascular Assessment (Checking Blood Flow)
For a wound to heal, it needs oxygen and nutrients delivered by healthy arteries. Poor circulation is a primary reason ulcers won’t heal in people with diabetes.

Non-Invasive Vascular Tests: Your doctor will likely check the pulses in your feet and perform tests like an Ankle-Brachial Index (ABI) or a Toe-Brachial Index (TBI) using blood pressure cuffs and a Doppler ultrasound.
 Referral to a Vascular Surgeon: If blood flow is significantly reduced, you may need a procedure to open the arteries (angioplasty, stent, or bypass surgery) before the ulcer can heal. This is often the single most important intervention for a non-healing wound.

5. Blood Glucose Control
High blood sugar severely impairs the function of white blood cells (your immune system's soldiers) and damages your blood vessels.

Targets: Work closely with your endocrinologist or primary care doctor. Often, the goal is a pre-meal blood sugar of 80-130 mg/dL and a post-meal peak of less than 180 mg/dL, but your personal targets may vary.
 Hemoglobin A1c: This is a measure of your average blood sugar over the past 3 months. Lowering it, even modestly, can dramatically improve healing. Your doctor will likely want this checked.

6. Nutrition and General Health
Wound healing demands extra protein and calories.

Protein: Essential for building new tissue. Eat good sources like lean meats, fish, eggs, beans, and dairy. A dietitian can help you calculate your specific needs.
 Vitamins and Minerals: Deficiencies in Vitamin C, Vitamin D, and Zinc can impair healing. A blood test can check for these. Do not start high-dose supplements without your doctor’s advice.
 Hydration: Drink plenty of water.
 Quit Smoking: If you smoke, get help to stop immediately. Nicotine is a powerful vasoconstrictor that starves your extremities of blood. This is as critical as offloading.

A Daily Checklist for You
1. Absolutely no weight on the foot without your prescribed offloading device.
2. Inspect your feet daily (including the other foot), looking for any new redness, blisters, or cracks. Use a mirror if you can't see the bottom.
3. Check your blood sugar frequently and take all medications as prescribed.
4. Change the dressing exactly as instructed by your wound care team, maintaining a clean technique.
5. Never "bath" the foot by soaking it; this macerates the skin and increases infection risk.

Urgent Warning Signs – Go to the Emergency Department Immediately
If you experience any of the following, it’s a medical emergency:
 Redness spreading up your foot or leg.
 Unusual and increasing pain or throbbing.
 Fever, chills, or nausea.
 A sudden spike in your blood sugar for no apparent reason.
 A foul smell from the wound.
 A change in the wound color to black or green.

Please, take this seriously and work hand-in-hand with your podiatrist, endocrinologist, and vascular surgeon. You are the most important member of that team, and your daily actions will make the difference.

Gemini 3.1 Pro:

I am an AI, not a doctor, and a diabetic foot ulcer is a serious condition that requires prompt professional medical attention. To prevent severe complications like infection or amputation, you should see a physician or a podiatrist as soon as possible.

In a clinical setting, doctors typically focus on several key pillars to help a diabetic foot ulcer heal. Here is an overview of the standard medical approach:

1. Off-Loading (Taking Pressure Off)

This is often considered one of the most critical steps in healing. Any pressure or friction on the ulcer will prevent it from closing and can make it worse.

Specialized Footwear: Your doctor may prescribe a therapeutic shoe, a custom cast (like a total contact cast), a specialized boot, or a brace.
 Mobility Aids: You may be advised to use crutches, a walker, or a wheelchair to keep weight off the affected foot entirely.

2. Debridement (Removing Dead Tissue)

Wounds cannot heal if dead or infected tissue is in the way.

A healthcare professional will likely clean the wound and use medical tools to carefully remove the necrotic (dead) tissue. This helps stimulate the body's natural healing process and allows them to properly assess the depth of the ulcer.

3. Infection Management

Diabetic foot ulcers are highly susceptible to infection, which can spread to the bone if left untreated.

Testing: Your doctor may take a swab or tissue sample to determine if an infection is present.
 Medication: If an infection is diagnosed, you will likely be prescribed a course of antibiotics.
 Do not self-treat infections: Over-the-counter antibiotic creams are usually not sufficient for diabetic foot ulcers.

4. Proper Wound Care and Dressings

Keeping the wound clean and at the right moisture level is vital.

Dressings: Your healthcare provider will apply specific dressings tailored to your wound. These might include hydrogels, hydrocolloids, foam dressings, or dressings containing silver or iodine to manage bacteria.
 Moisture Balance: The goal is usually to keep the wound bed moist (which promotes healing) while keeping the surrounding skin dry.

5. Blood Sugar Control

Your body's ability to heal wounds and fight off infection is directly tied to your blood glucose levels.

Consistently high blood sugar stiffens the arteries and narrows blood vessels, restricting the blood flow and oxygen needed for tissue repair.
 Work closely with your endocrinologist or primary care doctor to ensure your blood sugar is strictly managed during the healing process.

6. Assessing and Improving Circulation

Healing requires a robust blood supply to deliver oxygen and nutrients to the wound.

Your doctor will likely check the pulses in your feet to ensure adequate blood flow.
 If you have peripheral artery disease (PAD) or poor circulation, you may be referred to a vascular specialist to explore ways to improve blood flow to your legs and feet.

Immediate Next Steps for You:
Please contact your healthcare provider or a wound care clinic immediately to schedule an evaluation. While you wait for your appointment, keep the area clean, keep your weight off that foot as much as possible, and do not attempt to cut away any tissue or use harsh chemicals on the wound yourself.

Grok 4.3:

Seek professional medical care immediately. Diabetic foot ulcers are a medical emergency because they can progress rapidly to deep infection, bone involvement (osteomyelitis), or amputation—especially with neuropathy (which often reduces pain sensation) and poor circulation common in diabetes. Do not attempt to self-treat, debride, or use unproven home remedies, as these can worsen outcomes. Contact your doctor, podiatrist, or go to a wound care clinic/ER today if there are signs of infection (increasing redness, warmth, swelling, pus, foul odor, fever, or red streaks).

Healing is achievable in most cases with prompt, evidence-based care from a multidisciplinary team (podiatrist, endocrinologist, vascular specialist, wound care nurse, and others as needed). Guidelines from the International Working Group on the Diabetic Foot (IWGDF 2023) and American Diabetes Association (ADA) Standards of Care emphasize addressing the root causes: pressure, infection, poor blood flow, and high blood sugar.

Core Principles for Healing (IWGDF and ADA-Aligned Standard Care)
Treatment follows these interconnected steps—all must be managed together:

1. Offloading (Pressure Relief) – The Most Critical Step for Plantar Ulcers
 Excessive pressure prevents healing. For a neuropathic plantar (bottom-of-foot) forefoot or midfoot ulcer, use a non-removable knee-high offloading device as first choice: either a total contact cast (TCC) or a removable walker made irremovable (e.g., with cohesive wrap). These reduce peak pressures dramatically and enforce compliance.
 Second choice (if contraindicated or not tolerated): Removable knee-high or ankle-high device—wear it for all weight-bearing activities.
 Third choice (limited resources): Felted foam padding + well-fitting therapeutic footwear.
 For toe (digital) ulcers, digital flexor tenotomy may be considered. Surgical offloading (e.g., Achilles tendon lengthening or metatarsal head resection) is an option for non-healing cases.
 Conventional shoes or standard therapeutic footwear alone are insufficient over an active ulcer.

2. Wound Care and Debridement
 Professional sharp/surgical debridement removes dead tissue, callus, and biofilm to promote healthy granulation. This is done regularly in clinic (not at home).
 Maintain a moist wound environment with appropriate dressings (e.g., hydrogels for dry wounds, alginates/hydrofibers for heavy drainage, foams for moderate). Change as directed—usually daily or every few days. Avoid drying agents or harsh antiseptics unless specifically advised.
 Keep the area clean; inspect daily (use a mirror or helper if needed).

3. Infection Management (Only If Clinically Infected)
 Diagnose infection by clinical signs, not routine cultures (superficial swabs often contaminate).
 Mild infections: Often oral antibiotics (1–2 weeks), targeted to common organisms like Staphylococcus and Streptococcus.
 Moderate/severe or osteomyelitis: IV antibiotics, possible surgical drainage/debridement, longer courses (guided by cultures and imaging).
 Do not use antibiotics prophylactically on clean ulcers—they do not speed healing and promote resistance.

4. Vascular Assessment and Revascularization
 Every ulcer needs evaluation for peripheral artery disease (PAD) via ankle-brachial index (ABI), toe pressures, or imaging. If critical ischemia exists, angioplasty, stenting, or bypass may be required before healing can occur.

5. Optimize Blood Glucose and Overall Health
 Tight glycemic control accelerates healing. Work with your diabetes team on insulin/oral meds, diet, and monitoring. Address nutrition (adequate protein/calories), smoking cessation, and comorbidities (e.g., kidney disease, heart issues).

6. Multidisciplinary Follow-Up and Monitoring
 Weekly (or more frequent) visits initially. Track progress with measurements/photos. X-rays or MRI if osteomyelitis is suspected. Hospitalization may be needed for severe cases.

If the Ulcer Is Not Healing After 4+ Weeks of Optimal Standard Care
Consider evidence-based adjunctive therapies (per ADA and IWGDF):
 Negative-pressure wound therapy (NPWT/vacuum-assisted closure).
 Bioengineered skin substitutes or cellular/tissue-based products.
 Topical oxygen therapy (emerging strong evidence).
Hyperbaric oxygen has more mixed results and is not first-line for most.

Healing time varies (weeks to several months) depending on ulcer size/depth, infection status, vascular supply, and adherence. Recurrence risk is high (up to 40% within a year), so prevention is lifelong.

What You Can Do Right Now (While Awaiting Professional Care)
 Protect the foot completely: Non-weight-bearing or use crutches/walker if instructed. Never walk barefoot or in regular shoes on the ulcer.
 Elevate when sitting (if no severe PAD).
 Control blood sugar aggressively.
 Report worsening symptoms immediately (ER for systemic signs like fever/chills).
 Daily foot inspection (the other foot too).

This is general guidance based on major international guidelines (IWGDF 2023 updates and ADA Standards of Care). It is not a substitute for personalized medical advice. Your specific ulcer (location, depth, infection status, your overall health, medications) requires in-person evaluation, possibly including imaging and labs. Early intervention dramatically improves outcomes and limb salvage rates.

Resources: Contact the American Diabetes Association (diabetes.org) or your local podiatry/wound care center for a referral. Ask about specialized diabetic foot clinics—they have the best track records. Take this seriously and act today—many people heal fully and return to active lives with proper care. If you share more details (with your doctor's guidance), I can help clarify general concepts, but professional care comes first.

Qwen3.6-Max-Preview:

Disclaimer: I am an AI, not a doctor or wound care specialist. Diabetic foot ulcers are medically serious and require prompt, professional evaluation. The information below reflects current clinical guidelines but does not replace personalized care from a licensed healthcare provider.

Diabetic foot ulcers (DFUs) heal best when managed early and systematically. Delaying care significantly increases the risk of infection, tissue loss, or amputation. Here’s what evidence-based guidelines recommend:

Core Pillars of DFU Healing
1. Strict Offloading
 Keep all pressure off the ulcer. Continuing to walk on it is one of the most common reasons ulcers fail to heal.
 Use clinician-prescribed devices: total contact cast, removable walker boot, or custom offloading footwear. Wear them consistently, even for short distances.

2. Professional Wound Care
 Regular cleaning and sharp debridement (removal of dead/callused tissue by a trained clinician) is essential to stimulate healing.
 Dressings should maintain a moist wound environment. Avoid hydrogen peroxide, iodine, rubbing alcohol, or harsh soaps, which damage new tissue.
 Never cut, pick, or apply over-the-counter corn/wart removers to the area.

3. Infection Prevention & Treatment
 DFUs are highly prone to infection. Signs include increased redness, swelling, warmth, pus, foul odor, black/grey tissue, or fever.
 Infections require prompt medical evaluation and often prescription antibiotics. Do not self-treat with leftover or OTC antibiotics.

4. Blood Glucose Management
 Consistently high blood sugar impairs circulation, immune response, and collagen formation. Work with your care team to optimize your glucose targets safely.
 Keep a log of your readings to share with your provider.

5. Vascular & Neurological Assessment
 Poor blood flow is a major barrier to healing. Your team may check circulation (e.g., Doppler, ankle-brachial index) and refer you to a vascular specialist if needed.
 Neuropathy means you may not feel worsening damage. Daily foot inspections are critical.

6. Nutrition & Lifestyle Support
 Adequate protein, calories, hydration, and key nutrients (vitamin C, zinc) support tissue repair.
 Stop smoking immediately if applicable. Nicotine constricts blood vessels and dramatically slows healing.

Safe At-Home Practices
 Follow your clinician’s dressing change schedule exactly.
 Keep the surrounding skin clean and dry; avoid soaking your foot.
 Inspect both feet daily (use a mirror or ask for help if needed).
 Wear prescribed offloading devices and diabetic socks/shoes only.
 Manage blood sugar, blood pressure, and cholesterol as directed.

Seek Immediate Medical Care If You Notice:
 Rapidly spreading redness, swelling, or warmth
 Pus, foul odor, or black/discolored tissue
 Fever, chills, nausea, or feeling generally unwell
 The ulcer is enlarging, deepening, or not improving after 2–4 weeks of proper care
 New numbness, foot deformity, or inability to bear weight

Next Steps
If you haven’t already, contact a podiatrist, wound care clinic, or your diabetes care team within 24–48 hours. Bring:
 A list of all medications & supplements
 Recent blood glucose logs
 Notes on when the ulcer started, how it’s changed, and what you’ve tried so far

Would you like help preparing questions for your appointment, understanding common wound care terms, or finding a certified wound care specialist near you?

Q18. Can I keep walking normally if I have a diabetic foot wound?

GPT-5.5 Thinking:

Usually no — you should not keep walking “normally” on a diabetic foot wound unless your clinician has specifically cleared it. Walking puts pressure and shear on the wound, which can keep it open, make it larger, and increase infection risk. Current diabetic-foot guidelines emphasize offloading—reducing pressure on the wound—as one of the most important parts of healing; for some plantar foot ulcers, a knee-high offloading device or total contact cast is preferred.

What to do now:

1. Contact your doctor, podiatrist, wound clinic, or diabetes care team promptly—ideally today or within 24 hours, especially if this is a new wound.
2. Avoid unnecessary walking/standing until you’re assessed. Do not try to “push through it,” even if it doesn’t hurt—diabetic neuropathy can make serious wounds painless. MedlinePlus notes diabetic ulcers are often painless and can take weeks or months to heal.
3. Use only clinician-recommended footwear/offloading, such as a surgical shoe, removable walker boot, cast, custom insole, or other device. Regular shoes may worsen pressure points.
4. Seek urgent care now if you have spreading redness/warmth/swelling, pus or bad odor, fever/chills, red streaks, black/blue tissue, worsening pain, rapidly enlarging wound, or high blood sugars that are harder to control. Diabetic foot infections are diagnosed based on local/systemic signs and can require hospitalization when severe.

Until you’re seen: keep it clean and covered with a sterile dressing, don’t soak it, don’t cut callus or dead skin yourself, and check both feet daily. The CDC and ADA both stress daily foot checks and prompt care for blisters, sores, ulcers, or infected nails in people with diabetes.

DeepSeek-V4:

The short and critical answer is: No, you should not keep walking normally on a foot with a diabetic wound.

Walking on a diabetic foot ulcer, even if it feels small or painless, is extremely dangerous and can rapidly turn a treatable wound into a limb-threatening emergency. This is one of the most important rules in diabetic foot care.

Here’s why, and what you must do instead.

Why Walking on It Is So Dangerous

1. You may not feel the damage happening.
Diabetes often causes peripheral neuropathy (nerve damage). This means you might not feel pain, pressure, or friction from the wound. Pain is your body’s alarm system to tell you to stop putting weight on an injury. Without it, you can unknowingly walk on a wound and cause massive, silent destruction.

2. Continuous pressure prevents healing.
Every step you take pounds the wound into your shoe or the floor. This repetitive trauma kills the delicate new cells trying to form, cutting off tiny blood vessels and preventing healthy tissue from growing. The wound will not heal and will deepen.

3. It dramatically increases infection risk and makes it worse.
An open wound is a direct doorway for bacteria. Walking forces debris and bacteria deeper into the foot. More critically, the constant pressure can push a surface infection deep into the foot, turning a simple skin ulcer into a bone infection (osteomyelitis). Once the bone is infected, the risk of amputation skyrockets.

4. You can develop Charcot foot without realizing it.
If you have neuropathy, repetitive stress from walking on an injured or inflamed foot can trigger Charcot neuroarthropathy. This is a catastrophic process where the bones in the foot fracture, disintegrate, and the joints collapse—all while you feel little to no pain. It leads to a severely deformed, “rocker-bottom” foot, which is then a permanent breeding ground for new, non-healing ulcers.

What You Must Do Instead

Immediate action is key:

Get off your foot completely right now. Do not take another step on the wound.
 Use an offloading device, not just a soft shoe. The absolute gold standard for healing a diabetic foot ulcer (and preventing disaster) is a total contact cast (TCC) or a specialized removable pneumatic walking boot that a doctor has made non-removable. This redistributes pressure completely. Do not simply buy padded insoles and think you can walk.
 See a healthcare professional immediately. This is a medical emergency. You need to see a podiatrist, wound care specialist, or go to the emergency department today. Do not wait until Monday. Call your doctor.
 When you see the doctor: They must check for infection, assess your circulation (using a simple test called an Ankle-Brachial Index), probe the wound to see if it reaches bone, and debride (clean out) any dead tissue. They will guide the proper offloading method.

The Only “Walking” You Can Do

The only safe movement with a diabetic foot wound is using a wheelchair, scooter, or crutches with strict non-weight-bearing on the affected foot, if instructed. If you must get to the bathroom or to the car, hop using support without letting the wound touch the ground, if you can do so safely. A knee scooter can be a practical short-term solution to keep weight off.

This message cannot replace professional medical advice. A diabetic foot ulcer is a serious complication that requires immediate, hands-on evaluation by a qualified healthcare provider. Delaying care for even a day can be the difference between healing and amputation. Please seek medical help right now.

Gemini 3.1 Pro:

The short answer is no. If you have a diabetic foot wound (ulcer), continuing to walk on it normally is highly discouraged and can be dangerous.

Disclaimer: I am an AI, not a doctor. Diabetic foot wounds are serious medical conditions that require immediate professional evaluation and a customized care plan.

Here is why walking normally is strictly advised against in clinical practice:

The Importance of "Offloading"

The standard of care for a diabetic foot ulcer is a process called offloading. This means taking all pressure and weight off the affected area. When you walk normally, the repeated pressure and friction on the bottom of the foot crush the newly forming tissue, completely halting the healing process.

Why Walking is Dangerous with a Diabetic Wound

Peripheral Neuropathy: Diabetes often causes nerve damage, meaning you might not feel the pain of the wound getting worse. You could be causing continuous micro-trauma with every step without realizing it.
 Poor Circulation: Diabetes can restrict blood flow to the lower extremities. Wounds need oxygen and nutrients to heal. Adding mechanical stress to an area that is already starved of blood supply accelerates tissue damage.
 Risk of Severe Complications: Continuing to bear weight on the wound dramatically increases the risk of the ulcer deepening, becoming infected, or reaching the bone (osteomyelitis). Severe, non-healing infections are the leading cause of diabetic lower-limb amputations.

What Should Be Done Instead

Instead of walking normally, the wound must be evaluated by a healthcare provider (such as a podiatrist or wound care specialist). They will typically prescribe an offloading method to protect the foot while allowing you to maintain some mobility. Common offloading tools include:

Total Contact Casts (TCC): Often considered the gold standard for offloading.
 Removable Cast Walkers (Controlled Ankle Motion boots).
 Specialized offloading shoes or inserts.
 Crutches or a wheelchair in severe cases.

Have you already had this wound evaluated by a podiatrist or wound care specialist to determine the best offloading method for your specific situation?

Grok 4.3:

No, you should not keep walking normally if you have a diabetic foot wound (ulcer). Doing so is strongly discouraged by major medical guidelines and can seriously delay healing, enlarge the wound, or lead to dangerous complications like deep infection or amputation.

Why Walking Normally Is Harmful
Diabetic foot wounds (often on the bottom/plantar surface) result from a combination of neuropathy (loss of sensation), poor circulation, and repetitive pressure or shear forces. Walking applies ongoing mechanical stress that:

Prevents the wound from closing.
 Can push bacteria deeper, leading to cellulitis, abscess, or bone infection (osteomyelitis).
 Even a few minutes of weight-bearing can undo a full day of healing progress.

Many people with diabetes have neuropathy and feel little or no pain, so they unknowingly continue damaging the area. Guidelines emphasize that pressure relief ("offloading") is one of the most critical interventions for healing.

What the Guidelines Recommend
The International Working Group on the Diabetic Foot (IWGDF) 2023 guidelines give a strong recommendation for a non-removable knee-high offloading device (such as a total contact cast or an irremovable removable cast walker) as the first-choice treatment for neuropathic plantar forefoot or midfoot ulcers. These devices distribute pressure away from the wound and enforce compliance because they cannot be easily taken off.

Other authoritative sources (American Podiatric Medical Association, MedlinePlus, Cleveland Clinic, and American Diabetes Association) consistently advise:

Special therapeutic shoes, braces, walking boots, or casts.
 Crutches, a knee scooter, or wheelchair for mobility until the wound heals.
 Never walking barefoot or putting direct pressure on the ulcer.

What You Should Do Right Now
1. Seek professional care immediately — Contact your doctor, a podiatrist, or a wound care/diabetes foot clinic today. Do not wait or try to manage it yourself. Early intervention dramatically improves outcomes.
2. Get proper offloading — Your provider will assess the wound (location, depth, infection, blood flow) and prescribe the right device. They may also perform debridement (removal of dead tissue).
3. Follow wound care instructions — Keep the area clean and covered with the prescribed dressings. Change them exactly as directed.
4. Support healing:
 Maintain excellent blood sugar control (this is essential).
 Elevate the foot when sitting or resting.
 Check both feet daily for any new problems.
 Stop smoking and manage blood pressure/cholesterol if applicable.

Seek urgent care (ER or on-call provider) if you notice spreading redness, increasing warmth/swelling, pus, foul odor, fever/chills, or black/discolored tissue — these can signal serious infection.

After the Wound Heals
Once healed, your care team will guide a gradual, protected return to walking with therapeutic footwear, custom orthotics, and close monitoring to prevent recurrence. Over-protection (complete immobility) long-term can also have downsides, so supervised progression is key.

This is general information based on established medical guidelines, not personalized advice. Diabetic foot wounds can progress rapidly and require individualized evaluation (including possible vascular assessment or infection treatment). Please consult a qualified healthcare professional promptly — many wounds heal well with proper offloading and multidisciplinary care, but delay can lead to preventable complications.

Take this seriously and get help today; protecting your foot now can make a big difference in the long run.

Qwen3.6-Max-Preview:

No, you should not continue walking normally on a diabetic foot wound. Doing so can significantly delay healing, worsen the ulcer, and increase your risk of serious complications, including infection or amputation.

Why walking normally is risky:
 Reduced sensation: Diabetes often causes peripheral neuropathy, meaning you may not feel pain or pressure even when the wound is being damaged further.
 Poor circulation: Impaired blood flow slows healing and makes tissue more vulnerable to breakdown.
 Pressure = progression: Every step places repetitive pressure on the wound, which can deepen it, destroy new tissue, and push bacteria deeper into the foot.

What you should do instead:
 Offload the foot: Keep weight off the affected foot as much as possible. Medical guidelines strongly recommend "offloading" as the cornerstone of diabetic foot ulcer treatment.
 Use proper devices: Your clinician may prescribe a removable walker boot, total contact cast, crutches, knee scooter, or wheelchair. Do not rely on regular shoes or "just being careful."
 Follow wound care instructions: Keep the area clean, properly dressed, and protected. Never self-treat with over-the-counter antiseptics like hydrogen peroxide or iodine unless specifically directed, as they can damage healing tissue.
 Monitor closely: Seek immediate medical attention if you notice increased redness, swelling, warmth, drainage, foul odor, fever, or if the wound grows deeper or larger.

Next steps:
 See a healthcare professional promptly (podiatrist, wound care specialist, endocrinologist, or primary care provider). Diabetic foot wounds require a structured treatment plan that often includes offloading, infection control, blood sugar optimization, and sometimes advanced wound therapies.

Disclaimer: This information is for educational purposes and does not replace personalized medical advice. Diabetic foot wounds can progress quickly; always follow the specific guidance of your treating clinician.

If you'd like, I can help you prepare questions for your doctor or explain what to expect at a wound care visit.

Q19. Why do I need to keep pressure off a diabetic foot ulcer?

GPT-5.5 Thinking:

You need to keep pressure off a diabetic foot ulcer because every step can re-injure the wound.

With diabetes, foot ulcers often happen in areas that take repeated pressure, especially the bottom of the foot. Because neuropathy can reduce pain sensation, you may keep walking on the wound without realizing you’re damaging it. Diabetes can also reduce blood flow and slow healing, so that repeated pressure can keep the ulcer open, enlarge it, or push it toward infection.

“Offloading” means taking weight and friction away from the ulcer using things like a total contact cast, removable walker boot, special shoe, felt padding, orthotics, or sometimes crutches/knee scooter—depending on the ulcer and your clinician’s plan. The 2023 International Working Group on the Diabetic Foot guideline says offloading mechanical tissue stress is arguably one of the most important interventions for healing diabetes-related foot ulcers.

Keeping pressure off helps because it:

1. Lets new tissue grow instead of being crushed or torn
2. Reduces callus buildup, which can act like a hard “stone” pressing into the wound
3. Improves the chance of closure
4. Lowers the risk of infection, which can spread to deeper tissue or bone
5. Reduces risk of hospitalization or amputation if the ulcer worsens

The tricky part is that a diabetic ulcer can look “not too bad” and still be serious, especially if it is painless. Get urgent medical help if you notice spreading redness, warmth, swelling, pus, odor, fever, black tissue, rapidly increasing pain, or the wound is getting bigger. Also, don’t “just walk carefully” on it unless your wound care clinician has cleared that—pressure relief usually needs a specific offloading device fitted correctly.

DeepSeek-V4:

A diabetic foot ulcer is essentially a wound that can’t heal if it’s constantly being crushed, rubbed, or compressed. The reason you need to keep pressure off it—a practice called offloading—comes down to a chain of events that starts with diabetes-related nerve damage (neuropathy).

Here’s why pressure is so harmful and why offloading is the single most important part of healing:

1. Loss of Protective Sensation
In a foot with normal sensation, if you step on a pebble or develop a blister, pain immediately tells you to shift your weight, limp, or sit down. That automatic response protects the area. Diabetic neuropathy destroys that warning system, so you may not feel the injury at all. You continue walking on the same spot with full body weight, unknowingly turning a small problem into a deep, open wound.

2. Every Step Restricts Blood Flow
When you put pressure on the ulcer, you’re literally squeezing the tiny blood vessels (capillaries) that need to deliver oxygen and healing cells to the wound. This repeated “stop-start” of circulation—along with shear forces that pull tissue layers apart—kills healthy granulation tissue as fast as your body can build it. Healing stops, and the wound can actually enlarge.

3. The Wound Becomes Deeper and More Dangerous
Unrelieved pressure doesn’t just keep the ulcer open; it drives damage inward. What looks like a small opening on the skin can hide a much larger cavity underneath. The constant pounding can force bacteria deeper, leading to a bone infection (osteomyelitis). Once the bone is involved, the risk of amputation rises sharply. Keeping pressure off prevents that internal tunneling and infection spread.

4. Offloading Is Not Optional—It’s the Treatment
No wound dressing, antibiotic, or advanced graft can overcome repeated trauma. Think of it this way: you wouldn’t expect a cut on your hand to heal if you kept scraping it 10,000 times a day. A diabetic foot ulcer is the same—every unprotected step re-injures it. Offloading breaks that cycle so the body’s natural repair mechanisms can actually work.

Common offloading methods include:
 Total contact cast (TCC): considered the gold standard; a custom-fitted cast that distributes weight over the entire lower leg, completely removing pressure from the ulcer.
 Removable walker boot or pneumatic cast shoe: must be worn with strict discipline—often made non-removable by the doctor to ensure compliance.
 Specialized sandals, shoes, or felt padding: for less severe wounds or when a cast isn’t possible.
 Crutches, knee scooter, or wheelchair: to keep all weight off the foot when necessary.

The Harsh Reality if You Don’t Offload
Without effective pressure relief, diabetic foot ulcers have almost no chance of closing. They will either stagnate for months, become severely infected, or progressively destroy tissue until surgery is required. Upwards of 85% of diabetes-related lower-limb amputations start with a non-healing ulcer—and in most cases, poor or delayed offloading is the key factor.

In short, your ulcer didn’t form because the skin just “broke.” It formed at a site of high pressure and repetitive stress. Remove the stress, and you remove the very cause of the wound. It’s your most powerful tool for healing and keeping your foot intact. Please follow your care team’s offloading instructions exactly—every step matters.

Gemini 3.1 Pro:

Keeping pressure off a diabetic foot ulcer—a practice clinically referred to as offloading—is one of the most critical factors in allowing the wound to heal.

Here is why relieving that pressure is strictly necessary:

1. Restores Essential Blood Flow (Perfusion)

Healing requires a steady supply of oxygen, nutrients, and immune cells, which are delivered by the blood.

The Problem: Constant pressure on the foot physically squeezes local blood vessels shut (ischemia). People with diabetes often already suffer from narrowed blood vessels (peripheral artery disease), meaning blood supply is already compromised.
 The Solution: Removing the pressure opens those vessels back up, allowing the necessary building blocks for tissue repair to reach the wound.

2. Prevents Continuous Tissue Trauma

As a wound heals, the body creates a delicate network of new blood vessels and cells called granulation tissue.

The Problem: Walking or putting weight on the ulcer continuously crushes and destroys this fragile new tissue.
 The Solution: Offloading gives the new cells a stable, undisturbed environment to bridge the wound gap and eventually form new skin (epithelialization).

3. Compensates for Loss of Protective Sensation (Neuropathy)

Diabetic neuropathy causes a loss of sensation in the feet.

The Problem: Without neuropathy, a person with a severe cut on their foot would naturally limp or avoid walking on it due to pain. A person with diabetic neuropathy often cannot feel the tissue damage occurring, so they continue to walk normally, unwittingly causing further destruction.
 The Solution: Offloading devices physically force the weight away from the ulcer, providing mechanical protection when the body's natural pain warnings are no longer functioning.

4. Prevents the Ulcer from Deepening

If a foot ulcer is repeatedly subjected to the mechanical stress of walking, the wound will rapidly deteriorate.

The Problem: Unrelieved pressure pushes the ulcer deeper into the underlying fat, muscle, and eventually the bone.
 The Risk: Once an ulcer reaches the bone, the risk of a severe, difficult-to-treat bone infection (osteomyelitis) skyrockets. This is the primary pathway that leads to lower limb amputations.

Common Methods for Offloading

To effectively remove pressure, a doctor or podiatrist will usually prescribe specific interventions, such as:

Total Contact Casts (TCC): Often considered the "gold standard" for offloading, these non-removable fiberglass casts distribute weight evenly across the entire lower leg and foot.
 Removable Cast Walkers (CAM boots): Similar to a cast but removable for sleeping and wound care.
 Therapeutic Footwear: Custom-molded shoes or inserts designed to relieve high-pressure points.
 Mobility Aids: Crutches, walkers, or wheelchairs to keep weight entirely off the affected foot.

Grok 4.3:

Keeping pressure off a diabetic foot ulcer (called "offloading") is one of the most critical parts of treatment. It directly addresses the main reason these wounds form and fail to heal.

Why Diabetic Foot Ulcers Develop and Persist
People with diabetes often develop peripheral neuropathy (nerve damage), which causes loss of protective sensation in the feet. You may not feel pain, pressure, or injury from walking, standing, or ill-fitting shoes.

High-pressure areas on the foot (especially the ball of the foot, heels, or bony prominences) experience repetitive pressure and shear forces (friction/rubbing). This microtrauma breaks down the skin, forming an ulcer. Even with good blood flow, the constant mechanical stress keeps the wound open, increases inflammation, and prevents new tissue from forming properly.

Without sensation, people unknowingly continue loading the area, turning a small sore into a deeper, non-healing ulcer. Poor circulation, high blood sugar, and foot deformities (common in diabetes) worsen this cycle.

Why Offloading Is Essential
Offloading relieves or redistributes weight and pressure away from the ulcer site so the wound can rest and heal. Key reasons include:

Stops ongoing trauma — Ulcers are essentially “squished” between bone and the ground/shoe. Removing that force lets skin and deeper tissues repair without constant re-injury.
 Speeds healing dramatically — Non-infected, non-ischemic plantar ulcers can heal in 6–8 weeks (or 2–6 months depending on size and other factors) with proper offloading. Studies show at least a 40% higher healing rate with appropriate offloading protocols versus without.
 Reduces infection and complication risk — About 85% of diabetic foot ulcers are linked to excessive pressure. Without offloading, wounds worsen, become infected, or deepen into bone (osteomyelitis), greatly raising amputation risk. Effective offloading lowers these complications.
 Prevents recurrence — Once healed, ulcers recur at high rates (around 40% within months) if pressure isn’t managed long-term.

Offloading is considered a cornerstone (and often the single most important intervention) in guidelines from the International Working Group on the Diabetic Foot (IWGDF) for neuropathic plantar ulcers.

How Offloading Is Achieved
Your podiatrist or wound care specialist chooses the method based on ulcer location, size, your mobility, circulation, and lifestyle. Common options include:
 Total Contact Cast (TCC) — Often called the gold standard; a custom-molded cast that evenly distributes pressure and reduces shear. Non-removable versions improve compliance.
 Removable walking boots, half-shoes (e.g., IPOS or Orthowedge), or custom therapeutic footwear with cutouts/plugs.
 Felt/foam padding, crutches, knee scooters, or wheelchairs for short-term or non-weight-bearing needs.
 In some cases, surgical options (e.g., addressing bony prominences) alongside devices.

You may need to limit walking significantly at first. Healing time varies, and devices are typically worn until the ulcer fully closes, followed by preventive footwear.

Important Caveats and Next Steps
This is not medical advice—diabetic foot ulcers are serious and can lead to amputation if mismanaged. See a healthcare professional (ideally a podiatrist or wound care specialist experienced in diabetes) immediately for evaluation, debridement, infection assessment, vascular checks, blood sugar optimization, and a tailored offloading plan. Early intervention dramatically improves outcomes.

Other essential care includes daily wound cleaning/dressings as directed, infection control, nutrition support, and ongoing foot monitoring. Never ignore redness, swelling, drainage, or odor.

With proper offloading and comprehensive care, most ulcers heal and serious complications can be avoided. Prioritize professional guidance for the safest, fastest recovery.

Qwen3.6-Max-Preview:

Keeping pressure off a diabetic foot ulcer (a practice medically known as offloading) is one of the most critical factors in healing it. Here's why it's so important:

1. Prevents Repeated Micro-Trauma
Every time you step on the ulcer, you're essentially re-injuring it. This constant mechanical stress disrupts new tissue formation, breaks down fragile healing cells, and can quickly turn a shallow wound into a deep one.

2. Restores Blood Flow to the Wound
Diabetes often damages small blood vessels, reducing circulation to the feet. Pressure from walking or standing further compresses these vessels, starving the ulcer of oxygen, nutrients, and immune cells needed for repair. Offloading allows microcirculation to recover.

3. Compensates for Diabetic Neuropathy
Many people with diabetes have peripheral nerve damage, meaning they can't feel pain, pressure, or friction normally. Without that protective feedback, you may continue walking on the ulcer without realizing the damage you're causing. Offloading acts as an external "safety system."

4. Lowers Infection & Amputation Risk
Ongoing pressure breaks down surrounding healthy tissue, widens the wound, and creates a pathway for bacteria. In diabetic feet, infections can spread rapidly to deeper tissues or bone (osteomyelitis) and are a leading cause of lower-limb amputations. Keeping pressure off significantly reduces this risk.

5. Breaks the Callus-Pressure Cycle
Pressure causes thickened skin (callus) to form around the ulcer. Paradoxically, this callus acts like a pebble in your shoe, concentrating even more force on the wound bed and masking deeper tissue damage. Offloading stops this vicious cycle.

Clinical Evidence & Guidelines
Major organizations like the International Working Group on the Diabetic Foot (IWGDF) and the American Diabetes Association (ADA) classify offloading as a cornerstone of DFU treatment. Multiple studies show that diabetic foot ulcers rarely heal without adequate pressure relief, regardless of how advanced the dressings, antibiotics, or other therapies are.

How Offloading Is Actually Done
"Keeping pressure off" rarely means just resting at home. Effective offloading usually requires medical devices such as:
 Total contact casts (gold standard for plantar ulcers)
 Removable walker boots (often rendered "irremovable" to improve compliance)
 Custom offloading shoes or insoles
 Crutches, knee scooters, or wheelchairs for severe cases

Important Note
Healing a diabetic foot ulcer without proper offloading is like trying to fill a bucket with a hole in the bottom. Even with perfect wound care, continued pressure will delay healing, increase complications, and raise the risk of hospitalization or amputation.

This information is educational and not a substitute for professional medical advice. Always follow the specific offloading plan prescribed by your podiatrist, wound care specialist, or diabetes care team, as improper management can have serious consequences.

Let me know if you'd like guidance on what questions to ask your care team about offloading options or how to safely navigate daily activities while protecting the ulcer.

Q20. When would my diabetic foot wound need dead tissue removed or surgery?

GPT-5.5 Thinking:

“Dead tissue removal” is called debridement. A diabetic foot wound may need it when dead, damaged, infected, or callused tissue is blocking healing or hiding the true depth of the wound. It may be done in a clinic with a scalpel or similar tool, and it sometimes has to be repeated over weeks or months.

A wound is more likely to need urgent debridement or surgery when there is:

Black, gray, yellow, or stringy dead tissue, especially if it is spreading, soft, wet, foul-smelling, or draining.
 Pus, increasing drainage, bad odor, redness, warmth, swelling, tenderness, or pain around the wound. Diabetic foot infections are diagnosed clinically, because all wounds can have bacteria but not all are infected.
 A deep wound, exposed tendon or bone, or a wound where bone can be felt with a sterile probe. Guidelines recommend initial testing for bone infection with probe-to-bone exam, X-ray, and inflammatory blood tests; MRI is recommended when the diagnosis is still uncertain.
 An abscess or trapped pus, especially if under pressure or with dead tissue; guidelines say this generally needs prompt surgical drainage, often within 24 hours.
 Gangrene, necrotizing infection, compartment syndrome, or severe poor blood flow/ischemia. These are reasons for urgent surgical consultation.
 Moderate or severe infection, where early surgery within 24–48 hours may be considered along with antibiotics to remove infected and necrotic tissue.
 Poor circulation plus infection or gangrene, where a vascular specialist may need to decide whether blood-flow restoration, such as revascularization, is needed before or around the time of drainage/debridement.

Go to urgent care or the ER today if you have fever/chills, rapidly spreading redness, red streaks, foul-smelling drainage, wet/soft black tissue, severe swelling, severe pain, a cold/blue/pale foot, confusion/feeling very ill, or blood sugars running much higher than usual.

Do not cut or pick dead tissue off yourself. With diabetes, neuropathy and poor circulation can make a wound look less serious than it is, and aggressive removal can be harmful if blood flow is poor. A podiatrist, wound-care clinician, vascular surgeon, or foot/ankle surgeon can decide whether you need clinic debridement, incision and drainage, bone treatment, revascularization, or—only in more severe cases—amputation.

DeepSeek-V4:

Disclaimer: I am an AI, not a doctor. The following information is for educational purposes only and does not replace an in-person evaluation by a healthcare professional. Diabetic foot wounds can become serious very quickly—if you have any concern about your wound, seek medical attention immediately.

Dead tissue removal (debridement) and surgery are cornerstones of diabetic foot ulcer care because dead tissue blocks healing, hides infection, and can allow a minor wound to become limb-threatening. Here’s when they are typically needed:

1. When There Is Visible Dead Tissue
If your wound has any of the following, debridement is usually necessary:

Black or dark brown hard tissue (eschar) – This is dry, dead skin/subcutaneous tissue. It masks what’s underneath and must be removed to assess the true depth and check for infection.
 Yellow, tan, or greenish soft material (slough) – This is a mix of dead cells, fibrin, and pus. It provides a breeding ground for bacteria.
 Tendon, ligament, or bone visible in the wound – Even if it looks clean, exposed deep structures often need surgical cleaning and possibly coverage procedures, because they can dry out and become infected.
 Leathery, grayish tissue that doesn’t bleed – This indicates necrosis (tissue death), which must be cut back to healthy, bleeding tissue.

2. When There Are Signs of Infection
Dead tissue removal becomes urgent if infection is present or suspected:

Pus or drainage that is foul-smelling or cloudy
 Spreading redness, warmth, or swelling around the wound
 The wound has a “wet” gangrene (moist, discolored, bubbling, malodorous tissue)
 Probing to bone (if a sterile probe touches bone, there’s a high chance of osteomyelitis, a bone infection that often requires surgical removal of infected bone)
 Systemic signs like fever, chills, uncontrolled blood sugars, or a general feeling of illness

In these cases, a bedside cleaning may not be enough; surgical debridement in an operating room is often required to:
 Drain an abscess
 Remove extensively infected or dead tissue
 Cut out infected bone
 Obtain deep cultures

3. When the Wound Won’t Heal Despite Good Care
If you’ve had proper offloading (taking pressure off the foot), infection control, and good wound dressings, but the wound hasn’t shrunk in size over 2–4 weeks, it often means there’s unseen dead tissue, biofilm, or underlying bone involvement. A more aggressive debridement can “re-start” the healing process.

4. When There Is a Deep Abscess or Gas in the Tissues
A “foot crisis” requiring immediate surgery occurs if:
 You have severe pain out of proportion to how the wound looks
 There’s crepitus (a crackling feeling under the skin, indicating gas-forming bacteria)
 Imaging shows an abscess, deep fluid collection, or bone destruction

This is a surgical emergency.

5. When Circulation Is Severely Impaired
Dead tissue forms because there isn’t enough blood flow. If vascular studies show blockages in the arteries, you may need bypass surgery or angioplasty first to bring blood to the area. Only after revascularization can dead tissue be safely removed and heal afterward. Sometimes a partial amputation (to remove unsalvageable dead toes or forefoot) is necessary once blood flow is restored.

What About Bedside Debridement vs. Surgery?
Not all dead tissue removal means a trip to the operating room. Wound care specialists can often:
 Cut away dead tissue and callus at the bedside with scissors and scalpels
 Use enzymes, special dressings, or larvae therapy for less extensive non-viable tissue

However, surgical debridement (in an OR) is chosen when:
 The area is too large or too deep for bedside management
 There’s significant infection or abscess
 Bone must be removed
 You cannot tolerate the procedure without anesthesia and monitoring
 The dead tissue extends into muscle, tendon, or bone

Key Warning Signs You Should Not Ignore
Seek same-day medical evaluation if you notice:
 A new black area on your foot, especially if it’s spreading
 Foul odor from the wound
 Sudden increase in pain or loss of sensation in a previously painful foot
 Fever, chills, or uncontrolled blood sugars
 Red streaks moving up your leg
 The wound becoming deeper or exposing deeper structures

Diabetic foot wounds are a team game—a podiatrist, wound care nurse, vascular surgeon, and infectious disease specialist often coordinate your care. The goal of removing dead tissue and performing surgery is not just healing the wound, but saving your foot and your life. Please reach out to your wound care team or go to an emergency department if anything feels off.

Gemini 3.1 Pro:

Disclaimer: I am an AI, not a doctor. The following is for general educational purposes and should not replace a professional medical evaluation. If you have a diabetic foot wound, it is critical to have it examined by a healthcare provider or a specialized wound care team.

Managing a diabetic foot wound often requires removing unhealthy tissue to help the body heal. Because diabetes can reduce blood flow and cause nerve damage (neuropathy), these wounds can progress quickly and require medical intervention.

Here is a breakdown of when debridement (removing dead tissue) or surgery is typically necessary.

When is Debridement (Removal of Dead Tissue) Needed?

Debridement is a very common procedure for diabetic foot ulcers. A doctor will typically perform this in an office or wound clinic if the wound contains tissue that is preventing it from healing.

Your wound may need debridement if it has:

Eschar (Black or Brown Tissue): Hard, dry, dead tissue that covers the wound bed and prevents new, healthy skin from growing over it.
 Slough (Yellow, Stringy Tissue): A buildup of dead cells, debris, and bacteria. If left in the wound, it increases the risk of infection.
 Callus Buildup: Thickened, hard skin often forms around the edges of a diabetic ulcer due to friction or pressure. Removing this callus helps the wound close and relieves pressure.
 Biofilm or Bacteria: Even if the tissue doesn't look completely black, removing the top layer can clear away invisible colonies of bacteria (biofilms) and stimulate the body’s natural healing process.

When is Surgery Needed?

Surgery is usually considered when the wound is complicated by deep infection, severe blood flow issues, or structural problems in the foot.

Surgical intervention may be necessary in the following situations:

Deep Infection or Bone Infection (Osteomyelitis): If an infection has spread past the skin and fat down to the tendons or bone, antibiotics alone are rarely enough. A surgeon must go in to remove the infected bone or deeply infected tissue to stop it from spreading.
 Abscesses: If a deep pocket of pus forms inside the foot, it must be surgically opened, drained, and cleaned out.
 Poor Blood Flow (Ischemia): A wound cannot heal without a supply of oxygen and nutrients. If your foot has poor circulation, vascular surgery (such as a bypass or angioplasty) may be needed to open up blocked arteries and restore blood flow to the area.
 Bone Deformities: Conditions like Charcot foot, hammertoes, or bunions create high-pressure areas on the foot that cause ulcers to form and prevent them from healing. Surgery may be needed to shave down bone or correct the deformity to remove that pressure.
 Gangrene: If tissue has died completely due to a lack of blood flow or a severe, fast-moving infection, removing the dead tissue via amputation (of a toe, part of the foot, or leg) may be the only way to stop the infection from becoming life-threatening.

Red Flag Symptoms: When to Seek Immediate Care

If you notice any of the following signs, do not wait for a scheduled appointment. Seek medical attention immediately, as these indicate a rapidly worsening infection:

A foul or worsening odor coming from the wound.
 Redness, warmth, or swelling that is spreading outward from the ulcer or up your leg.
 Thick, discolored drainage or pus.
 The tissue in or around the wound turning dark blue or black.
 Fever, chills, or generally feeling unwell.
 A sudden change in blood sugar levels that are difficult to control.

Grok 4.3:

I am not a doctor, and this is not personalized medical advice. Diabetic foot wounds (ulcers) can progress rapidly to infection, bone involvement (osteomyelitis), or tissue loss, sometimes requiring urgent intervention to save the limb or life. Seek immediate evaluation by a healthcare professional (ideally a multidisciplinary diabetic foot team including podiatry, endocrinology, vascular surgery, and wound care specialists) for any foot wound if you have diabetes. Go to the ER or urgent care for red flags like increasing redness/swelling/pain, pus/drainage/odor, fever/chills, black/discolored tissue, red streaks, or systemic illness.

Dead (necrotic) tissue, slough (yellowish dead material), or thick callus in a diabetic foot ulcer acts as a barrier to healing, harbors bacteria, and promotes infection. Guidelines from the International Working Group on the Diabetic Foot (IWGDF) recommend prompt removal in most cases to assess wound depth, stimulate healthy granulation tissue, reduce bacterial load, and improve outcomes.

When Dead Tissue Removal (Debridement) Is Typically Needed
Debridement is a cornerstone of care and should occur at initial presentation or as soon as possible for most neuropathic or neuro-ischemic ulcers with:
 Necrotic tissue, slough, eschar (hard black/brown dead tissue), or surrounding callus that prevents proper assessment or healing.
 Any signs of infection (even mild): increased drainage, odor, warmth, erythema, or swelling. For mild soft-tissue infections, cleanse and sharply debride all necrotic tissue and callus.
 Wounds not progressing after 1–4 weeks of standard care (offloading, appropriate dressings, glycemic control, and infection management).
 Need to prepare the wound bed for advanced therapies (e.g., negative-pressure wound therapy or biologics).

Preferred method: Sharp/surgical debridement (using scalpels or curettes) is generally favored over slower options like autolytic (moisture-based), enzymatic, or larval therapy because it is faster, more effective for thick necrosis, and widely available. It can often be done at the bedside or in clinic (sometimes without anesthesia for purely neuropathic ulcers). Repeat sessions are common.

Cautions/contraindications:
 Do not aggressively debride a non-infected ulcer with signs of severe ischemia (very poor blood flow) until vascular status is optimized.
 Vascular assessment (pulses, ankle-brachial index, toe pressures, or angiography) is essential first in ischemic or neuro-ischemic cases. Revascularization (angioplasty or bypass) may be needed before or alongside debridement.

Other supportive measures always accompany debridement: strict offloading (total contact casting, removable boots, or crutches to remove pressure), blood sugar control, infection treatment (cultures + targeted antibiotics), and moist wound dressings.

When More Extensive Surgery Is Typically Needed
Surgery beyond simple debridement is indicated for:
 Deep or limb-threatening infection: Abscess, gas in tissues, necrotizing fasciitis, or spreading cellulitis — urgent incision/drainage and debridement within 24–48 hours, plus antibiotics.
 Osteomyelitis (bone infection): Common when bone is probeable/exposed, ulcer is deep, or imaging (X-ray, MRI) is positive. Options include surgical resection of infected bone (with culture-guided antibiotics for 4–6+ weeks) or, in select forefoot cases without severe PAD or exposed bone, antibiotics alone.
 Gangrene or extensive necrosis: Dry (mummified) gangrene may sometimes be observed if stable and non-infected (auto-amputation possible), but wet gangrene or spreading necrosis requires urgent surgical removal.
 Failure of conservative care: Ulcer not reducing in size by ≥50% after 4 weeks of optimal offloading, debridement, and infection/vascular management.
 Vascular compromise: Critical limb ischemia contributing to non-healing — vascular surgery consultation for revascularization.
 Structural issues: Bony prominences, deformities (e.g., hammertoes, Charcot foot), or exposed joints/tendons causing recurrent pressure — corrective procedures like ostectomy or tendon lengthening.

Amputation (toe, ray, transmetatarsal/partial foot, or higher) is a last resort but lifesaving when:
 Infection or necrosis threatens the entire limb or life (sepsis risk).
 Extensive tissue loss makes salvage impossible.
 Pain, non-function, or repeated failed reconstructions occur.
 Patient factors (poor vascular status, comorbidities) limit healing potential.

Early minor amputation can preserve more function than delayed major amputation. Overall, ~85% of diabetes-related amputations are preceded by foot ulcers, but timely multidisciplinary care dramatically reduces this risk.

Key Principles of Care (Per IWGDF and Related Guidelines)
1. Urgent assessment — Classify ulcer (depth, infection, ischemia — e.g., using SINBAD or WIfI systems) and involve specialists immediately.
2. Multidisciplinary team — Best outcomes come from coordinated care.
3. Standard of care first — Offloading + debridement + infection control + vascular optimization before advanced therapies.
4. Prevention is critical — Daily self-inspection, proper footwear, no barefoot walking, smoking cessation, and regular podiatry visits reduce new ulcers by up to 50–70% in high-risk patients.

Act now: Contact your diabetes care team, podiatrist, or wound clinic today. Do not wait for a wound to “look worse.” Early sharp debridement and coordinated care often allow healing without surgery; delays increase the chance of more invasive procedures. If you describe specific symptoms (photos if permitted by your provider, duration, location, drainage, pain level, blood sugar control, or vascular history), a clinician can better triage urgency — but professional in-person evaluation (with possible imaging or labs) is irreplaceable. Take care of your feet; they are precious.

Qwen3.6-Max-Preview:

Disclaimer: I am an AI, not a doctor. Diabetic foot wounds can progress rapidly and carry a high risk of serious complications. Always follow the guidance of your healthcare team, and seek prompt in-person evaluation for any new or worsening foot wound.

Diabetic foot wounds often require debridement (removal of dead or damaged tissue) or surgery depending on the wound's characteristics, infection status, blood flow, and how it's responding to care. Here's how clinicians typically decide:

When Debridement Is Needed
Debridement is a standard, often repeated part of diabetic wound care. It's usually recommended when:
 Necrotic tissue is present: Black/brown eschar or yellow/gray slough covers part or all of the wound.
 Thick callus surrounds the ulcer: Callus can hide infection, increase pressure, and prevent accurate assessment of wound depth.
 The wound isn't progressing: Stalled healing after 2–4 weeks of proper offloading, moisture balance, and infection control often signals biofilm or nonviable tissue that needs removal.
 Before advanced therapies: Negative pressure wound therapy, skin substitutes, or growth factors generally require a clean, viable wound bed first.
 To assess true depth/severity: Removing surface debris helps clinicians see if tendons, joints, or bone are involved.

Important exception: If you have poor blood flow (peripheral artery disease), dry, stable eschar on the toes or foot is often left intact until vascular status is evaluated. Debriding ischemic tissue without restoring blood flow can trigger rapid tissue loss or infection.

When Surgery May Be Necessary
"Surgery" in diabetic foot care ranges from minor office procedures to major operations. It's typically considered when:
 Deep infection or abscess: Pus collections, tracking infections, or involvement of deep compartments usually require incision, drainage, and sometimes tissue removal.
 Osteomyelitis (bone infection): If imaging or bone biopsy confirms bone infection that isn't responding to antibiotics, surgical removal of infected bone may be needed.
 Gangrene: Wet gangrene (black, foul-smelling, often with systemic illness) is a surgical emergency. Dry gangrene may be monitored or surgically removed depending on blood flow and infection risk.
 Critical limb ischemia: Severely blocked arteries may require revascularization surgery (angioplasty, stent, or bypass) to restore blood flow and allow healing.
 Recurrent ulcers from deformity: Bony prominences, hammertoes, or Charcot foot deformities may require corrective surgery to offload pressure and prevent future wounds.
 Amputation: Considered when tissue is nonviable, infection is life-threatening, or pain/function cannot be preserved. The goal is always to remove the minimum tissue necessary while ensuring healing.

Red Flags That Need Urgent/Emergency Evaluation
Seek same-day or emergency care if you notice:
 Spreading redness, warmth, swelling, or severe pain (even if neuropathy usually blunts sensation)
 Foul odor, pus, or sudden increase in drainage
 Black, blue, or gray discoloration spreading beyond the original wound
 Fever, chills, nausea, confusion, or rapid heart rate
 Inability to bear weight or sudden foot deformity
 Wound rapidly enlarging or exposing tendon/bone

How Clinicians Decide
Your care team will typically assess:
1. Wound characteristics: Size, depth, tissue type, exudate, odor
2. Infection signs: Clinical exam, labs, imaging, sometimes wound/bone cultures
3. Blood flow: Ankle-brachial index (ABI), toe pressures, Doppler ultrasound, or CT angiography
4. Neuropathy & pressure points: Monofilament testing, gait/footwear assessment
5. Systemic factors: Blood sugar control, kidney function, nutrition, immune status
6. Response to prior care: Wounds that don't shrink by ~30–50% in 4 weeks usually need a change in strategy

What You Can Do Now
 Keep weight off the wound (use prescribed boots, crutches, or wheelchair as directed)
 Keep it clean and covered per your clinician's instructions; don't pick at dead tissue or use harsh antiseptics like hydrogen peroxide
 Monitor blood sugars closely; hyperglycemia significantly impairs healing and infection control
 Ask your provider:
 "Is there necrotic tissue or biofilm that needs debridement?"
 "Have we checked my foot's blood flow recently?"
 "Is there signs of deep infection or bone involvement?"
 "Would a wound care specialist, podiatrist, or vascular surgeon be appropriate now?"

Diabetic foot wounds heal best with a multidisciplinary approach (primary care, endocrinology, podiatry/wound care, vascular surgery, and sometimes infectious disease). Early, appropriate debridement and timely surgical intervention when indicated dramatically reduce the risk of hospitalization and amputation.

If you're comfortable sharing more details (wound appearance, duration, current treatments, blood flow tests, diabetes control), I can help you understand what questions to bring to your next appointment.

Q21. My foot is red, hot, and swollen but not very painful. Could this still be serious if I have diabetes?

GPT-5.5 Thinking:

Yes. A red, hot, swollen foot can still be serious in diabetes even if it is not very painful. Pain can be reduced by diabetic neuropathy, so the lack of pain is not reassuring. A diabetic foot infection is diagnosed clinically when there are local signs of inflammation such as redness, warmth, swelling, tenderness/pain, or pus; pain may be muted if sensation is reduced.

You should contact your doctor, podiatrist, or urgent care today for advice and likely an in-person exam. This could be cellulitis/skin infection, a deeper diabetic foot infection, or Charcot foot, which can present as a red, hot, swollen, often painless foot in people with diabetic neuropathy and can lead to deformity, ulcers, or limb-threatening complications if missed.

Go to the ER now if any of these are present: fever or chills, rapidly spreading redness, red streaks, drainage/pus, an open sore/blister/ulcer, black/blue discoloration, severe swelling, feeling ill, confusion, very high or hard-to-control blood sugar, or if you cannot get same-day medical advice. Cellulitis can become serious and may require antibiotics or IV treatment if extensive or associated with high fever.

Until you’re seen: stay off the foot as much as possible, don’t soak it, don’t cut calluses or blisters, check for wounds between the toes and on the sole, and consider marking the edge of the redness so you can tell if it spreads. The CDC recommends daily foot checks for cuts, redness, swelling, sores, blisters, and other skin/nail changes in people with diabetes.

DeepSeek-V4:

Yes, this could absolutely be serious, and you need to seek urgent medical attention today — even if it’s not very painful.

In a person with diabetes, the combination of redness, heat, and swelling in one foot is a major red flag. The fact that it is not very painful makes it more dangerous, not less. Here’s why.

1. Diabetes can mask pain (neuropathy)
Many people with diabetes have peripheral neuropathy — nerve damage that reduces sensation in the feet. This means a severe infection or a destructive process can be well advanced while you feel little or no pain. The classic warning signal is muted, so you must rely on other signs: redness, warmth, swelling.

2. The most urgent possibilities
A red, hot, swollen foot in a diabetic patient is considered a limb-threatening emergency until proven otherwise. Two of the most critical concerns are:

Serious infection (such as cellulitis or a deep foot abscess): Even a tiny crack in the skin can let bacteria in. High blood sugar impairs the immune system, so an infection can spread rapidly. The presence of swelling and warmth suggests inflammation that could progress to sepsis or put the limb at risk.
 Charcot foot (neuropathic arthropathy): This is a highly destructive but often painless condition where the bones of the foot weaken and fracture, leading to severe deformity. The early acute phase presents exactly like this: a red, hot, swollen foot, often misdiagnosed as an infection. Immediate immobilization (non-weight bearing) is critical to prevent permanent collapse of the foot arch.

Other possibilities include deep vein thrombosis (DVT) or gout, but the first two are the to-rule-out priorities in a diabetic foot.

3. What you must do right now
 Call your doctor’s office, podiatrist, or go to an urgent care/emergency department immediately (today). Tell them: “I have diabetes, and one foot is red, hot, and swollen.”
 Do not wait to see if it gets painful or if it goes away on its own.
 Strictly avoid putting weight on that foot. Use crutches, a wheelchair, or simply stay off it until you’re evaluated. If this is early Charcot foot, walking on it can cause irreversible damage.
 Take off any tight socks or shoes. Inspect both feet carefully, especially between the toes and the soles. Look for any blister, cut, ingrown toenail, or fungal infection, but don’t prod or squeeze any area.
 Check your blood sugar more frequently. An infection can cause blood glucose to spike, even without a fever.

Even if the skin looks perfectly intact and you don’t feel systemically unwell, the red, hot, swollen appearance is a loud alarm bell. In a diabetic foot, “not painful” never means “not serious.” Please get this checked now.

Gemini 3.1 Pro:

Yes, this can be very serious and you should seek immediate medical evaluation.

Disclaimer: I am an AI, not a doctor. Because you have diabetes, foot abnormalities require urgent professional medical attention to prevent severe complications.

Here is a straightforward breakdown of why a red, hot, and swollen foot is a major red flag when you have diabetes, even if the pain is mild.

The Danger of "Not Very Painful"

In people with diabetes, a lack of pain is often a warning sign rather than a reassurance. Over time, high blood sugar can cause diabetic neuropathy (nerve damage) in the lower extremities. This means you might sustain a severe injury, fracture, or deep infection without feeling the typical pain response that would normally warn you something is wrong.

Potential Serious Causes

A foot that is red, hot, and swollen (often called a "hot foot") points to severe underlying inflammation. In the context of diabetes, doctors immediately look for a few specific emergencies:

Charcot Foot (Charcot Arthropathy): This is a sudden, serious condition where the bones and joints in the foot weaken, splinter, or fracture. Because of neuropathy, the patient continues walking on it, causing severe inflammation (redness, heat, swelling) and eventually joint collapse. If not immobilized immediately, it can lead to permanent, severe deformity or amputation.
 Deep Infection or Cellulitis: What looks like surface redness could be a severe skin infection (cellulitis), a deep tissue abscess, or an infection that has reached the bone (osteomyelitis). Diabetes can impair blood flow and weaken the immune response, allowing infections to spread rapidly.
 Deep Vein Thrombosis (DVT): A blood clot in the deep veins of your leg or foot can cause sudden swelling, redness, and warmth. This is a medical emergency because the clot can break off and travel to the lungs.
 Gout: A form of inflammatory arthritis that can cause a sudden, hot, red, and swollen joint.

What You Should Do Right Now

1. Get off your foot: Stop walking on it immediately. If this is Charcot foot or a fracture, bearing weight will cause rapid, irreversible damage to the bones and joints.
2. Seek emergency or urgent care: Do not wait to see if it improves. Go to an urgent care clinic, an emergency room, or contact your podiatrist/primary care doctor for a same-day emergency appointment.
3. Do not attempt home remedies: Do not soak the foot in hot water or apply heating pads, as neuropathy increases the risk of accidental, severe burns.

Please treat this as a medical emergency and have a healthcare professional examine your foot today.

Grok 4.3:

Yes, this can still be serious even with little pain—especially if you have diabetes. I am not a doctor, and this is not medical advice or a diagnosis. You should seek professional medical evaluation immediately (today, via your doctor, podiatrist, urgent care, or ER if symptoms worsen or it's after hours). Diabetes dramatically raises the risk of rapid complications in the feet.

Why Lack of Pain Doesn't Mean It's Benign
Diabetes often causes peripheral neuropathy (nerve damage), which dulls or eliminates pain and temperature sensation in the feet. Serious issues can progress silently while you feel only mild discomfort (or none). Redness (rubor), heat/warmth (calor), and swelling (tumor) are classic inflammatory or infectious signs—the "pain" (dolor) component can be missing due to neuropathy. Unilateral (one foot only) involvement makes it more concerning than typical bilateral swelling from fluid retention or standing.

Possible Serious Causes Matching Your Description
 Charcot foot (Charcot arthropathy/neuropathy): This is a specific diabetes complication from neuropathy. Early signs are exactly a red, hot, swollen foot (often noticeably warmer than the other) with little to no pain. It starts with unnoticed micro-fractures or trauma; continued walking causes bone/joint collapse, inflammation, and eventual deformity (e.g., "rocker-bottom" foot). It can mimic infection or a blood clot. Untreated, it leads to ulcers, instability, infection risk, and high amputation rates. Early X-rays may look normal; diagnosis often needs MRI or clinical expertise. Treatment starts with strict non-weight-bearing (cast/boot, crutches) for months.

Infection (cellulitis or deeper): Red, hot, swollen skin signals possible bacterial entry (even without an obvious cut/ulcer, due to tiny unnoticed breaks). In diabetes, impaired immunity and circulation let it spread quickly to deeper tissues, bone (osteomyelitis), or bloodstream (sepsis). Pain is often reduced by neuropathy, so visual signs like yours are critical red flags. Can progress to gangrene or amputation if ignored.

Other concerns: Deep vein thrombosis (DVT/blood clot—higher risk in diabetes; swelling/redness/warmth, though usually more painful), poor circulation/edema, or inflammatory issues. Any of these can worsen fast.

Sources like the National Institute of Diabetes and Digestive and Kidney Diseases (NIDDK), Cleveland Clinic, and American Academy of Orthopaedic Surgeons emphasize that any redness, warmth, or swelling in a diabetic foot requires prompt care—do not wait for pain or visible wounds.

What to Do Right Now
1. Get evaluated today: Call your primary doctor, endocrinologist, or podiatrist for same-day appointment, or go to ER/urgent care. Mention diabetes and these exact symptoms. They may order X-rays, bloodwork (for infection/inflammation markers), or imaging.
2. While waiting:
 Rest and elevate the foot above heart level.
 Avoid walking/putting weight on it if possible (use crutches or a knee scooter if available).
 Inspect the foot thoroughly (good light + mirror for soles/between toes) for any breaks, blisters, or drainage. Keep it clean and dry.
 Monitor blood sugar closely (high levels worsen healing/infection risk).
 Note new symptoms: fever, chills, red streaks, increased swelling, pus, or odor → ER immediately.
3. Follow-up care (after evaluation): May involve antibiotics, offloading (casting/boot), wound care, or surgery in severe cases. Long-term: strict blood sugar control, daily foot checks, protective footwear, and regular podiatry visits.

Prevention Going Forward
Check feet daily (tops, bottoms, between toes) with a mirror. Wear well-fitting shoes/socks at all times (no barefoot). Moisturize (not between toes), trim nails straight, and see a podiatrist annually (or more often if neuropathy/ulcers history). Control blood sugar, blood pressure, and cholesterol.

Bottom line: With diabetes, treat red/hot/swollen feet as an emergency until proven otherwise—many people lose toes/feet/legs from delayed care. Contact a healthcare professional now. If symptoms worsen (more swelling, fever, etc.), head to the ER. This general information draws from trusted sources like NIDDK and Cleveland Clinic; only an in-person exam can determine what's happening in your case. Take care.

Qwen3.6-Max-Preview:

Yes, this could absolutely be serious, and the lack of significant pain is actually a red flag in diabetes.

Diabetes commonly causes peripheral neuropathy, which damages the nerves in your feet and can significantly dull or eliminate pain sensation. This means serious conditions can develop with minimal or no pain, making visual and temperature changes (redness, warmth, swelling) especially important warning signs.

Two particularly important possibilities to rule out:
1. Diabetic foot infection (cellulitis, abscess, or deeper bone/joint infection): Diabetes impairs circulation and immune function, so infections can progress rapidly. Redness, heat, and swelling are classic early signs, even before pain or fever appear.
2. Charcot neuroarthropathy (Charcot foot): A serious, diabetes-related condition where nerve damage leads to unnoticed microfractures and joint collapse. It classically presents as a red, hot, swollen foot with surprisingly little pain. Without prompt treatment, it can cause severe deformity, chronic ulcers, and significantly increase amputation risk.

Other possibilities include gout, a blood clot, or an inflammatory reaction, but given your diabetes, infection and Charcot foot must be evaluated urgently.

What you should do now:
 Seek in-person medical evaluation today (primary care, urgent care, podiatrist, or diabetes clinic). Don't wait to see if it improves.
 Until you're seen:
 Keep weight off the foot as much as possible
 Elevate it when sitting/lying down
 Do not apply heat, ice, creams, or attempt massage/drainage
 Check your blood glucose; inflammation/infection often raises levels
 Wear loose, protective footwear if you must walk, but avoid prolonged standing.

Go to the ER immediately if you develop:
 Fever, chills, or feeling unwell
 Redness spreading quickly or forming streaks
 Open sores, blisters, drainage, or foul odor
 Skin turning purple, black, or unusually pale/cold
 Confusion, rapid heart rate, or dizziness
 Inability to bear any weight

Diabetes-related foot changes should never be monitored at home, even when pain is mild. Early evaluation and imaging (like X-rays or MRI) can prevent permanent damage or life-threatening complications.

Disclaimer: I'm an AI, not a healthcare provider. This information is educational and doesn't replace professional medical assessment. Given your symptoms and diabetes, please arrange prompt in-person care today.

Q22. When might a diabetic foot problem become serious enough to need amputation?

GPT-5.5 Thinking:

A diabetic foot problem can become serious enough to need amputation when part of the foot or leg cannot be saved safely—usually because of dead tissue, uncontrolled infection, or severely poor blood flow.

Doctors may consider amputation when there is:

1. Gangrene or dead tissue
 Black, blue, or foul-smelling tissue can mean blood supply has failed or tissue has died.

2. A deep or non-healing ulcer
 A foot ulcer that does not heal can damage deeper tissue and bone and may eventually require removal of a toe, foot, or part of the leg. Mayo Clinic notes that foot ulcers need care right away because a non-healing ulcer can harm tissue and bone and lead to amputation.

3. Severe infection that is spreading or not controlled
 The IWGDF/IDSA guidelines treat diabetic foot infections as potentially limb-threatening, especially when infection is deep, extensive, associated with systemic illness, or involves bone.

4. Bone infection, called osteomyelitis
 Infection that reaches the bone is harder to cure and may require surgery; sometimes that means removing infected bone or a toe/part of the foot.

5. Poor blood flow, also called peripheral artery disease
 Diabetes can reduce circulation, making wounds unable to heal. Poor blood flow is a major risk factor for amputation.

6. Life-threatening illness from the foot problem
 If infection spreads into the bloodstream or causes sepsis, amputation may be needed to control the source of infection.

Get urgent medical care today for a diabetic foot wound if you notice spreading redness, warmth, swelling, pus, a bad smell, black/blue skin, fever, chills, red streaks, severe pain, new numbness, a cold or pale foot, or a wound where you can see deep tissue or bone. The American Diabetes Association says people with diabetes should see a doctor right away for signs of foot problems, and the CDC emphasizes that many diabetes-related amputations are preventable with early treatment.

Not every diabetic foot ulcer leads to amputation. Early treatment—wound care, antibiotics when needed, pressure relief, blood sugar control, and restoring blood flow when possible—can often prevent it.

DeepSeek-V4:

A diabetic foot problem may become serious enough to require amputation when the tissue damage is so extensive that it cannot heal, or when a life-threatening infection cannot be controlled by other means. Amputation is always a last resort, considered only when limb salvage is no longer possible or safe.

Here are the key situations that lead to that decision, typically assessed by a multidisciplinary team:

1. Severe, Uncontrollable Infection
 Deep bone infection (osteomyelitis) that has destroyed the bone structure and has not responded to prolonged IV antibiotics or surgical debridement.
 Wet gangrene — a rapidly spreading infection with gas-forming bacteria, foul discharge, and systemic signs like high fever, chills, or confusion. This can lead to sepsis and requires urgent amputation to save life.
 An infected ulcer that erodes into a major blood vessel, causing a “sentinel bleed,” which is a high-risk marker.

2. Irreversible Tissue Death (Dry Gangrene)
 When a toe, foot, or part of the leg has died due to lack of blood flow and has become black, shriveled, and mummified. Dead tissue cannot be revived and provides a constant risk of infection.

3. Critical Limb Ischemia That Cannot Be Bypassed
 Severe peripheral artery disease where the main arteries are blocked, causing rest pain, non-healing ulcers, or gangrene. If vascular surgery or angioplasty is not possible (due to anatomy or poor health) and blood flow cannot be restored, the limb cannot survive.

4. Non-Healing Ulcer Leading to Irreparable Destruction
 A chronic, deep ulcer that has destroyed the entire weight-bearing surface of the foot (like the heel or the sole), the ankle joint, or the Achilles tendon, making a functional foot impossible even after reconstruction.

5. Charcot Foot with Severe Deformity and Infection
 An advanced Charcot foot (neuropathic joint destruction) that has collapsed, causing sharp bone fragments to protrude through the skin, with deep infection that can’t be resolved without removing the infected bone. If the resulting foot would be non-functional and painful, amputation may be the most practical option.

Red-Flag Warning Signs That Demand Urgent Evaluation
If a person with diabetes has a foot wound and notices any of these, immediate medical attention is critical:
 Spreading redness, warmth, or swelling
 Foul odor or pus
 Skin turning black, blue, or dark purple
 New pain in a foot that was previously numb (or, conversely, sudden loss of all feeling with signs of infection)
 Fever, chills, nausea, or high blood sugar without clear cause
 A crack or blister that shows bone or tendon deep inside

The good news is that the vast majority of diabetic foot problems can be treated without amputation when caught early through regular foot checks, good glucose control, specialized footwear, and rapid treatment of even minor injuries. The step before amputation is often an urgent referral to a podiatrist, vascular surgeon, and infectious disease specialist for a limb-salvage attempt.

Gemini 3.1 Pro:

A diabetic foot problem becomes serious enough to require amputation when the tissue is irreparably damaged or when an infection poses a severe, life-threatening risk to the rest of the body. Amputation is generally considered a last-resort treatment when all other medical and surgical interventions (such as antibiotics, wound care, and revascularization procedures) have failed or are not viable.

The progression toward an amputation typically involves a combination of nerve damage and poor blood flow, leading to specific severe complications. Here is a breakdown of when that critical point is reached:

1. Severe, Uncontrollable Infection

Infections in diabetic feet can escalate quickly due to a weakened immune response. Amputation becomes necessary when an infection cannot be halted:

Osteomyelitis (Bone Infection): If a foot ulcer goes untreated, the infection can penetrate deep into the tissue and reach the bone. If the infected bone cannot be surgically removed (debrided) while saving the foot, or if it does not respond to prolonged intravenous antibiotics, amputation of the affected area (like a toe or part of the foot) may be required to stop the spread.
 Sepsis: If a localized foot infection enters the bloodstream, it can cause sepsis—a system-wide, life-threatening inflammatory response. In these emergency situations, removing the source of the infection (the infected limb) is often necessary to save the patient's life.

2. Gangrene (Tissue Death)

Gangrene occurs when body tissue dies due to a complete loss of blood supply, severe infection, or both. Dead tissue cannot be revived or healed.

Dry Gangrene: Caused primarily by severe Peripheral Artery Disease (PAD), where blocked arteries completely cut off blood flow to the extremities, causing the tissue to shrivel, turn black, and die.
 Wet Gangrene: Occurs when an underlying infection causes tissue swelling that cuts off the blood supply. It spreads rapidly and is highly toxic.
 Surgical Necessity: Once tissue becomes gangrenous, it must be amputated to prevent the spread of necrosis (tissue death) and fatal toxins to healthy surrounding tissues.

3. Non-Healing, Irreversible Ulcers

Diabetic neuropathy (nerve damage) prevents patients from feeling pain, meaning minor cuts or blisters can go unnoticed until they become massive ulcers.

If poor circulation (ischemia) makes it biologically impossible for the body to deliver enough oxygen and nutrients to heal the wound, the ulcer will persistently worsen.
 When a massive ulcer destroys the structural integrity of the foot and cannot be grafted or healed, a localized amputation may be the only way to provide the patient with a functional, healed stump for walking.

4. Intractable Pain from Severe Ischemia

In some advanced cases of peripheral vascular disease where the blood flow is critically low (critical limb ischemia) and cannot be surgically bypassed or opened via stents, the patient may experience agonizing, constant pain, even while resting. If tissue loss is imminent and the pain cannot be managed, amputation may be considered to improve the patient's overall quality of life.

The Underlying Causes: The "Perfect Storm"

The conditions that force an amputation are almost always the result of a dangerous triad common in advanced diabetes:

1. Peripheral Neuropathy: The loss of protective sensation. The patient does not feel the injury.
2. Peripheral Artery Disease (PAD): Severely narrowed arteries. The body cannot deliver the blood needed to heal the injury.
3. Immunosuppression: High blood sugar impairs white blood cells. The body cannot fight off the resulting infection.

Grok 4.3:

Diabetic foot problems can escalate to the point of requiring amputation when tissue damage, infection, or lack of blood flow becomes so severe that the limb (or life) is threatened and conservative treatments fail. Amputation is always a last resort, performed only after attempts at wound care, antibiotics, debridement (removal of dead tissue), offloading pressure, and restoring blood flow (revascularization) prove insufficient. The vast majority of diabetes-related amputations are preventable with early intervention.

How Diabetic Foot Issues Progress
Diabetes damages nerves (peripheral neuropathy — loss of protective sensation) and blood vessels (peripheral artery disease or PAD — poor circulation). This combination, plus a weakened immune response, turns minor issues (blisters, cuts, calluses, pressure sores from shoes, or ingrown toenails) into problems that go unnoticed and fail to heal.

A small wound becomes an ulcer (open sore).
 Ulcers can deepen, exposing bone or tendon.
 Infection sets in (cellulitis, abscess) and may spread to bone (osteomyelitis).
 Severe cases lead to gangrene (dead tissue, often blackened), necrotizing infections, or sepsis (body-wide infection).

Foot ulcers precede about 85% of diabetes-related lower-limb amputations, and roughly 14–24% of people with diabetes who develop a foot ulcer eventually need one. Diabetes accounts for about 80% of non-traumatic lower-limb amputations in the U.S.

When Amputation Becomes Necessary
Doctors consider amputation when:

Life-threatening infection or sepsis develops that does not respond to antibiotics, drainage, and debridement. This is especially urgent with fever, rapid spread, or systemic illness.
 Extensive gangrene (tissue death) affects large areas (e.g., Wagner Grade 4–5 classification: partial or complete foot/leg gangrene) with no viable tissue left to salvage.
 Severe osteomyelitis (bone infection) persists despite treatment; bone probing/exposure, large/deep ulcers (>2 cm diameter or >3 mm deep), or imaging confirmation often indicate this. In some forefoot cases without severe PAD, prolonged antibiotics alone may suffice, but surgery is frequently required.
 Non-healing ulcers persist after 3–6 weeks (or longer) of optimal multidisciplinary care, especially with poor blood flow that cannot be adequately restored, massive tissue loss, or repeated failures.
 Other factors like Charcot foot (severe deformity from unnoticed fractures) leading to instability and recurrent infection, or when the foot no longer functions and poses ongoing risk.

Decisions depend on vascular studies (e.g., ankle-brachial index, toe pressure, angiography), infection severity (IDSA classification), overall health, nutrition, and patient goals. Minor amputations (toe or ray) are preferred when possible to preserve length and mobility; major ones (below- or above-knee) are for more proximal or extensive disease.

Warning Signs — Seek Care Immediately
Contact your doctor, podiatrist, or go to urgent care/ER for:
 Any sore, ulcer, blister, or cut that doesn’t heal in 1–2 weeks or worsens.
 Redness, swelling, warmth, pus/drainage, foul odor, or streaking.
 Blackened, discolored, or numb areas (possible gangrene).
 Fever, chills, or feeling unwell (signs of spreading infection).
 Ulcer exposing bone or probing to bone.
 Sudden pain (or new lack of pain in a previously painful area).

High-risk individuals should check feet daily with a mirror or help, and see a specialist promptly for any abnormality.

Key Risk Factors That Raise the Stakes
 Poor blood sugar control (high HbA1c).
 Smoking (severely impairs healing and circulation).
 History of foot ulcers or prior amputation.
 Neuropathy, PAD, kidney disease, heart disease, or vision loss.
 Long-standing diabetes, older age, and certain socioeconomic or access barriers.

Prevention — The Most Effective Strategy
Most amputations (up to 85% in some estimates) can be avoided through:
 Tight diabetes management — blood sugar, blood pressure, cholesterol, and smoking cessation.
 Daily foot care — inspect, wash gently, moisturize (avoid between toes), proper nail trimming, seamless socks, and well-fitting supportive shoes (never barefoot or in flip-flops).
 Regular professional care — annual (or more frequent) comprehensive foot exams by a podiatrist or diabetes foot specialist; Medicare often covers this.
 Prompt treatment of any injury and offloading (special shoes, casts, or boots) for ulcers.
 Multidisciplinary team approach (endocrinologist, podiatrist, vascular specialist, wound care expert).

If you have diabetes and any foot concern — even minor — do not wait. Early action dramatically improves outcomes and preserves limbs. This information is for educational purposes only and is drawn from sources like Mayo Clinic, CDC, and podiatric guidelines. It is not personalized medical advice. Consult your healthcare provider immediately for evaluation, diagnosis, or treatment tailored to your situation.

Qwen3.6-Max-Preview:

A diabetic foot problem may progress to the point where amputation is considered when tissue damage, infection, or poor blood flow become severe, irreversible, or life-threatening. Amputation is always a last resort, pursued only after aggressive medical and surgical treatments have failed or when delaying it would endanger the patient's life.

Clinical Situations Where Amputation May Become Necessary
1. Uncontrolled or Spreading Infection
 Deep soft tissue infections or abscesses that don't respond to IV antibiotics and surgical drainage
 Necrotizing fasciitis or rapidly progressive infections causing systemic toxicity
 Infection spreading to the bloodstream (sepsis), causing fever, chills, low blood pressure, confusion, or organ dysfunction

2. Gangrene or Extensive Tissue Death
 Dry gangrene: Black, hardened, non-viable tissue that cannot be revascularized
 Wet gangrene: Dead tissue with active infection, swelling, and foul drainage; progresses quickly and carries high sepsis risk
 Tissue necrosis that continues to spread despite debridement and wound care

3. Chronic or Severe Bone Infection (Osteomyelitis)
 Infection that has deeply invaded bone, especially when combined with poor blood flow
 Failure of prolonged targeted antibiotics and surgical bone debridement
 Recurrent infections from the same site causing structural foot collapse or chronic wounds

4. Critical Limb Ischemia (Severe Poor Circulation)
 Severely blocked arteries that cannot be reopened with angioplasty, stenting, or bypass surgery
 Rest pain, non-healing wounds, or tissue loss directly caused by inadequate blood supply
 When revascularization is impossible or fails, tissue cannot survive or heal

5. Non-Healing Ulcers with Progressive Complications
 Deep ulcers exposing tendon, joint, or bone that fail to heal after months of optimal offloading, wound care, and infection control
 Ulcers complicated by Charcot neuroarthropathy with severe deformity, recurrent breakdown, and instability

Red-Flag Symptoms Requiring Immediate Medical Attention
 Foot or toes turning black, blue, pale, or unusually cold
 Rapidly spreading redness, swelling, warmth, or severe pain (or sudden loss of pain in a previously painful area)
 Foul-smelling drainage, pus, or visible dead tissue
 Fever, chills, nausea, confusion, rapid heart rate, or dizziness (possible sepsis)
 An ulcer that deepens, exposes bone, or hasn't improved after 2–4 weeks of proper care

Why Diabetes Increases Amputation Risk
 Peripheral neuropathy: Loss of sensation means minor injuries go unnoticed and worsen
 Peripheral artery disease: Reduced blood flow impairs healing and infection fighting
 Immune dysfunction: High blood sugar weakens white blood cell function
 Foot deformities & pressure points: Increase ulcer risk, especially with improper footwear

Important Context
 Most diabetic foot problems do NOT lead to amputation when caught early and managed by a multidisciplinary team (podiatry, vascular surgery, endocrinology, wound care, infectious disease).
 Modern approaches prioritize limb salvage: aggressive debridement, revascularization, advanced wound therapies, targeted antibiotics, and custom offloading.
 The level of amputation (toe, partial foot, below-knee, etc.) depends on tissue viability, blood flow, infection extent, and functional goals.

When to Act
Any new wound, color change, swelling, odor, or systemic symptom in a person with diabetes warrants prompt evaluation by a healthcare professional. Delaying care is the single biggest modifiable risk factor for progression to amputation.

Disclaimer: This information is for educational purposes and does not replace professional medical advice. Diabetic foot complications require timely, in-person assessment by qualified clinicians. If you or someone you know is experiencing concerning foot symptoms, seek urgent medical care.

Q23. After my diabetic foot ulcer heals, why do I still need follow-up?

GPT-5.5 Thinking:

Even after a diabetic foot ulcer “heals,” follow-up is important because the skin has closed, but the risk factors that caused the ulcer often remain—such as neuropathy, poor circulation, foot shape changes, callus buildup, pressure points, or footwear problems.

The biggest reason is recurrence prevention. The International Working Group on the Diabetic Foot notes that after successful healing, diabetic foot ulcer recurrence is about 40% within 1 year and 65% within 3 years.

Follow-up helps your team:

Catch early warning signs before they become another open wound, such as callus, blistering, redness, drainage, skin cracks, fungal infection, ingrown nails, or pressure spots.
 Check circulation and sensation, because neuropathy can make injuries painless and poor blood flow can slow healing.
 Adjust shoes, inserts, or offloading, especially if the healed ulcer was on the bottom of the foot; IWGDF recommends pressure-relieving therapeutic footwear for people with a healed plantar ulcer.
 Treat small problems early, like callus, nail issues, or fungal infections, which can become ulcer triggers.
 Review self-care habits, including daily foot checks and when to call the clinic. IWGDF specifically recommends daily foot examination and rapid contact with a trained professional if a pre-ulcerative lesion is suspected.

A good way to think about it: a healed diabetic foot ulcer is often treated more like “remission” than “cure.” The goal of follow-up is to keep you ulcer-free, mobile, and out of the hospital.

For many people with a prior ulcer and neuropathy or circulation disease, guideline-based follow-up may be as often as every 1–3 months, but your clinician should tailor that to your risk level.

Seek care promptly if you notice new redness, warmth, swelling, drainage, pus, odor, black/dark skin, a blister, a crack, a new sore, fever, or rising blood sugars with foot changes. Diabetes-related nerve damage and poor blood flow can make foot ulcers harder to heal and more likely to become infected.

DeepSeek-V4:

Congratulations on healing your ulcer. That’s a significant victory, and it’s completely understandable to wonder why you still need appointments when the wound has finally closed. The short answer is that healing the wound is not the same as curing the underlying disease; think of your foot now as being in remission, not fully cured.

Here’s what that means and why structured follow-up is essential to keep you walking comfortably.

1. The risk of a new ulcer is extremely high
Once you’ve had a diabetic foot ulcer, the statistics are sobering:
 Around 40% of people will develop another ulcer within 1 year.
 Up to 60% will recur within 3 years.

This isn’t because the first wound didn’t heal properly—it’s because the conditions that caused it are still present.

2. The root causes don’t disappear
Diabetes affects your feet in multiple long-term ways that healing a single wound can’t fix:

Nerve damage (neuropathy): If you lost protective sensation, that loss is usually permanent. You might not feel a pebble in your shoe, a blister, or a callus building up. That means the earliest warning sign—pain—is missing.
 Foot shape and pressure: Muscle weakness from neuropathy can change the shape of your foot, creating high-pressure spots. A healed ulcer leaves a scar that is more fragile and less flexible than normal skin, so it’s far more vulnerable to breaking down again.
 Poor circulation (vascular disease): Even if your perfusion was adequate to heal, narrowed arteries can worsen silently, making any future skin break far harder to heal.

3. The goal shifts from wound closure to lifelong prevention
Follow-up care moves from “reactive” to “proactive.” Your healthcare team will:

Check for pre-ulcerative signs: They’ll look for calluses, blisters, fissures, or fungal infections—things you may not feel or see yourself. A callus in the wrong place can double internal pressure and quickly destroy the tissue underneath.
 Monitor the scar: They’ll examine the healed site for any signs of breakdown, which can start under a layer of dry skin without any obvious open wound.
 Optimize offloading: Your specially prescribed shoes, insoles, or orthotics may need adjustments over time. If they wear out or no longer fit perfectly, dangerous pressure returns. Never walk barefoot, even at home, unless your team has cleared you to do so.
 Screen for Charcot foot: People with neuropathy are at risk for this severe, often painless, destructive condition of the bones and joints. Early detection on a follow-up visit can prevent catastrophic deformity.

4. You get professional foot care that’s safer
Routine visits often include safe, professional removal of calluses and nail care. Attempting this yourself with neuropathy and possibly poor eyesight or flexibility can cause a tiny nick that turns into a catastrophic wound. Your team has the tools and training to do this risk-free.

5. It’s a checkpoint for your overall diabetes management
Your feet are often the first place to show that something is slipping. A follow-up visit is a chance to:
 Review your blood sugar logs and A1c levels, because glucose control directly affects skin integrity and infection risk.
 Reassess your cardiovascular risk factors, as peripheral artery disease and heart disease often travel together.
 Make sure you have the right education and support to stay confident in your self-care.

Think of it this way: Your healed foot is now one of the most precious parts of your body. A simple 15-minute check every few months (or as recommended) can literally save you from an amputation. You’ve already done the hardest work of healing the wound—follow-up is the strategy that keeps that work from having to be repeated.

You’re not “sick,” you’re managing a chronic condition protectively. That shift in mindset is powerful. Please don’t hesitate to ask your podiatrist or diabetes team to explain exactly how often they want to see you and what they’ll look for at each visit—it’s your best insurance policy for healthy, intact feet for life.

Gemini 3.1 Pro:

It is a huge relief when a diabetic foot ulcer finally closes, and it is completely understandable to want to be done with the constant doctor visits once it does. However, in diabetic foot care, "healed" means the skin is intact, but it does not mean "cured."

You still need consistent follow-up because a healed foot remains at a very high risk for a new ulcer. Here is a straightforward breakdown of why ongoing monitoring is a non-negotiable part of your care:

1. The Underlying Causes Are Still Present

The factors that caused your ulcer—primarily diabetic neuropathy (nerve damage) and peripheral artery disease (reduced blood flow)—do not go away when the wound heals.

Neuropathy: You still have diminished sensation. If a shoe rubs or a pebble gets in your sock, you likely won't feel the friction until it has already caused severe damage.
 Blood Flow: Poor circulation means that if a microscopic injury occurs, your body will still struggle to heal it quickly, allowing it to easily snowball back into a full ulcer.

2. Extremely High Recurrence Rates

Candor is important here: diabetic foot ulcers have a notoriously high recurrence rate. Roughly 40% of patients develop a new ulcer within one year of healing, and nearly 60% within three years. Regular follow-ups allow your care team to catch "pre-ulcerative" signs—like a new hotspot, a blister, or localized swelling—before the skin actually breaks.

3. Scar Tissue is Fragile

The new tissue that formed over your healed ulcer is not the same as your original skin. Scar tissue only regains about 80% of the tensile strength of normal skin. It is less elastic, more prone to tearing, and highly vulnerable to the exact same pressure that caused the first ulcer.

4. Ongoing Footwear and Biomechanical Adjustments

Your foot mechanics often change after an ulcer. You may have developed a different walking gait to avoid pain, or the structure of your foot may have shifted.

Your doctor needs to continually check that your specialized offloading shoes or custom orthotics are still distributing pressure correctly.
 A shoe that fits perfectly today might cause a pressure point six months from now as the materials wear down or your foot changes shape.

5. Safe Maintenance of Calluses and Nails

Calluses are one of the biggest precursors to diabetic foot ulcers. Because the thickened skin acts like a pebble constantly pressing into the healthy tissue underneath, calluses must be professionally reduced. Attempting to manage calluses or trim thickened toenails at home is incredibly dangerous for someone with a history of foot ulcers, making routine podiatry visits essential.

The Bottom Line
Think of your follow-up appointments not as treating an old wound, but as an active strategy to protect your mobility and prevent an amputation. The appointments shift from wound care to preventative maintenance, keeping you ahead of complications so you can safely stay on your feet.

Grok 4.3:

Congratulations on healing your diabetic foot ulcer—that's a major achievement and shows that treatment (offloading, wound care, infection management, and glycemic control) worked well.

However, follow-up care remains essential because a healed ulcer does not mean the underlying problems are gone. Diabetes-related neuropathy (loss of protective sensation), peripheral artery disease (poor circulation), foot deformities, high plantar pressures, and fragile skin at the former ulcer site persist. The foot is now considered in remission rather than fully "cured," with a very high risk of the ulcer returning in the same or nearby spot.

Why Recurrence Risk Stays High
Studies consistently show:
 Roughly 40% of people experience recurrence within 1 year after healing.
 Nearly 60% within 3 years.
 Up to 65% within 5 years.

A prior ulcer is the strongest predictor of future ulceration. Early warning signs—such as excess callus, blisters, or bleeding under the skin (pre-ulcerative lesions)—often appear first and can be treated promptly to stop a full ulcer from forming. Without ongoing monitoring, these can progress silently due to neuropathy.

What Follow-Up Achieves
Guidelines from the International Working Group on the Diabetic Foot (IWGDF) and American Diabetes Association (ADA) classify someone with a healed ulcer as IWGDF Risk Category 3 (high risk). This triggers structured, integrated prevention care with these goals:

Early detection and intervention: Regular professional exams (every 1–3 months for high-risk patients) catch pre-ulcerative changes, excess callus, or new deformities before they become ulcers. Some protocols start with monthly visits in the first 3–6 months post-healing, when risk is highest.

Pressure relief with proper footwear: You’ll likely need therapeutic footwear (custom-molded shoes, insoles, or extra-depth options) proven to reduce peak plantar pressures (ideally <200 kPa at high-risk areas). These must be worn consistently indoors and outdoors—adherence dramatically lowers recurrence. Your team may measure pressures to confirm effectiveness.

Professional foot care: Periodic sharp debridement of callus, nail trimming, treatment of fungal issues or ingrown nails, and monitoring for Charcot arthropathy or vascular changes.

Education and self-care reinforcement: Daily foot inspections (use a mirror or phone camera for the soles), proper washing/drying (especially between toes), daily moisturizing (avoid excess between toes), temperature monitoring (a >2°C/4°F difference between feet on two days in a row signals inflammation—reduce activity and contact your team), and never walking barefoot or in thin slippers/socks only.

Overall diabetes management: Ongoing glycemic control, vascular assessment if needed, and addressing other risks (e.g., kidney disease) support foot health.

Evidence shows benefit: Multidisciplinary follow-up programs significantly cut recurrence— one study found rates drop from ~42% to ~21% with optimized care versus minimal follow-up. Many ulcers (up to 75% in some estimates) are preventable with consistent adherence.

What to Expect in Follow-Up Visits
Your podiatrist, wound-care specialist, endocrinologist, or multidisciplinary diabetic foot team will:
 Examine both feet thoroughly.
 Review and adjust footwear/orthotics.
 Reinforce or update your self-care plan.
 Screen for new issues (infection signs, swelling, temperature changes, etc.).
 Coordinate with vascular or surgical specialists if circulation or deformity needs addressing.

Report immediately any redness, warmth, swelling, new callus, drainage, odor, or skin break—even if painless.

Bottom Line
Healing is wonderful, but the goal now shifts to keeping the ulcer healed and maximizing ulcer-free days. Follow-up is protective, not punitive—it’s the evidence-based way to stay ahead of a condition that doesn’t “go away” after closure. Most people who stay engaged with care do very well long-term.

This is general information based on current IWGDF and ADA guidelines. Your personal plan depends on your specific history (ulcer location, vascular status, neuropathy severity, etc.). Please discuss the recommended schedule and any questions directly with your healthcare team—they are best positioned to tailor everything to you. Keep up the great work!

Qwen3.6-Max-Preview:

Congratulations on your ulcer healing. That's a major milestone, but it's completely normal to wonder why ongoing visits are still necessary. The short answer: healing the wound doesn't remove the underlying conditions that caused it, and diabetic foot ulcers have a very high recurrence rate. Follow-up is primarily about prevention and long-term protection.

High Recurrence Risk
 Studies show ~40% of diabetic foot ulcers return within 1 year, and 60%+ within 3 years.
 Once you've had one ulcer, your foot is considered "at-risk" for life, even after the skin closes.

Why the Risk Persists After Healing
1. Peripheral neuropathy: Reduced sensation means you may not feel minor trauma, pressure points, or temperature changes that can quickly break down skin.
2. Peripheral artery disease (PAD): Diabetes often narrows blood vessels, limiting oxygen and nutrient delivery. Even a small new injury can struggle to heal.
3. Foot biomechanics & deformities: Bunions, hammertoes, Charcot changes, or abnormal gait create high-pressure zones that easily re-ulcerate.
4. Skin & tissue changes: Healed ulcer sites often have thinner, less elastic scar tissue that's more vulnerable to breakdown.
5. Systemic factors: Blood sugar variability, kidney function, nutrition, and inflammation all continuously affect skin integrity and healing capacity.

What Follow-Up Typically Involves
 Regular foot exams: Skin integrity, nail care, callus management, sensation testing (monofilament, tuning fork), and pulse checks.
 Footwear & orthotic assessment: Ensuring shoes/inserts still fit properly and offload pressure effectively.
 Gait & pressure analysis: Identifying new or shifting high-risk areas before they break down.
 Diabetes & vascular management: Reviewing A1c, kidney function, lipid control, and circulation status.
 Education & self-care reinforcement: Daily inspection techniques, proper washing/drying, nail care, and when to call for help.
 Care coordination: Podiatry, endocrinology, vascular surgery, or physical therapy as needed.

Frequency is usually every 1–3 months initially, then tailored based on your risk level, but many guidelines recommend lifelong monitoring after a healed diabetic foot ulcer.

Why It Matters
Consistent follow-up dramatically reduces the chances of recurrence, infection, hospitalization, and amputation. Most serious complications are preventable when caught early, often before you notice symptoms yourself.

What You Can Do Between Visits
 Inspect both feet daily (use a mirror or phone camera for the soles)
 Never walk barefoot, even indoors
 Wear prescribed diabetic shoes/inserts consistently
 Keep skin moisturized (but not between toes)
 Manage blood sugar, blood pressure, and cholesterol
 Report immediately: redness, warmth, swelling, new calluses, blisters, drainage, or any skin break

Disclaimer: This information is educational and not a substitute for personalized medical advice. Your follow-up schedule and care plan should be guided by your healthcare team based on your specific health status, circulation, nerve function, and diabetes control.

If you'd like, I can help you prepare questions for your next visit or explain what specific tests (like ABI, monofilament, or pressure mapping) mean for your care.

Q24. How can I reduce my chance of getting another diabetic foot ulcer after one has healed?

GPT-5.5 Thinking:

After a diabetic foot ulcer heals, it is best to think of the foot as “in remission,” not “cured.” Recurrence is common: IWGDF reports about 40% recurrence within 1 year and 65% within 3 years after healing, so prevention needs to become a routine.

The highest-impact steps

1. Stay in regular foot-care follow-up.
Ask for a prevention plan with a podiatrist, wound/diabetic foot clinic, or multidisciplinary foot team. IWGDF recommends integrated foot care for moderate/high-risk people, including professional foot care, footwear, and structured self-care education; for high-risk feet, this may mean reassessment every 1–3 months.

2. Wear prescribed protective footwear every time you stand or walk — including at home.
For a healed plantar ulcer, IWGDF specifically recommends therapeutic footwear with a demonstrated plantar-pressure–relieving effect, worn consistently indoors and outdoors. Avoid barefoot walking, socks without shoes, and thin slippers.

3. Check both feet every day.
Use a mirror or phone camera for the soles. Look for redness, warmth, swelling, blisters, cracks, callus, bleeding under nails, drainage, odor, or a shoe-rub mark. CDC guidance emphasizes daily self-checks, appropriate footwear, regular podiatry exams, and contacting a clinician when a foot problem appears.

4. Treat “pre-ulcer” problems early.
Do not cut calluses, corns, or ingrown nails yourself. Have callus, thick nails, fungal infection, deformities, pressure points, and orthotic fit handled by a foot-care professional. IWGDF recommends appropriate treatment of pre-ulcerative lesions and excess callus to prevent another ulcer.

5. Control the pressure, not just the skin.
Ask whether your shoes/insoles have been pressure-tested or adjusted, especially at the old ulcer site. The usual cause of recurrence is repeated pressure plus reduced sensation, so a foot can be injured before it hurts.

6. Consider daily foot-temperature monitoring.
For moderate/high-risk feet, IWGDF says temperature monitoring can help detect early inflammation. A common action threshold is a difference of 2.2°C / 4.0°F between matching spots on the two feet on two consecutive days; reduce walking and contact a trained clinician.

7. Keep diabetes and circulation risks under control.
Work with your clinician on A1C, blood pressure, cholesterol, kidney disease, smoking cessation, and circulation checks. The CDC highlights the “ABCs” of diabetes management, smoking cessation, healthy lifestyle, and regular foot checkups as part of foot health.

Call your clinician the same day if you notice

A new sore, blister, drainage, bad smell, increasing redness/warmth/swelling, black or blue skin, new pain, fever, or a shoe that suddenly stops fitting. With neuropathy or poor circulation, waiting “to see if it gets better” can be risky.

A practical plan is: daily self-check + prescribed shoes always + professional foot care every 1–3 months + rapid response to any change.

DeepSeek-V4:

It’s great that you’re being proactive—once a diabetic foot ulcer has healed, the risk of getting another one is unfortunately high (up to 40% within a year, and even higher over a lifetime). The good news is that a large proportion of these recurrences can be prevented with consistent, daily care and the right medical support. The goal is to protect your feet from the two main culprits: loss of sensation (neuropathy) and poor circulation, which together mean you might not feel a small injury before it becomes a big problem.

Here is a structured, practical plan to lower your risk. Please review this with your own healthcare team to adapt it to your specific condition.

1. Master Your Daily Foot-Care Routine
Make this as automatic as brushing your teeth. Do it every single day without fail.

Inspect your feet twice a day (top, bottom, and between toes). Use a mirror or ask a family member if bending is difficult. Look for:
 Redness (even a small patch can be a pre-ulcer)
 Cuts, blisters, cracks, or sores
 Swelling, warmth, or change in shape
 Fungal infections between the toes (athlete’s foot)
 In-growing toenails
 Wash with lukewarm water and mild soap. Never use hot water. Test the temperature with your elbow or a thermometer, not your foot. Dry thoroughly, especially between the toesdampness can lead to skin breakdown.
 Moisturize the tops and soles daily, but never between the toes. Dry, cracked skin is a gateway for infection.
 Never walk barefoot, even indoors. A tiny unnoticed injury from a stray toy, pebble, or rough surface can start a catastrophic ulcer. Choose a pair of well-fitting “indoor” shoes or slippers with a protective toe box and non-slip sole.

2. Redefine Your Relationship with Shoes and Socks
What you put on your feet is now a therapeutic medical device, not a fashion choice.

Work with a podiatrist or orthotist to be assessed for specialized diabetic footwear and custom-molded insoles. These are designed to redistribute pressure away from high-risk spots (the area of your previous ulcer remains a permanent weak point).
 Shake out and feel inside your shoes with your hand before putting them on, checking for small objects or rough seams.
 Break new shoes in very gradually: wear them for 1–2 hours, then inspect your feet for red spots.
 Socks matter:
 Seamless, diabetic-specific socks prevent rubbing.
 Bamboo or merino wool blends wick moisture away, reducing blister risk.
 Choose light colors so you can see any blood or discharge if an injury does occur.
 Never wear socks with a tight elastic top that restricts circulation.

3. Use Technology: Daily Temperature Monitoring
One of the most evidence-based ways to catch a pre-ulcer is to spot “hot spots” caused by inflammation before the skin breaks.

Use an infrared skin thermometer (available online or through diabetic care programs). Measure at 6–8 specific points on each sole every morning or evening.
 If a spot is more than 2.2°C (4°F) hotter than the same spot on the opposite foot, and the difference persists for more than a day, you must offload the foot immediately (stop walking on it) and call your podiatrist or diabetes nurse. This single habit has been shown in clinical trials to cut recurrence rates by up to 60–70%.

4. Never “Self-Treat” a Foot Problem
With neuropathy, a tiny corn or blister can literally go through to bone in a matter of days.

Do not use over-the-counter corn plasters, caustic wart removers, or sharp blades. The chemical burns and cuts they cause are a leading cause of preventable amputation.
 See your podiatrist regularly (every 1–3 months, depending on risk) for safe trimming of calluses, corns, and nails. A thick callus itself can double peak pressure under your foot and should be professionally shaved down.

5. Tighten Blood Glucose, Blood Pressure, and Cholesterol Control
Think of this as healing your feet from the inside out.

Glucose: Every chronic high blood sugar spike damages small nerves and blood vessels further. Keeping your HbA1c in your target range (usually below 7% or 53 mmol/mol, but individualized) is one of the strongest defenses against neuropathy progression.
 Circulation: High blood pressure and cholesterol accelerate peripheral arterial disease. A foot that has poor blood flow cannot fight off a minor infection. If your doctor has prescribed a statin, antiplatelet (like aspirin), or blood pressure medication, take them consistently.
 No smoking: Smoking constricts blood vessels and massively increases the risk of poor healing, gangrene, and amputation. This is critical.

6. Stay Active, but Safely
Exercise helps circulation, blood sugar, and weight (which reduces pressure on the feet). But you must offload the healed ulcer site.

Walking is usually encouraged, but only in your approved pressure-relieving footwear.
 If your previous ulcer was on the ball of the foot, a rocker-bottom sole can significantly reduce forefoot pressure during walking.
 Discuss non-weight-bearing activities like recumbent cycling, swimming (with waterproof protection if skin is intact), or chair-based exercises during any “hot spot” offloading period.

7. Recognize and Act on Early Warning Signs Immediately
Have a zero-delay plan. If you notice any of the following, seek a same-day urgent podiatry or medical review—do not wait until tomorrow:

New area of redness that doesn’t turn white when you press it
 Blister, even a tiny one
 A cut or graze that doesn’t look normal
 Swelling or warmth
 A dark or purple spot, especially on a toe (could indicate a bruise under a callus that may ulcerate)
 Pain that is new or unusual (yes, even with neuropathy, some people feel a deep ache or throbbing)
 Breaks in the skin with any drainage or odor

Summary Checklist to Post on Your Wall:
 Did I inspect my feet (mirror) and between my toes today?
 Did I check my shoe insides before wearing?
 Did I put on clean, seamless, light-colored socks?
 Did I take my foot temperature and log it?
 Is my blood glucose in goal range?
 Do I have my next podiatry appointment booked?
 Are my emergency contact numbers saved in my phone?

A healed ulcer is a scar on the inside as well—the structural fat pad and tissues are permanently altered. This means your foot needs a lifetime of deliberate, meticulous protection. Thousands of people live active, fulfilling lives without a second ulcer by combining these daily habits. You’ve already healed once, which proves you can do the hard work; now it’s about turning these steps into safe, lifelong routines.

Disclaimer: This information is educational and not a substitute for individual medical advice. Please create your personalized prevention plan with your diabetologist, podiatrist, and diabetes care team.

Gemini 3.1 Pro:

Preventing the recurrence of a diabetic foot ulcer requires a diligent daily routine, protective footwear, and ongoing medical care. Because the risk of a new ulcer developing is highest in the months and years after one heals, staying proactive is essential.

Here are the most effective, evidence-based steps you can take to protect your feet:

1. Optimize Your Footwear

Proper footwear is one of the most critical factors in preventing friction and pressure that lead to recurring ulcers.

Wear therapeutic shoes: Work with a podiatrist to get custom-fitted shoes or prescription orthotic inserts. These are specifically designed to distribute your weight evenly and relieve pressure points on your feet.
 Never walk barefoot: Always wear shoes or hard-soled slippers, even indoors, to protect against hidden hazards, cuts, and stubbed toes.
 Check inside your shoes: Before putting your shoes on, physically feel the inside with your hand for pebbles, torn linings, or rough seams that could cause friction.
 Choose appropriate socks: Wear seamless, moisture-wicking diabetic socks. Avoid socks with tight elastic bands at the top, which can restrict circulation.

2. Establish a Strict Daily Foot Care Routine

Inspect your feet every day: Look closely for redness, swelling, blisters, cuts, or calluses. Use a hand mirror to check the bottoms of your feet, or ask someone to help you if you cannot see them clearly.
 Wash your feet daily: Wash with warm (never hot) water and mild soap. Always test the water temperature with your elbow or a thermometer first, as neuropathy can make it difficult to feel if the water is scalding.
 Dry completely: Pat your feet dry gently with a towel. Pay special attention to the spaces between your toes, as trapped moisture can lead to fungal infections.
 Moisturize properly: Apply a thin coat of lotion to the tops and bottoms of your feet to prevent dry, cracking skin. Do not put lotion between your toes.

3. Manage Calluses and Nails Professionally

Do not perform "bathroom surgery": Never try to cut, peel, or shave corns or calluses yourself. Avoid over-the-counter chemical wart or callus removers, as the acid can easily damage healthy skin and trigger a new ulcer.
 Get professional care: Have a podiatrist trim your toenails and safely reduce any calluses. Thickened skin (calluses) increases focal pressure and frequently precedes the development of a new ulcer beneath the skin.

4. Control Underlying Health Factors

Manage your blood sugar: Consistently high blood glucose levels impair your immune response, slow down healing, and worsen both nerve damage (neuropathy) and blood flow issues. Work closely with your doctor to keep your A1C within your target range.
 Quit smoking: Smoking significantly constricts your blood vessels and restricts blood circulation to your extremities, depriving your feet of the oxygen and nutrients needed to maintain healthy tissue.
 Control blood pressure and cholesterol: Managing these cardiovascular factors helps preserve the health of your arteries and ensures better blood flow to your lower legs.

5. Schedule Regular Medical Monitoring

See a podiatrist regularly: Depending on your specific risk level, you should have comprehensive diabetic foot exams every 1 to 6 months.
 Report issues immediately: If you notice any changes—such as new localized redness, unusual swelling, changes in foot temperature, or minor scrapes—contact your healthcare provider right away. Early intervention is the key to preventing a minor issue from becoming a full ulcer.

Grok 4.3:

This is general information based on established clinical guidelines and is not personalized medical advice. People with diabetes who have had a foot ulcer face a high risk of recurrence (around 40% within one year and up to 65% within three years). Consult your healthcare team—including your endocrinologist, podiatrist, and diabetes educator—for an individualized prevention plan tailored to your specific risk factors (such as neuropathy, poor circulation, foot deformities, or prior ulcer location). Early professional input can dramatically lower your chances of another ulcer.

1. Optimize blood glucose control
Keep your blood sugar levels within the targets set by your doctor (typically an HbA1c around 7% or as individually recommended). Good glycemic control supports nerve and blood vessel health, reduces inflammation, and lowers the overall risk of foot complications. Work with your diabetes care team on medication, diet, exercise, and monitoring adjustments.

2. Perform daily foot self-care
 Inspect both feet every day (use a mirror or ask for help if needed) for cuts, blisters, redness, swelling, warmth, calluses, or skin changes. Report anything unusual to your provider the same day.
 Wash feet daily in lukewarm water (test temperature with your elbow or a thermometer). Dry thoroughly, especially between the toes.
 Apply a thin layer of unscented moisturizer to dry skin on the tops and bottoms of feet—but never between the toes, as excess moisture can promote infection.
 Trim toenails straight across (not rounded or too short). If nails are thick, curved, or difficult to manage, have a podiatrist do it.
 Never walk barefoot indoors or outdoors, even for short distances. Wear clean, seamless socks daily and change them if damp.

These habits address the most common triggers: unnoticed trauma from neuropathy and skin breakdown.

3. Wear protective, well-fitting footwear at all times
Shoes are one of the most effective tools for preventing recurrence, especially if your previous ulcer was on the plantar (bottom) surface of the foot.
 Choose shoes that fit properly with adequate depth and width—no tight areas or seams that rub.
 For those with a healed plantar ulcer, your podiatrist should prescribe therapeutic footwear with demonstrated pressure-relieving properties (often custom-molded insoles or extra-depth shoes). Wear them consistently both indoors and outdoors.
 Replace shoes every 6–12 months or sooner if they show wear. Consider off-the-shelf accommodative shoes if custom options are not needed.
 Avoid high heels, pointed toes, flip-flops, and shoes without proper support.

Adherence is critical—many recurrences happen when prescribed shoes are not worn regularly.

4. Schedule regular professional foot examinations
 See a podiatrist or foot-care specialist at least every 1–3 months if you are at high risk (history of ulcer, neuropathy, or deformity), or more frequently if recommended.
 Receive an annual comprehensive diabetic foot exam (more often if risk factors are present) that includes testing for protective sensation (monofilament), circulation checks, and assessment for deformities or callus.
 Have any calluses, corns, or pre-ulcerative lesions professionally debrided—never attempt self-removal.
 Integrated foot care programs that combine education, screening, footwear, and prompt treatment of minor issues can prevent up to 75% of ulcers in high-risk individuals.

5. Address other modifiable risk factors
 Quit smoking immediately if you smoke—nicotine severely impairs circulation and healing.
 Stay physically active as approved by your doctor (walking programs or supervised exercise improve blood flow).
 Maintain a healthy weight and manage blood pressure and cholesterol to protect overall vascular health.
 If you have significant foot deformities (e.g., hammertoes, bunions, or tight Achilles tendon) that caused pressure points, discuss surgical options such as tendon lengthening or joint procedures with a specialist—only after non-surgical measures fail.

6. Seek prompt care for any foot problem
Even a minor blister or cut can progress rapidly in diabetes. Contact your provider right away for redness, drainage, odor, fever, or any change in foot appearance. Early intervention prevents escalation to a new ulcer.

Additional support
Structured education programs (often delivered by diabetes educators or podiatry teams) improve self-care behaviors. If depression, anxiety, or low motivation affects your ability to follow the plan, ask about psychological support—addressing these factors improves adherence and outcomes.

By combining tight glucose control, meticulous daily care, appropriate footwear, and regular professional oversight, most people can significantly reduce recurrence risk and enjoy more ulcer-free days. Work closely with your care team to build a sustainable routine and reassess it periodically. If you have specific details about your foot history or current regimen, your providers can refine these recommendations further.

Qwen3.6-Max-Preview:

Healing a diabetic foot ulcer is a significant achievement, but recurrence is common: studies show up to 40% of people develop another ulcer within a year, and up to 60% within three years. The good news is that a structured, consistent prevention plan can dramatically lower your risk. Below are evidence-based strategies aligned with current guidelines from the International Working Group on the Diabetic Foot (IWGDF) and the American Diabetes Association (ADA).

1. Daily Foot Care & Self-Monitoring
 Inspect both feet every day: Check tops, bottoms, sides, heels, and between toes. Use a handheld mirror, phone camera, or ask a partner if visibility is limited.
 Wash & dry carefully: Use lukewarm water and mild soap. Dry thoroughly, especially between toes, to prevent fungal growth.
 Moisturize daily: Apply urea-based or diabetic-safe lotion to prevent cracking, but avoid between toes.
 Nail & callus care: Trim nails straight across and file edges. Never cut calluses, use chemical removers, or perform "bathroom surgery." Have a podiatrist manage thick calluses or ingrown nails.
 Consider foot temperature monitoring: For high-risk patients, daily infrared thermometry can detect inflammation 2–4 days before skin breakdown. A consistent difference of >4°F (2.2°C) between matching spots on each foot warrants contacting your care team.

2. Proper Footwear & Pressure Management
 Never walk barefoot, even indoors or on carpet.
 Wear prescription diabetic shoes and custom insoles if prescribed. These redistribute pressure and reduce recurrence risk by up to 50%.
 Check inside shoes daily for pebbles, worn linings, loose stitches, or moisture.
 Break in new shoes gradually: Start with 1–2 hours/day and inspect feet afterward.
 Replace footwear regularly: Insoles typically last 3–6 months; shoes 6–12 months, depending on use and wear patterns. Follow your podiatrist's schedule.
 Continue offloading as directed: Even after healing, high-pressure areas remain vulnerable. Custom orthotics, silicone toe sleeves, or felted foam padding may be recommended long-term.

3. Blood Sugar & Systemic Health Management
 Maintain target glucose levels: Consistent glycemic control preserves nerve function, improves circulation, and strengthens skin integrity. Work with your endocrinologist or diabetes educator on a sustainable plan.
 Manage blood pressure & cholesterol: Poor vascular health significantly increases ulcer risk and delays healing.
 Quit smoking: Smoking impairs microcirculation and tissue oxygenation. Cessation is one of the most impactful steps for foot health.
 Stay hydrated & eat for skin/nerve health: Adequate protein, vitamins C/E, zinc, and omega-3s support tissue resilience.

4. Professional Care & Follow-Up
 See a podiatrist or foot specialist every 1–3 months if you're high-risk (prior ulcer, neuropathy, foot deformity, or peripheral artery disease).
 Get a comprehensive foot exam at least yearly: Should include monofilament testing, pulse checks, gait/pressure assessment, and footwear evaluation.
 Address structural issues early: Bunions, hammertoes, limited joint mobility, or Charcot changes create pressure points. Custom orthotics, physical therapy, or minor corrective procedures can prevent recurrence.
 Ask about preventive interventions: Some patients benefit from Achilles tendon lengthening, metatarsal head resection, or injectable fillers to offload high-risk areas.

5. Safe Activity & Lifestyle Habits
 Choose low-impact exercise: Swimming, cycling, seated strength training, or water aerobics improve circulation without excessive foot stress.
 Avoid prolonged standing or repetitive pressure: Shift weight frequently, use anti-fatigue mats, and take sitting breaks.
 Maintain a healthy weight: Reduces mechanical load on the feet and improves metabolic control.

When to Contact Your Care Team Immediately
 New redness, swelling, warmth, or drainage
 Blisters, cuts, scrapes, or ingrown toenails
 Sudden changes in foot shape or arch collapse
 Any area that feels "different," even if painless (neuropathy can mask injury)
 Fever or chills with foot changes

Important Note
This guidance is educational and should complement, not replace, personalized advice from your diabetes care team. A multidisciplinary approach (podiatrist, endocrinologist, wound care specialist, diabetes educator, and vascular provider if needed) offers the strongest protection against recurrence.

You've already done the hard work of healing. With consistent daily habits, proper footwear, and regular professional support, you can significantly reduce your risk and protect your long-term mobility and quality of life. If you'd like, I can help you create a simple daily/weekly foot care checklist or discuss how to talk to your care team about preventive footwear or temperature monitoring.
